# Comparative genomic analysis of eutherian fibroblast growth factor genes

**DOI:** 10.1186/s12864-020-06958-4

**Published:** 2020-08-05

**Authors:** Marko Premzl

**Affiliations:** The Australian National University Alumni, 4 Kninski trg Sq., Zagreb, Croatia

**Keywords:** Gene annotations, Eutheria, Molecular evolution, Phylogenetic analysis, RRID:SCR_014401

## Abstract

**Background:**

The eutherian fibroblast growth factors were implicated as key regulators in developmental processes. However, there were major disagreements in descriptions of comprehensive eutherian fibroblast growth factors gene data sets including either 18 or 22 homologues. The present analysis attempted to revise and update comprehensive eutherian fibroblast growth factor gene data sets, and address and resolve major discrepancies in their descriptions using eutherian comparative genomic analysis protocol and 35 public eutherian reference genomic sequence data sets.

**Results:**

Among 577 potential coding sequences, the tests of reliability of eutherian public genomic sequences annotated most comprehensive curated eutherian third-party data gene data set of fibroblast growth factor genes including 267 complete coding sequences. The present study first described 8 superclusters including 22 eutherian fibroblast growth factor major gene clusters, proposing their updated classification and nomenclature.

**Conclusions:**

The integrated gene annotations, phylogenetic analysis and protein molecular evolution analysis argued that comprehensive eutherian fibroblast growth factor gene data set classifications included 22 rather than 18 homologues.

## Background

The eutherian fibroblast growth factors or FGFs were implicated as key developmental regulators [1–3]. First, the 15 paradigmatic paracrine or canonical fibroblast growth factors FGF1–10, FGF16–18, FGF20 and FGF22 were described as ligands to single-chain receptor tyrosine kinases named FGF receptors or FGFRs [2–11]. After paracrine FGF ligand and heparan sulphate glycosaminoglycan binding, the dimerized FGFRs become activated through autophosphorylation, interacting with cytosolic adaptor proteins and intracellular signaling cascades. Such transmembrane signal transduction was implicated in regulation of embryogenesis, implantation, gastrulation, body plan formation, branching morphogenesis and organogenesis, as well as in pathogeneses of human hereditary diseases including deafness, Kallmann syndrome, lacrimo-auriculo-dentodigital syndrome and different skeletal syndromes, and in tumorigenesis. Second, there were 3 endocrine fibroblast growth factors FGF19, FGF21 and FGF23 binding FGFRs and klotho protein cofactors [2, 3, 7, 12]. The endocrine FGFs were implicated in metabolism regulation including phosphate and vitamin D homeostasis, cholesterol and bile acid homeostasis and glucose and lipid homeostasis, as well as in pathogenesis of autosomal dominant hypophosphataemic rickets. Third, the 4 intracellular fibroblast growth factors named fibroblast homologous factors included FGF11 or FHF3, FGF12 or FHF1, FGF13 or FHF2 and FGF14 or FGF4 [1, 3, 13–16]. The intracellular FGFs were described as regulators of nervous system development and function including integration and encoding of complex synaptic inputs into action potential outputs in central nervous system neurons, and implicated in pathogenesis of early-onset spinocerebellar ataxia. The molecular evolution and protein structure analyses indicated that eutherian FGFs folded into β-trefoil protein tertiary structures including 11 or 12 β-strands [1–3, 7, 12, 13, 17–28]. However, there were major disagreements in descriptions of comprehensive eutherian *FGF* gene data sets. Specifically, Belov and Mohammadi [2] and Beenken and Mohammadi [7] argued that bona fide eutherian FGF homologues included 18 secreted paracrine and endocrine FGFs. On the other hand, the eutherian FGF classifications by Goldfarb [1] and Ornitz and Itoh [3] included both 18 secreted FGFs and 4 intracellular FGFs.

Undoubtedly, the public eutherian reference genomic sequence data sets advanced biological and medical sciences [29–34]. Indeed, the comparative genomics momentum was maintained by considerable international efforts in production and analysis of public eutherian reference genomic sequence data sets. For example, the initial sequencing and analysis of human genome attempted to revise and update human genes, and uncover potential new drugs, drug targets and molecular markers in medical diagnostics [35, 36]. Nevertheless, due to the incompleteness of eutherian reference genomic sequence assemblies [35, 37] and potential genomic sequence errors [36, 38], future updates and revisions of public eutherian reference genomic sequence data sets were expected. Inevitably, the potential genomic sequence errors including analytical and bioinformatical errors (erroneous gene annotations, genomic sequence misassemblies) and Sanger DNA sequencing method errors (artefactual nucleotide deletions, insertions and substitutions) could compromise unquestionable utility of public eutherian reference genomic sequence data sets. For example, Gajer et al. [39] described so-called lexicographical bias in some genomic sequence assemblers. In addition, the potential genomic sequence errors affecting phylogenetic analyses [40] were observed more frequently in reference genomic sequence assemblies including lower genomic sequence redundancies [41–43]. Thus, the eutherian comparative genomic analysis protocol was established as guidance in protection against potential genomic sequence errors in public eutherian reference genomic sequence data sets [44–46]. Using public eutherian reference genomic sequence data sets, the protocol published new test of reliability of public eutherian genomic sequences using genomic sequence redundancies, and new test of protein molecular evolution using relative synonymous codon usage statistics. The protocol revised and updated 12 eutherian gene data sets implicated in major physiological and pathological processes, including 1853 published complete coding sequences. Of note, there was positive correlation between genomic sequence redundancies of 35 public eutherian reference genomic sequence data sets respectively and published complete coding sequence numbers [46].

Therefore, the present analysis attempted to revise and update comprehensive eutherian *FGF* gene data sets, and address and resolve major disagreements in their descriptions using eutherian comparative genomic analysis protocol and 35 public eutherian reference genomic sequence data sets.

## Results

### Gene annotations

**Fig. 1:**
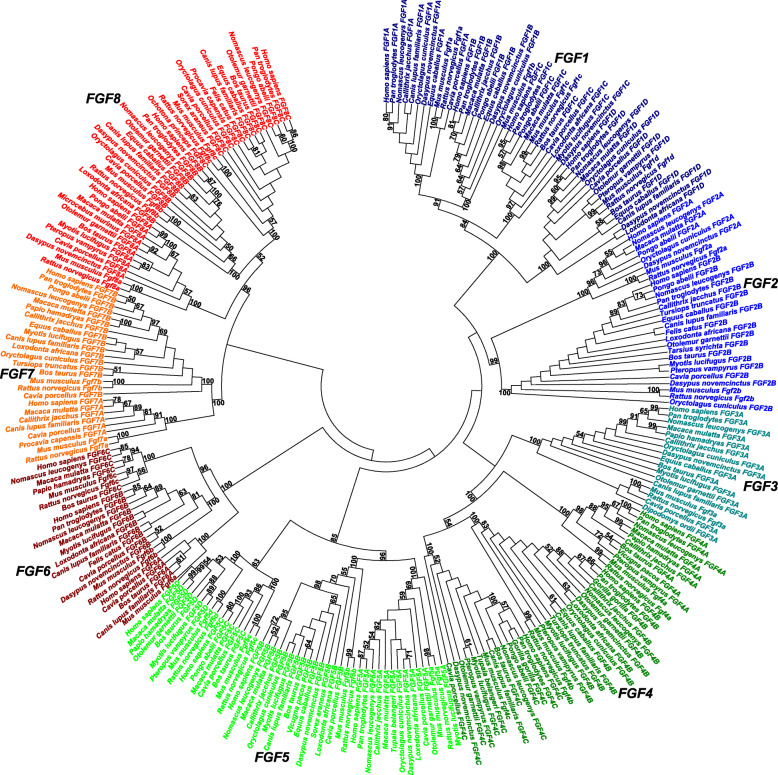
Phylogenetic analysis of eutherian fibroblast growth factor genes. The minimum evolution phylogenetic tree including bootstrap estimates higher than 50% after 1000 replicates was calculated using maximum composite likelihood method. The 8 major gene superclusters *FGF1*–*8* were indicated

The tests of reliability of eutherian public genomic sequences annotated 267 *FGF* complete coding sequences among 577 *FGF* potential coding sequences (Fig. 1). The most comprehensive curated eutherian *FGF* third-party data gene data set was deposited in European Nucleotide Archive under accessions: LR130242-LR130508 [47, 48] (Additional file 1).

The present study first described 8 superclusters *FGF1*–*8* including 22 major gene clusters of eutherian *FGF* genes, proposing their updated nomenclature (Fig. 1). The supercluster *FGF1* included 4 major gene clusters *FGF1A* (11 *FGF12* or *FHF1* genes), *FGF1B* (9 *FGF14* or *FGF4* genes), *FGF1C* (11 *FGF13* or *FHF2* genes) and *FGF1D* (15 *FGF11* or *FHF3* genes) (Additional file 2A-D). The supercluster *FGF2* included 2 major gene clusters *FGF2A* (8 *FGF2* genes) and *FGF2B* (20 *FGF1* genes) (Additional file 2E-F). The supercluster *FGF3* included 1 major gene cluster *FGF3A* (17 *FGF5* genes) (Additional file 2G). The supercluster *FGF4* included 3 major gene clusters *FGF4A* (11 *FGF20* genes), *FGF4B* (16 *FGF9* genes) and *FGF4C* (14 *FGF16* genes) (Additional file 2H-J). The supercluster *FGF5* included 4 major gene clusters *FGF5A* (14 *FGF10* genes), *FGF5B* (16 *FGF7* genes), *FGF5C* (7 *FGF3* genes) and *FGF5D* (9 *FGF22* genes) (Additional file 2 K-N). The supercluster *FGF6* included 3 major gene clusters *FGF6A* (5 *FGF18* genes), *FGF6B* (12 *FGF17* genes) and *FGF6C* (7 *FGF8* genes) (Additional file 2O-Q). The supercluster *FGF7* included 2 major gene clusters *FGF7A* (8 *FGF4* genes) and *FGF7B* (17 *FGF6* genes) (Additional file 2R-S). Finally, The supercluster *FGF8* included 3 major gene clusters *FGF8A* (12 *FGF19* genes), *FGF8B* (12 *FGF23* genes) and *FGF8C* (16 *FGF21* genes) (Additional file 2 T-V).

The present study included new genomics tests of contiguity of eutherian public genomic sequences that analysed numbers of coding exons in *FGF* genes and their relative orientation (Additional files 1 and 2). The analysis including 903 *FGF* coding exons indicated that there were no coding exon misassemblies among 267 eutherian genomic sequences harbouring *FGF* complete coding sequences. The eutherian *FGF* genes included either 5 coding exons (5 major gene clusters *FGF1A*-*D* and *FGF6A*) or 3 coding exons (17 other major gene clusters). The eutherian *FGF* coding exon numbers were constant within major gene clusters, and there was no evidence of differential gene expansions indicating that 22 eutherian *FGF* major gene clusters respectively included orthologues. For example, whereas the human *FGF1A* gene included 5 coding exons along 264,215 bp (Additional file 2A), human *FGF7A* gene included 3 coding exons along 1776 bp (Additional file 2R).

Therefore, the present study annotating 22 eutherian *FGF* major gene clusters agreed with Goldfarb [1] and Ornitz and Itoh [3] but disagreed with Belov and Mohammadi [2] and Beenken and Mohammadi [7].

### Phylogenetic analysis

The present minimum evolution phylogenetic tree calculations (Fig. 1) and calculations of pairwise nucleotide sequence identity patterns (Additional file 3) first classified 22 eutherian *FGF* major gene clusters among 8 superclusters *FGF1*–*8*. The clustering of major gene clusters *FGF1A*-*D* within supercluster *FGF1* agreed with subfamily *FGF11* descriptions [3, 23], Smallwood et al. [13], Ornitz and Itoh [21], subfamily *Fgf11/12/13/14* description [25] and Nam et al. [28]. The clustering of major gene clusters *FGF2A*-*B* within supercluster *FGF2* agreed with subfamily *FGF1* descriptions [3, 23], Smallwood et al. [13], Coulier et al. [17], Ornitz and Itoh [21], subfamily *Fgf1/2* description [25] and Nam et al. [28]. The supercluster *FGF3* description including 1 major gene cluster *FGF3A* agreed with Nam et al. [28] but disagreed with phylogenetic analyses of Ornitz and Itoh [3, 21], Coulier et al. [17] and Itoh and Ornitz [23, 25]. The clustering of major gene clusters *FGF4A*-*C* within supercluster *FGF4* agreed with subfamily *FGF9* descriptions [3, 23], Ornitz and Itoh [21] and subfamily *Fgf9/16/20* description [25] but disagreed with Nam et al. [28]. The clustering of major gene clusters *FGF5A*-*D* within supercluster *FGF5* disagreed with phylogenetic analyses of Ornitz and Itoh [3, 21], Itoh and Ornitz [23, 25] and Nam et al. [28]. The clustering of major gene clusters *FGF6A*-*C* within supercluster *FGF6* agreed with subfamily *FGF8* descriptions [3, 23], Ornitz and Itoh [21], subfamily *Fgf8/17/18* description [25] and Nam et al. [28]. The clustering of major gene clusters *FGF7A*-*B* within supercluster *FGF7* agreed with Smallwood et al. [13], Coulier et al. [17], Ornitz and Itoh [21] and Nam et al. [28] but disagreed with Ornitz and Itoh [3] and Itoh and Ornitz [23, 25]. Finally, the clustering of major gene clusters *FGF8A*-*C* within supercluster *FGF8* agreed with Ornitz and Itoh [21] but disagreed with Ornitz and Itoh [3], Itoh and Ornitz [23, 25] and Nam et al. [28].

Indeed, the calculations of pairwise nucleotide sequence identity patterns confirmed present phylogenetic classification of eutherian *FGF* genes (Additional file 3). The eutherian *FGF* gene data set included average pairwise nucleotide sequence identity *ā* = 0,3 (*a*_max_ = 1, *a*_min_ = 0,115, *ā*_ad_ = 0,094) [1–3, 7, 12, 13, 17, 21, 23, 25–28]. Among 22 eutherian *FGF* major gene clusters respectively, there were nucleotide sequence identity patterns of very close eutherian orthologues (*FGF1A*-*B*, *FGF4B*), close eutherian orthologues (*FGF1C*-*D*, *FGF2A-B*, *FGF4A*, *FGF4C*, *FGF5B*, *FGF6A*, *FGF7B*), typical eutherian orthologues (*FGF3A*, *FGF5A*, *FGF5C*-*D*, *FGF6B*-*C*, *FGF7A*, *FGF8A*, *FGF8C*) and distant eutherian orthologues (*FGF8B*). In comparisons between eutherian *FGF* major gene clusters within superclusters, there were nucleotide sequence identity patterns of very close eutherian homologues (superclusters *FGF1*–*2*, *FGF4*, *FGF7*), very close and close eutherian homologues (supercluster *FGF6*), close and typical eutherian homologues (supercluster *FGF5*) and typical eutherian homologues (supercluster *FGF8*). Finally, in comparisons between eutherian *FGF* major gene clusters between superclusters, there were nucleotide sequence identity patterns of close, typical, distant and very distant eutherian homologues.

Therefore, the present phylogenetic analysis proposed updated classification of eutherian *FGF* genes.

### Protein molecular evolution analysis

**Fig. 2:**
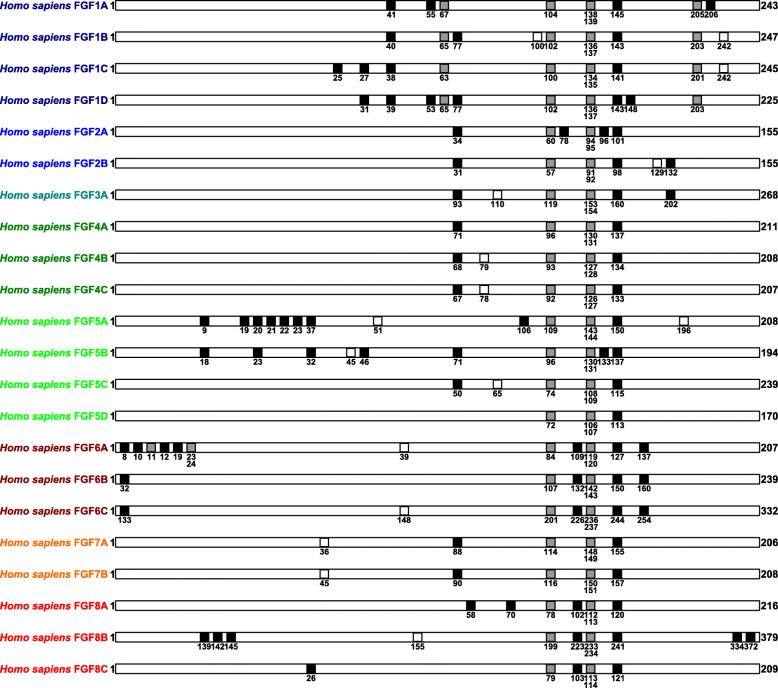
Major landmarks in eutherian fibroblast growth factor protein sequence alignments. The black squares labelled common cysteine amino acid residues. The grey squares labelled common exon-intron splice site amino acid sites. The white squares labelled common N-glycosylation sites. The numbers indicated numbers of amino acid residues

The protein molecular evolution analysis used protein primary structure features as major alignment landmarks in eutherian FGF protein amino acid sequence alignments, including common cysteine amino acid residues, common exon-intron splice site amino acid sites and common predicted N-glycosylation sites (Fig. 2) (Additional file 4). There were between 1 and 9 common cysteine amino acid residues included among eutherian FGF major protein clusters respectively. For example, whereas the major protein cluster FGF5D included 1 common cysteine amino acid residue, major protein cluster FGF5A included 9 common cysteine amino acid residues. There were either 4 common exon-intron splice site amino acid sites (5 major protein clusters FGF1A-D and FGF6A) or 2 common exon-intron splice site amino acid sites (17 other major protein clusters) among eutherian FGF major protein clusters respectively. Finally, there were between 0 and 2 common predicted N-glycosylation sites among eutherian FGF major protein clusters respectively.

**Fig. 3:**
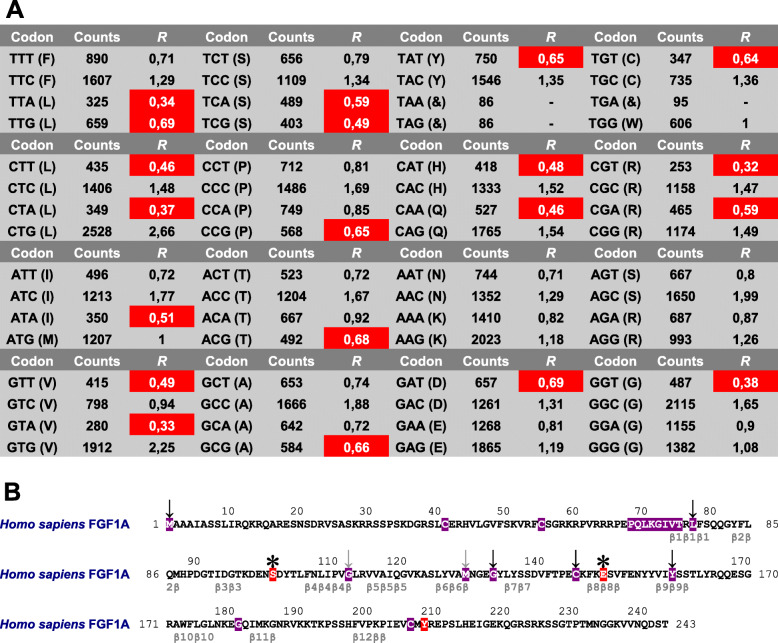
Tests of protein molecular evolution of eutherian fibroblast growth factors. **a** Relative synonymous codon usage statistics of eutherian *FGF* gene data set. The not preferable amino acid codons were indicated by white letters on red backgrounds. Counts, observed amino acid codon counts; *R*, relative synonymous codon usage statistics; &, stop codons. **b** Reference human FGF1A protein amino acid sequence. The 19 invariant amino acid sites were shown using white letters on violet backgrounds. Whereas the 5 amino acid sites that were invariant among 22 FGF major protein clusters were indicated by black arrows (except that M1 was invariant among 266 FGF protein primary structures), grey arrows indicated 2 amino acid sites that were invariant among 21 FGF major protein clusters respectively. The 3 forward amino acid sites were shown using white letters on red backgrounds. The stars labelled 2 forward amino acid sites described among 22 FGF major protein clusters. The positions of 12 β-strands implicated in β-trefoil protein tertiary structure were indicated below reference human FGF1A protein primary structure [22, 24]

Next, the tests of protein molecular evolution first calculated relative synonymous codon usage statistics (*R*) of eutherian *FGF* gene data set using 267 *FGF* complete coding sequences (Additional file 4), and described 20 amino acid codons including *R ≤* 0,7 as not preferable amino acid codons (Fig. 3a). The tests used human FGF1A protein primary structure as reference protein amino acid sequence (Fig. 3b). Among 243 human FGF1A protein amino acid residues, the tests of protein molecular evolution described 19 invariant amino acid sites, viz.: M1, C41, C55, P68, Q69, L70, K71, G72, I73, V74, T75, L77, G112, M129, G133, C145, Y159, G181 and C206, as well as 3 forward amino acid sites S101, E149 and Y208. First, the human FGF1A amino acid sites M1, L77, G133, C145 and Y159 were invariant among 267 eutherian FGF protein primary structures (except that M1 was invariant among 266 FGF protein primary structures). For example, the human FGF1A invariant amino acid sites L77, G133 and C145 were described by Goetz et al. [12, 24], Smallwood et al. [13], Coulier et al. [17], Venkataraman et al. [18], Plotnikov et al. [19] and Olsen et al. [22]. Furthermore, the human FGF1A amino acid sites G112 and M129 respectively were invariant among 21 eutherian FGF major protein clusters. For example, the human FGF1A amino acid site G112 was homologous to human FGF2B amino amino acid site G67 that was implicated in interactions between FGF2B ligand and FGFR2 receptor [19, 20]. In addition, the human FGF1A amino acid site G181 that was invariant among 7 eutherian FGF1–7 protein superclusters was described as first glycine amino acid residue in paracrine FGF glycine box protein amino acid sequence motif G-x(4)-G-x(2)-S/T [2]. The human FGF1A amino acid sites P68, Q69, L70, K71, G72, I73, V74 and T75 were invariant among 4 eutherian FGF1A-D major protein clusters. For example, the human FGF1A amino acid sites K71 and I73 were described as residues engaged in voltage-gated sodium channel binding [24]. Finally, the human FGF1A forward amino acid sites S101 and E149 were described among 267 eutherian FGF protein primary structures, and forward amino acid site Y208 was described among 2 eutherian FGF1–2 protein superclusters. For example, the human FGF1A forward amino acid site E149 was homologous to human FGF2A amino amino acid site E105 that was implicated in hydrogen bonding between FGF2A ligand and D3 domain of FGFR2 receptor [19, 26].

Therefore, the tests of protein molecular evolution using relative synonymous codon usage statistics described amino acid sites implicated as critical in FGF protein secondary, tertiary and quaternary structural features.

## Discussion

The major disagreements in descriptions of comprehensive eutherian *FGF* gene data sets included classifications of either 18 *FGF* genes [2, 7] or 22 *FGF* genes [1, 3]. The present analysis attempted to address and resolve these discrepancies using eutherian comparative genomic analysis protocol and public eutherian reference genomic sequence data sets [29–36, 44–46]. The advantages of eutherian reference genomic sequence data sets were well established phylogeny [29, 30, 34] and calibrated taxon sampling including genomic sequence redundancies that were applicable in tests of reliability of eutherian public genomic sequences [31–33]. Therefore, the tests of reliability of eutherian public genomic sequences annotated most comprehensive curated eutherian third-party data gene data set of *FGF* genes that included 267 complete coding sequences among 577 potential coding sequences. Second, the present study first described 8 superclusters of eutherian *FGF* genes that included 22 major gene clusters, proposing their updated nomenclature. Third, the new genomics tests of contiguity of eutherian public genomic sequences included 903 coding exons, and annotated either 3 or 5 coding exons in eutherian *FGF* genes including no evidence of differential gene expansions. Fourth, the present phylogenetic analysis proposed updated classification of eutherian *FGF* genes. Finally, the tests of protein molecular evolution using relative synonymous codon usage statistics described 19 invariant amino acid sites and 3 forward amino acid sites in reference human FGF1A protein primary structure, including amino acid residues described as critical in FGF protein secondary, tertiary and quaternary structural features. In conclusion, the present comparative genomic analysis integrating gene annotations, phylogenetic analysis and protein molecular evolution analysis argued that 22 *FGF* genes [1, 3], rather than 18 *FGF* genes [2, 7], were included in comprehensive eutherian *FGF* gene data set classifications.

## Methods

### Eutherian comparative genomic analysis protocol

**Fig. 4:**
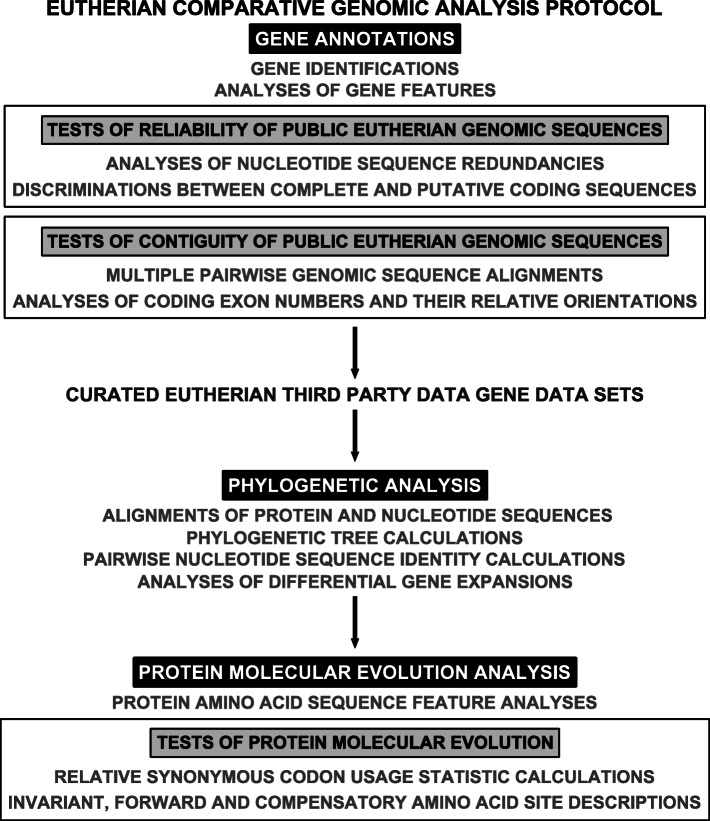
Eutherian comparative genomic analysis protocol flowchart

The eutherian comparative genomic analysis protocol RRID:SCR_014401 integrated gene annotations, phylogenetic analysis and protein molecular evolution analysis with tests of reliability of eutherian public genomic sequences, tests of contiguity of eutherian public genomic sequences and tests of protein molecular evolution into one framework of eutherian gene descriptions (Fig. 4) [44–46].

### Gene annotations

The protocol used gene identifications in 35 public genomic sequence assemblies, tests of reliability of eutherian public genomic sequences and new genomics tests of contiguity of eutherian public genomic sequences in eutherian *FGF* gene annotations. First, the sequence alignment editor BioEdit 7.0.5.3 was used in all analyses and manipulations of nucleotide and protein sequences [49]. The National Center for Biotechnology Information (NCBI) BLAST Genomes was used in identifications of *FGF* potential coding sequences in eutherian reference genomic sequence data sets [50–53], as well as Ensembl genome browser BLAST or BLAT tools [54, 55]. Second, the tests of reliability of eutherian public genomic sequences used *FGF* potential coding sequences. Using BLASTN and primary Sanger DNA sequencing information deposited in NCBI Trace Archive [51, 56], the first test steps analysed nucleotide sequence coverages of each *FGF* potential coding sequence. If consensus trace sequence coverages were available for every nucleotide, the protocol described *FGF* potential coding sequences as *FGF* complete coding sequences. However, if consensus trace sequence coverages were not available for every nucleotide, the protocol described *FGF* potential coding sequences as *FGF* putative coding sequences (not used in analyses). The protocol then deposited *FGF* complete coding sequences in European Nucleotide Archive as curated third-party data gene information [57–60]. The protocol used guidelines of human gene nomenclature [61] and guidelines of mouse gene nomenclature [62] in updated eutherian *FGF* gene classification and nomenclature. Third, the protocol used new genomics tests of contiguity of eutherian public genomic sequences in eutherian *FGF* gene annotations. Using multiple pairwise genomic sequence alignments of eutherian genomic sequences harbouring *FGF* complete coding sequences, the tests of contiguity analysed numbers of coding exons in *FGF* genes and their relative orientation. The tests discriminated between *FGF* genes not including coding exon misassemblies in eutherian genomic sequence assemblies and *FGF* genes including coding exon misassemblies. The tests used mVISTA AVID option in multiple pairwise genomic sequence alignments, using default settings [63, 64]. The empirically determined cut-offs of detection of common genomic sequence regions in pairwise alignments with base sequences (*Homo sapiens*) were 95% nucleotide sequence identity along 100 bp (*Pan troglodytes*, *Gorilla gorilla*), 90% along 100 bp (*Pongo abelii*, *Nomascus leucogenys*), 85% along 100 bp (*Macaca mulatta*, *Papio hamadryas*), 80% along 100 bp (*Callithrix jacchus*), 75% along 100 bp (*Tarsius syrichta*, *Microcebus murinus*, *Otolemur garnettii*), 65% along 100 bp (Rodentia) or 70% along 100 bp in other pairwise alignments [44–46]. In preparatory steps of multiple pairwise genomic sequence alignments, the protocol did not include masking of transposable elements in genomic sequences harbouring *FGF* complete coding sequences.

### Phylogenetic analysis

The protocol used protein and nucleotide sequence alignments, calculations of phylogenetic trees, calculations of pairwise nucleotide sequence identities and analysis of differential gene expansions in phylogenetic analysis of eutherian *FGF* gene data set. First, using BioEdit 7.0.5.3, the protocol translated *FGF* complete coding sequences, and aligned them at amino acid level using ClustalW implemented in BioEdit 7.0.5.3. After manual corrections of FGF protein primary structure alignments, the *FGF* nucleotide sequence alignments were prepared accordingly. Second, the MEGA 6.06 program was used in phylogenetic tree calculations, using minimum evolution method that was applicable in phylogenetic analysis of very close, close, typical, distant and very distant eutherian *FGF* homologues (default settings, except gaps/missing data treatment = pairwise deletion and maximum composite likelihood method) [65, 66]. Third, the protocol used BioEdit 7.0.5.3 in calculations of pairwise nucleotide sequence identities of *FGF* complete coding sequences that were used in statistical analyses. The Microsoft Office Excel common statistical functions were used in calculations of pairwise nucleotide sequence identity patterns of eutherian *FGF* gene data set. Using pairwise nucleotide sequence identities of *FGF* nucleotide sequence alignments including 267 *FGF* complete coding sequences, the protocol calculated average pairwise nucleotide sequence identities (*ā*) and their average absolute deviations (*ā*_ad_), and largest (*a*_max_) and smallest (*a*_min_) pairwise nucleotide sequence identities.

### Protein molecular evolution analysis

The protocol used analysis of FGF protein amino acid sequence features and tests of protein molecular evolution integrating patterns of *FGF* nucleotide sequence similarities with FGF protein primary structures in protein molecular evolution analysis. The protocol used complete *FGF* nucleotide sequence alignments in tests of protein molecular evolution, including 267 *FGF* complete coding sequences and 58,533 codons. Among eutherian *FGF* complete coding sequences, the average number of codons was 219. Using MEGA 6.06, the relative synonymous codon usage statistics were calculated as ratios between observed and expected amino acid codon counts (*R* = Counts / Expected counts). The protocol then described 20 amino acid codons including *R ≤* 0,7 as not preferable amino acid codons, viz.: TTA, TTG, CTT, CTA, ATA, GTT, GTA, TCA, TCG, CCG, ACG, GCG, TAT, CAT, CAA, GAT, TGT, CGT, CGA, GGT (Fig. 3b). Finally, the protocol described reference human FGF1A protein sequence amino acid sites as invariant amino acid sites (invariant alignment positions), forward amino acid sites (variant alignment positions that did not include amino acid codons with *R ≤* 0,7) or compensatory amino acid sites (variant alignment positions that included amino acid codons with *R ≤* 0,7).

## Supplementary information

**Additional file 1.** Third-party data gene data set of eutherian fibroblast growth factor genes.

**Additional file 2 **Multiple pairwise genomic sequence alignments of eutherian fibroblast growth factor genes. The *FGF* coding exon sequence regions in base sequences (*Homo sapiens*) were displayed as indigo rectangles, and grey arrows indicated their relative orientation (top). The genomic sequence regions including sequence identity levels above empirical cut-offs of detection of common genomic sequence regions were shown accordingly in multiple pairwise alignments.

**Additional file 3.** Pairwise nucleotide sequence identity patterns of eutherian fibroblast growth factor genes.

**Additional file 4.** Protein amino acid sequence alignments of eutherian fibroblast growth factors. The amino acid positions were labelled using white letters on black background (100% sequence identity level), white letters on dark grey background (≥ 75% sequence identity level) or black letters on grey background (≥50% sequence identity level). The 19 invariant amino acid sites were shown using white letters on violet backgrounds and 3 forward amino acid sites were shown using white letters on red backgrounds in reference human FGF1A protein primary structure (top). The stop codons were indicated by &s.

## Data Availability

The original curated third-party data gene data set including 267 eutherian *FGF* complete coding sequences was deposited in European Nucleotide Archive under accessions: LR130242-LR130508 [47]. The present study was registered under NCBI BioProject entitled “Curated eutherian third-party data gene data sets” (NCBI BioProject accession: PRJNA453891; NCBI BioSample accessions: SAMN09005565-SAMN09005599) [48, 67]. The public eutherian reference genomic sequence data sets (Additional file 1) were available in NCBI GenBank [51, 52] and Ensembl [54].

## Abbreviations

FGF: Fibroblast growth factor
*FGF1*–*8*: Eutherian fibroblast growth factor gene superclusters
