# Supplementary material for: Comparative genomic analysis of eutherian fibroblast growth factor genes
Source: BMC Genomics. 2020 Aug 5;21:542. doi: 10.1186/s12864-020-06958-4 (PMC7430813; doi:10.1186/s12864-020-06958-4)
Supplement: Supplementary file 1 — Additional file 1. Third-party data gene data set of eutherian fibroblast growth factor genes. [file 12864_2020_6958_MOESM1_ESM.pdf]

**Additional File 1:** Third-party data gene data set of eutherian fibroblast growth factor genes.

| Superordinal clade | Order | Species [a] | Genome assembly | Gene number | Gene [b] | Genomic coordinates [c] | GenBank |
|---|---|---|---|---|---|---|---|
| Euarchontoglires | Primates | Human | GCF_000001405.38 | 22 | *FGF1A* (*FGF12*) | exon 1: ch.3: 192408025-192408223 bp<br>exon 2: ch.3: 192360428-192360538 bp<br>exon 3: ch.3: 192335361-192335464 bp<br>exon 4: ch.3: 192170458-192170656 bp<br>exon 5: ch.3: 192144009-192144127 bp | LR130242 |
| | | | | | *FGF1B* (*FGF14*) | exon 1: ch.13: 101916453-101916645 bp<br>exon 2: ch.13: 101875186-101875296 bp<br>exon 3: ch.13: 101868725-101868828 bp<br>exon 4: ch.13: 101726612-101726810 bp<br>exon 5: ch.13: 101722831-101722967 bp | LR130243 |
| | | | | | *FGF1C* (*FGF13*) | exon 1: ch.X: 138710817-138711003 bp<br>exon 2: ch.X: 138708818-138708928 bp<br>exon 3: ch.X: 138702984-138703087 bp<br>exon 4: ch.X: 138635457-138635655 bp<br>exon 5: ch.X: 138632850-138632986 bp | LR130244 |
| | | | | | *FGF1D* (*FGF11*) | exon 1: ch.17: 7439621-7439813 bp<br>exon 2: ch.17: 7441471-7441581 bp<br>exon 3: ch.17: 7441776-7441879 bp<br>exon 4: ch.17: 7442594-7442792 bp<br>exon 5: ch.17: 7443076-7443146 bp | LR130245 |
| | | | | | *FGF2A* (*FGF2*) | exon 1: ch.4: 122827175-122827352 bp<br>exon 2: ch.4: 122876321-122876424 bp<br>exon 3: ch.4: 122892211-122892396 bp | LR130246 |
| | | | | | *FGF2B* (*FGF1*) | exon 1: ch.5: 142613959-142614127 bp<br>exon 2: ch.5: 142600702-142600805 bp<br>exon 3: ch.5: 142595290-142595484 bp | LR130247 |
| | | | | | *FGF3A* (*FGF5*) | exon 1: ch.4: 80266825-80267179 bp<br>exon 2: ch.4: 80274909-80275012 bp<br>exon 3: ch.4: 80286325-80286672 bp | LR130248 |
| | | | | | *FGF4A* (*FGF20*) | exon 1: ch.8: 17001747-17002032 bp<br>exon 2: ch.8: 16995655-16995758 bp<br>exon 3: ch.8: 16993072-16993317 bp | LR130249 |

| | | | | | | | |
|---|---|---|---|---|---|---|---|
| | | | | | *FGF4B* (*FGF9*) | exon 1: ch.13: 21671913-21672189 bp<br>exon 2: ch.13: 21681042-21681145 bp<br>exon 3: ch.13: 21701190-21701435 bp | LR130250 |
| | | | | | *FGF4C* (*FGF16*) | exon 1: ch.X: 77447675-77447948 bp<br>exon 2: ch.X: 77454157-77454260 bp<br>exon 3: ch.X: 77456277-77456522 bp | LR130251 |
| | | | | | *FGF5A* (*FGF10*) | exon 1: ch.5: 44388358-44388682 bp<br>exon 2: ch.5: 44310427-44310530 bp<br>exon 3: ch.5: 44304995-44305192 bp | LR130252 |
| | | | | | *FGF5B* (*FGF7*) | exon 1: ch.15: 49424298-49424583 bp<br>exon 2: ch.15: 49483151-49483254 bp<br>exon 3: ch.15: 49484310-49484504 bp | LR130253 |
| | | | | | *FGF5C* (*FGF3*) | exon 1: ch.11: 69818714-69818933 bp<br>exon 2: ch.11: 69816320-69816423 bp<br>exon 3: ch.11: 69810305-69810700 bp | LR130254 |
| | | | | | *FGF5D* (*FGF22*) | exon 1: ch.19: 639926-640139 bp<br>exon 2: ch.19: 643235-643338 bp<br>exon 3: ch.19: 643410-643604 bp | LR130255 |
| | | | | | *FGF6A* (*FGF18*) | exon 1: ch.5: 171420200-171420231 bp<br>exon 2: ch.5: 171420407-171420443 bp<br>exon 3: ch.5: 171436093-171436273 bp<br>exon 4: ch.5: 171449147-171449253 bp<br>exon 5: ch.5: 171456539-171456805 bp | LR130256 |
| | | | | | *FGF6B* (*FGF17*) | exon 1: ch.8: 22045973-22046291 bp<br>exon 2: ch.8: 22046527-22046633 bp<br>exon 3: ch.8: 22047956-22048249 bp | LR130257 |
| | | | | | *FGF6C* (*FGF8*) | exon 1: ch.10: 101774732-101775332 bp<br>exon 2: ch.10: 101771463-101771569 bp<br>exon 3: ch.10: 101770329-101770619 bp | LR130258 |
| | | | | | *FGF7A* (*FGF4*) | exon 1: ch.11: 69774745-69775084 bp<br>exon 2: ch.11: 69774024-69774127 bp<br>exon 3: ch.11: 69773309-69773485 bp | LR130259 |
| | | | | | *FGF7B* (*FGF6*) | exon 1: ch.12: 4445225-4445570 bp<br>exon 2: ch.12: 4444133-4444236 bp<br>exon 3: ch.12: 4434215-4434391 bp | LR130260 |

| | | | | | | | |
|---|---|---|---|---|---|---|---|
| | | | | | *FGF8A* (*FGF19*) | exon 1: ch.11: 69703645-69703876 bp<br>exon 2: ch.11: 69703261-69703364 bp<br>exon 3: ch.11: 69699262-69699576 bp | LR130261 |
| | | | | | *FGF8B* (*FGF23*) | exon 1: ch.12: 4379372-4379966 bp<br>exon 2: ch.12: 4372594-4372697 bp<br>exon 3: ch.12: 4370343-4370783 bp | LR130262 |
| | | | | | *FGF8C* (*FGF21*) | exon 1: ch.19: 48756237-48756471 bp<br>exon 2: ch.19: 48756926-48757029 bp<br>exon 3: ch.19: 48757930-48758220 bp | LR130263 |
| | | Common chimpanzee | GCF_000001515.7 | 14 | *FGF1A* | exon 1: ch.3: 196719823-196720021 bp<br>exon 2: ch.3: 196672237-196672347 bp<br>exon 3: ch.3: 196647378-196647481 bp<br>exon 4: ch.3: 196481042-196481240 bp<br>exon 5: ch.3: 196460590-196460708 bp | LR130264 |
| | | | | | *FGF1B* | exon 1: ch.13: 87673052-87673244 bp<br>exon 2: ch.13: 87630407-87630517 bp<br>exon 3: ch.13: 87623977-87624080 bp<br>exon 4: ch.13: 87480245-87480443 bp<br>exon 5: ch.13: 87476488-87476624 bp | LR130265 |
| | | | | | *FGF1C* | exon 1: ch.X: 138593979-138594165 bp<br>exon 2: ch.X: 138591980-138592090 bp<br>exon 3: ch.X: 138586148-138586251 bp<br>exon 4: ch.X: 138518290-138518488 bp<br>exon 5: ch.X: 138515688-138515824 bp | LR130266 |
| | | | | | *FGF1D* | exon 1: ch.17: 7701340-7701532 bp<br>exon 2: ch.17: 7703185-7703295 bp<br>exon 3: ch.17: 7703490-7703593 bp<br>exon 4: ch.17: 7704314-7704512 bp<br>exon 5: ch.17: 7704796-7704866 bp | LR130267 |
| | | | | | *FGF2B* | exon 1: ch.5: 142674796-142674964 bp<br>exon 2: ch.5: 142661574-142661677 bp<br>exon 3: ch.5: 142656443-142656637 bp | LR130268 |
| | | | | | *FGF3A* | exon 1: ch.4: 50206142-50206496 bp<br>exon 2: ch.4: 50198257-50198360 bp<br>exon 3: ch.4: 50186600-50186947 bp | LR130269 |
| | | | | | *FGF4A* | exon 1: ch.8: 16171052-16171337 bp | LR130270 |

| | | | | | | | |
|---|---|---|---|---|---|---|---|
| | | | | | | exon 2: ch.8: 16164948-16165051 bp<br>exon 3: ch.8: 16162368-16162613 bp | |
| | | | | | *FGF4B* | exon 1: ch.13: 6251322-6251598 bp<br>exon 2: ch.13: 6260450-6260553 bp<br>exon 3: ch.13: 6280542-6280787 bp | LR130271 |
| | | | | | *FGF4C* | exon 1: ch.X: 76929162-76929435 bp<br>exon 2: ch.X: 76935644-76935747 bp<br>exon 3: ch.X: 76937767-76938012 bp | LR130272 |
| | | | | | *FGF5A* | exon 1: ch.5: 69757406-69757730 bp<br>exon 2: ch.5: 69834979-69835082 bp<br>exon 3: ch.5: 69840325-69840522 bp | LR130273 |
| | | | | | *FGF6B* | exon 1: ch.8: 21347270-21347588 bp<br>exon 2: ch.8: 21347824-21347930 bp<br>exon 3: ch.8: 21349269-21349562 bp | LR130274 |
| | | | | | *FGF7B* | exon 1: ch.12: 4645274-4645619 bp<br>exon 2: ch.12: 4644182-4644285 bp<br>exon 3: ch.12: 4634244-4634420 bp | LR130275 |
| | | | | | *FGF8B* | exon 1: ch.12: 4579126-4579720 bp<br>exon 2: ch.12: 4572387-4572490 bp<br>exon 3: ch.12: 4570136-4570576 bp | LR130276 |
| | | | | | *FGF8C* | exon 1: ch.19: 50936673-50936907 bp<br>exon 2: ch.19: 50937290-50937393 bp<br>exon 3: ch.19: 50938300-50938590 bp | LR130277 |
| | | | | | putative | exon 1: ch.4: 125731064-125731241 bp<br>exon 2: ch.4: 125780211-125780314 bp<br>exon 3: ch.4: 125795786-125795971 bp | - |
| | | | | | putative | exon 1: ch.5: 171962154-171962185 bp<br>exon 2: ch.5: 171962361-171962397 bp<br>exon 3: ch.5: 171977747-171977927 bp<br>exon 4: ch.5: 171990807-171990913 bp<br>exon 5: ch.5: 171998242-171998508 bp | - |
| | | | | | putative | exon 1: ch.10: 103224629-103225229 bp<br>exon 2: ch.10: 103221357-103221463 bp<br>exon 3: ch.10: 103220224-103220514 bp | - |
| | | | | | putative | exon 1: ch.11: 69598782-69599013 bp | - |

| | | | | | | | |
|---|---|---|---|---|---|---|---|
| | | | | | | exon 2: ch.11: 69598398-69598501 bp<br>exon 3: ch.11: 69594385-69594699 bp | |
| | | | | | putative | exon 1: ch.11: 69669707-69670046 bp<br>exon 2: ch.11: 69668986-69669089 bp<br>exon 3: ch.11: 69668271-69668447 bp | - |
| | | | | | putative | exon 1: ch.11: 69713615-69713834 bp<br>exon 2: ch.11: 69711221-69711324 bp<br>exon 3: ch.11: 69705307-69705702 bp | - |
| | | | | | putative | exon 1: ch.15: 30016512-30016797 bp<br>exon 2: ch.15: 30077466-30077569 bp<br>exon 3: ch.15: 30078625-30078819 bp | - |
| | | | | | putative | exon 1: ch.19: 558346-558559 bp<br>exon 2: ch.19: 561621-561724 bp<br>exon 3: ch.19: 561796-561990 bp | - |
| | | Western gorilla | GCF_000151905.2 | 2 | *FGF4B* | exon 1: ch.13: 3266402-3266678 bp<br>exon 2: ch.13: 3275519-3275622 bp<br>exon 3: ch.13: 3295732-3295977 bp | LR130278 |
| | | | | | *FGF4C* | exon 1: ch.X: 76998729-76999002 bp<br>exon 2: ch.X: 77005496-77005599 bp<br>exon 3: ch.X: 77007623-77007868 bp | LR130279 |
| | | | | | putative | exon 1: ch.3: 195708426-195708624 bp<br>exon 2: ch.3: 195658358-195658468 bp<br>exon 3: ch.3: 195624445-195624548 bp<br>exon 4: ch.3: 195457338-195457536 bp<br>exon 5: ch.3: 195425580-195425698 bp | - |
| | | | | | putative | exon 1: ch.4: 91226619-91226973 bp<br>exon 2: ch.4: 91234653-91234756 bp<br>exon 3: ch.4: 91246077-91246424 bp | - |
| | | | | | putative | exon 1: ch.4: 134793810-134793987 bp<br>exon 2: ch.4: 134843536-134843639 bp<br>exon 3: ch.4: 134859105-134859290 bp | - |
| | | | | | putative | exon 1: ch.5: 126202384-126202552 bp<br>exon 2: ch.5: 126189766-126189869 bp<br>exon 3: ch.5: 126184630-126184824 bp | - |
| | | | | | putative | exon 1: ch.8: 21203535-21203853 bp | - |

| | | | | | | | |
|---|---|---|---|---|---|---|---|
| | | | | | | exon 2: ch.8: 21204089-21204195 bp<br>exon 3: ch.8: 21205519-21205812 bp | |
| | | | | | putative | exon 1: ch.10: 116422481-116423081 bp<br>exon 2: ch.10: 116419209-116419315 bp<br>exon 3: ch.10: 116418076-116418366 bp | - |
| | | | | | putative | exon 1: ch.11: 68992780-68993011 bp<br>exon 2: ch.11: 68992341-68992444 bp<br>exon 3: ch.11: 68988333-68988647 bp | - |
| | | | | | putative | exon 1: ch.11: 69068112-69068451 bp<br>exon 2: ch.11: 69067397-69067500 bp<br>exon 3: ch.11: 69066682-69066858 bp | - |
| | | | | | putative | exon 1: ch.11: 69111100-69111319 bp<br>exon 2: ch.11: 69108767-69108870 bp<br>exon 3: ch.11: 69102831-69103226 bp | - |
| | | | | | putative | exon 1: ch.12: 4400961-4401555 bp<br>exon 2: ch.12: 4394210-4394313 bp<br>exon 3: ch.12: 4392389-4392829 bp | - |
| | | | | | putative | exon 1: ch.12: 4466749-4467094 bp<br>exon 2: ch.12: 4465657-4465760 bp<br>exon 3: ch.12: 4455722-4455898 bp | - |
| | | | | | putative | exon 1: ch.13: 84848943-84849135 bp<br>exon 2: ch.13: 84806330-84806440 bp<br>exon 3: ch.13: 84799871-84799974 bp<br>exon 4: ch.13: 84658507-84658705 bp<br>exon 5: ch.13: 84654740-84654876 bp | - |
| | | | | | putative | exon 1: ch.15: 27650210-27650495 bp<br>exon 2: ch.15: 27710334-27710437 bp<br>exon 3: ch.15: 27711491-27711685 bp | - |
| | | | | | putative | exon 1: ch.17: 7460314-7460506 bp<br>exon 2: ch.17: 7462152-7462262 bp<br>exon 3: ch.17: 7462456-7462559 bp<br>exon 4: ch.17: 7463281-7463479 bp<br>exon 5: ch.17: 7463763-7463839 bp | - |
| | | | | | putative | exon 1: ch.17: 50995000-50995321 bp<br>exon 2: ch.17: 51076166-51076269 bp | - |

| | | | | | | | |
|---|---|---|---|---|---|---|---|
| | | | | | | exon 3: ch.17: 51081839-51082036 bp | |
| | | | | | putative | exon 1: ch.19: 48013768-48014002 bp<br>exon 2: ch.19: 48014475-48014578 bp<br>exon 3: ch.19: 48016977-48017267 bp | - |
| | | | | | putative | exon 1: ch.X: 138769905-138770091 bp<br>exon 2: ch.X: 138767910-138768020 bp<br>exon 3: ch.X: 138762075-138762178 bp<br>exon 4: ch.X: 138694646-138694844 bp<br>exon 5: ch.X: 138692051-138692187 bp | - |
| | | Sumatran orangutan | GCF_000001545.4 | 9 | *FGF1B* | exon 1: ch.13: 104297153-104297345 bp<br>exon 2: ch.13: 104254332-104254442 bp<br>exon 3: ch.13: 104247570-104247673 bp<br>exon 4: ch.13: 104103049-104103247 bp<br>exon 5: ch.13: 104099288-104099424 bp | LR130280 |
| | | | | | *FGF1C* | exon 1: ch.X: 138321820-138322006 bp<br>exon 2: ch.X: 138319824-138319934 bp<br>exon 3: ch.X: 138314000-138314103 bp<br>exon 4: ch.X: 138243895-138244093 bp<br>exon 5: ch.X: 138240958-138241094 bp | LR130281 |
| | | | | | *FGF2A* | exon 1: ch.4: 127467064-127467241 bp<br>exon 2: ch.4: 127516902-127517005 bp<br>exon 3: ch.4: 127535471-127535656 bp | LR130282 |
| | | | | | *FGF2B* | exon 1: ch.5: 144166965-144167133 bp<br>exon 2: ch.5: 144153360-144153463 bp<br>exon 3: ch.5: 144148242-144148436 bp | LR130283 |
| | | | | | *FGF4C* | exon 1: ch.X: 74984133-74984406 bp<br>exon 2: ch.X: 74990651-74990754 bp<br>exon 3: ch.X: 74992792-74993037 bp | LR130284 |
| | | | | | *FGF5C* | exon 1: ch.11: 6211629-6211848 bp<br>exon 2: ch.11: 6214374-6214477 bp<br>exon 3: ch.11: 6220672-6221067 bp | LR130285 |
| | | | | | *FGF7B* | exon 1: ch.12: 4556471-4556816 bp<br>exon 2: ch.12: 4555393-4555496 bp<br>exon 3: ch.12: 4545529-4545705 bp | LR130286 |
| | | | | | *FGF8A* | exon 1: ch.11: 6332148-6332379 bp | LR130287 |

| | | | | | | | |
|---|---|---|---|---|---|---|---|
| | | | | | | exon 2: ch.11: 6332642-6332745 bp<br>exon 3: ch.11: 6336424-6336738 bp | |
| | | | | | *FGF8C* | exon 1: ch.19: 50298662-50298896 bp<br>exon 2: ch.19: 50299334-50299437 bp<br>exon 3: ch.19: 50300234-50300524 bp | LR130288 |
| | | | | | putative | exon 1: ch.4: 83700092-83700446 bp<br>exon 2: ch.4: 83708150-83708253 bp<br>exon 3: ch.4: 83719706-83720053 bp | - |
| | | | | | putative | exon 1: ch.5: 45582602-45582923 bp<br>exon 2: ch.5: 45502032-45502135 bp<br>exon 3: ch.5: 45493306-45493503 bp | - |
| | | | | | putative | exon 1: ch.5: 174018581-174018612 bp<br>exon 2: ch.5: 174018788-174018824 bp<br>exon 3: ch.5: 174034193-174034373 bp<br>exon 4: ch.5: 174050558-174050664 bp<br>exon 5: ch.5: 174058000-174058266 bp | - |
| | | | | | putative | exon 1: ch.8: 16341800-16342085 bp<br>exon 2: ch.8: 16335698-16335801 bp<br>exon 3: ch.8: 16332674-16332919 bp | - |
| | | | | | putative | exon 1: ch.8: 21612594-21612912 bp<br>exon 2: ch.8: 21613132-21613238 bp<br>exon 3: ch.8: 21614566-21614859 bp | - |
| | | | | | putative | exon 1: ch.11: 6257884-6258223 bp<br>exon 2: ch.11: 6258841-6258944 bp<br>exon 3: ch.11: 6259483-6259659 bp | - |
| | | | | | putative | exon 1: ch.13: 20812453-20812729 bp<br>exon 2: ch.13: 20821504-20821607 bp<br>exon 3: ch.13: 20841514-20841759 bp | - |
| | | | | | putative | exon 1: ch.15: 45957220-45957505 bp<br>exon 2: ch.15: 46016383-46016486 bp<br>exon 3: ch.15: 46017577-46017771 bp | - |
| | | Northern white-cheeked gibbon | GCF_000146795.2 | 15 | *FGF1A* | exon 1: ch.11: 117322696-117322894 bp<br>exon 2: ch.11: 117274788-117274898 bp<br>exon 3: ch.11: 117250102-117250205 bp<br>exon 4: ch.11: 117081672-117081870 bp | LR130289 |

| | | | | | | | |
|---|---|---|---|---|---|---|---|
| | | | | | | exon 5: ch.11: 117054706-117054824 bp | |
| | | | | | *FGF1D* | exon 1: ch.19: 74178281-74178473 bp<br>exon 2: ch.19: 74176457-74176567 bp<br>exon 3: ch.19: 74176159-74176262 bp<br>exon 4: ch.19: 74175242-74175440 bp<br>exon 5: ch.19: 74174888-74174958 bp | LR130290 |
| | | | | | *FGF2A* | exon 1: ch.7B: 33621224-33621401 bp<br>exon 2: ch.7B: 33570888-33570991 bp<br>exon 3: ch.7B: 33554965-33555150 bp | LR130291 |
| | | | | | *FGF2B* | exon 1: ch.2: 38638497-38638665 bp<br>exon 2: ch.2: 38651892-38651995 bp<br>exon 3: ch.2: 38657230-38657424 bp | LR130292 |
| | | | | | *FGF3A* | exon 1: ch.9: 65176369-65176720 bp<br>exon 2: ch.9: 65184383-65184486 bp<br>exon 3: ch.9: 65195761-65196108 bp | LR130293 |
| | | | | | *FGF4A* | exon 1: ch.4: 136310106-136310391 bp<br>exon 2: ch.4: 136316398-136316501 bp<br>exon 3: ch.4: 136318842-136319087 bp | LR130294 |
| | | | | | *FGF4B* | exon 1: ch.5: 63368596-63368872 bp<br>exon 2: ch.5: 63359648-63359751 bp<br>exon 3: ch.5: 63339473-63339718 bp | LR130295 |
| | | | | | *FGF4C* | exon 1: ch.X: 69095690-69095963 bp<br>exon 2: ch.X: 69102187-69102290 bp<br>exon 3: ch.X: 69104891-69105136 bp | LR130296 |
| | | | | | *FGF5A* | exon 1: ch.7B: 41003446-41003770 bp<br>exon 2: ch.7B: 40924284-40924387 bp<br>exon 3: ch.7B: 40918838-40919035 bp | LR130297 |
| | | | | | *FGF5B* | exon 1: ch.6: 51119603-51119888 bp<br>exon 2: ch.6: 51063816-51063919 bp<br>exon 3: ch.6: 51062536-51062730 bp | LR130298 |
| | | | | | *FGF6B* | exon 1: ch.8: 27566385-27566703 bp<br>exon 2: ch.8: 27566939-27567045 bp<br>exon 3: ch.8: 27568387-27568680 bp | LR130299 |
| | | | | | *FGF6C* | exon 1: ch.3: 32557719-32557932 bp<br>exon 2: ch.3: 32561087-32561193 bp | LR130300 |

| | | | | | | | |
|---|---|---|---|---|---|---|---|
| | | | | | | exon 3: ch.3: 32562032-32562322 bp | |
| | | | | | *FGF7B* | exon 1: ch.23: 30093289-30093634 bp<br>exon 2: ch.23: 30094606-30094709 bp<br>exon 3: ch.23: 30104325-30104501 bp | LR130301 |
| | | | | | *FGF8B* | exon 1: ch.23: 30164636-30165230 bp<br>exon 2: ch.23: 30171885-30171988 bp<br>exon 3: ch.23: 30173348-30173788 bp | LR130302 |
| | | | | | *FGF8C* | exon 1: ch.10: 69149455-69149689 bp<br>exon 2: ch.10: 69150127-69150230 bp<br>exon 3: ch.10: 69151075-69151365 bp | LR130303 |
| | | | | | putative | exon 1: ch.4: 89935604-89935835 bp<br>exon 2: ch.4: 89935219-89935322 bp<br>exon 3: ch.4: 89931202-89931516 bp | - |
| | | | | | putative | exon 1: ch.17: 99452954-99453167 bp<br>exon 2: ch.17: 99449980-99450083 bp<br>exon 3: ch.17: 99449714-99449908 bp | - |
| | | Rhesus monkey | GCF_000772875.2 | 15 | *FGF1B* | exon 1: ch.17: 83606157-83606349 bp<br>exon 2: ch.17: 83559151-83559261 bp<br>exon 3: ch.17: 83552851-83552954 bp<br>exon 4: ch.17: 83410449-83410647 bp<br>exon 5: ch.17: 83407081-83407217 bp | LR130304 |
| | | | | | *FGF1C* | exon 1: ch.X: 132586505-132586691 bp<br>exon 2: ch.X: 132584495-132584605 bp<br>exon 3: ch.X: 132578662-132578765 bp<br>exon 4: ch.X: 132513438-132513636 bp<br>exon 5: ch.X: 132510726-132510862 bp | LR130305 |
| | | | | | *FGF1D* | exon 1: ch.16: 7416633-7416825 bp<br>exon 2: ch.16: 7418474-7418584 bp<br>exon 3: ch.16: 7418779-7418882 bp<br>exon 4: ch.16: 7419627-7419825 bp<br>exon 5: ch.16: 7420109-7420179 bp | LR130306 |
| | | | | | *FGF2A* | exon 1: ch.5: 121818532-121818709 bp<br>exon 2: ch.5: 121868899-121869002 bp<br>exon 3: ch.5: 121884173-121884358 bp | LR130307 |
| | | | | | *FGF3A* | exon 1: ch.5: 54136076-54136430 bp | LR130308 |

| | | | | | | | |
|---|---|---|---|---|---|---|---|
| | | | | | | exon 2: ch.5: 54128341-54128444 bp<br>exon 3: ch.5: 54116658-54117005 bp | |
| | | | | | *FGF4A* | exon 1: ch.8: 16676331-16676616 bp<br>exon 2: ch.8: 16670213-16670316 bp<br>exon 3: ch.8: 16667570-16667815 bp | LR130309 |
| | | | | | *FGF5A* | exon 1: ch.6: 45344348-45344672 bp<br>exon 2: ch.6: 45266433-45266536 bp<br>exon 3: ch.6: 45261430-45261627 bp | LR130310 |
| | | | | | *FGF5B* | exon 1: ch.7: 25744853-25745138 bp<br>exon 2: ch.7: 25811336-25811439 bp<br>exon 3: ch.7: 25812528-25812722 bp | LR130311 |
| | | | | | *FGF5C* | exon 1: ch.14: 4804885-4805104 bp<br>exon 2: ch.14: 4807277-4807380 bp<br>exon 3: ch.14: 4813024-4813419 bp | LR130312 |
| | | | | | *FGF5D* | exon 1: ch.19: 410374-410587 bp<br>exon 2: ch.19: 412908-413011 bp<br>exon 3: ch.19: 413088-413282 bp | LR130313 |
| | | | | | *FGF6B* | exon 1: ch.8: 21941549-21942149 bp<br>exon 2: ch.8: 21942385-21942491 bp<br>exon 3: ch.8: 21943812-21944105 bp | LR130314 |
| | | | | | *FGF6C* | exon 1: ch.9: 97449077-97449290 bp<br>exon 2: ch.9: 97445827-97445933 bp<br>exon 3: ch.9: 97444697-97444987 bp | LR130315 |
| | | | | | *FGF7A* | exon 1: ch.14: 4849206-4849545 bp<br>exon 2: ch.14: 4850162-4850265 bp<br>exon 3: ch.14: 4850805-4850981 bp | LR130316 |
| | | | | | *FGF7B* | exon 1: ch.11: 4605514-4605859 bp<br>exon 2: ch.11: 4604437-4604540 bp<br>exon 3: ch.11: 4594357-4594533 bp | LR130317 |
| | | | | | *FGF8A* | exon 1: ch.14: 4919086-4919317 bp<br>exon 2: ch.14: 4919599-4919702 bp<br>exon 3: ch.14: 4923538-4923852 bp | LR130318 |
| | | | | | putative | exon 1: ch.2: 199137261-199137459 bp<br>exon 2: ch.2: 199088744-199088854 bp<br>exon 3: ch.2: 199064145-199064248 bp | - |

| | | | | | | | |
|---|---|---|---|---|---|---|---|
| | | | | | | exon 4: ch.2: 198901539-198901737 bp<br>exon 5: ch.2: 198884982-198885100 bp | |
| | | | | | putative | exon 1: ch.6: 169667529-169667560 bp<br>exon 2: ch.6: 169667736-169667772 bp<br>exon 3: ch.6: 169683642-169683822 bp<br>exon 4: ch.6: 169696776-169696882 bp<br>exon 5: ch.6: 169704713-169704979 bp | - |
| | | | | | putative | exon 1: ch.11: 4537341-4537755 bp<br>exon 2: ch.11: 4530407-4530510 bp<br>exon 3: ch.11: 4528271-4528711 bp | - |
| | | | | | putative | exon 1: ch.17: 1924221-1924497 bp<br>exon 2: ch.17: 1933329-1933432 bp<br>exon 3: ch.17: 1953249-1953494 bp | - |
| | | | | | putative | exon 1: ch.19: 44124793-44125027 bp<br>exon 2: ch.19: 44125932-44126035 bp<br>exon 3: ch.19: 44127032-44127322 bp | - |
| | | Hamadryas baboon | Pham_1.0 (Ensembl) | 5 | *FGF3A* | exon 1: Contig762019_Contig379044: 51492-51846 bp<br>exon 2: Contig762019_Contig379044: 43760-43863 bp<br>exon 3: Contig762019_Contig379044: 32106-32453 bp | LR130319 |
| | | | | | *FGF4A* | exon 1: Contig566918_Contig770812: 69355-69640 bp<br>exon 2: Contig566918_Contig770812: 75684-75787 bp<br>exon 3: Contig566918_Contig770812: 78189-78434 bp | LR130320 |
| | | | | | *FGF5D* | exon 1: Contig718534_Contig718165: 22524-22737 bp<br>exon 2: Contig718534_Contig718165: 20187-20290 bp<br>exon 3: Contig718534_Contig718165: 19922-20116 bp | LR130321 |
| | | | | | *FGF6C* | exon 1: Contig592989_Contig296769: 116324-116537 bp<br>exon 2: Contig592989_Contig296769: 119685-119791 bp<br>exon 3: Contig592989_Contig296769: 120630-120920 bp | LR130322 |
| | | | | | *FGF7B* | exon 1:<br>Contig776295_Contig673922_Contig402161_Contig519974:<br>68526-68871 bp<br>exon 2:<br>Contig776295_Contig673922_Contig402161_Contig519974:<br>70169-70272 bp<br>exon 3:<br>Contig776295_Contig673922_Contig402161_Contig519974: | LR130323 |

| | | | | | | | |
|---|---|---|---|---|---|---|---|
| | | | | | | 80144-80320 bp | |
| | | | | | putative | exon 1: Contig72255_Contig746947: 19747-20086 bp<br>exon 2: Contig72255_Contig746947: 19027-19130 bp<br>exon 3: Contig72255_Contig746947: 18311-18487 bp | - |
| | | | | | putative | exon 1: Contig72255_Contig746947: 64349-64568 bp<br>exon 2: Contig72255_Contig746947: 61435-61538 bp<br>exon 3: Contig72255_Contig746947: 53939-54334 bp | - |
| | | | | | putative | exon 1: Contig190260_Contig718866: 4820-5093 bp<br>exon 2: Contig190260_Contig718866: 11302-11405 bp<br>exon 3: Contig190260_Contig718866: 13807-14052 bp | - |
| | | | | | putative | exon 1: Contig415093_Contig565782: 46225-46456 bp<br>exon 2: Contig415093_Contig565782: 46738-46841 bp<br>exon 3: Contig415093_Contig565782: 50495-50809 bp | - |
| | | | | | putative | exon 1: Contig528609_Contig741373: 423872-424472 bp<br>exon 2: Contig528609_Contig741373: 423530-423636 bp<br>exon 3: Contig528609_Contig741373: 421931-422224 bp | - |
| | | | | | putative | exon 1: Contig650550_Contig491865: 197750-197781 bp<br>exon 2: Contig650550_Contig491865: 197538-197574 bp<br>exon 3: Contig650550_Contig491865: 181568-181748 bp<br>exon 4: Contig650550_Contig491865: 168766-168872 bp<br>exon 5: Contig650550_Contig491865: 160566-160832 bp | - |
| | | | | | putative | exon 1: Contig722043_Contig18964: 52600-52768 bp<br>exon 2: Contig722043_Contig18964: 65389-65492 bp<br>exon 3: Contig722043_Contig18964: 71274-71468 bp | - |
| | | | | | putative | exon 1: Contig776295_Contig673922_Contig402161_Contig519974: 140638-141052 bp<br>exon 2: Contig776295_Contig673922_Contig402161_Contig519974: 147168-147271 bp<br>exon 3: Contig776295_Contig673922_Contig402161_Contig519974: 149272-149712 bp | - |
| | | | | | putative | exon 1: Contig818725_Contig482630: 140080-140272 bp<br>exon 2: Contig818725_Contig482630: 141917-142027 bp<br>exon 3: Contig818725_Contig482630: 142222-142325 bp | - |

| | | | | | | | |
|---|---|---|---|---|---|---|---|
| | | | | | | exon 4: Contig818725_Contig482630: 143081-143279 bp<br>exon 5: Contig818725_Contig482630: 143563-143633 bp | |
| | | Common marmoset | GCF_000004665.1 | 10 | *FGF1A* | exon 1: ch.15: 14036186-14036384 bp<br>exon 2: ch.15: 13986860-13986970 bp<br>exon 3: ch.15: 13961739-13961842 bp<br>exon 4: ch.15: 13795486-13795684 bp<br>exon 5: ch.15: 13776719-13776837 bp | LR130324 |
| | | | | | *FGF1B* | exon 1: ch.1: 17138218-17138410 bp<br>exon 2: ch.1: 17098233-17098343 bp<br>exon 3: ch.1: 17091995-17092098 bp<br>exon 4: ch.1: 16935251-16935449 bp<br>exon 5: ch.1: 16930959-16931095 bp | LR130325 |
| | | | | | *FGF2B* | exon 1: ch.2: 39620756-39620924 bp<br>exon 2: ch.2: 39606267-39606370 bp<br>exon 3: ch.2: 39599428-39599622 bp | LR130326 |
| | | | | | *FGF3A* | exon 1: ch.3: 114241632-114241986 bp<br>exon 2: ch.3: 114234076-114234179 bp<br>exon 3: ch.3: 114221991-114222338 bp | LR130327 |
| | | | | | *FGF4A* | exon 1: ch.13: 21344880-21345165 bp<br>exon 2: ch.13: 21350875-21350978 bp<br>exon 3: ch.13: 21353413-21353658 bp | LR130328 |
| | | | | | *FGF4B* | exon 1: ch.5: 134860454-134860730 bp<br>exon 2: ch.5: 134869227-134869330 bp<br>exon 3: ch.5: 134892361-134892606 bp | LR130329 |
| | | | | | *FGF5A* | exon 1: ch.2: 158954441-158954768 bp<br>exon 2: ch.2: 159028472-159028575 bp<br>exon 3: ch.2: 159033580-159033777 bp | LR130330 |
| | | | | | *FGF5B* | exon 1: ch.10: 26558668-26558953 bp<br>exon 2: ch.10: 26616620-26616723 bp<br>exon 3: ch.10: 26617804-26617998 bp | LR130331 |
| | | | | | *FGF7A* | exon 1: ch.11: 125216311-125216650 bp<br>exon 2: ch.11: 125215646-125215749 bp<br>exon 3: ch.11: 125214935-125215111 bp | LR130332 |
| | | | | | *FGF7B* | exon 1: ch.9: 16095471-16095816 bp<br>exon 2: ch.9: 16094392-16094495 bp | LR130333 |

| | | | | | | | |
|---|---|---|---|---|---|---|---|
| | | | | | | exon 3: ch.9: 16084566-16084742 bp | |
| | | | | | putative | exon 1: ch.2: 68933705-68933736 bp<br>exon 2: ch.2: 68933493-68933529 bp<br>exon 3: ch.2: 68918728-68918908 bp<br>exon 4: ch.2: 68905398-68905504 bp<br>exon 5: ch.2: 68896748-68897014 bp | - |
| | | | | | putative | exon 1: ch.5: 74433324-74433516 bp<br>exon 2: ch.5: 74431224-74431334 bp<br>exon 3: ch.5: 74430926-74431029 bp<br>exon 4: ch.5: 74429996-74430194 bp<br>exon 5: ch.5: 74429333-74429403 bp | - |
| | | | | | putative | exon 1: ch.11: 125260425-125260644 bp<br>exon 2: ch.11: 125257556-125257659 bp<br>exon 3: ch.11: 125252068-125252463 bp | - |
| | | | | | putative | exon 1: ch.22: 363571-363784 bp<br>exon 2: ch.22: 366172-366275 bp<br>exon 3: ch.22: 366348-366542 bp | - |
| | | Philippine tarsier | GCF_000164805.1 | 1 | *FGF2B* | exon 1: NW_007087112.1: 56735-56903 bp<br>exon 2: NW_007087112.1: 43554-43657 bp<br>exon 3: NW_007087112.1: 37280-37474 bp | LR130334 |
| | | | | | putative | exon 1: NW_007070626.1: 179293-179638 bp<br>exon 2: NW_007070626.1: 177698-177801 bp<br>exon 3: NW_007070626.1: 166420-166596 bp | - |
| | | | | | putative | exon 1: NW_007074576.1: 19054-19288 bp<br>exon 2: NW_007074576.1: 19401-19504 bp<br>exon 3: NW_007074576.1: 20762-21052 bp | - |
| | | | | | putative | exon 1: NW_007150143.1: 291778-291964 bp<br>exon 2: NW_007150143.1: 289770-289880 bp<br>exon 3: NW_007150143.1: 283978-284081 bp<br>exon 4: NW_007150143.1: 213718-213916 bp<br>exon 5: NW_007150143.1: 212302-212438 bp | - |
| | | | | | putative | exon 1: NW_007249784.1: 38876-39068 bp<br>exon 2: NW_007249784.1: 40701-40811 bp<br>exon 3: NW_007249784.1: 41015-41118 bp<br>exon 4: NW_007249784.1: 41691-41889 bp<br>exon 5: NW_007249784.1: 42169-42239 bp | - |

| | | | | | | | |
|---|---|---|---|---|---|---|---|
| | | | | | putative | exon 1: NW_007252738.1: 435811-436132 bp<br>exon 2: NW_007252738.1: 364122-364225 bp<br>exon 3: NW_007252738.1: 358664-358861 bp | - |
| | | | | | putative | exon 1: NW_007256482.1: 98808-99093 bp<br>exon 2: NW_007256482.1: 34740-34843 bp<br>exon 3: NW_007256482.1: 33493-33687 bp | - |
| | | | | | putative | exon 1: NW_007258182.1: 595263-595620 bp<br>exon 2: NW_007258182.1: 595857-595963 bp<br>exon 3: NW_007258182.1: 596832-597125 bp | - |
| | | Gray mouse lemur | GCF_000165445.2 | 1 | *FGF8A* | exon 1: NC_033664.1: 14265326-14265800 bp<br>exon 2: NC_033664.1: 14264962-14265065 bp<br>exon 3: NC_033664.1: 14261594-14261908 bp | LR130335 |
| | | | | | putative | exon 1: NC_033660.1: 83921830-83922028 bp<br>exon 2: NC_033660.1: 83876820-83876930 bp<br>exon 3: NC_033660.1: 83850329-83850432 bp<br>exon 4: NC_033660.1: 83713666-83713864 bp<br>exon 5: NC_033660.1: 83700056-83700174 bp | - |
| | | | | | putative | exon 1: NC_033662.1: 82745160-82745445 bp<br>exon 2: NC_033662.1: 82682231-82682334 bp<br>exon 3: NC_033662.1: 82680949-82681143 bp | - |
| | | | | | putative | exon 1: NC_033664.1: 14320451-14320790 bp<br>exon 2: NC_033664.1: 14319838-14319941 bp<br>exon 3: NC_033664.1: 14319127-14319303 bp | - |
| | | | | | putative | exon 1: NC_033664.1: 14352454-14352673 bp<br>exon 2: NC_033664.1: 14350126-14350229 bp<br>exon 3: NC_033664.1: 14345169-14345549 bp | - |
| | | | | | putative | exon 1: NC_033666.1: 106428401-106428746 bp<br>exon 2: NC_033666.1: 106429618-106429721 bp<br>exon 3: NC_033666.1: 106437670-106437846 bp | - |
| | | | | | putative | exon 1: NC_033666.1: 106482930-106483140 bp<br>exon 2: NC_033666.1: 106488708-106488811 bp<br>exon 3: NC_033666.1: 106490095-106490532 bp | - |
| | | | | | putative | exon 1: NC_033671.1: 44189638-44189962 bp<br>exon 2: NC_033671.1: 44120370-44120473 bp<br>exon 3: NC_033671.1: 44114530-44114727 bp | - |

| | | | | | | | |
|---|---|---|---|---|---|---|---|
| | | | | | putative | exon 1: NC_033672.1: 12274662-12274854 bp<br>exon 2: NC_033672.1: 12322913-12323023 bp<br>exon 3: NC_033672.1: 12329043-12329146 bp<br>exon 4: NC_033672.1: 12463042-12463240 bp<br>exon 5: NC_033672.1: 12466252-12466388 bp | - |
| | | | | | putative | exon 1: NC_033672.1: 82003745-82004021 bp<br>exon 2: NC_033672.1: 81995443-81995546 bp<br>exon 3: NC_033672.1: 81976800-81977045 bp | - |
| | | | | | putative | exon 1: NC_033673.1: 73817480-73817657 bp<br>exon 2: NC_033673.1: 73773938-73774041 bp<br>exon 3: NC_033673.1: 73761653-73761838 bp | - |
| | | | | | putative | exon 1: NC_033674.1: 51430128-51430380 bp<br>exon 2: NC_033674.1: 51426740-51426846 bp<br>exon 3: NC_033674.1: 51425641-51425931 bp | - |
| | | | | | putative | exon 1: NC_033675.1: 55614068-55614260 bp<br>exon 2: NC_033675.1: 55612333-55612443 bp<br>exon 3: NC_033675.1: 55612038-55612141 bp<br>exon 4: NC_033675.1: 55611268-55611466 bp<br>exon 5: NC_033675.1: 55610918-55610988 bp | - |
| | | | | | putative | exon 1: NC_033678.1: 34710953-34711121 bp<br>exon 2: NC_033678.1: 34699384-34699487 bp<br>exon 3: NC_033678.1: 34693799-34693993 bp | - |
| | | | | | putative | exon 1: NC_033679.1: 21320855-21321095 bp<br>exon 2: NC_033679.1: 21320510-21320616 bp<br>exon 3: NC_033679.1: 21319226-21319519 bp | - |
| | | | | | putative | exon 1: NC_033679.1: 25178508-25178793 bp<br>exon 2: NC_033679.1: 25184353-25184456 bp<br>exon 3: NC_033679.1: 25186256-25186501 bp | - |
| | | | | | putative | exon 1: NC_033681.1: 18914677-18914917 bp<br>exon 2: NC_033681.1: 18915034-18915137 bp<br>exon 3: NC_033681.1: 18915722-18916012 bp | - |
| | | | | | putative | exon 1: NC_033682.1: 7567412-7567443 bp<br>exon 2: NC_033682.1: 7567616-7567652 bp<br>exon 3: NC_033682.1: 7580231-7580411 bp<br>exon 4: NC_033682.1: 7592070-7592176 bp<br>exon 5: NC_033682.1: 7599254-7599520 bp | - |

| | | | | | | | |
|---|---|---|---|---|---|---|---|
| | | | | | putative | exon 1: NC_033683.1: 9933099-9933318 bp<br>exon 2: NC_033683.1: 9935373-9935479 bp<br>exon 3: NC_033683.1: 9935556-9935750 bp | - |
| | | | | | putative | exon 1: NC_033688.1: 21711586-21711952 bp<br>exon 2: NC_033688.1: 21725594-21725697 bp<br>exon 3: NC_033688.1: 21749301-21749648 bp | - |
| | | | | | putative | exon 1: NC_033692.1: 7606699-7606972 bp<br>exon 2: NC_033692.1: 7614688-7614791 bp<br>exon 3: NC_033692.1: 7616149-7616394 bp | - |
| | | | | | putative | exon 1: NC_033692.1: 79440097-79440283 bp<br>exon 2: NC_033692.1: 79438077-79438187 bp<br>exon 3: NC_033692.1: 79432548-79432651 bp<br>exon 4: NC_033692.1: 79369049-79369247 bp<br>exon 5: NC_033692.1: 79367432-79367568 bp | - |
| | | Northern greater galago | GCF_000181295.1 | 10 | *FGF1D* | exon 1: NW_003852441.1: 11922515-11922707 bp<br>exon 2: NW_003852441.1: 11920775-11920885 bp<br>exon 3: NW_003852441.1: 11920481-11920584 bp<br>exon 4: NW_003852441.1: 11919678-11919876 bp<br>exon 5: NW_003852441.1: 11919324-11919394 bp | LR130336 |
| | | | | | *FGF2B* | exon 1: NW_003852398.1: 33343323-33343491 bp<br>exon 2: NW_003852398.1: 33355864-33355967 bp<br>exon 3: NW_003852398.1: 33360415-33360609 bp | LR130337 |
| | | | | | *FGF3A* | exon 1: NW_003852434.1: 9015851-9016208 bp<br>exon 2: NW_003852434.1: 9008347-9008450 bp<br>exon 3: NW_003852434.1: 8996792-8997139 bp | LR130338 |
| | | | | | *FGF4B* | exon 1: NW_003852501.1: 3785614-3785890 bp<br>exon 2: NW_003852501.1: 3794935-3795038 bp<br>exon 3: NW_003852501.1: 3816390-3816635 bp | LR130339 |
| | | | | | *FGF4C* | exon 1: NW_003852570.1: 2665154-2665427 bp<br>exon 2: NW_003852570.1: 2658684-2658787 bp<br>exon 3: NW_003852570.1: 2656879-2657124 bp | LR130340 |
| | | | | | *FGF5A* | exon 1: NW_003852458.1: 11010599-11010923 bp<br>exon 2: NW_003852458.1: 10938962-10939065 bp<br>exon 3: NW_003852458.1: 10933217-10933414 bp | LR130341 |
| | | | | | *FGF5D* | exon 1: NW_003852424.1: 13314062-13314266 bp | LR130342 |

| | | | | | | | |
|---|---|---|---|---|---|---|---|
| | | | | | | exon 2: NW_003852424.1: 13317948-13318054 bp<br>exon 3: NW_003852424.1: 13318130-13318324 bp | |
| | | | | | *FGF8A* | exon 1: NW_003852529.1: 1040053-1040293 bp<br>exon 2: NW_003852529.1: 1040544-1040647 bp<br>exon 3: NW_003852529.1: 1043302-1043616 bp | LR130343 |
| | | | | | *FGF8B* | exon 1: NW_003852499.1: 4274500-4274710 bp<br>exon 2: NW_003852499.1: 4279112-4279215 bp<br>exon 3: NW_003852499.1: 4280434-4280877 bp | LR130344 |
| | | | | | *FGF8C* | exon 1: NW_003852606.1: 1873516-1873756 bp<br>exon 2: NW_003852606.1: 1873297-1873400 bp<br>exon 3: NW_003852606.1: 1872423-1872713 bp | LR130345 |
| | | | | | putative | exon 1: NW_003852406.1: 2431550-2431835 bp<br>exon 2: NW_003852406.1: 2473961-2474064 bp<br>exon 3: NW_003852406.1: 2475152-2475346 bp | - |
| | | | | | putative | exon 1: NW_003852416.1: 18893276-18893528 bp<br>exon 2: NW_003852416.1: 18897479-18897585 bp<br>exon 3: NW_003852416.1: 18898394-18898684 bp | - |
| | | | | | putative | exon 1: NW_003852429.1: 10884838-10885030 bp<br>exon 2: NW_003852429.1: 10837084-10837194 bp<br>exon 3: NW_003852429.1: 10827126-10827229 bp<br>exon 4: NW_003852429.1: 10692225-10692423 bp<br>exon 5: NW_003852429.1: 10688840-10688976 bp | - |
| | | | | | putative | exon 1: NW_003852455.1: 4689431-4689629 bp<br>exon 2: NW_003852455.1: 4737250-4737360 bp<br>exon 3: NW_003852455.1: 4770868-4770971 bp<br>exon 4: NW_003852455.1: 4907995-4908193 bp<br>exon 5: NW_003852455.1: 4927385-4927503 bp | - |
| | | | | | putative | exon 1: NW_003852469.1: 7020691-7020931 bp<br>exon 2: NW_003852469.1: 7020352-7020458 bp<br>exon 3: NW_003852469.1: 7019072-7019365 bp | - |
| | | | | | putative | exon 1: NW_003852499.1: 4216158-4216503 bp<br>exon 2: NW_003852499.1: 4217401-4217504 bp<br>exon 3: NW_003852499.1: 4226438-4226614 bp | - |
| | | | | | putative | exon 1: NW_003852512.1: 1862458-1862644 bp<br>exon 2: NW_003852512.1: 1860397-1860507 bp | - |

| | | | | | | | |
|---|---|---|---|---|---|---|---|
| | | | | | | exon 3: NW_003852512.1: 1854191-1854294 bp<br>exon 4: NW_003852512.1: 1782790-1782988 bp<br>exon 5: NW_003852512.1: 1781147-1781283 bp | |
| | | | | | putative | exon 1: NW_003852516.1: 1003248-1003533 bp<br>exon 2: NW_003852516.1: 1009711-1009814 bp<br>exon 3: NW_003852516.1: 1012611-1012856 bp | - |
| | | | | | putative | exon 1: NW_003852529.1: 954310-954529 bp<br>exon 2: NW_003852529.1: 956697-956800 bp<br>exon 3: NW_003852529.1: 962117-962494 bp | - |
| | | | | | putative | exon 1: NW_003852529.1: 989743-990082 bp<br>exon 2: NW_003852529.1: 990649-990752 bp<br>exon 3: NW_003852529.1: 991296-991472 bp | - |
| | Scadentia | Northern treeshrew | tupBel1 (Ensembl) | 2 | *FGF4C* | exon 1: scaffold_148398: 58001-58274 bp<br>exon 2: scaffold_148398: 67056-67159 bp<br>exon 3: scaffold_148398: 68755-69000 bp | LR130346 |
| | | | | | *FGF5A* | exon 1: GeneScaffold_403: 217684-218005 bp<br>exon 2: GeneScaffold_403: 144915-145018 bp<br>exon 3: GeneScaffold_403: 138238-138435 bp | LR130347 |
| | | | | | putative | exon 1: GeneScaffold_1410: 265641-265917 bp<br>exon 2: GeneScaffold_1410: 273889-273992 bp<br>exon 3: GeneScaffold_1410: 300463-300708 bp | - |
| | | | | | putative | exon 1: GeneScaffold_1465: 263508-263676 bp<br>exon 2: GeneScaffold_1465: 237299-237402 bp<br>exon 3: GeneScaffold_1465: 225536-225730 bp | - |
| | Rodentia | Mouse | GCF_000001635.24 | 22 | *Fgf1a* | exon 1: ch.16: 28444993-28445191 bp<br>exon 2: ch.16: 28398278-28398388 bp<br>exon 3: ch.16: 28372913-28373016 bp<br>exon 4: ch.16: 28189434-28189632 bp<br>exon 5: ch.16: 28162417-28162535 bp | LR130348 |
| | | | | | *Fgf1b* | exon 1: ch.14: 124192329-124192521 bp<br>exon 2: ch.14: 124136182-124136292 bp<br>exon 3: ch.14: 124132463-124132566 bp<br>exon 4: ch.14: 123983769-123983967 bp<br>exon 5: ch.14: 123980346-123980482 bp | LR130349 |
| | | | | | *Fgf1c* | exon 1: ch.X: 59133877-59134063 bp | LR130350 |

| | | | | | | | |
|---|---|---|---|---|---|---|---|
| | | | | | | exon 2: ch.X: 59131840-59131950 bp<br>exon 3: ch.X: 59125894-59125997 bp<br>exon 4: ch.X: 59067401-59067599 bp<br>exon 5: ch.X: 59063533-59063669 bp | |
| | | | | | *Fgf1d* | exon 1: ch.11: 69801433-69801625 bp<br>exon 2: ch.11: 69799666-69799776 bp<br>exon 3: ch.11: 69799352-69799455 bp<br>exon 4: ch.11: 69798564-69798762 bp<br>exon 5: ch.11: 69798202-69798272 bp | LR130351 |
| | | | | | *Fgf2a* | exon 1: ch.3: 37348850-37349024 bp<br>exon 2: ch.3: 37396185-37396288 bp<br>exon 3: ch.3: 37404612-37404797 bp | LR130352 |
| | | | | | *Fgf2b* | exon 1: ch.18: 38858546-38858714 bp<br>exon 2: ch.18: 38847047-38847150 bp<br>exon 3: ch.18: 38841927-38842121 bp | LR130353 |
| | | | | | *Fgf3a* | exon 1: ch.5: 98254412-98254760 bp<br>exon 2: ch.5: 98261943-98262046 bp<br>exon 3: ch.5: 98275211-98275552 bp | LR130354 |
| | | | | | *Fgf4a* | exon 1: ch.8: 40286422-40286707 bp<br>exon 2: ch.8: 40281111-40281214 bp<br>exon 3: ch.8: 40279760-40280005 bp | LR130355 |
| | | | | | *Fgf4b* | exon 1: ch.14: 58073056-58073332 bp<br>exon 2: ch.14: 58083201-58083304 bp<br>exon 3: ch.14: 58109333-58109578 bp | LR130356 |
| | | | | | *Fgf4c* | exon 1: ch.X: 105764541-105764814 bp<br>exon 2: ch.X: 105772518-105772621 bp<br>exon 3: ch.X: 105773935-105774180 bp | LR130357 |
| | | | | | *Fgf5a* | exon 1: ch.13: 118715231-118715711 bp<br>exon 2: ch.13: 118781506-118781609 bp<br>exon 3: ch.13: 118789119-118789316 bp | LR130358 |
| | | | | | *Fgf5b* | exon 1: ch.2: 126035715-126036000 bp<br>exon 2: ch.2: 126088235-126088338 bp<br>exon 3: ch.2: 126089367-126089561 bp | LR130359 |
| | | | | | *Fgf5c* | exon 1: ch.7: 144838708-144838927 bp<br>exon 2: ch.7: 144840671-144840774 bp | LR130360 |

| | | | | | | | |
|---|---|---|---|---|---|---|---|
| | | | | | | exon 3: ch.7: 144842575-144842988 bp | |
| | | | | | *Fgf5d* | exon 1: ch.10: 79755119-79755305 bp<br>exon 2: ch.10: 79756580-79756683 bp<br>exon 3: ch.10: 79756764-79756961 bp | LR130361 |
| | | | | | *Fgf6a* | exon 1: ch.11: 33147242-33147273 bp<br>exon 2: ch.11: 33147045-33147081 bp<br>exon 3: ch.11: 33134203-33134383 bp<br>exon 4: ch.11: 33124613-33124719 bp<br>exon 5: ch.11: 33117765-33118031 bp | LR130362 |
| | | | | | *Fgf6b* | exon 1: ch.14: 70638802-70639231 bp<br>exon 2: ch.14: 70638467-70638573 bp<br>exon 3: ch.14: 70636730-70637023 bp | LR130363 |
| | | | | | *Fgf6c* | exon 1: ch.19: 45741499-45741712 bp<br>exon 2: ch.19: 45738118-45738224 bp<br>exon 3: ch.19: 45736990-45737280 bp | LR130364 |
| | | | | | *Fgf7a* | exon 1: ch.7: 144861505-144861832 bp<br>exon 2: ch.7: 144862232-144862335 bp<br>exon 3: ch.7: 144862765-144862941 bp | LR130365 |
| | | | | | *Fgf7b* | exon 1: ch.6: 127015586-127015931 bp<br>exon 2: ch.6: 127016994-127017097 bp<br>exon 3: ch.6: 127023981-127024157 bp | LR130366 |
| | | | | | *Fgf8a* | exon 1: ch.7: 144896679-144896931 bp<br>exon 2: ch.7: 144897116-144897219 bp<br>exon 3: ch.7: 144899649-144899948 bp | LR130367 |
| | | | | | *Fgf8b* | exon 1: ch.6: 127073030-127073240 bp<br>exon 2: ch.6: 127078140-127078243 bp<br>exon 3: ch.6: 127080038-127080478 bp | LR130368 |
| | | | | | *Fgf8c* | exon 1: ch.7: 45615069-45615306 bp<br>exon 2: ch.7: 45614866-45614969 bp<br>exon 3: ch.7: 45614020-45614310 bp | LR130369 |
| | | Brown rat | GCF_000001895.5 | 19 | *Fgf1a* | exon 1: ch.11: 75905443-75905641 bp<br>exon 2: ch.11: 75955020-75955130 bp<br>exon 3: ch.11: 75980363-75980466 bp<br>exon 4: ch.11: 76151403-76151601 bp<br>exon 5: ch.11: 76168673-76168791 bp | LR130370 |

| | | | | | | | |
|---|---|---|---|---|---|---|---|
| | | | | | *Fgf1c* | exon 1: ch.X: 142131359-142131545 bp<br>exon 2: ch.X: 142129421-142129531 bp<br>exon 3: ch.X: 142123677-142123780 bp<br>exon 4: ch.X: 142059218-142059416 bp<br>exon 5: ch.X: 142054963-142055099 bp | LR130371 |
| | | | | | *Fgf1d* | exon 1: ch.10: 56408737-56408929 bp<br>exon 2: ch.10: 56406826-56406936 bp<br>exon 3: ch.10: 56406512-56406615 bp<br>exon 4: ch.10: 56405744-56405942 bp<br>exon 5: ch.10: 56405415-56405485 bp | LR130372 |
| | | | | | *Fgf2a* | exon 1: ch.2: 124081604-124081778 bp<br>exon 2: ch.2: 124123341-124123444 bp<br>exon 3: ch.2: 124133929-124134114 bp | LR130373 |
| | | | | | *Fgf2b* | exon 1: ch.18: 32340673-32340841 bp<br>exon 2: ch.18: 32351614-32351717 bp<br>exon 3: ch.18: 32356430-32356624 bp | LR130374 |
| | | | | | *Fgf3a* | exon 1: ch.14: 12995236-12995584 bp<br>exon 2: ch.14: 12988082-12988185 bp<br>exon 3: ch.14: 12974921-12975268 bp | LR130375 |
| | | | | | *Fgf4a* | exon 1: ch.16: 55152748-55153033 bp<br>exon 2: ch.16: 55158044-55158147 bp<br>exon 3: ch.16: 55159104-55159352 bp | LR130376 |
| | | | | | *Fgf4b* | exon 1: ch.15: 38341809-38342085 bp<br>exon 2: ch.15: 38352245-38352348 bp<br>exon 3: ch.15: 38386418-38386663 bp | LR130377 |
| | | | | | *Fgf5a* | exon 1: ch.2: 51673480-51673825 bp<br>exon 2: ch.2: 51740159-51740262 bp<br>exon 3: ch.2: 51747336-51747533 bp | LR130378 |
| | | | | | *Fgf5b* | exon 1: ch.3: 118317869-118318154 bp<br>exon 2: ch.3: 118365585-118365688 bp<br>exon 3: ch.3: 118366678-118366872 bp | LR130379 |
| | | | | | *Fgf5c* | exon 1: ch.1: 218003018-218003237 bp<br>exon 2: ch.1: 218004975-218005078 bp<br>exon 3: ch.1: 218006529-218006942 bp | LR130380 |
| | | | | | *Fgf5d* | exon 1: ch.7: 12831489-12831675 bp | LR130381 |

| | | | | | | | |
|---|---|---|---|---|---|---|---|
| | | | | | | exon 2: ch.7: 12830063-12830166 bp<br>exon 3: ch.7: 12829785-12829982 bp | |
| | | | | | *Fgf6b* | exon 1: ch.15: 52328791-52329217 bp<br>exon 2: ch.15: 52328436-52328542 bp<br>exon 3: ch.15: 52326778-52327071 bp | LR130382 |
| | | | | | *Fgf6c* | exon 1: ch.1: 265497637-265497850 bp<br>exon 2: ch.1: 265494313-265494419 bp<br>exon 3: ch.1: 265493124-265493414 bp | LR130383 |
| | | | | | *Fgf7a* | exon 1: ch.1: 218025810-218026137 bp<br>exon 2: ch.1: 218026535-218026638 bp<br>exon 3: ch.1: 218027044-218027220 bp | LR130384 |
| | | | | | *Fgf7b* | exon 1: ch.4: 159563798-159564143 bp<br>exon 2: ch.4: 159565186-159565289 bp<br>exon 3: ch.4: 159572157-159572333 bp | LR130385 |
| | | | | | *Fgf8a* | exon 1: ch.1: 218058405-218058657 bp<br>exon 2: ch.1: 218058840-218058943 bp<br>exon 3: ch.1: 218061394-218061693 bp | LR130386 |
| | | | | | *Fgf8b* | exon 1: ch.4: 159622185-159622614 bp<br>exon 2: ch.4: 159627825-159627928 bp<br>exon 3: ch.4: 159629642-159630082 bp | LR130387 |
| | | | | | *Fgf8c* | exon 1: ch.1: 101596585-101596822 bp<br>exon 2: ch.1: 101596384-101596487 bp<br>exon 3: ch.1: 101595579-101595863 bp | LR130388 |
| | | | | | putative | exon 1: ch.X: 76786728-76787001 bp<br>exon 2: ch.X: 76794601-76794704 bp<br>exon 3: ch.X: 76796066-76796311 bp | - |
| | | Ord's kangaroo rat | GCF_000151885.1 | 1 | *FGF3A* | exon 1: NW_012267265.1: 8792943-8793309 bp<br>exon 2: NW_012267265.1: 8800989-8801092 bp<br>exon 3: NW_012267265.1: 8812121-8812468 bp | LR130389 |
| | | | | | putative | exon 1: NW_012267217.1: 29500375-29500651 bp<br>exon 2: NW_012267217.1: 29512982-29513085 bp<br>exon 3: NW_012267217.1: 29548774-29549019 bp | - |
| | | | | | putative | exon 1: NW_012267220.1: 20190943-20191228 bp<br>exon 2: NW_012267220.1: 20195972-20196075 bp<br>exon 3: NW_012267220.1: 20197117-20197362 bp | - |

| | | | | | | | |
|---|---|---|---|---|---|---|---|
| | | | | | putative | exon 1: NW_012267227.1: 12105116-12105401 bp<br>exon 2: NW_012267227.1: 12054621-12054724 bp<br>exon 3: NW_012267227.1: 12053461-12053655 bp | - |
| | | | | | putative | exon 1: NW_012267235.1: 12031074-12031266 bp<br>exon 2: NW_012267235.1: 12029246-12029356 bp<br>exon 3: NW_012267235.1: 12028971-12029074 bp<br>exon 4: NW_012267235.1: 12028236-12028434 bp<br>exon 5: NW_012267235.1: 12027906-12027976 bp | - |
| | | | | | putative | exon 1: NW_012267252.1: 5281333-5281651 bp<br>exon 2: NW_012267252.1: 5278250-5278356 bp<br>exon 3: NW_012267252.1: 5277088-5277378 bp | - |
| | | | | | putative | exon 1: NW_012267257.1: 6429899-6430268 bp<br>exon 2: NW_012267257.1: 6497336-6497439 bp<br>exon 3: NW_012267257.1: 6499387-6499584 bp | - |
| | | | | | putative | exon 1: NW_012267261.1: 4267630-4267828 bp<br>exon 2: NW_012267261.1: 4188986-4189096 bp<br>exon 3: NW_012267261.1: 4160603-4160706 bp<br>exon 4: NW_012267261.1: 4000504-4000702 bp<br>exon 5: NW_012267261.1: 3989462-3989580 bp | - |
| | | | | | putative | exon 1: NW_012267266.1: 969356-969524 bp<br>exon 2: NW_012267266.1: 983408-983511 bp<br>exon 3: NW_012267266.1: 988578-988772 bp | - |
| | | | | | putative | exon 1: NW_012267276.1: 8602636-8602810 bp<br>exon 2: NW_012267276.1: 8575468-8575571 bp<br>exon 3: NW_012267276.1: 8563926-8564111 bp | - |
| | | | | | putative | exon 1: NW_012267280.1: 3696877-3697090 bp<br>exon 2: NW_012267280.1: 3699340-3699443 bp<br>exon 3: NW_012267280.1: 3699515-3699709 bp | - |
| | | | | | putative | exon 1: NW_012267281.1: 4436677-4436869 bp<br>exon 2: NW_012267281.1: 4476229-4476339 bp<br>exon 3: NW_012267281.1: 4482591-4482694 bp<br>exon 4: NW_012267281.1: 4594691-4594889 bp<br>exon 5: NW_012267281.1: 4595470-4595606 bp | - |
| | | | | | putative | exon 1: NW_012267285.1: 1864867-1865173 bp<br>exon 2: NW_012267285.1: 1866801-1866904 bp<br>exon 3: NW_012267285.1: 1872129-1872305 bp | - |

| | | | | | | | |
|---|---|---|---|---|---|---|---|
| | | | | | putative | exon 1: NW_012267305.1: 9949667-9949853 bp<br>exon 2: NW_012267305.1: 9951839-9951949 bp<br>exon 3: NW_012267305.1: 9958255-9958358 bp<br>exon 4: NW_012267305.1: 10025265-10025463 bp<br>exon 5: NW_012267305.1: 10027778-10027914 bp | - |
| | | | | | putative | exon 1: NW_012267321.1: 573870-574329 bp<br>exon 2: NW_012267321.1: 574456-574559 bp<br>exon 3: NW_012267321.1: 575538-575813 bp | - |
| | | | | | putative | exon 1: NW_012267332.1: 2131009-2131282 bp<br>exon 2: NW_012267332.1: 2122645-2122748 bp<br>exon 3: NW_012267332.1: 2120061-2120306 bp | - |
| | | | | | putative | exon 1: NW_012267405.1: 2838463-2838796 bp<br>exon 2: NW_012267405.1: 2838105-2838211 bp<br>exon 3: NW_012267405.1: 2836505-2836798 bp | - |
| | | Domesticated guinea pig | GCF_000151735.1 | 16 | *FGF1A* | exon 1: NT_176412.1: 56503041-56503239 bp<br>exon 2: NT_176412.1: 56459792-56459902 bp<br>exon 3: NT_176412.1: 56437786-56437889 bp<br>exon 4: NT_176412.1: 56290097-56290295 bp<br>exon 5: NT_176412.1: 56275753-56275871 bp | LR130390 |
| | | | | | *FGF1C* | exon 1: NT_176340.1: 3465192-3465378 bp<br>exon 2: NT_176340.1: 3467308-3467418 bp<br>exon 3: NT_176340.1: 3472291-3472394 bp<br>exon 4: NT_176340.1: 3533391-3533589 bp<br>exon 5: NT_176340.1: 3535069-3535205 bp | LR130391 |
| | | | | | *FGF1D* | exon 1: NT_176358.1: 9208494-9208686 bp<br>exon 2: NT_176358.1: 9206781-9206891 bp<br>exon 3: NT_176358.1: 9206486-9206589 bp<br>exon 4: NT_176358.1: 9205762-9205960 bp<br>exon 5: NT_176358.1: 9205411-9205481 bp | LR130392 |
| | | | | | *FGF2B* | exon 1: NT_176413.1: 4907024-4907192 bp<br>exon 2: NT_176413.1: 4894506-4894609 bp<br>exon 3: NT_176413.1: 4891073-4891264 bp | LR130393 |
| | | | | | *FGF3A* | exon 1: NT_176414.1: 4323641-4323998 bp<br>exon 2: NT_176414.1: 4330943-4331046 bp<br>exon 3: NT_176414.1: 4340570-4340917 bp | LR130394 |
| | | | | | *FGF4C* | exon 1: NT_176393.1: 19167518-19167791 bp | LR130395 |

| | | | | | | | |
|---|---|---|---|---|---|---|---|
| | | | | | | exon 2: NT_176393.1: 19160795-19160898 bp<br>exon 3: NT_176393.1: 19159192-19159437 bp | |
| | | | | | *FGF5A* | exon 1: NT_176390.1: 12870769-12871117 bp<br>exon 2: NT_176390.1: 12940580-12940683 bp<br>exon 3: NT_176390.1: 12944979-12945176 bp | LR130396 |
| | | | | | *FGF5B* | exon 1: NT_176396.1: 27735089-27735374 bp<br>exon 2: NT_176396.1: 27782053-27782156 bp<br>exon 3: NT_176396.1: 27783300-27783494 bp | LR130397 |
| | | | | | *FGF5C* | exon 1: NT_176377.1: 9595288-9595507 bp<br>exon 2: NT_176377.1: 9593177-9593280 bp<br>exon 3: NT_176377.1: 9588761-9589144 bp | LR130398 |
| | | | | | *FGF6A* | exon 1: NT_176402.1: 17407473-17407504 bp<br>exon 2: NT_176402.1: 17407663-17407699 bp<br>exon 3: NT_176402.1: 17420992-17421172 bp<br>exon 4: NT_176402.1: 17433545-17433651 bp<br>exon 5: NT_176402.1: 17440497-17440763 bp | LR130399 |
| | | | | | *FGF6B* | exon 1: NT_176418.1: 58874666-58875101 bp<br>exon 2: NT_176418.1: 58875301-58875407 bp<br>exon 3: NT_176418.1: 58876254-58876547 bp | LR130400 |
| | | | | | *FGF7A* | exon 1: NT_176377.1: 9568077-9568416 bp<br>exon 2: NT_176377.1: 9567415-9567518 bp<br>exon 3: NT_176377.1: 9566741-9566917 bp | LR130401 |
| | | | | | *FGF7B* | exon 1: NT_176312.1: 2536578-2536923 bp<br>exon 2: NT_176312.1: 2535552-2535655 bp<br>exon 3: NT_176312.1: 2525992-2526168 bp | LR130402 |
| | | | | | *FGF8A* | exon 1: NT_176377.1: 9516816-9517071 bp<br>exon 2: NT_176377.1: 9516472-9516575 bp<br>exon 3: NT_176377.1: 9513735-9514040 bp | LR130403 |
| | | | | | *FGF8B* | exon 1: NT_176312.1: 2471910-2472102 bp<br>exon 2: NT_176312.1: 2465590-2465693 bp<br>exon 3: NT_176312.1: 2463881-2464315 bp | LR130404 |
| | | | | | *FGF8C* | exon 1: NT_176349.1: 8485071-8485323 bp<br>exon 2: NT_176349.1: 8484888-8484991 bp<br>exon 3: NT_176349.1: 8484111-8484401 bp | LR130405 |
| | | | | | putative | exon 1: NT_176063.1: 83074-83287 bp | - |

| | | | | | | | |
|---|---|---|---|---|---|---|---|
| | | | | | | exon 2: NT_176063.1: 84921-85024 bp<br>exon 3: NT_176063.1: 85113-85307 bp | |
| | | Thirteen-lined ground squirrel | GCF_000236235.1 | 0 | putative | exon 1: NW_004936471.1: 11637004-11637289 bp<br>exon 2: NW_004936471.1: 11690441-11690544 bp<br>exon 3: NW_004936471.1: 11691608-11691802 bp | - |
| | | | | | putative | exon 1: NW_004936472.1: 9019736-9019928 bp<br>exon 2: NW_004936472.1: 9066351-9066461 bp<br>exon 3: NW_004936472.1: 9073052-9073155 bp<br>exon 4: NW_004936472.1: 9223669-9223867 bp<br>exon 5: NW_004936472.1: 9227464-9227600 bp | - |
| | | | | | putative | exon 1: NW_004936480.1: 18114859-18115189 bp<br>exon 2: NW_004936480.1: 18186854-18186957 bp<br>exon 3: NW_004936480.1: 18193015-18193212 bp | - |
| | | | | | putative | exon 1: NW_004936504.1: 11664823-11664991 bp<br>exon 2: NW_004936504.1: 11676654-11676757 bp<br>exon 3: NW_004936504.1: 11681896-11682090 bp | - |
| | | | | | putative | exon 1: NW_004936513.1: 8839628-8839814 bp<br>exon 2: NW_004936513.1: 8841739-8841849 bp<br>exon 3: NW_004936513.1: 8847877-8847980 bp<br>exon 4: NW_004936513.1: 8907870-8908068 bp<br>exon 5: NW_004936513.1: 8909607-8909743 bp | - |
| | | | | | putative | exon 1: NW_004936555.1: 5875748-5876111 bp<br>exon 2: NW_004936555.1: 5876324-5876430 bp<br>exon 3: NW_004936555.1: 5877263-5877556 bp | - |
| | | | | | putative | exon 1: NW_004936573.1: 6062399-6062684 bp<br>exon 2: NW_004936573.1: 6056769-6056872 bp<br>exon 3: NW_004936573.1: 6055343-6055588 bp | - |
| | | | | | putative | exon 1: NW_004936588.1: 227685-227898 bp<br>exon 2: NW_004936588.1: 229541-229644 bp<br>exon 3: NW_004936588.1: 229716-229910 bp | - |
| | | | | | putative | exon 1: NW_004936595.1: 722562-722754 bp<br>exon 2: NW_004936595.1: 724380-724490 bp<br>exon 3: NW_004936595.1: 724686-724789 bp<br>exon 4: NW_004936595.1: 725333-725531 bp<br>exon 5: NW_004936595.1: 725990-726060 bp | - |

| | | | | | | | |
|---|---|---|---|---|---|---|---|
| | | | | | putative | exon 1: NW_004936599.1: 868634-868820 bp<br>exon 2: NW_004936599.1: 870635-870738 bp<br>exon 3: NW_004936599.1: 875093-875464 bp | - |
| | | | | | putative | exon 1: NW_004936600.1: 4005972-4006185 bp<br>exon 2: NW_004936600.1: 4009243-4009349 bp<br>exon 3: NW_004936600.1: 4010183-4010473 bp | - |
| | | | | | putative | exon 1: NW_004936606.1: 4561751-4561961 bp<br>exon 2: NW_004936606.1: 4557047-4557150 bp<br>exon 3: NW_004936606.1: 4555034-4555477 bp | - |
| | | | | | putative | exon 1: NW_004936609.1: 3706457-3706488 bp<br>exon 2: NW_004936609.1: 3706272-3706308 bp<br>exon 3: NW_004936609.1: 3694444-3694624 bp<br>exon 4: NW_004936609.1: 3684140-3684246 bp<br>exon 5: NW_004936609.1: 3678002-3678268 bp | - |
| | | | | | putative | exon 1: NW_004936664.1: 2826823-2827057 bp<br>exon 2: NW_004936664.1: 2827145-2827248 bp<br>exon 3: NW_004936664.1: 2827932-2828219 bp | - |
| | | | | | putative | exon 1: NW_004936683.1: 2454375-2454648 bp<br>exon 2: NW_004936683.1: 2461286-2461389 bp<br>exon 3: NW_004936683.1: 2462706-2462951 bp | - |
| | | | | | putative | exon 1: NW_004936711.1: 2149661-2149859 bp<br>exon 2: NW_004936711.1: 2194736-2194846 bp<br>exon 3: NW_004936711.1: 2217785-2217888 bp<br>exon 4: NW_004936711.1: 2362769-2362967 bp<br>exon 5: NW_004936711.1: 2375778-2375896 bp | - |
| | | | | | putative | exon 1: NW_004936720.1: 315530-315806 bp<br>exon 2: NW_004936720.1: 306485-306588 bp<br>exon 3: NW_004936720.1: 282881-283126 bp | - |
| | | | | | putative | exon 1: NW_004936749.1: 1761197-1761560 bp<br>exon 2: NW_004936749.1: 1753483-1753586 bp<br>exon 3: NW_004936749.1: 1742618-1742965 bp | - |
| | Lagomorpha | European rabbit | GCF_000003625.3 | 12 | *FGF1A* | exon 1: ch.14: 88268065-88268263 bp<br>exon 2: ch.14: 88214200-88214310 bp<br>exon 3: ch.14: 88179018-88179121 bp<br>exon 4: ch.14: 87994842-87995040 bp<br>exon 5: ch.14: 87979823-87979941 bp | LR130406 |

| | | | | | | | |
|---|---|---|---|---|---|---|---|
| | | | | | *FGF1B* | exon 1: ch.8: 103488767-103488959 bp<br>exon 2: ch.8: 103439891-103440001 bp<br>exon 3: ch.8: 103432570-103432673 bp<br>exon 4: ch.8: 103277534-103277732 bp<br>exon 5: ch.8: 103273772-103273908 bp | LR130407 |
| | | | | | *FGF1D* | exon 1: ch.19: 11731076-11731268 bp<br>exon 2: ch.19: 11729316-11729426 bp<br>exon 3: ch.19: 11729028-11729131 bp<br>exon 4: ch.19: 11727528-11727726 bp<br>exon 5: ch.19: 11727225-11727295 bp | LR130408 |
| | | | | | *FGF2A* | exon 1: ch.15: 98654512-98654689 bp<br>exon 2: ch.15: 98708361-98708464 bp<br>exon 3: ch.15: 98738738-98738923 bp | LR130409 |
| | | | | | *FGF2B* | exon 1: ch.3: 24596819-24596987 bp<br>exon 2: ch.3: 24584059-24584162 bp<br>exon 3: ch.3: 24578886-24579080 bp | LR130410 |
| | | | | | *FGF3A* | exon 1: ch.15: 70122204-70122555 bp<br>exon 2: ch.15: 70114248-70114351 bp<br>exon 3: ch.15: 70103383-70103730 bp | LR130411 |
| | | | | | *FGF4B* | exon 1: ch.8: 43084215-43084491 bp<br>exon 2: ch.8: 43074674-43074777 bp<br>exon 3: ch.8: 43047133-43047378 bp | LR130412 |
| | | | | | *FGF5A* | exon 1: ch.11: 65079268-65079628 bp<br>exon 2: ch.11: 64999752-64999855 bp<br>exon 3: ch.11: 64992608-64992805 bp | LR130413 |
| | | | | | *FGF5B* | exon 1: ch.17: 23043622-23043907 bp<br>exon 2: ch.17: 22974928-22975031 bp<br>exon 3: ch.17: 22973697-22973891 bp | LR130414 |
| | | | | | *FGF7B* | exon 1: ch.8: 30536866-30537211 bp<br>exon 2: ch.8: 30535951-30536054 bp<br>exon 3: ch.8: 30525951-30526127 bp | LR130415 |
| | | | | | *FGF8B* | exon 1: ch.8: 30476716-30477109 bp<br>exon 2: ch.8: 30473066-30473169 bp<br>exon 3: ch.8: 30470870-30471310 bp | LR130416 |
| | | | | | *FGF8C* | exon 1: NW_003159655.1: 213632-213866 bp | LR130417 |

| | | | | | | | |
|---|---|---|---|---|---|---|---|
| | | | | | | exon 2: NW_003159655.1: 213189-213292 bp<br>exon 3: NW_003159655.1: 212251-212541 bp | |
| | | | | | putative | exon 1: ch.3: 52642280-52642311 bp<br>exon 2: ch.3: 52642448-52642484 bp<br>exon 3: ch.3: 52655133-52655313 bp<br>exon 4: ch.3: 52667541-52667647 bp<br>exon 5: ch.3: 52675367-52675633 bp | - |
| | | | | | putative | exon 1: NW_003159326.1: 1900246-1900432 bp<br>exon 2: NW_003159326.1: 1898173-1898283 bp<br>exon 3: NW_003159326.1: 1891529-1891632 bp<br>exon 4: NW_003159326.1: 1792628-1792826 bp<br>exon 5: NW_003159326.1: 1790988-1791124 bp | - |
| | | | | | putative | exon 1: NW_003161787.1: 347-587 bp<br>exon 2: NW_003161787.1: 811-914 bp<br>exon 3: NW_003161787.1: 2719-3030 bp | - |
| | | American pika | GCF_000292845.1 | 1 | *FGF8C* | exon 1: NW_004535522.1: 4121872-4122094 bp<br>exon 2: NW_004535522.1: 4121694-4121797 bp<br>exon 3: NW_004535522.1: 4120544-4120828 bp | LR130418 |
| | | | | | putative | exon 1: NW_004535437.1: 8173065-8173257 bp<br>exon 2: NW_004535437.1: 8209959-8210069 bp<br>exon 3: NW_004535437.1: 8218362-8218465 bp<br>exon 4: NW_004535437.1: 8335648-8335846 bp<br>exon 5: NW_004535437.1: 8338635-8338771 bp | - |
| | | | | | putative | exon 1: NW_004535437.1: 71562565-71562841 bp<br>exon 2: NW_004535437.1: 71554404-71554507 bp<br>exon 3: NW_004535437.1: 71524124-71524369 bp | - |
| | | | | | putative | exon 1: NW_004535438.1: 8154752-8154950 bp<br>exon 2: NW_004535438.1: 8109855-8109965 bp<br>exon 3: NW_004535438.1: 8080942-8081045 bp<br>exon 4: NW_004535438.1: 7925692-7925890 bp<br>exon 5: NW_004535438.1: 7911515-7911633 bp | - |
| | | | | | putative | exon 1: NW_004535438.1: 48181214-48181499 bp<br>exon 2: NW_004535438.1: 48130501-48130604 bp<br>exon 3: NW_004535438.1: 48128950-48129144 bp | - |
| | | | | | putative | exon 1: NW_004535442.1: 23569734-23570079 bp<br>exon 2: NW_004535442.1: 23573054-23573160 bp | - |

| | | | | | | | |
|---|---|---|---|---|---|---|---|
| | | | | | | exon 3: NW_004535442.1: 23574007-23574297 bp | |
| | | | | | putative | exon 1: NW_004535452.1: 20296921-20297269 bp<br>exon 2: NW_004535452.1: 20222399-20222502 bp<br>exon 3: NW_004535452.1: 20216319-20216516 bp | - |
| | | | | | putative | exon 1: NW_004535460.1: 22948027-22948195 bp<br>exon 2: NW_004535460.1: 22936408-22936511 bp<br>exon 3: NW_004535460.1: 22931334-22931528 bp | - |
| | | | | | putative | exon 1: NW_004535463.1: 5576981-5577012 bp<br>exon 2: NW_004535463.1: 5576788-5576824 bp<br>exon 3: NW_004535463.1: 5562643-5562823 bp<br>exon 4: NW_004535463.1: 5551851-5551957 bp<br>exon 5: NW_004535463.1: 5545257-5545523 bp | - |
| | | | | | putative | exon 1: NW_004535464.1: 19202056-19202242 bp<br>exon 2: NW_004535464.1: 19200044-19200154 bp<br>exon 3: NW_004535464.1: 19194612-19194715 bp<br>exon 4: NW_004535464.1: 19117167-19117365 bp<br>exon 5: NW_004535464.1: 19115521-19115657 bp | - |
| | | | | | putative | exon 1: NW_004535478.1: 16653695-16654049 bp<br>exon 2: NW_004535478.1: 16646169-16646272 bp<br>exon 3: NW_004535478.1: 16634874-16635218 bp | - |
| | | | | | putative | exon 1: NW_004535483.1: 6597370-6597805 bp<br>exon 2: NW_004535483.1: 6596991-6597097 bp<br>exon 3: NW_004535483.1: 6596244-6596537 bp | - |
| | | | | | putative | exon 1: NW_004535491.1: 5419056-5419329 bp<br>exon 2: NW_004535491.1: 5425083-5425186 bp<br>exon 3: NW_004535491.1: 5426553-5426798 bp | - |
| | | | | | putative | exon 1: NW_004535505.1: 9226602-9226779 bp<br>exon 2: NW_004535505.1: 9267072-9267175 bp<br>exon 3: NW_004535505.1: 9281742-9281927 bp | - |
| | | | | | putative | exon 1: NW_004535507.1: 6554608-6554800 bp<br>exon 2: NW_004535507.1: 6552836-6552946 bp<br>exon 3: NW_004535507.1: 6552563-6552666 bp<br>exon 4: NW_004535507.1: 6551167-6551365 bp<br>exon 5: NW_004535507.1: 6550850-6550920 bp | - |
| | | | | | putative | exon 1: NW_004535513.1: 5955578-5955791 bp | - |

| | | | | | | | |
|---|---|---|---|---|---|---|---|
| | | | | | | exon 2: NW_004535513.1: 5954518-5954621 bp<br>exon 3: NW_004535513.1: 5953807-5954001 bp | |
| | | | | | putative | exon 1: NW_004535518.1: 1539223-1539433 bp<br>exon 2: NW_004535518.1: 1534343-1534446 bp<br>exon 3: NW_004535518.1: 1532423-1532863 bp | - |
| | | | | | putative | exon 1: NW_004535518.1: 1572171-1572516 bp<br>exon 2: NW_004535518.1: 1571193-1571296 bp<br>exon 3: NW_004535518.1: 1565272-1565448 bp | - |
| | | | | | putative | exon 1: NW_004535537.1: 2724594-2724879 bp<br>exon 2: NW_004535537.1: 2730177-2730280 bp<br>exon 3: NW_004535537.1: 2732348-2732593 bp | - |
| | | | | | putative | exon 1: NW_004535547.1: 1714802-1715489 bp<br>exon 2: NW_004535547.1: 1714493-1714596 bp<br>exon 3: NW_004535547.1: 1712560-1712868 bp | - |
| | | | | | putative | exon 1: NW_004535547.1: 1764632-1764851 bp<br>exon 2: NW_004535547.1: 1763539-1763642 bp<br>exon 3: NW_004535547.1: 1760487-1760885 bp | - |
| Laurasiatheria | Cetacea | Bottlenose dolphin | GCF_000151865.1 | 3 | *FGF2B* | exon 1: NW_004198086.1: 49714-49882 bp<br>exon 2: NW_004198086.1: 63383-63486 bp<br>exon 3: NW_004198086.1: 68486-68680 bp | LR130419 |
| | | | | | *FGF4B* | exon 1: NW_004201738.1: 141160-141436 bp<br>exon 2: NW_004201738.1: 132590-132693 bp<br>exon 3: NW_004201738.1: 113774-114019 bp | LR130420 |
| | | | | | *FGF7B* | exon 1: NW_004210751.1: 26071-26416 bp<br>exon 2: NW_004210751.1: 25021-25124 bp<br>exon 3: NW_004210751.1: 15679-15855 bp | LR130421 |
| | | | | | putative | exon 1: NW_004197588.1: 353874-353905 bp<br>exon 2: NW_004197588.1: 354081-354117 bp<br>exon 3: NW_004197588.1: 368455-368635 bp<br>exon 4: NW_004197588.1: 380863-380969 bp<br>exon 5: NW_004197588.1: 388414-388680 bp | - |
| | | | | | putative | exon 1: NW_004198685.1: 45888-46119 bp<br>exon 2: NW_004198685.1: 46242-46345 bp<br>exon 3: NW_004198685.1: 47896-48186 bp | - |
| | | | | | putative | exon 1: NW_004202709.1: 76283-76457 bp | - |

| | | | | | | | |
|---|---|---|---|---|---|---|---|
| | | | | | | exon 2: NW_004202709.1: 35793-35896 bp<br>exon 3: NW_004202709.1: 23838-24023 bp | |
| | | | | | putative | exon 1: NW_004204372.1: 46030-46315 bp<br>exon 2: NW_004204372.1: 51745-51848 bp<br>exon 3: NW_004204372.1: 53935-54180 bp | - |
| | | | | | putative | exon 1: NW_004208957.1: 18162-18483 bp<br>exon 2: NW_004208957.1: 17813-17919 bp<br>exon 3: NW_004208957.1: 16506-16799 bp | - |
| | | | | | putative | exon 1: NW_004209066.1: 85276-85561 bp<br>exon 2: NW_004209066.1: 22950-23053 bp<br>exon 3: NW_004209066.1: 21599-21793 bp | - |
| | Artiodactyla | Domestic cattle | GCA_000003205.6 | 14 | *FGF1C* | exon 1: ch.X: 22235736-22235922 bp<br>exon 2: ch.X: 22233742-22233852 bp<br>exon 3: ch.X: 22228266-22228369 bp<br>exon 4: ch.X: 22163671-22163869 bp<br>exon 5: ch.X: 22162227-22162363 bp | LR130422 |
| | | | | | *FGF1D* | exon 1: ch.19: 27893666-27893858 bp<br>exon 2: ch.19: 27895518-27895628 bp<br>exon 3: ch.19: 27895826-27895929 bp<br>exon 4: ch.19: 27896555-27896753 bp<br>exon 5: ch.19: 27897027-27897097 bp | LR130423 |
| | | | | | *FGF2B* | exon 1: ch.7: 55738349-55738517 bp<br>exon 2: ch.7: 55724868-55724971 bp<br>exon 3: ch.7: 55717854-55718048 bp | LR130424 |
| | | | | | *FGF3A* | exon 1: ch.6: 97123204-97123564 bp<br>exon 2: ch.6: 97133045-97133148 bp<br>exon 3: ch.6: 97145615-97145962 bp | LR130425 |
| | | | | | *FGF4A* | exon 1: ch.27: 19400763-19401048 bp<br>exon 2: ch.27: 19406810-19406913 bp<br>exon 3: ch.27: 19409345-19409590 bp | LR130426 |
| | | | | | *FGF4C* | exon 1: ch.X: 80900886-80901159 bp<br>exon 2: ch.X: 80894831-80894934 bp<br>exon 3: ch.X: 80893227-80893472 bp | LR130427 |
| | | | | | *FGF5B* | exon 1: ch.10: 61276790-61277075 bp<br>exon 2: ch.10: 61216611-61216714 bp | LR130428 |

| | | | | | | | |
|---|---|---|---|---|---|---|---|
| | | | | | | exon 3: ch.10: 61215170-61215364 bp | |
| | | | | | *FGF5C* | exon 1: ch.29: 48031134-48031353 bp<br>exon 2: ch.29: 48028805-48028908 bp<br>exon 3: ch.29: 48024336-48024722 bp | LR130429 |
| | | | | | *FGF5D* | exon 1: ch.7: 45066366-45066771 bp<br>exon 2: ch.7: 45068810-45068913 bp<br>exon 3: ch.7: 45069011-45069205 bp | LR130430 |
| | | | | | *FGF6A* | exon 1: ch.20: 3148952-3148983 bp<br>exon 2: ch.20: 3149155-3149191 bp<br>exon 3: ch.20: 3164003-3164183 bp<br>exon 4: ch.20: 3203211-3203317 bp<br>exon 5: ch.20: 3210190-3210456 bp | LR130431 |
| | | | | | *FGF6C* | exon 1: ch.26: 22500559-22500871 bp<br>exon 2: ch.26: 22497259-22497365 bp<br>exon 3: ch.26: 22496142-22496432 bp | LR130432 |
| | | | | | *FGF7B* | exon 1: ch.5: 106460105-106460450 bp<br>exon 2: ch.5: 106461391-106461494 bp<br>exon 3: ch.5: 106471942-106472118 bp | LR130433 |
| | | | | | *FGF8A* | exon 1: ch.29: 47925711-47925951 bp<br>exon 2: ch.29: 47925205-47925308 bp<br>exon 3: ch.29: 47922143-47922454 bp | LR130434 |
| | | | | | *FGF8C* | exon 1: ch.18: 56070172-56070406 bp<br>exon 2: ch.18: 56070537-56070640 bp<br>exon 3: ch.18: 56071340-56071630 bp | LR130435 |
| | | | | | putative | exon 1: ch.1: 75891828-75892026 bp<br>exon 2: ch.1: 75938975-75939085 bp<br>exon 3: ch.1: 75975294-75975397 bp<br>exon 4: ch.1: 76153374-76153572 bp<br>exon 5: ch.1: 76176355-76176473 bp | - |
| | | | | | putative | exon 1: ch.5: 106510375-106510570 bp<br>exon 2: ch.5: 106516823-106516926 bp<br>exon 3: ch.5: 106518516-106518953 bp | - |
| | | | | | putative | exon 1: ch.17: 35369773-35369950 bp<br>exon 2: ch.17: 35330443-35330546 bp<br>exon 3: ch.17: 35316556-35316741 bp | - |

| | | | | | | | |
|---|---|---|---|---|---|---|---|
| | | Wild boar | GCF_000003025.6 | 0 | putative | exon 1: ch.1: 122277199-122277484 bp<br>exon 2: ch.1: 122218041-122218144 bp<br>exon 3: ch.1: 122216828-122217022 bp | - |
| | | | | | putative | exon 1: ch.2: 3527113-3527452 bp<br>exon 2: ch.2: 3528065-3528168 bp<br>exon 3: ch.2: 3528697-3528873 bp | - |
| | | | | | putative | exon 1: ch.2: 3583027-3583267 bp<br>exon 2: ch.2: 3583672-3583775 bp<br>exon 3: ch.2: 3586437-3586751 bp | - |
| | | | | | putative | exon 1: ch.2: 77687022-77687235 bp<br>exon 2: ch.2: 77684812-77684915 bp<br>exon 3: ch.2: 77684539-77684733 bp | - |
| | | | | | putative | exon 1: ch.2: 144154500-144154668 bp<br>exon 2: ch.2: 144141795-144141898 bp<br>exon 3: ch.2: 144136900-144137094 bp | - |
| | | | | | putative | exon 1: ch.5: 65976083-65976428 bp<br>exon 2: ch.5: 65977400-65977503 bp<br>exon 3: ch.5: 65985400-65985576 bp | - |
| | | | | | putative | exon 1: ch.6: 54082783-54083014 bp<br>exon 2: ch.6: 54083426-54083529 bp<br>exon 3: ch.6: 54084213-54084503 bp | - |
| | | | | | putative | exon 1: ch.8: 101343256-101343433 bp<br>exon 2: ch.8: 101298118-101298221 bp<br>exon 3: ch.8: 101284364-101284549 bp | - |
| | | | | | putative | exon 1: ch.8: 137553899-137554259 bp<br>exon 2: ch.8: 137546115-137546218 bp<br>exon 3: ch.8: 137533261-137533611 bp | - |
| | | | | | putative | exon 1: ch.11: 1554796-1555072 bp<br>exon 2: ch.11: 1564292-1564395 bp<br>exon 3: ch.11: 1581649-1581894 bp | - |
| | | | | | putative | exon 1: ch.11: 70421972-70422164 bp<br>exon 2: ch.11: 70378854-70378964 bp<br>exon 3: ch.11: 70372321-70372424 bp<br>exon 4: ch.11: 70257566-70257764 bp<br>exon 5: ch.11: 70253354-70253490 bp | - |

| | | | | | | | |
|---|---|---|---|---|---|---|---|
| | | | | | putative | exon 1: ch.12: 52743455-52743647 bp<br>exon 2: ch.12: 52745326-52745436 bp<br>exon 3: ch.12: 52745656-52745759 bp<br>exon 4: ch.12: 52746380-52746578 bp<br>exon 5: ch.12: 52746873-52746943 bp | - |
| | | | | | putative | exon 1: ch.13: 129502298-129502496 bp<br>exon 2: ch.13: 129460057-129460167 bp<br>exon 3: ch.13: 129427710-129427813 bp<br>exon 4: ch.13: 129262046-129262244 bp<br>exon 5: ch.13: 129242021-129242139 bp | - |
| | | | | | putative | exon 1: ch.14: 6312907-6313288 bp<br>exon 2: ch.14: 6313537-6313643 bp<br>exon 3: ch.14: 6314683-6314976 bp | - |
| | | | | | putative | exon 1: ch.14: 112812884-112813136 bp<br>exon 2: ch.14: 112809571-112809677 bp<br>exon 3: ch.14: 112808396-112808686 bp | - |
| | | | | | putative | exon 1: ch.16: 28776564-28776897 bp<br>exon 2: ch.16: 28703342-28703445 bp<br>exon 3: ch.16: 28698206-28698403 bp | - |
| | | | | | putative | exon 1: ch.16: 52759657-52759688 bp<br>exon 2: ch.16: 52759452-52759488 bp<br>exon 3: ch.16: 52744485-52744665 bp<br>exon 4: ch.16: 52733037-52733143 bp<br>exon 5: ch.16: 52725712-52725978 bp | - |
| | | | | | putative | exon 1: ch.X: 61552659-61552932 bp<br>exon 2: ch.X: 61559336-61559439 bp<br>exon 3: ch.X: 61560810-61561055 bp | - |
| | | | | | putative | exon 1: ch.X: 113530760-113530946 bp<br>exon 2: ch.X: 113528710-113528820 bp<br>exon 3: ch.X: 113523080-113523183 bp<br>exon 4: ch.X: 113462869-113463067 bp<br>exon 5: ch.X: 113461470-113461606 bp | - |
| | | Vicugna | GCF_000164845.1 | 2 | *FGF4A* | exon 1: NW_005882710.1: 13810235-13810520 bp<br>exon 2: NW_005882710.1: 13815903-13816006 bp<br>exon 3: NW_005882710.1: 13817818-13818063 bp | LR130436 |
| | | | | | *FGF5B* | exon 1: NW_005882786.1: 6853278-6853563 bp | LR130437 |

| | | | | | | | |
|---|---|---|---|---|---|---|---|
| | | | | | | exon 2: NW_005882786.1: 6799842-6799945 bp<br>exon 3: NW_005882786.1: 6798633-6798827 bp | |
| | | | | | putative | exon 1: NW_005882727.1: 4790379-4790577 bp<br>exon 2: NW_005882727.1: 4833639-4833749 bp<br>exon 3: NW_005882727.1: 4860590-4860693 bp<br>exon 4: NW_005882727.1: 5002491-5002689 bp<br>exon 5: NW_005882727.1: 5020229-5020347 bp | - |
| | | | | | putative | exon 1: NW_005882739.1: 9111871-9112111 bp<br>exon 2: NW_005882739.1: 9111514-9111620 bp<br>exon 3: NW_005882739.1: 9110171-9110464 bp | - |
| | | | | | putative | exon 1: NW_005882746.1: 7093621-7093789 bp<br>exon 2: NW_005882746.1: 7105911-7106014 bp<br>exon 3: NW_005882746.1: 7110425-7110619 bp | - |
| | | | | | putative | exon 1: NW_005882775.1: 5650510-5650846 bp<br>exon 2: NW_005882775.1: 5582998-5583101 bp<br>exon 3: NW_005882775.1: 5579137-5579334 bp | - |
| | | | | | putative | exon 1: NW_005882786.1: 979720-979912 bp<br>exon 2: NW_005882786.1: 1020831-1020941 bp<br>exon 3: NW_005882786.1: 1026906-1027009 bp<br>exon 4: NW_005882786.1: 1128512-1128710 bp<br>exon 5: NW_005882786.1: 1130988-1131124 bp | - |
| | | | | | putative | exon 1: NW_005882787.1: 3352223-3352457 bp<br>exon 2: NW_005882787.1: 3352007-3352110 bp<br>exon 3: NW_005882787.1: 3351191-3351481 bp | - |
| | | | | | putative | exon 1: NW_005882817.1: 2578645-2578885 bp<br>exon 2: NW_005882817.1: 2578168-2578271 bp<br>exon 3: NW_005882817.1: 2575159-2575473 bp | - |
| | | | | | putative | exon 1: NW_005882819.1: 3476080-3476425 bp<br>exon 2: NW_005882819.1: 3475042-3475145 bp<br>exon 3: NW_005882819.1: 3467446-3467622 bp | - |
| | | | | | putative | exon 1: NW_005882946.1: 1406509-1406785 bp<br>exon 2: NW_005882946.1: 1399011-1399114 bp<br>exon 3: NW_005882946.1: 1382612-1382857 bp | - |
| | | | | | putative | exon 1: NW_005882992.1: 89949-90135 bp<br>exon 2: NW_005882992.1: 87880-87990 bp | - |

| | | | | | | | |
|---|---|---|---|---|---|---|---|
| | | | | | | exon 3: NW_005882992.1: 82260-82363 bp<br>exon 4: NW_005882992.1: 24746-24944 bp<br>exon 5: NW_005882992.1: 23183-23319 bp | |
| | | | | | putative | exon 1: NW_005883089.1: 409918-410110 bp<br>exon 2: NW_005883089.1: 408159-408269 bp<br>exon 3: NW_005883089.1: 407857-407960 bp<br>exon 4: NW_005883089.1: 407036-407234 bp<br>exon 5: NW_005883089.1: 406692-406762 bp | - |
| | Perissodactyla | Horse | GCF_002863925.1 | 10 | *FGF1A* | exon 1: ch.19: 32033807-32034005 bp<br>exon 2: ch.19: 31988639-31988749 bp<br>exon 3: ch.19: 31960962-31961065 bp<br>exon 4: ch.19: 31815880-31816078 bp<br>exon 5: ch.19: 31799716-31799834 bp | LR130438 |
| | | | | | *FGF1B* | exon 1: ch.17: 70502390-70502582 bp<br>exon 2: ch.17: 70456736-70456846 bp<br>exon 3: ch.17: 70450761-70450864 bp<br>exon 4: ch.17: 70338574-70338772 bp<br>exon 5: ch.17: 70335155-70335291 bp | LR130439 |
| | | | | | *FGF1D* | exon 1: ch.11: 50760889-50761081 bp<br>exon 2: ch.11: 50762724-50762834 bp<br>exon 3: ch.11: 50763028-50763131 bp<br>exon 4: ch.11: 50763692-50763887 bp<br>exon 5: ch.11: 50764161-50764231 bp | LR130440 |
| | | | | | *FGF2B* | exon 1: ch.14: 33844354-33844522 bp<br>exon 2: ch.14: 33856537-33856640 bp<br>exon 3: ch.14: 33860864-33861058 bp | LR130441 |
| | | | | | *FGF3A* | exon 1: ch.3: 57926312-57926669 bp<br>exon 2: ch.3: 57918779-57918882 bp<br>exon 3: ch.3: 57906462-57906809 bp | LR130442 |
| | | | | | *FGF4B* | exon 1: ch.17: 2438777-2439053 bp<br>exon 2: ch.17: 2447706-2447809 bp<br>exon 3: ch.17: 2467978-2468223 bp | LR130443 |
| | | | | | *FGF5B* | exon 1: ch.1: 141595029-141595314 bp<br>exon 2: ch.1: 141539814-141539917 bp<br>exon 3: ch.1: 141538555-141538749 bp | LR130444 |
| | | | | | *FGF7B* | exon 1: ch.6: 33224009-33224354 bp | LR130445 |

| | | | | | | | |
|---|---|---|---|---|---|---|---|
| | | | | | | exon 2: ch.6: 33222731-33222834 bp<br>exon 3: ch.6: 33213320-33213496 bp | |
| | | | | | *FGF8B* | exon 1: ch.6: 33165364-33165559 bp<br>exon 2: ch.6: 33159710-33159813 bp<br>exon 3: ch.6: 33157934-33158374 bp | LR130446 |
| | | | | | *FGF8C* | exon 1: ch.10: 19031853-19032087 bp<br>exon 2: ch.10: 19032602-19032705 bp<br>exon 3: ch.10: 19033308-19033598 bp | LR130447 |
| | | | | | putative | exon 1: ch.1: 28439237-28439489 bp<br>exon 2: ch.1: 28442620-28442726 bp<br>exon 3: ch.1: 28443587-28443877 bp | - |
| | | | | | putative | exon 1: ch.2: 51201072-51201393 bp<br>exon 2: ch.2: 51201628-51201734 bp<br>exon 3: ch.2: 51202844-51203137 bp | - |
| | | | | | putative | exon 1: ch.2: 105690175-105690352 bp<br>exon 2: ch.2: 105647544-105647647 bp<br>exon 3: ch.2: 105637423-105637608 bp | - |
| | | | | | putative | exon 1: ch.7: 239766-239979 bp<br>exon 2: ch.7: 241516-241619 bp<br>exon 3: ch.7: 241710-241904 bp | - |
| | | | | | putative | exon 1: ch.12: 32478864-32479104 bp<br>exon 2: ch.12: 32478518-32478621 bp<br>exon 3: ch.12: 32475361-32475675 bp | - |
| | | | | | putative | exon 1: ch.12: 32530585-32530924 bp<br>exon 2: ch.12: 32529887-32529990 bp<br>exon 3: ch.12: 32529226-32529402 bp | - |
| | | | | | putative | exon 1: ch.12: 32565340-32565559 bp<br>exon 2: ch.12: 32562896-32562999 bp<br>exon 3: ch.12: 32556679-32557050 bp | - |
| | | | | | putative | exon 1: ch.14: 9070492-9070523 bp<br>exon 2: ch.14: 9070284-9070320 bp<br>exon 3: ch.14: 9056757-9056937 bp<br>exon 4: ch.14: 9045058-9045164 bp<br>exon 5: ch.14: 9038077-9038343 bp | - |
| | | | | | putative | exon 1: ch.21: 23148984-23149332 bp | - |

| | | | | | | | |
|---|---|---|---|---|---|---|---|
| | | | | | | exon 2: ch.21: 23221199-23221302 bp<br>exon 3: ch.21: 23223736-23223933 bp | |
| | | | | | putative | exon 1: ch.27: 20158808-20159093 bp<br>exon 2: ch.27: 20153313-20153416 bp<br>exon 3: ch.27: 20150976-20151221 bp | - |
| | | | | | putative | exon 1: ch.X: 60531414-60531687 bp<br>exon 2: ch.X: 60537643-60537746 bp<br>exon 3: ch.X: 60539116-60539361 bp | - |
| | | | | | putative | exon 1: ch.X: 114391409-114391595 bp<br>exon 2: ch.X: 114389389-114389499 bp<br>exon 3: ch.X: 114383427-114383530 bp<br>exon 4: ch.X: 114327027-114327225 bp<br>exon 5: ch.X: 114325590-114325726 bp | - |
| | Carnivora | Domestic dog | GCF_000002285.3 | 13 | *FGF1A* | exon 1: ch.34: 24065277-24065475 bp<br>exon 2: ch.34: 24024024-24024134 bp<br>exon 3: ch.34: 23996954-23997057 bp<br>exon 4: ch.34: 23839330-23839528 bp<br>exon 5: ch.34: 23819774-23819892 bp | LR130448 |
| | | | | | *FGF1D* | exon 1: ch.5: 32369831-32370023 bp<br>exon 2: ch.5: 32371670-32371780 bp<br>exon 3: ch.5: 32371973-32372076 bp<br>exon 4: ch.5: 32372343-32372541 bp<br>exon 5: ch.5: 32372950-32373020 bp | LR130449 |
| | | | | | *FGF2B* | exon 1: ch.2: 37488863-37489031 bp<br>exon 2: ch.2: 37475389-37475492 bp<br>exon 3: ch.2: 37470985-37471179 bp | LR130450 |
| | | | | | *FGF3A* | exon 1: ch.32: 4509084-4509444 bp<br>exon 2: ch.32: 4517268-4517371 bp<br>exon 3: ch.32: 4528527-4528874 bp | LR130451 |
| | | | | | *FGF4B* | exon 1: ch.25: 16662755-16663031 bp<br>exon 2: ch.25: 16653786-16653889 bp<br>exon 3: ch.25: 16633382-16633627 bp | LR130452 |
| | | | | | *FGF4C* | exon 1: ch.X: 59739398-59739671 bp<br>exon 2: ch.X: 59745755-59745858 bp<br>exon 3: ch.X: 59747210-59747455 bp | LR130453 |

| | | | | | | | |
|---|---|---|---|---|---|---|---|
| | | | | | *FGF5B* | exon 1: ch.30: 15556199-15556484 bp<br>exon 2: ch.30: 15604881-15604984 bp<br>exon 3: ch.30: 15606274-15606468 bp | LR130454 |
| | | | | | *FGF6A* | exon 1: ch.4: 40748303-40748334 bp<br>exon 2: ch.4: 40748096-40748132 bp<br>exon 3: ch.4: 40733885-40734065 bp<br>exon 4: ch.4: 40722268-40722374 bp<br>exon 5: ch.4: 40715755-40716021 bp | LR130455 |
| | | | | | *FGF6B* | exon 1: ch.25: 35196438-35196768 bp<br>exon 2: ch.25: 35196090-35196196 bp<br>exon 3: ch.25: 35194748-35195041 bp | LR130456 |
| | | | | | *FGF7A* | exon 1: ch.18: 48413480-48413819 bp<br>exon 2: ch.18: 48414392-48414495 bp<br>exon 3: ch.18: 48415030-48415206 bp | LR130457 |
| | | | | | *FGF7B* | exon 1: ch.27: 40440576-40440921 bp<br>exon 2: ch.27: 40441875-40441978 bp<br>exon 3: ch.27: 40452510-40452686 bp | LR130458 |
| | | | | | *FGF8B* | exon 1: ch.27: 40501731-40502127 bp<br>exon 2: ch.27: 40507979-40508082 bp<br>exon 3: ch.27: 40509329-40509766 bp | LR130459 |
| | | | | | *FGF8C* | exon 1: ch.1: 107572892-107573126 bp<br>exon 2: ch.1: 107572628-107572731 bp<br>exon 3: ch.1: 107571556-107571846 bp | LR130460 |
| | | | | | putative | exon 1: ch.4: 65852050-65852422 bp<br>exon 2: ch.4: 65924095-65924198 bp<br>exon 3: ch.4: 65927995-65928192 bp | - |
| | | | | | putative | exon 1: ch.20: 57902848-57903061 bp<br>exon 2: ch.20: 57900704-57900807 bp<br>exon 3: ch.20: 57900416-57900610 bp | - |
| | | | | | putative | exon 1: ch.28: 14366957-14367170 bp<br>exon 2: ch.28: 14363681-14363787 bp<br>exon 3: ch.28: 14362527-14362817 bp | - |
| | | | | | putative | exon 1: ch.X: 108729152-108729338 bp<br>exon 2: ch.X: 108727086-108727196 bp<br>exon 3: ch.X: 108721530-108721633 bp | - |

| | | | | | | | |
|---|---|---|---|---|---|---|---|
| | | | | | | exon 4: ch.X: 108661054-108661252 bp<br>exon 5: ch.X: 108659387-108659523 bp | |
| | | Domestic cat | GCF_000181335.3 | 3 | *FGF2B* | exon 1: ch.A1: 120617552-120617720 bp<br>exon 2: ch.A1: 120602901-120603004 bp<br>exon 3: ch.A1: 120597227-120597421 bp | LR130461 |
| | | | | | *FGF6B* | exon 1: ch.B1: 36112880-36113336 bp<br>exon 2: ch.B1: 36112533-36112639 bp<br>exon 3: ch.B1: 36111183-36111476 bp | LR130462 |
| | | | | | *FGF8C* | exon 1: ch.E2: 8990533-8990770 bp<br>exon 2: ch.E2: 8990308-8990411 bp<br>exon 3: ch.E2: 8989415-8989705 bp | LR130463 |
| | | | | | putative | exon 1: ch.A1: 2510132-2510408 bp<br>exon 2: ch.A1: 2519094-2519197 bp<br>exon 3: ch.A1: 2541989-2542234 bp | - |
| | | | | | putative | exon 1: ch.A1: 72972441-72972633 bp<br>exon 2: ch.A1: 72923773-72923883 bp<br>exon 3: ch.A1: 72917704-72917807 bp<br>exon 4: ch.A1: 72803945-72804143 bp<br>exon 5: ch.A1: 72800686-72800822 bp | - |
| | | | | | putative | exon 1: ch.A1: 180276967-180276998 bp<br>exon 2: ch.A1: 180276757-180276793 bp<br>exon 3: ch.A1: 180263199-180263379 bp<br>exon 4: ch.A1: 180252178-180252284 bp<br>exon 5: ch.A1: 180245643-180245909 bp | - |
| | | | | | putative | exon 1: ch.A1: 206200214-206200580 bp<br>exon 2: ch.A1: 206276425-206276528 bp<br>exon 3: ch.A1: 206280565-206280762 bp | - |
| | | | | | putative | exon 1: ch.A2: 295073-295385 bp<br>exon 2: ch.A2: 297792-297895 bp<br>exon 3: ch.A2: 298069-298263 bp | - |
| | | | | | putative | exon 1: ch.B1: 21654731-21655016 bp<br>exon 2: ch.B1: 21660447-21660550 bp<br>exon 3: ch.B1: 21661690-21661935 bp | - |
| | | | | | putative | exon 1: ch.B1: 142185641-142186001 bp<br>exon 2: ch.B1: 142177860-142177963 bp | - |

| | | | | | | | |
|---|---|---|---|---|---|---|---|
| | | | | | | exon 3: ch.B1: 142165614-142165961 bp | |
| | | | | | putative | exon 1: ch.B3: 56630900-56631185 bp<br>exon 2: ch.B3: 56576738-56576841 bp<br>exon 3: ch.B3: 56575463-56575657 bp | - |
| | | | | | putative | exon 1: ch.B4: 39678373-39678718 bp<br>exon 2: ch.B4: 39677308-39677411 bp<br>exon 3: ch.B4: 39667611-39667787 bp | - |
| | | | | | putative | exon 1: ch.C2: 78150063-78150261 bp<br>exon 2: ch.C2: 78196613-78196723 bp<br>exon 3: ch.C2: 78225897-78226000 bp<br>exon 4: ch.C2: 78393252-78393450 bp<br>exon 5: ch.C2: 78409809-78409927 bp | - |
| | | | | | putative | exon 1: ch.D1: 113856034-113856274 bp<br>exon 2: ch.D1: 113855577-113855680 bp<br>exon 3: ch.D1: 113852486-113852800 bp | - |
| | | | | | putative | exon 1: ch.D1: 113901911-113902250 bp<br>exon 2: ch.D1: 113901288-113901391 bp<br>exon 3: ch.D1: 113900599-113900775 bp | - |
| | | | | | putative | exon 1: ch.D1: 113932492-113932711 bp<br>exon 2: ch.D1: 113930361-113930464 bp<br>exon 3: ch.D1: 113924092-113924457 bp | - |
| | | | | | putative | exon 1: ch.D2: 62329143-62329395 bp<br>exon 2: ch.D2: 62325822-62325928 bp<br>exon 3: ch.D2: 62324683-62324973 bp | - |
| | | | | | putative | exon 1: ch.E1: 2360642-2360834 bp<br>exon 2: ch.E1: 2362490-2362600 bp<br>exon 3: ch.E1: 2362807-2362910 bp<br>exon 4: ch.E1: 2363701-2363899 bp<br>exon 5: ch.E1: 2364183-2364253 bp | - |
| | | | | | putative | exon 1: ch.X: 65151111-65151384 bp<br>exon 2: ch.X: 65157493-65157596 bp<br>exon 3: ch.X: 65158933-65159178 bp | - |
| | | | | | putative | exon 1: ch.X: 116404045-116404231 bp<br>exon 2: ch.X: 116401948-116402058 bp<br>exon 3: ch.X: 116396029-116396132 bp | - |

| | | | | | | | |
|---|---|---|---|---|---|---|---|
| | | | | | | exon 4: ch.X: 116340664-116340862 bp<br>exon 5: ch.X: 116339418-116339554 bp | |
| | Chiroptera | Little brown myotis | GCF_000147115.1 | 11 | *FGF1C* | exon 1: NW_005871050.1: 1728367-1728553 bp<br>exon 2: NW_005871050.1: 1730449-1730559 bp<br>exon 3: NW_005871050.1: 1736775-1736878 bp<br>exon 4: NW_005871050.1: 1825155-1825353 bp<br>exon 5: NW_005871050.1: 1826638-1826774 bp | LR130464 |
| | | | | | *FGF2B* | exon 1: NW_005871076.1: 3432286-3432454 bp<br>exon 2: NW_005871076.1: 3448070-3448173 bp<br>exon 3: NW_005871076.1: 3452764-3452958 bp | LR130465 |
| | | | | | *FGF3A* | exon 1: NW_005871060.1: 2424907-2425252 bp<br>exon 2: NW_005871060.1: 2432593-2432696 bp<br>exon 3: NW_005871060.1: 2445243-2445593 bp | LR130466 |
| | | | | | *FGF4B* | exon 1: NW_005871067.1: 5702735-5703011 bp<br>exon 2: NW_005871067.1: 5722288-5722391 bp<br>exon 3: NW_005871067.1: 5762674-5762919 bp | LR130467 |
| | | | | | *FGF4C* | exon 1: NW_005871052.1: 6975684-6975957 bp<br>exon 2: NW_005871052.1: 6969457-6969560 bp<br>exon 3: NW_005871052.1: 6967745-6967990 bp | LR130468 |
| | | | | | *FGF5A* | exon 1: NW_005871201.1: 684565-684937 bp<br>exon 2: NW_005871201.1: 612243-612346 bp<br>exon 3: NW_005871201.1: 607644-607841 bp | LR130469 |
| | | | | | *FGF5B* | exon 1: NW_005871071.1: 7708263-7708548 bp<br>exon 2: NW_005871071.1: 7648270-7648373 bp<br>exon 3: NW_005871071.1: 7647032-7647226 bp | LR130470 |
| | | | | | *FGF5D* | exon 1: NW_005871431.1: 850169-850382 bp<br>exon 2: NW_005871431.1: 847499-847605 bp<br>exon 3: NW_005871431.1: 847218-847412 bp | LR130471 |
| | | | | | *FGF6B* | exon 1: NW_005871493.1: 2456-2696 bp<br>exon 2: NW_005871493.1: 2160-2266 bp<br>exon 3: NW_005871493.1: 392-685 bp | LR130472 |
| | | | | | *FGF7B* | exon 1: NW_005871215.1: 1291708-1292053 bp<br>exon 2: NW_005871215.1: 1293052-1293155 bp<br>exon 3: NW_005871215.1: 1302823-1302999 bp | LR130473 |
| | | | | | *FGF8A* | exon 1: NW_005871266.1: 354138-354378 bp | LR130474 |

| | | | | | | | |
|---|---|---|---|---|---|---|---|
| | | | | | | exon 2: NW_005871266.1: 353629-353732 bp<br>exon 3: NW_005871266.1: 350608-350922 bp | |
| | | | | | putative | exon 1: NW_005871738.1: 164956-165148 bp<br>exon 2: NW_005871738.1: 166508-166618 bp<br>exon 3: NW_005871738.1: 166819-166922 bp<br>exon 4: NW_005871738.1: 167639-167837 bp<br>exon 5: NW_005871738.1: 168099-168169 bp | - |
| | | Large flying fox | GCF_000151845.1 | 7 | *FGF1D* | exon 1: NW_011888825.1: 154776-154968 bp<br>exon 2: NW_011888825.1: 156668-156778 bp<br>exon 3: NW_011888825.1: 156966-157069 bp<br>exon 4: NW_011888825.1: 157845-158043 bp<br>exon 5: NW_011888825.1: 158321-158391 bp | LR130475 |
| | | | | | *FGF2B* | exon 1: NW_011888915.1: 1224564-1224732 bp<br>exon 2: NW_011888915.1: 1210975-1211078 bp<br>exon 3: NW_011888915.1: 1206561-1206755 bp | LR130476 |
| | | | | | *FGF4A* | exon 1: NW_011888889.1: 2494145-2494430 bp<br>exon 2: NW_011888889.1: 2499851-2499954 bp<br>exon 3: NW_011888889.1: 2501451-2501696 bp | LR130477 |
| | | | | | *FGF4B* | exon 1: NW_011888833.1: 4236632-4236908 bp<br>exon 2: NW_011888833.1: 4245021-4245124 bp<br>exon 3: NW_011888833.1: 4261284-4261529 bp | LR130478 |
| | | | | | *FGF4C* | exon 1: NW_011889416.1: 892032-892305 bp<br>exon 2: NW_011889416.1: 886059-886162 bp<br>exon 3: NW_011889416.1: 884443-884688 bp | LR130479 |
| | | | | | *FGF5D* | exon 1: NW_011889048.1: 766210-766423 bp<br>exon 2: NW_011889048.1: 763900-764003 bp<br>exon 3: NW_011889048.1: 763625-763819 bp | LR130480 |
| | | | | | *FGF8A* | exon 1: NW_011889259.1: 233769-234000 bp<br>exon 2: NW_011889259.1: 234413-234516 bp<br>exon 3: NW_011889259.1: 237476-237790 bp | LR130481 |
| | | | | | putative | exon 1: NW_011888795.1: 3088998-3089196 bp<br>exon 2: NW_011888795.1: 3133246-3133356 bp<br>exon 3: NW_011888795.1: 3156800-3156903 bp<br>exon 4: NW_011888795.1: 3305433-3305631 bp<br>exon 5: NW_011888795.1: 3325982-3326100 bp | - |

| | | | | | | | |
|---|---|---|---|---|---|---|---|
| | | | | | putative | exon 1: NW_011888800.1: 13221227-13221512 bp<br>exon 2: NW_011888800.1: 13277905-13278008 bp<br>exon 3: NW_011888800.1: 13279083-13279277 bp | - |
| | | | | | putative | exon 1: NW_011888837.1: 7594248-7594605 bp<br>exon 2: NW_011888837.1: 7602792-7602895 bp<br>exon 3: NW_011888837.1: 7616283-7616630 bp | - |
| | | | | | putative | exon 1: NW_011888851.1: 7870807-7871152 bp<br>exon 2: NW_011888851.1: 7872131-7872234 bp<br>exon 3: NW_011888851.1: 7881118-7881294 bp | - |
| | | | | | putative | exon 1: NW_011888851.1: 7939361-7939556 bp<br>exon 2: NW_011888851.1: 7945176-7945279 bp<br>exon 3: NW_011888851.1: 7946792-7947280 bp | - |
| | | | | | putative | exon 1: NW_011888864.1: 4597285-4597477 bp<br>exon 2: NW_011888864.1: 4643261-4643371 bp<br>exon 3: NW_011888864.1: 4652843-4652946 bp<br>exon 4: NW_011888864.1: 4764764-4764962 bp<br>exon 5: NW_011888864.1: 4767025-4767161 bp | - |
| | | | | | putative | exon 1: NW_011888907.1: 872739-873069 bp<br>exon 2: NW_011888907.1: 872383-872489 bp<br>exon 3: NW_011888907.1: 871016-871309 bp | - |
| | | | | | putative | exon 1: NW_011889015.1: 849570-849909 bp<br>exon 2: NW_011889015.1: 784094-784197 bp<br>exon 3: NW_011889015.1: 779785-779982 bp | - |
| | | | | | putative | exon 1: NW_011889099.1: 566201-566387 bp<br>exon 2: NW_011889099.1: 564150-564260 bp<br>exon 3: NW_011889099.1: 558781-558884 bp<br>exon 4: NW_011889099.1: 503219-503417 bp<br>exon 5: NW_011889099.1: 501790-501926 bp | - |
| | | | | | putative | exon 1: NW_011889129.1: 2442852-2443104 bp<br>exon 2: NW_011889129.1: 2439528-2439634 bp<br>exon 3: NW_011889129.1: 2438385-2438675 bp | - |
| | | | | | putative | exon 1: NW_011917830.1: 544-883 bp<br>exon 2: NW_011917830.1: 1512-1615 bp<br>exon 3: NW_011917830.1: 2136-2312 bp | - |
| | Eulipotyphla | West European hedgehog | GCF_000296755.1 | 0 | putative | exon 1: NW_006803932.1: 5456925-5457117 bp | - |

| | | | | | | | |
|---|---|---|---|---|---|---|---|
| | | | | | | exon 2: NW_006803932.1: 5458767-5458877 bp<br>exon 3: NW_006803932.1: 5459107-5459210 bp<br>exon 4: NW_006803932.1: 5463165-5463363 bp<br>exon 5: NW_006803932.1: 5463651-5463721 bp | |
| | | | | | putative | exon 1: NW_006803963.1: 1662467-1662659 bp<br>exon 2: NW_006803963.1: 1621057-1621167 bp<br>exon 3: NW_006803963.1: 1614562-1614665 bp<br>exon 4: NW_006803963.1: 1450599-1450797 bp<br>exon 5: NW_006803963.1: 1441542-1441678 bp | - |
| | | | | | putative | exon 1: NW_006803974.1: 1944756-1945041 bp<br>exon 2: NW_006803974.1: 1938407-1938510 bp<br>exon 3: NW_006803974.1: 1935546-1935791 bp | - |
| | | | | | putative | exon 1: NW_006803993.1: 4847661-4847946 bp<br>exon 2: NW_006803993.1: 4912128-4912231 bp<br>exon 3: NW_006803993.1: 4913490-4913684 bp | - |
| | | | | | putative | exon 1: NW_006803996.1: 1156868-1157066 bp<br>exon 2: NW_006803996.1: 1104324-1104434 bp<br>exon 3: NW_006803996.1: 1077034-1077137 bp<br>exon 4: NW_006803996.1: 924574-924772 bp<br>exon 5: NW_006803996.1: 908851-908969 bp | - |
| | | | | | putative | exon 1: NW_006803999.1: 4253660-4254029 bp<br>exon 2: NW_006803999.1: 4166820-4166923 bp<br>exon 3: NW_006803999.1: 4161992-4162189 bp | - |
| | | | | | putative | exon 1: NW_006804012.1: 4708101-4708440 bp<br>exon 2: NW_006804012.1: 4700534-4700637 bp<br>exon 3: NW_006804012.1: 4685467-4685814 bp | - |
| | | | | | putative | exon 1: NW_006804019.1: 1693475-1693643 bp<br>exon 2: NW_006804019.1: 1668715-1668818 bp<br>exon 3: NW_006804019.1: 1663483-1663677 bp | - |
| | | | | | putative | exon 1: NW_006804167.1: 2745979-2746165 bp<br>exon 2: NW_006804167.1: 2743949-2744059 bp<br>exon 3: NW_006804167.1: 2736089-2736192 bp<br>exon 4: NW_006804167.1: 2645668-2645866 bp<br>exon 5: NW_006804167.1: 2644037-2644173 bp | - |
| | | | | | putative | exon 1: NW_006804178.1: 159316-159493 bp<br>exon 2: NW_006804178.1: 161320-161423 bp | - |

| | | | | | | | |
|---|---|---|---|---|---|---|---|
| | | | | | | exon 3: NW_006804178.1: 161542-161736 bp | |
| | | | | | putative | exon 1: NW_006804216.1: 2459387-2459582 bp<br>exon 2: NW_006804216.1: 2450884-2450987 bp<br>exon 3: NW_006804216.1: 2448895-2449335 bp | - |
| | | | | | putative | exon 1: NW_006804216.1: 2560676-2561234 bp<br>exon 2: NW_006804216.1: 2558858-2558961 bp<br>exon 3: NW_006804216.1: 2541972-2542148 bp | - |
| | | | | | putative | exon 1: NW_006804340.1: 1419459-1419852 bp<br>exon 2: NW_006804340.1: 1419191-1419297 bp<br>exon 3: NW_006804340.1: 1418184-1418477 bp | - |
| | | | | | putative | exon 1: NW_006804352.1: 1280242-1280518 bp<br>exon 2: NW_006804352.1: 1289576-1289679 bp<br>exon 3: NW_006804352.1: 1326126-1326371 bp | - |
| | | | | | putative | exon 1: NW_006804639.1: 1178470-1178743 bp<br>exon 2: NW_006804639.1: 1172121-1172224 bp<br>exon 3: NW_006804639.1: 1153751-1153996 bp | - |
| | | | | | putative | exon 1: NW_006804793.1: 94216-94447 bp<br>exon 2: NW_006804793.1: 94818-94921 bp<br>exon 3: NW_006804793.1: 96840-97115 bp | - |
| | | Common shrew | GCF_000181275.1 | 2 | *FGF5B* | exon 1: NW_004545905.1: 10049829-10050114 bp<br>exon 2: NW_004545905.1: 9994541-9994644 bp<br>exon 3: NW_004545905.1: 9992496-9992690 bp | LR130482 |
| | | | | | *FGF8C* | exon 1: NW_004546001.1: 1231611-1231845 bp<br>exon 2: NW_004546001.1: 1232503-1232606 bp<br>exon 3: NW_004546001.1: 1233761-1234054 bp | LR130483 |
| | | | | | putative | exon 1: NW_004545863.1: 24060834-24061032 bp<br>exon 2: NW_004545863.1: 24111501-24111611 bp<br>exon 3: NW_004545863.1: 24145352-24145455 bp<br>exon 4: NW_004545863.1: 24293358-24293556 bp<br>exon 5: NW_004545863.1: 24310795-24310913 bp | - |
| | | | | | putative | exon 1: NW_004545868.1: 6460451-6460727 bp<br>exon 2: NW_004545868.1: 6451522-6451625 bp<br>exon 3: NW_004545868.1: 6415956-6416201 bp | - |
| | | | | | putative | exon 1: NW_004545869.1: 21689649-21689841 bp<br>exon 2: NW_004545869.1: 21687999-21688109 bp | - |

| | | | | | | | |
|---|---|---|---|---|---|---|---|
| | | | | | | exon 3: NW_004545869.1: 21687704-21687807 bp<br>exon 4: NW_004545869.1: 21686813-21687011 bp<br>exon 5: NW_004545869.1: 21686490-21686560 bp | |
| | | | | | putative | exon 1: NW_004545877.1: 23792221-23792407 bp<br>exon 2: NW_004545877.1: 23794340-23794450 bp<br>exon 3: NW_004545877.1: 23800482-23800585 bp<br>exon 4: NW_004545877.1: 23868169-23868367 bp<br>exon 5: NW_004545877.1: 23870206-23870342 bp | - |
| | | | | | putative | exon 1: NW_004545888.1: 1142306-1142684 bp<br>exon 2: NW_004545888.1: 1059802-1059905 bp<br>exon 3: NW_004545888.1: 1054646-1054843 bp | - |
| | | | | | putative | exon 1: NW_004545897.1: 3215870-3216155 bp<br>exon 2: NW_004545897.1: 3209644-3209747 bp<br>exon 3: NW_004545897.1: 3207935-3208180 bp | - |
| | | | | | putative | exon 1: NW_004545898.1: 3056918-3057101 bp<br>exon 2: NW_004545898.1: 3048728-3048831 bp<br>exon 3: NW_004545898.1: 3046244-3046675 bp | - |
| | | | | | putative | exon 1: NW_004545898.1: 3133686-3134010 bp<br>exon 2: NW_004545898.1: 3132245-3132348 bp<br>exon 3: NW_004545898.1: 3118076-3118252 bp | - |
| | | | | | putative | exon 1: NW_004545901.1: 14987285-14987453 bp<br>exon 2: NW_004545901.1: 15001060-15001163 bp<br>exon 3: NW_004545901.1: 15006168-15006362 bp | - |
| | | | | | putative | exon 1: NW_004545904.1: 916176-916449 bp<br>exon 2: NW_004545904.1: 923599-923702 bp<br>exon 3: NW_004545904.1: 925310-925555 bp | - |
| | | | | | putative | exon 1: NW_004545925.1: 5210815-5211007 bp<br>exon 2: NW_004545925.1: 5174826-5174936 bp<br>exon 3: NW_004545925.1: 5169273-5169376 bp<br>exon 4: NW_004545925.1: 5040777-5040975 bp<br>exon 5: NW_004545925.1: 5037152-5037288 bp | - |
| | | | | | putative | exon 1: NW_004545929.1: 8002762-8003173 bp<br>exon 2: NW_004545929.1: 7994721-7994824 bp<br>exon 3: NW_004545929.1: 7980407-7980754 bp | - |
| | | | | | putative | exon 1: NW_004545935.1: 4252276-4252489 bp | - |

| | | | | | | | |
|---|---|---|---|---|---|---|---|
| | | | | | | exon 2: NW_004545935.1: 4255068-4255171 bp<br>exon 3: NW_004545935.1: 4255360-4255554 bp | |
| | | | | | putative | exon 1: NW_004545975.1: 3989680-3989917 bp<br>exon 2: NW_004545975.1: 3989292-3989395 bp<br>exon 3: NW_004545975.1: 3986902-3987216 bp | - |
| Xenarthra | Xenarthra | Nine-banded armadillo | GCF_000208655.1 | 14 | *FGF1A* | exon 1: NW_004480579.1: 3831948-3832146 bp<br>exon 2: NW_004480579.1: 3772930-3773040 bp<br>exon 3: NW_004480579.1: 3746114-3746217 bp<br>exon 4: NW_004480579.1: 3597827-3598025 bp<br>exon 5: NW_004480579.1: 3577524-3577642 bp | LR130484 |
| | | | | | *FGF1B* | exon 1: NW_004479389.1: 5895942-5896134 bp<br>exon 2: NW_004479389.1: 5962694-5962804 bp<br>exon 3: NW_004479389.1: 5969321-5969424 bp<br>exon 4: NW_004479389.1: 6143538-6143736 bp<br>exon 5: NW_004479389.1: 6146932-6147068 bp | LR130485 |
| | | | | | *FGF1C* | exon 1: NW_004501391.1: 261907-262093 bp<br>exon 2: NW_004501391.1: 264053-264163 bp<br>exon 3: NW_004501391.1: 269429-269532 bp<br>exon 4: NW_004501391.1: 339453-339651 bp<br>exon 5: NW_004501391.1: 343542-343678 bp | LR130486 |
| | | | | | *FGF1D* | exon 1: NW_004482888.1: 107576-107768 bp<br>exon 2: NW_004482888.1: 109381-109491 bp<br>exon 3: NW_004482888.1: 109687-109790 bp<br>exon 4: NW_004482888.1: 110362-110560 bp<br>exon 5: NW_004482888.1: 110848-110918 bp | LR130487 |
| | | | | | *FGF2A* | exon 1: NW_004488157.1: 1335237-1335414 bp<br>exon 2: NW_004488157.1: 1287688-1287791 bp<br>exon 3: NW_004488157.1: 1267427-1267612 bp | LR130488 |
| | | | | | *FGF2B* | exon 1: NW_004468898.1: 1740583-1740751 bp<br>exon 2: NW_004468898.1: 1725013-1725116 bp<br>exon 3: NW_004468898.1: 1720529-1720723 bp | LR130489 |
| | | | | | *FGF3A* | exon 1: NW_004483432.1: 2367021-2367360 bp<br>exon 2: NW_004483432.1: 2375734-2375837 bp<br>exon 3: NW_004483432.1: 2387327-2387674 bp | LR130490 |
| | | | | | *FGF4B* | exon 1: NW_004482607.1: 1417908-1418184 bp<br>exon 2: NW_004482607.1: 1428246-1428349 bp | LR130491 |

| | | | | | | | |
|---|---|---|---|---|---|---|---|
| | | | | | | exon 3: NW_004482607.1: 1449933-1450178 bp | |
| | | | | | *FGF4C* | exon 1: NW_004498295.1: 423513-423786 bp<br>exon 2: NW_004498295.1: 430926-431029 bp<br>exon 3: NW_004498295.1: 432372-432617 bp | LR130492 |
| | | | | | *FGF5A* | exon 1: NW_004485155.1: 1217874-1218216 bp<br>exon 2: NW_004485155.1: 1145572-1145675 bp<br>exon 3: NW_004485155.1: 1142624-1142821 bp | LR130493 |
| | | | | | *FGF5B* | exon 1: NW_004483542.1: 854227-854512 bp<br>exon 2: NW_004483542.1: 799579-799682 bp<br>exon 3: NW_004483542.1: 798290-798484 bp | LR130494 |
| | | | | | *FGF6B* | exon 1: NW_004480463.1: 175033-175156 bp<br>exon 2: NW_004480463.1: 175409-175515 bp<br>exon 3: NW_004480463.1: 176372-176665 bp | LR130495 |
| | | | | | *FGF8A* | exon 1: NW_004480359.1: 290938-291175 bp<br>exon 2: NW_004480359.1: 290649-290752 bp<br>exon 3: NW_004480359.1: 287988-288299 bp | LR130496 |
| | | | | | *FGF8B* | exon 1: NW_004480308.1: 840005-840206 bp<br>exon 2: NW_004480308.1: 835045-835148 bp<br>exon 3: NW_004480308.1: 833260-833700 bp | LR130497 |
| | | | | | putative | exon 1: NW_004458668.1: 365706-366261 bp<br>exon 2: NW_004458668.1: 369288-369394 bp<br>exon 3: NW_004458668.1: 370977-371267 bp | - |
| | | | | | putative | exon 1: NW_004480308.1: 902917-903262 bp<br>exon 2: NW_004480308.1: 901910-902013 bp<br>exon 3: NW_004480308.1: 892652-892828 bp | - |
| | | Hoffmann's two-toed sloth | GCA_000164785.2 | 0 | putative | exon 1: KN179307.1: 225062-225416 bp<br>exon 2: KN179307.1: 224734-224840 bp<br>exon 3: KN179307.1: 223359-223652 bp | - |
| | | | | | putative | exon 1: KN180088.1: 106571-106937 bp<br>exon 2: KN180088.1: 97475-97578 bp<br>exon 3: KN180088.1: 86484-86831 bp | - |
| | | | | | putative | exon 1: KN182184.1: 366461-366492 bp<br>exon 2: KN182184.1: 366261-366297 bp<br>exon 3: KN182184.1: 351511-351691 bp<br>exon 4: KN182184.1: 339813-339919 bp | - |

| | | | | | | | |
|---|---|---|---|---|---|---|---|
| | | | | | | exon 5: KN182184.1: 332586-332852 bp | |
| | | | | | putative | exon 1: KN185152.1: 446714-446954 bp<br>exon 2: KN185152.1: 446368-446471 bp<br>exon 3: KN185152.1: 443587-443901 bp | - |
| | | | | | putative | exon 1: KN185303.1: 105279-105558 bp<br>exon 2: KN185303.1: 110870-110973 bp<br>exon 3: KN185303.1: 112793-113038 bp | - |
| | | | | | putative | exon 1: KN190330.1: 810645-810879 bp<br>exon 2: KN190330.1: 810970-811073 bp<br>exon 3: KN190330.1: 811627-811899 bp | - |
| | | | | | putative | exon 1: KN190564.1: 376295-376640 bp<br>exon 2: KN190564.1: 375199-375302 bp<br>exon 3: KN190564.1: 366432-366608 bp | - |
| | | | | | putative | exon 1: KN191318.1: 600360-600636 bp<br>exon 2: KN191318.1: 590829-590932 bp<br>exon 3: KN191318.1: 571318-571563 bp | - |
| | | | | | putative | exon 1: KN192666.1: 548183-548351 bp<br>exon 2: KN192666.1: 558595-558698 bp<br>exon 3: KN192666.1: 563093-563287 bp | - |
| | | | | | putative | exon 1: KN193114.1: 408944-409283 bp<br>exon 2: KN193114.1: 323535-323638 bp<br>exon 3: KN193114.1: 316334-316531 bp | - |
| | | | | | putative | exon 1: KN195243.1: 99345-99522 bp<br>exon 2: KN195243.1: 43566-43669 bp<br>exon 3: KN195243.1: 28773-28958 bp | - |
| | | | | | putative | exon 1: KN196003.1: 106767-107052 bp<br>exon 2: KN196003.1: 40802-40905 bp<br>exon 3: KN196003.1: 39515-39709 bp | - |
| Afrotheria | Tenrecidae | Lesser hedgehog tenrec | GCF_000313985.1 | 0 | putative | exon 1: NW_004558702.1: 29995854-29996022 bp<br>exon 2: NW_004558702.1: 29979464-29979567 bp<br>exon 3: NW_004558702.1: 29972311-29972505 bp | - |
| | | | | | putative | exon 1: NW_004558702.1: 65904034-65904065 bp<br>exon 2: NW_004558702.1: 65904239-65904275 bp<br>exon 3: NW_004558702.1: 65921835-65922015 bp<br>exon 4: NW_004558702.1: 65933697-65933803 bp | - |

| | | | | | | | |
|---|---|---|---|---|---|---|---|
| | | | | | | exon 5: NW_004558702.1: 65941733-65941999 bp | |
| | | | | | putative | exon 1: NW_004558715.1: 19665507-19665867 bp<br>exon 2: NW_004558715.1: 19674848-19674951 bp<br>exon 3: NW_004558715.1: 19690013-19690360 bp | - |
| | | | | | putative | exon 1: NW_004558719.1: 12405146-12405338 bp<br>exon 2: NW_004558719.1: 12355485-12355595 bp<br>exon 3: NW_004558719.1: 12350425-12350528 bp<br>exon 4: NW_004558719.1: 12178137-12178335 bp<br>exon 5: NW_004558719.1: 12161422-12161558 bp | - |
| | | | | | putative | exon 1: NW_004558728.1: 9671371-9671494 bp<br>exon 2: NW_004558728.1: 9671719-9671825 bp<br>exon 3: NW_004558728.1: 9672851-9673144 bp | - |
| | | | | | putative | exon 1: NW_004558738.1: 7872662-7872947 bp<br>exon 2: NW_004558738.1: 7930556-7930659 bp<br>exon 3: NW_004558738.1: 7931695-7931889 bp | - |
| | | | | | putative | exon 1: NW_004558746.1: 14102474-14102708 bp<br>exon 2: NW_004558746.1: 14103452-14103555 bp<br>exon 3: NW_004558746.1: 14104159-14104443 bp | - |
| | | | | | putative | exon 1: NW_004558763.1: 10739692-10739890 bp<br>exon 2: NW_004558763.1: 10799727-10799837 bp<br>exon 3: NW_004558763.1: 10834306-10834409 bp<br>exon 4: NW_004558763.1: 10998090-10998288 bp<br>exon 5: NW_004558763.1: 11027540-11027658 bp | - |
| | | | | | putative | exon 1: NW_004558770.1: 1096546-1096738 bp<br>exon 2: NW_004558770.1: 1098411-1098521 bp<br>exon 3: NW_004558770.1: 1098679-1098782 bp<br>exon 4: NW_004558770.1: 1099363-1099561 bp<br>exon 5: NW_004558770.1: 1099811-1099881 bp | - |
| | | | | | putative | exon 1: NW_004558777.1: 6970623-6970863 bp<br>exon 2: NW_004558777.1: 6970361-6970464 bp<br>exon 3: NW_004558777.1: 6966861-6967166 bp | - |
| | | | | | putative | exon 1: NW_004558794.1: 4377123-4377399 bp<br>exon 2: NW_004558794.1: 4363593-4363696 bp<br>exon 3: NW_004558794.1: 4327231-4327476 bp | - |
| | | | | | putative | exon 1: NW_004558860.1: 1056847-1057237 bp | - |

| | | | | | | | |
|---|---|---|---|---|---|---|---|
| | | | | | | exon 2: NW_004558860.1: 1050226-1050329 bp<br>exon 3: NW_004558860.1: 1047495-1047968 bp | |
| | | | | | putative | exon 1: NW_004558860.1: 1134715-1135060 bp<br>exon 2: NW_004558860.1: 1133272-1133375 bp<br>exon 3: NW_004558860.1: 1118605-1118781 bp | - |
| | Proboscidea | African bush elephant | GCF_000001905.1 | 9 | *FGF1C* | exon 1: NW_003573509.1: 2483153-2483339 bp<br>exon 2: NW_003573509.1: 2485224-2485334 bp<br>exon 3: NW_003573509.1: 2490993-2491096 bp<br>exon 4: NW_003573509.1: 2546053-2546251 bp<br>exon 5: NW_003573509.1: 2547783-2547919 bp | LR130498 |
| | | | | | *FGF1D* | exon 1: NW_003573467.1: 11451443-11451635 bp<br>exon 2: NW_003573467.1: 11453294-11453404 bp<br>exon 3: NW_003573467.1: 11453602-11453705 bp<br>exon 4: NW_003573467.1: 11454256-11454454 bp<br>exon 5: NW_003573467.1: 11454739-11454809 bp | LR130499 |
| | | | | | *FGF2B* | exon 1: NW_003573421.1: 60478320-60478488 bp<br>exon 2: NW_003573421.1: 60463654-60463757 bp<br>exon 3: NW_003573421.1: 60457452-60457646 bp | LR130500 |
| | | | | | *FGF4B* | exon 1: NW_003573449.1: 6028859-6029135 bp<br>exon 2: NW_003573449.1: 6039157-6039260 bp<br>exon 3: NW_003573449.1: 6069205-6069450 bp | LR130501 |
| | | | | | *FGF5A* | exon 1: NW_003573427.1: 49365579-49365918 bp<br>exon 2: NW_003573427.1: 49270597-49270700 bp<br>exon 3: NW_003573427.1: 49268488-49268685 bp | LR130502 |
| | | | | | *FGF5B* | exon 1: NW_003573500.1: 1634222-1634507 bp<br>exon 2: NW_003573500.1: 1564671-1564774 bp<br>exon 3: NW_003573500.1: 1562992-1563186 bp | LR130503 |
| | | | | | *FGF6B* | exon 1: NW_003573442.1: 14544343-14544640 bp<br>exon 2: NW_003573442.1: 14543968-14544074 bp<br>exon 3: NW_003573442.1: 14542577-14542870 bp | LR130504 |
| | | | | | *FGF7B* | exon 1: NW_003573435.1: 48977591-48977936 bp<br>exon 2: NW_003573435.1: 48976011-48976114 bp<br>exon 3: NW_003573435.1: 48963567-48963743 bp | LR130505 |
| | | | | | *FGF8B* | exon 1: NW_003573435.1: 48892618-48893005 bp<br>exon 2: NW_003573435.1: 48886018-48886121 bp | LR130506 |

| | | | | | | | |
|---|---|---|---|---|---|---|---|
| | | | | | | exon 3: NW_003573435.1: 48883535-48883975 bp | |
| | | | | | putative | exon 1: NW_003573491.1: 22073-22385 bp<br>exon 2: NW_003573491.1: 22868-22971 bp<br>exon 3: NW_003573491.1: 23530-23706 bp | - |
| | Hyracoidea | Rock hyrax | GCA_000152225.2 | 2 | *FGF7A* | exon 1: KN676416.1: 593893-594205 bp<br>exon 2: KN676416.1: 593308-593411 bp<br>exon 3: KN676416.1: 592567-592743 bp | LR130507 |
| | | | | | *FGF8C* | exon 1: KN678459.1: 66397-66631 bp<br>exon 2: KN678459.1: 65735-65838 bp<br>exon 3: KN678459.1: 64579-64869 bp | LR130508 |
| | | | | | putative | exon 1: KN676151.1: 2927528-2927873 bp<br>exon 2: KN676151.1: 2929530-2929633 bp<br>exon 3: KN676151.1: 2941700-2941876 bp | - |
| | | | | | putative | exon 1: KN676151.1: 3014235-3014445 bp<br>exon 2: KN676151.1: 3022928-3023031 bp<br>exon 3: KN676151.1: 3025602-3026042 bp | - |
| | | | | | putative | exon 1: KN676186.1: 1968191-1968599 bp<br>exon 2: KN676186.1: 1968875-1968981 bp<br>exon 3: KN676186.1: 1970014-1970307 bp | - |
| | | | | | putative | exon 1: KN676194.1: 1394894-1395257 bp<br>exon 2: KN676194.1: 1383640-1383743 bp<br>exon 3: KN676194.1: 1369493-1369840 bp | - |
| | | | | | putative | exon 1: KN676204.1: 1372040-1372208 bp<br>exon 2: KN676204.1: 1390899-1391002 bp<br>exon 3: KN676204.1: 1397135-1397329 bp | - |
| | | | | | putative | exon 1: KN676211.1: 1372644-1372824 bp<br>exon 2: KN676211.1: 1450606-1450709 bp<br>exon 3: KN676211.1: 1466508-1466693 bp | - |
| | | | | | putative | exon 1: KN676291.1: 277698-277911 bp<br>exon 2: KN676291.1: 280220-280323 bp<br>exon 3: KN676291.1: 280421-280615 bp | - |
| | | | | | putative | exon 1: KN676398.1: 455967-456153 bp<br>exon 2: KN676398.1: 453983-454093 bp<br>exon 3: KN676398.1: 447824-447927 bp<br>exon 4: KN676398.1: 386241-386439 bp | - |

| | | | | | | | |
|---|---|---|---|---|---|---|---|
| | | | | | | exon 5: KN676398.1: 384090-384226 bp | |
| | | | | | putative | exon 1: KN676416.1: 520659-520899 bp<br>exon 2: KN676416.1: 520313-520416 bp<br>exon 3: KN676416.1: 516379-516690 bp | - |
| | | | | | putative | exon 1: KN676634.1: 82677-82953 bp<br>exon 2: KN676634.1: 72849-72952 bp<br>exon 3: KN676634.1: 43169-43414 bp | - |
| | | | | | putative | exon 1: KN676712.1: 1374144-1374175 bp<br>exon 2: KN676712.1: 1374337-1374373 bp<br>exon 3: KN676712.1: 1397811-1397991 bp<br>exon 4: KN676712.1: 1411695-1411801 bp<br>exon 5: KN676712.1: 1417839-1418105 bp | - |
| | | | | | putative | exon 1: KN676808.1: 1410628-1410841 bp<br>exon 2: KN676808.1: 1407344-1407450 bp<br>exon 3: KN676808.1: 1406195-1406485 bp | - |
| | | | | | putative | exon 1: KN677699.1: 107150-107342 bp<br>exon 2: KN677699.1: 105422-105532 bp<br>exon 3: KN677699.1: 105114-105217 bp<br>exon 4: KN677699.1: 104215-104413 bp<br>exon 5: KN677699.1: 103827-103897 bp | - |
| | | | | | putative | exon 1: KN678147.1: 683044-683371 bp<br>exon 2: KN678147.1: 775855-775958 bp<br>exon 3: KN678147.1: 777913-778110 bp | - |
| | | | | | putative | exon 1: KN679308.1: 157767-158052 bp<br>exon 2: KN679308.1: 59805-59908 bp<br>exon 3: KN679308.1: 58380-58574 bp | - |
| | | | | | putative | exon 1: KN679931.1: 68026-68299 bp<br>exon 2: KN679931.1: 74437-74540 bp<br>exon 3: KN679931.1: 76017-76262 bp | - |

[a], the eutherian species common names were cited from Wilson and Reeder [30]; [b], the human *FGF* gene nomenclature provided in parentheses was cited from Goldfarb [1], Belov and Mohammadi [2] and Ornitz and Itoh [3]; [c], translated exons; ch., chromosome.
