# Supplementary material for: Comparative genomic analysis of eutherian fibroblast growth factor genes
Source: BMC Genomics. 2020 Aug 5;21:542. doi: 10.1186/s12864-020-06958-4 (PMC7430813; doi:10.1186/s12864-020-06958-4)
Supplement: Supplementary file 2 — Additional file 2 Multiple pairwise genomic sequence alignments of eutherian fibroblast growth factor genes. The FGF coding exon sequence regions in base sequences (Homo sapiens) were displayed as indigo rectangles, and grey arrows indicated their relative orientation (top). The genomic sequence regions including sequence identity levels above empirical cut-offs of detection of common genomic sequence regions were shown accordingly in multiple pairwise alignments. [file 12864_2020_6958_MOESM2_ESM.pdf]

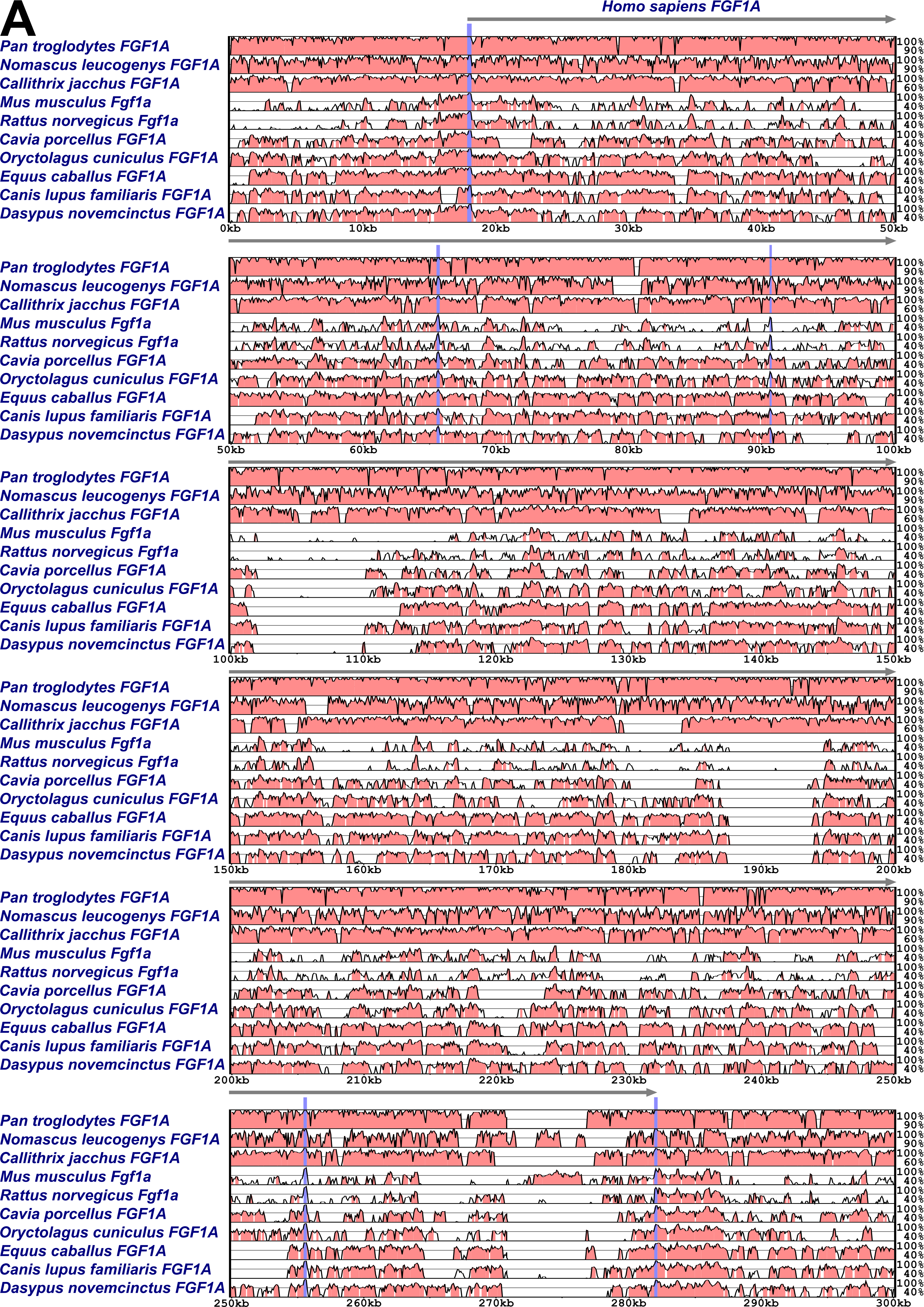
A
Homo sapiens FGF1A
Pan troglodytes FGF1A
Nomascus leucogenys FGF1A
Callithrix jacchus FGF1A
Mus musculus Fgf1a
Rattus norvegicus Fgf1a
Cavia porcellus FGF1A
Oryctolagus cuniculus FGF1A
Equus caballus FGF1A
Canis lupus familiaris FGF1A
Dasypus novemcinctus FGF1A
100%
90%
60%
40%
0kb
10kb
20kb
30kb
40kb
50kb
60kb
70kb
80kb
90kb
100kb
110kb
120kb
130kb
140kb
150kb
160kb
170kb
180kb
190kb
200kb
210kb
220kb
230kb
240kb
250kb
260kb
270kb
280kb
290kb
300kb

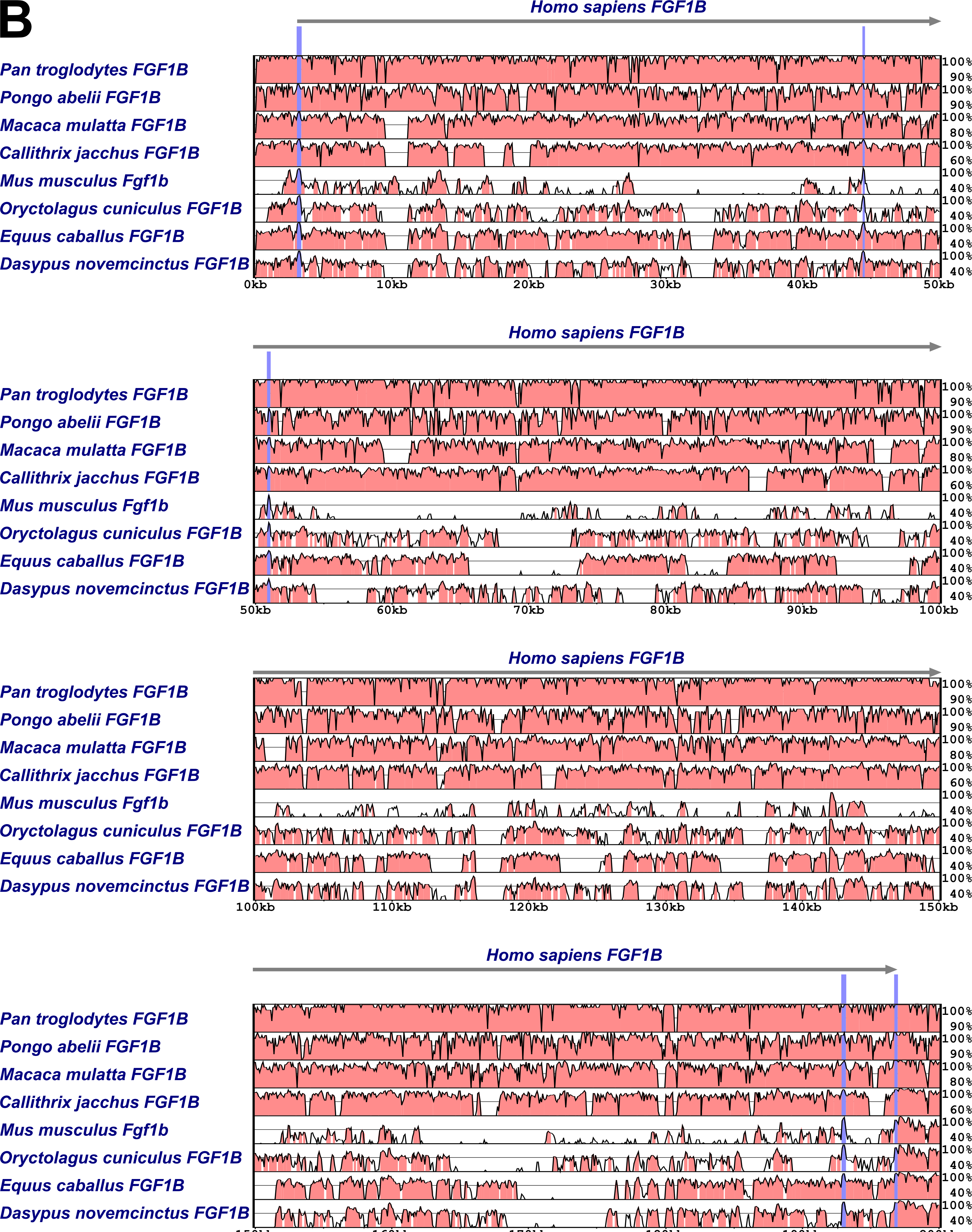
B
Homo sapiens FGF1B
Pan troglodytes FGF1B
Pongo abelii FGF1B
Macaca mulatta FGF1B
Callithrix jacchus FGF1B
Mus musculus Fgf1b
Oryctolagus cuniculus FGF1B
Equus caballus FGF1B
Dasypus novemcinctus FGF1B
100%
90%
80%
60%
40%
0kb
10kb
20kb
30kb
40kb
50kb
Homo sapiens FGF1B
Pan troglodytes FGF1B
Pongo abelii FGF1B
Macaca mulatta FGF1B
Callithrix jacchus FGF1B
Mus musculus Fgf1b
Oryctolagus cuniculus FGF1B
Equus caballus FGF1B
Dasypus novemcinctus FGF1B
50kb
60kb
70kb
80kb
90kb
100kb
Homo sapiens FGF1B
Pan troglodytes FGF1B
Pongo abelii FGF1B
Macaca mulatta FGF1B
Callithrix jacchus FGF1B
Mus musculus Fgf1b
Oryctolagus cuniculus FGF1B
Equus caballus FGF1B
Dasypus novemcinctus FGF1B
100kb
110kb
120kb
130kb
140kb
150kb
Homo sapiens FGF1B
Pan troglodytes FGF1B
Pongo abelii FGF1B
Macaca mulatta FGF1B
Callithrix jacchus FGF1B
Mus musculus Fgf1b
Oryctolagus cuniculus FGF1B
Equus caballus FGF1B
Dasypus novemcinctus FGF1B
150kb
160kb
170kb
180kb
190kb
200kb

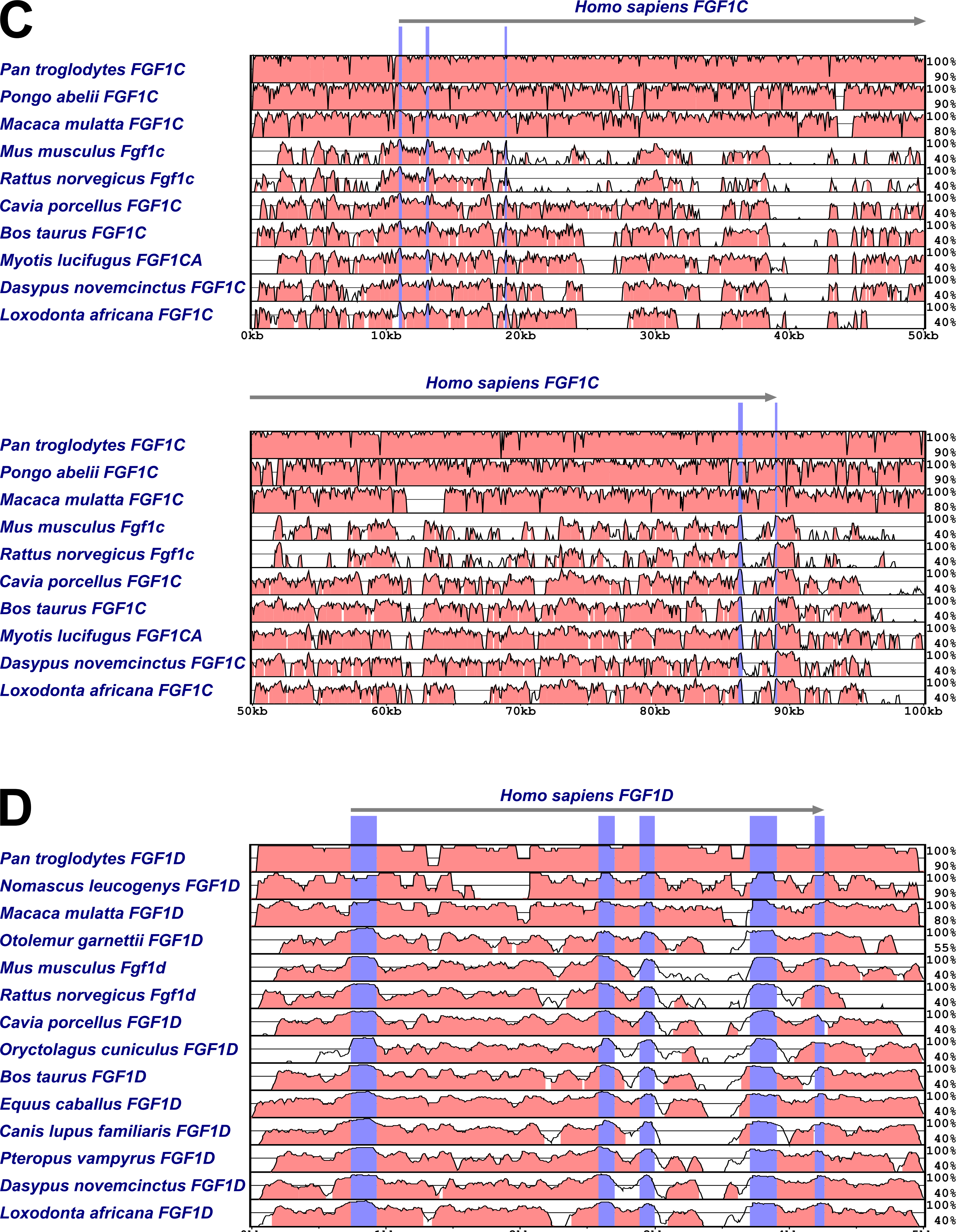
C
Homo sapiens FGF1C
Pan troglodytes FGF1C
Pongo abelii FGF1C
Macaca mulatta FGF1C
Mus musculus Fgf1c
Rattus norvegicus Fgf1c
Cavia porcellus FGF1C
Bos taurus FGF1C
Myotis lucifugus FGF1CA
Dasypus novemcinctus FGF1C
Loxodonta africana FGF1C
0kb
10kb
20kb
30kb
40kb
50kb
60kb
70kb
80kb
90kb
100kb
100%
90%
80%
40%
D
Homo sapiens FGF1D
Pan troglodytes FGF1D
Nomascus leucogenys FGF1D
Macaca mulatta FGF1D
Otolemur garnettii FGF1D
Mus musculus Fgf1d
Rattus norvegicus Fgf1d
Cavia porcellus FGF1D
Oryctolagus cuniculus FGF1D
Bos taurus FGF1D
Equus caballus FGF1D
Canis lupus familiaris FGF1D
Pteropus vampyrus FGF1D
Dasypus novemcinctus FGF1D
Loxodonta africana FGF1D
1kb
2kb
3kb
4kb
5kb
55%

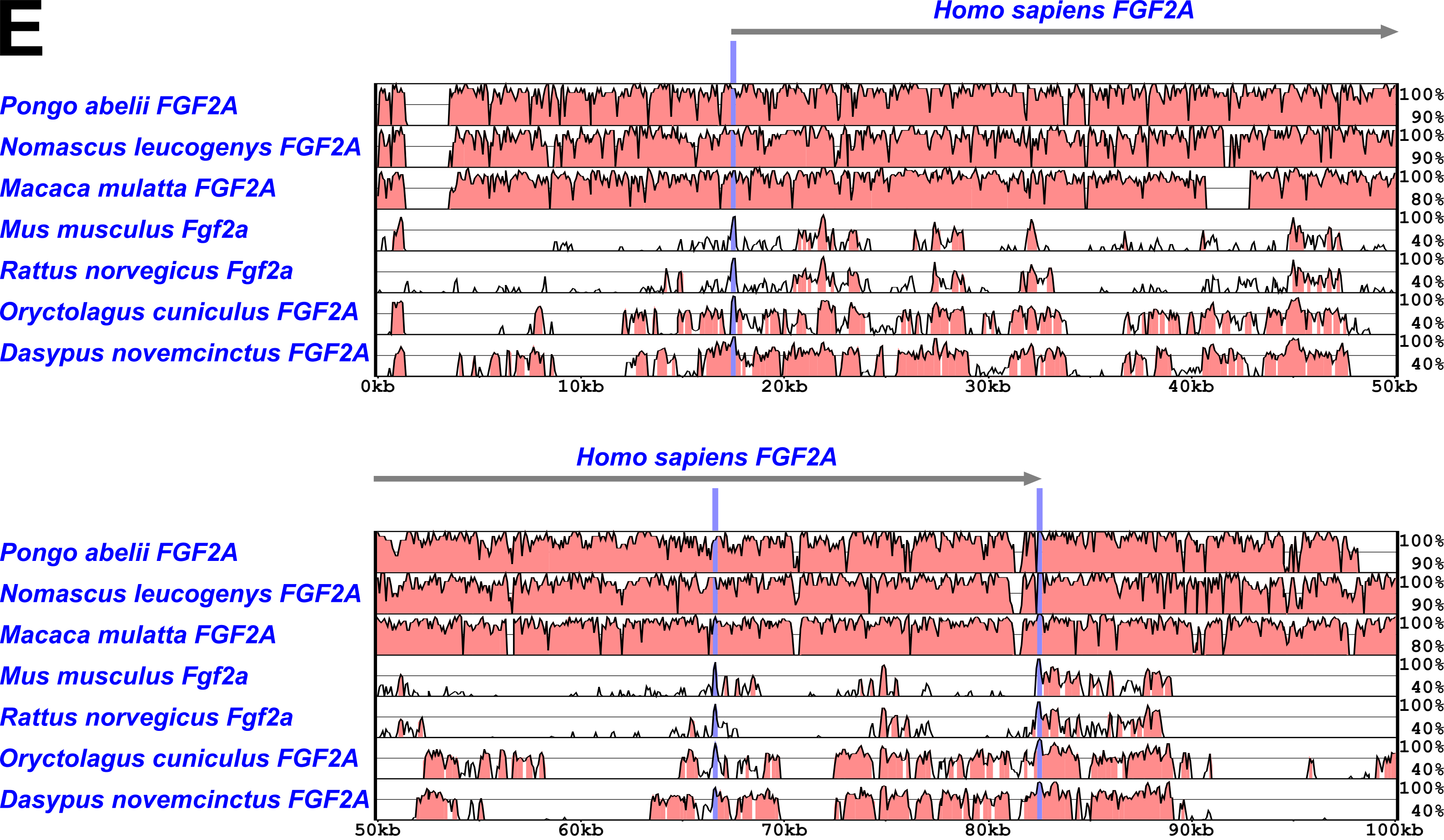
E
Homo sapiens FGF2A
Pongo abelii FGF2A
Nomascus leucogenys FGF2A
Macaca mulatta FGF2A
Mus musculus Fgf2a
Rattus norvegicus Fgf2a
Oryctolagus cuniculus FGF2A
Dasypus novemcinctus FGF2A
100%
90%
80%
40%
0kb
10kb
20kb
30kb
40kb
50kb
Homo sapiens FGF2A
Pongo abelii FGF2A
Nomascus leucogenys FGF2A
Macaca mulatta FGF2A
Mus musculus Fgf2a
Rattus norvegicus Fgf2a
Oryctolagus cuniculus FGF2A
Dasypus novemcinctus FGF2A
50kb
60kb
70kb
80kb
90kb
100kb

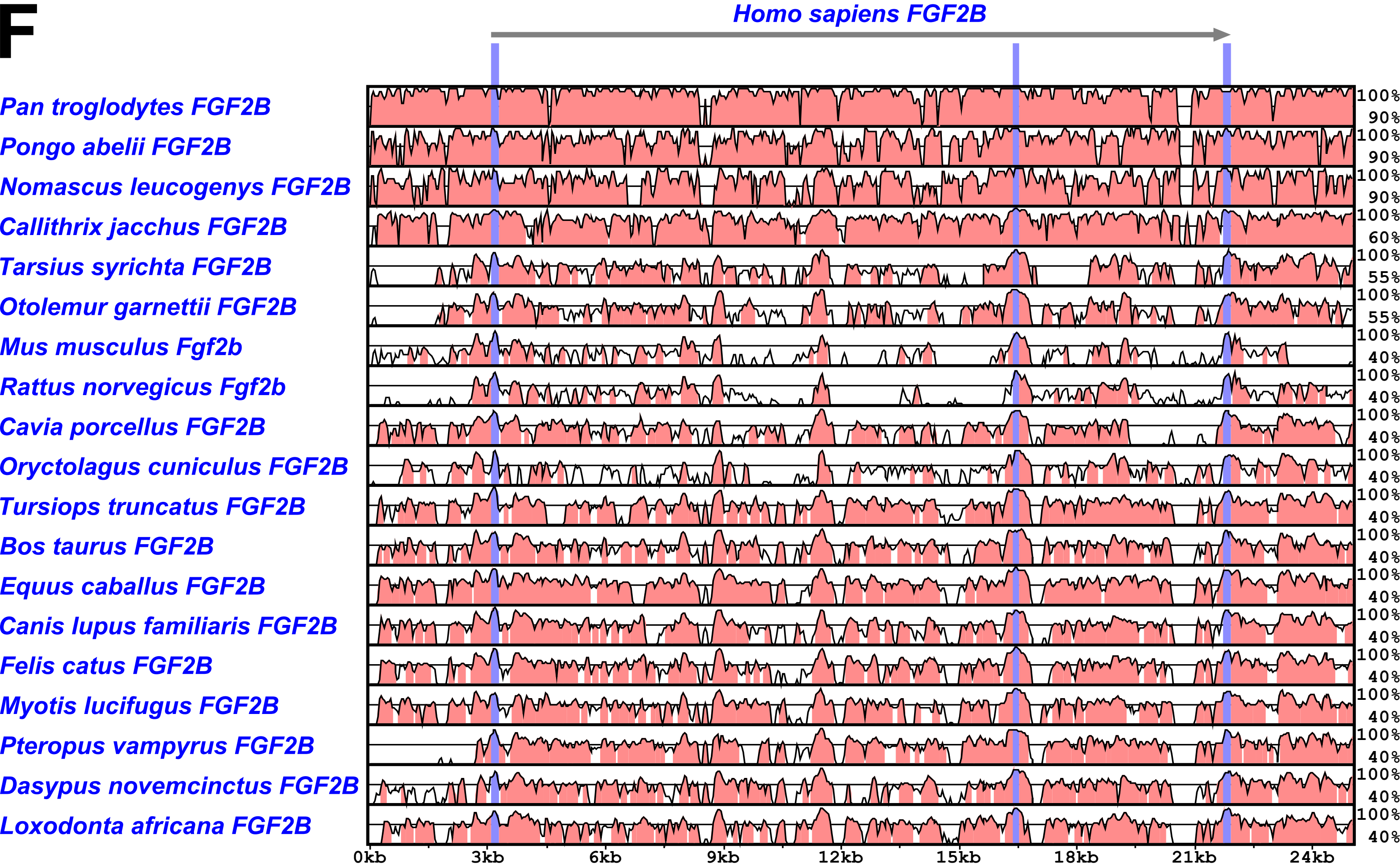
F
Homo sapiens FGF2B
Pan troglodytes FGF2B
Pongo abelii FGF2B
Nomascus leucogenys FGF2B
Callithrix jacchus FGF2B
Tarsius syrichta FGF2B
Otolemur garnettii FGF2B
Mus musculus Fgf2b
Rattus norvegicus Fgf2b
Cavia porcellus FGF2B
Oryctolagus cuniculus FGF2B
Tursiops truncatus FGF2B
Bos taurus FGF2B
Equus caballus FGF2B
Canis lupus familiaris FGF2B
Felis catus FGF2B
Myotis lucifugus FGF2B
Pteropus vampyrus FGF2B
Dasypus novemcinctus FGF2B
Loxodonta africana FGF2B
100%
90%
60%
55%
40%
0kb
3kb
6kb
9kb
12kb
15kb
18kb
21kb
24kb

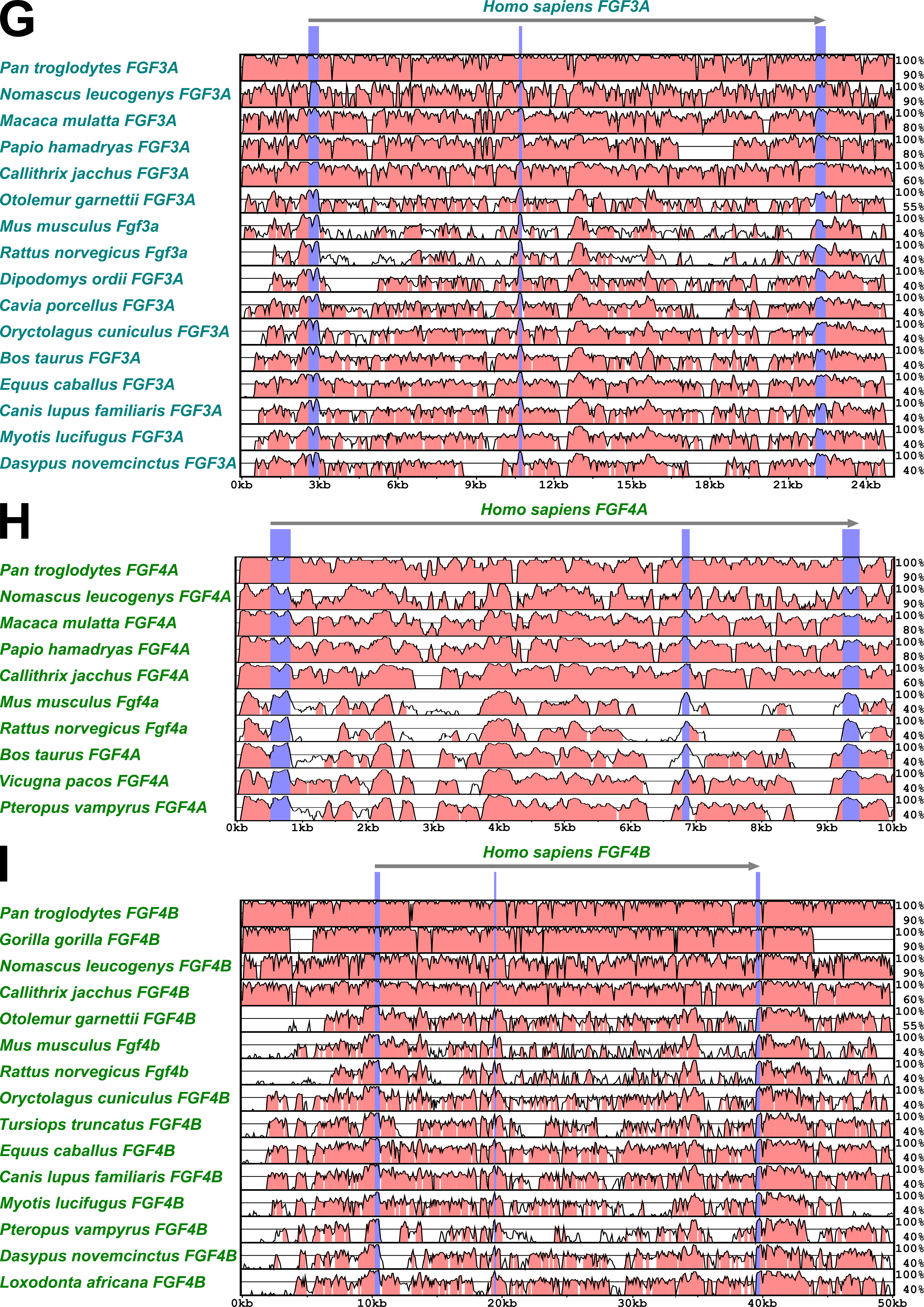

G
Homo sapiens FGF3A
Pan troglodytes FGF3A
Nomascus leucogenys FGF3A
Macaca mulatta FGF3A
Papio hamadryas FGF3A
Callithrix jacchus FGF3A
Otolemur garnettii FGF3A
Mus musculus Fgf3a
Rattus norvegicus Fgf3a
Dipodomys ordii FGF3A
Cavia porcellus FGF3A
Oryctolagus cuniculus FGF3A
Bos taurus FGF3A
Equus caballus FGF3A
Canis lupus familiaris FGF3A
Myotis lucifugus FGF3A
Dasypus novemcinctus FGF3A
0kb 3kb 6kb 9kb 12kb 15kb 18kb 21kb 24kb
H
Homo sapiens FGF4A
Pan troglodytes FGF4A
Nomascus leucogenys FGF4A
Macaca mulatta FGF4A
Papio hamadryas FGF4A
Callithrix jacchus FGF4A
Mus musculus Fgf4a
Rattus norvegicus Fgf4a
Bos taurus FGF4A
Vicugna pacos FGF4A
Pteropus vampyrus FGF4A
0kb 1kb 2kb 3kb 4kb 5kb 6kb 7kb 8kb 9kb 10kb
I
Homo sapiens FGF4B
Pan troglodytes FGF4B
Gorilla gorilla FGF4B
Nomascus leucogenys FGF4B
Callithrix jacchus FGF4B
Otolemur garnettii FGF4B
Mus musculus Fgf4b
Rattus norvegicus Fgf4b
Oryctolagus cuniculus FGF4B
Tursiops truncatus FGF4B
Equus caballus FGF4B
Canis lupus familiaris FGF4B
Myotis lucifugus FGF4B
Pteropus vampyrus FGF4B
Dasypus novemcinctus FGF4B
Loxodonta africana FGF4B
0kb 10kb 20kb 30kb 40kb 50kb
100% 90% 80% 60% 55% 40%

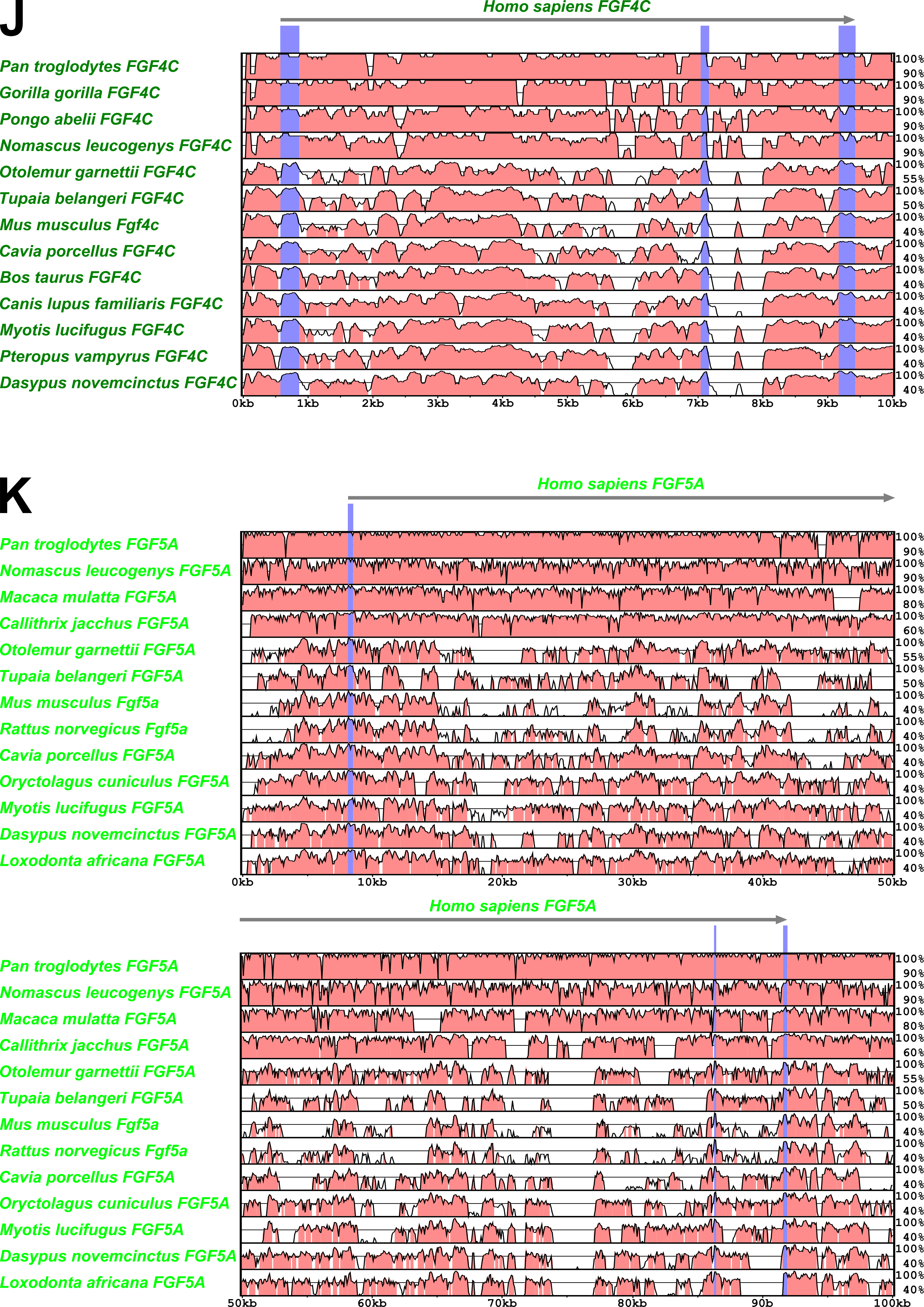
J
Homo sapiens FGF4C
Pan troglodytes FGF4C
Gorilla gorilla FGF4C
Pongo abelii FGF4C
Nomascus leucogenys FGF4C
Otolemur garnettii FGF4C
Tupaia belangeri FGF4C
Mus musculus Fgf4c
Cavia porcellus FGF4C
Bos taurus FGF4C
Canis lupus familiaris FGF4C
Myotis lucifugus FGF4C
Pteropus vampyrus FGF4C
Dasypus novemcinctus FGF4C
100%
90%
55%
50%
40%
0kb
1kb
2kb
3kb
4kb
5kb
6kb
7kb
8kb
9kb
10kb
K
Homo sapiens FGF5A
Pan troglodytes FGF5A
Nomascus leucogenys FGF5A
Macaca mulatta FGF5A
Callithrix jacchus FGF5A
Otolemur garnettii FGF5A
Tupaia belangeri FGF5A
Mus musculus Fgf5a
Rattus norvegicus Fgf5a
Cavia porcellus FGF5A
Oryctolagus cuniculus FGF5A
Myotis lucifugus FGF5A
Dasypus novemcinctus FGF5A
Loxodonta africana FGF5A
100%
90%
80%
60%
55%
50%
40%
0kb
10kb
20kb
30kb
40kb
50kb
60kb
70kb
80kb
90b
100kb

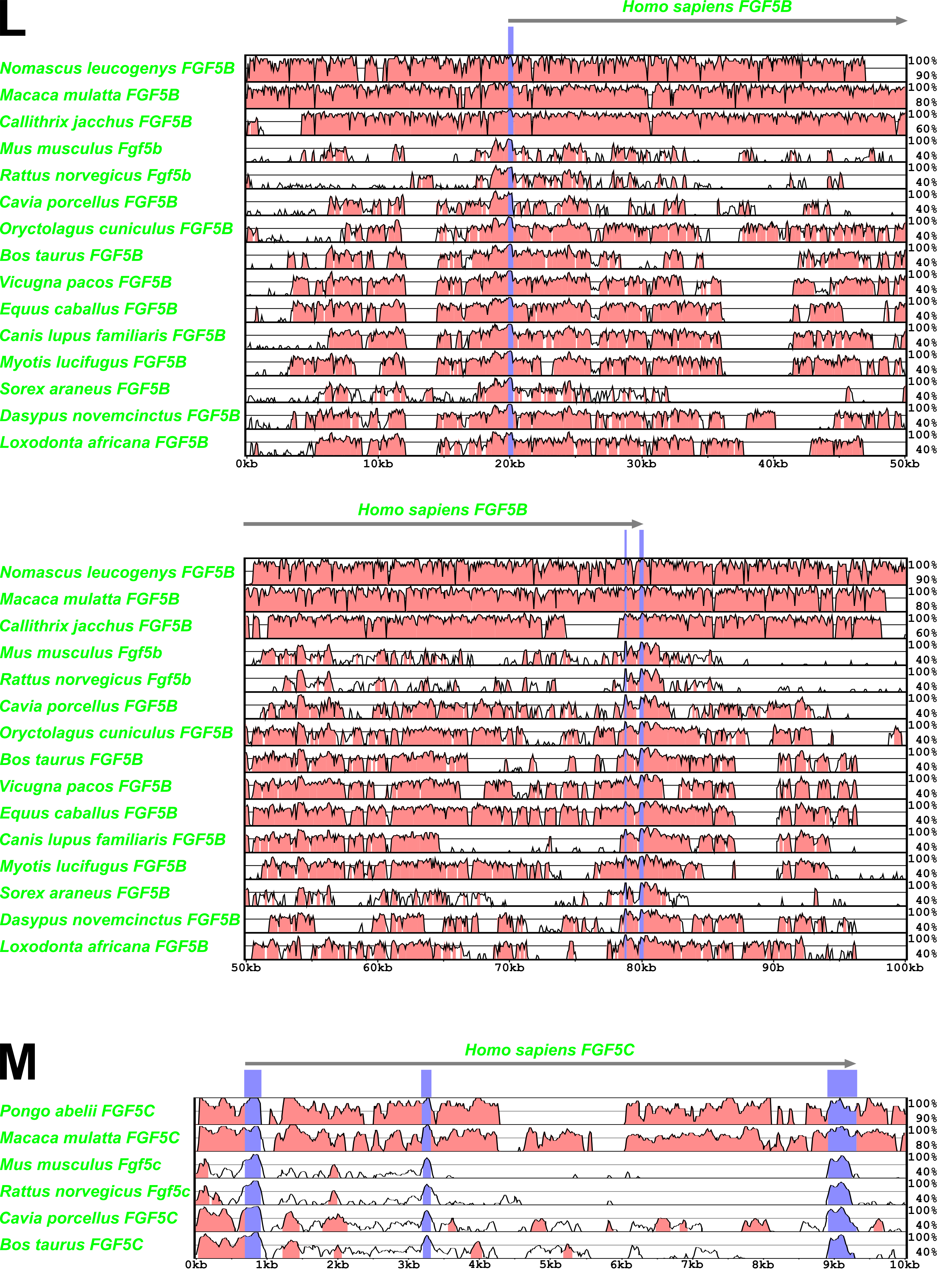

L
Homo sapiens FGF5B
Nomascus leucogenys FGF5B
Macaca mulatta FGF5B
Callithrix jacchus FGF5B
Mus musculus Fgf5b
Rattus norvegicus Fgf5b
Cavia porcellus FGF5B
Oryctolagus cuniculus FGF5B
Bos taurus FGF5B
Vicugna pacos FGF5B
Equus caballus FGF5B
Canis lupus familiaris FGF5B
Myotis lucifugus FGF5B
Sorex araneus FGF5B
Dasypus novemcinctus FGF5B
Loxodonta africana FGF5B
100%
90%
80%
60%
40%
0kb
10kb
20kb
30kb
40kb
50kb
60kb
70kb
80kb
90b
100kb
M
Homo sapiens FGF5C
Pongo abelii FGF5C
Macaca mulatta FGF5C
Mus musculus Fgf5c
Rattus norvegicus Fgf5c
Cavia porcellus FGF5C
Bos taurus FGF5C
1kb
2kb
3kb
4kb
5kb
6kb
7kb
8kb
9kb
10kb

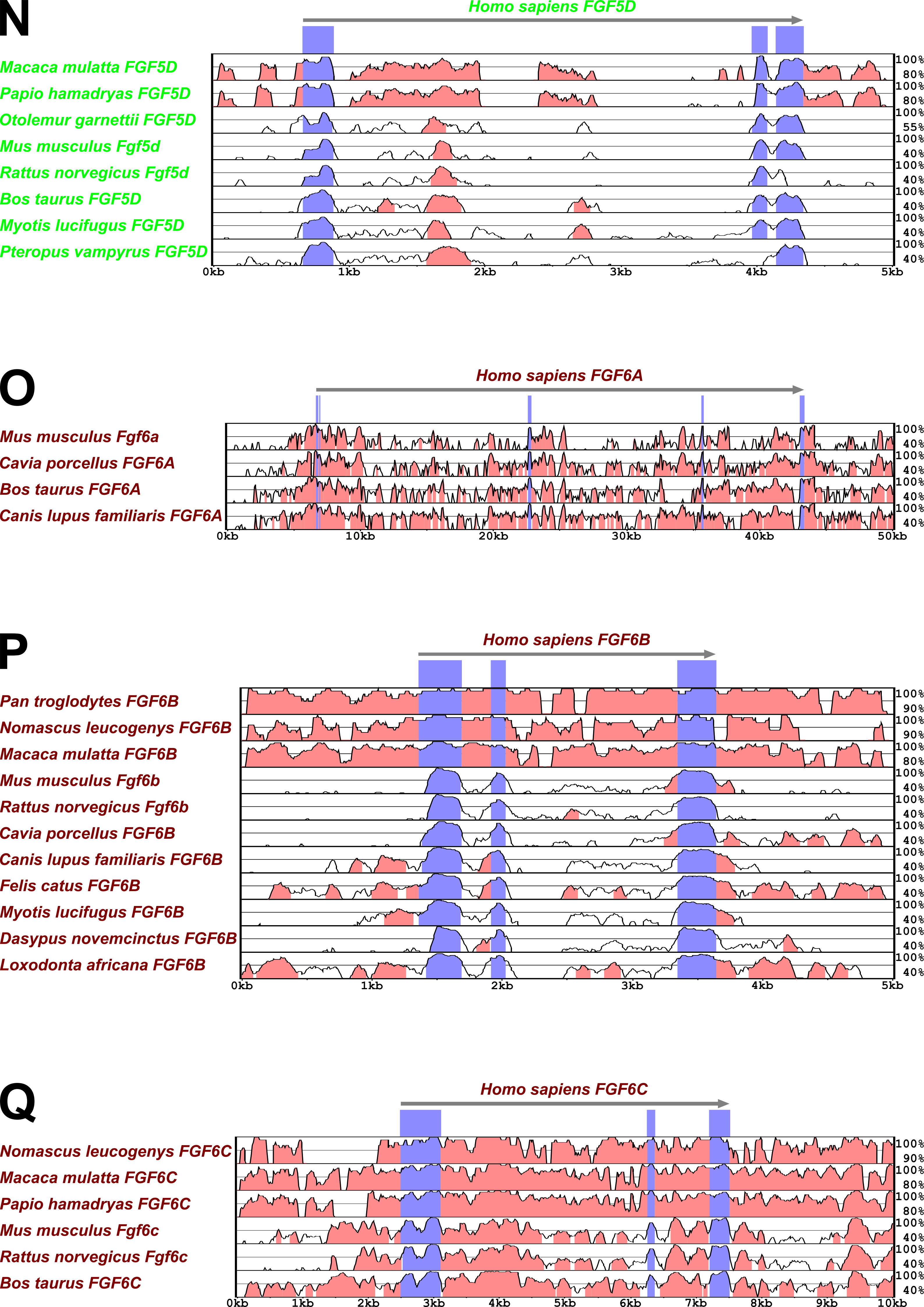
N
Homo sapiens FGF5D
Macaca mulatta FGF5D
Papio hamadryas FGF5D
Otolemur garnettii FGF5D
Mus musculus Fgf5d
Rattus norvegicus Fgf5d
Bos taurus FGF5D
Myotis lucifugus FGF5D
Pteropus vampyrus FGF5D
100%
80%
55%
40%
0kb
1kb
2kb
3kb
4kb
5kb
O
Homo sapiens FGF6A
Mus musculus Fgf6a
Cavia porcellus FGF6A
Bos taurus FGF6A
Canis lupus familiaris FGF6A
0kb
10kb
20kb
30kb
40kb
50kb
P
Homo sapiens FGF6B
Pan troglodytes FGF6B
Nomascus leucogenys FGF6B
Macaca mulatta FGF6B
Mus musculus Fgf6b
Rattus norvegicus Fgf6b
Cavia porcellus FGF6B
Canis lupus familiaris FGF6B
Felis catus FGF6B
Myotis lucifugus FGF6B
Dasypus novemcinctus FGF6B
Loxodonta africana FGF6B
90%
Q
Homo sapiens FGF6C
Nomascus leucogenys FGF6C
Macaca mulatta FGF6C
Papio hamadryas FGF6C
Mus musculus Fgf6c
Rattus norvegicus Fgf6c
Bos taurus FGF6C
6kb
7kb
8kb
9kb
10kb

R

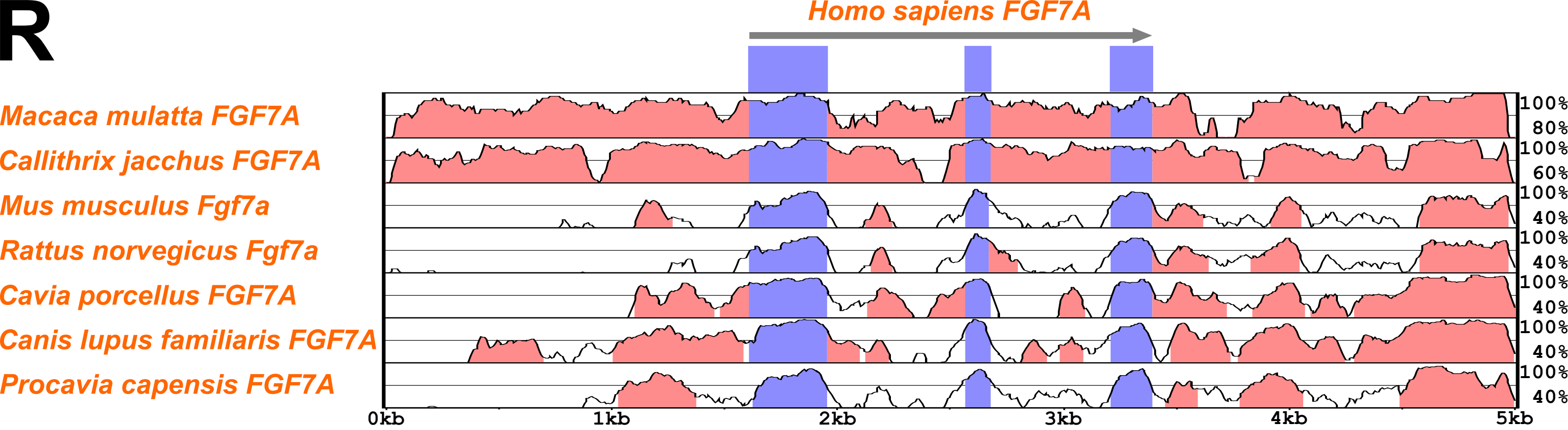
Homo sapiens FGF7A
Macaca mulatta FGF7A
Callithrix jacchus FGF7A
Mus musculus Fgf7a
Rattus norvegicus Fgf7a
Cavia porcellus FGF7A
Canis lupus familiaris FGF7A
Procavia capensis FGF7A
100%
80%
60%
40%
0kb
1kb
2kb
3kb
4kb
5kb

S

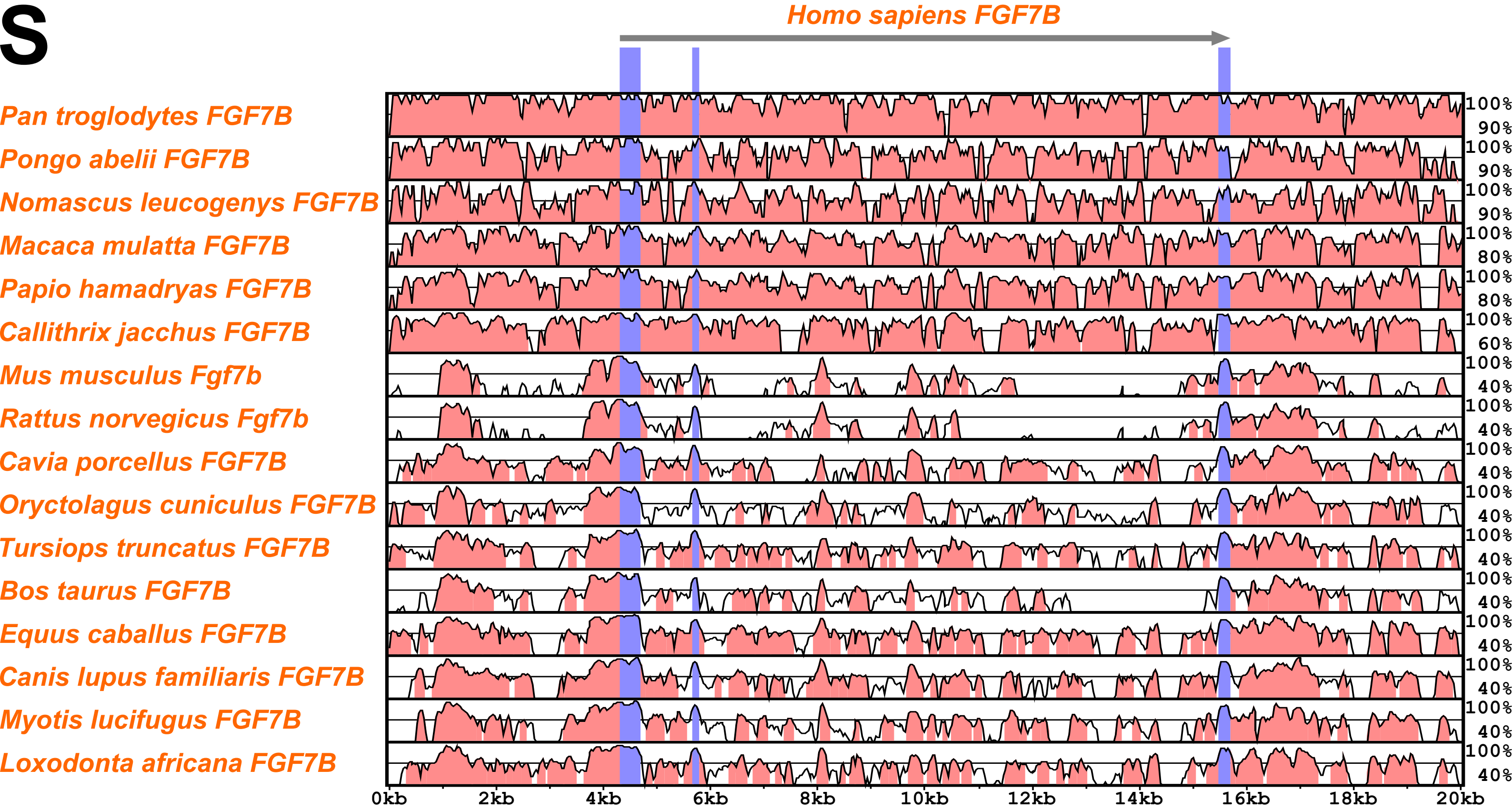
Homo sapiens FGF7B
Pan troglodytes FGF7B
Pongo abelii FGF7B
Nomascus leucogenys FGF7B
Macaca mulatta FGF7B
Papio hamadryas FGF7B
Callithrix jacchus FGF7B
Mus musculus Fgf7b
Rattus norvegicus Fgf7b
Cavia porcellus FGF7B
Oryctolagus cuniculus FGF7B
Tursiops truncatus FGF7B
Bos taurus FGF7B
Equus caballus FGF7B
Canis lupus familiaris FGF7B
Myotis lucifugus FGF7B
Loxodonta africana FGF7B
100%
90%
80%
60%
40%
0kb
2kb
4kb
6kb
8kb
10kb
12kb
14kb
16kb
18kb
20kb

T

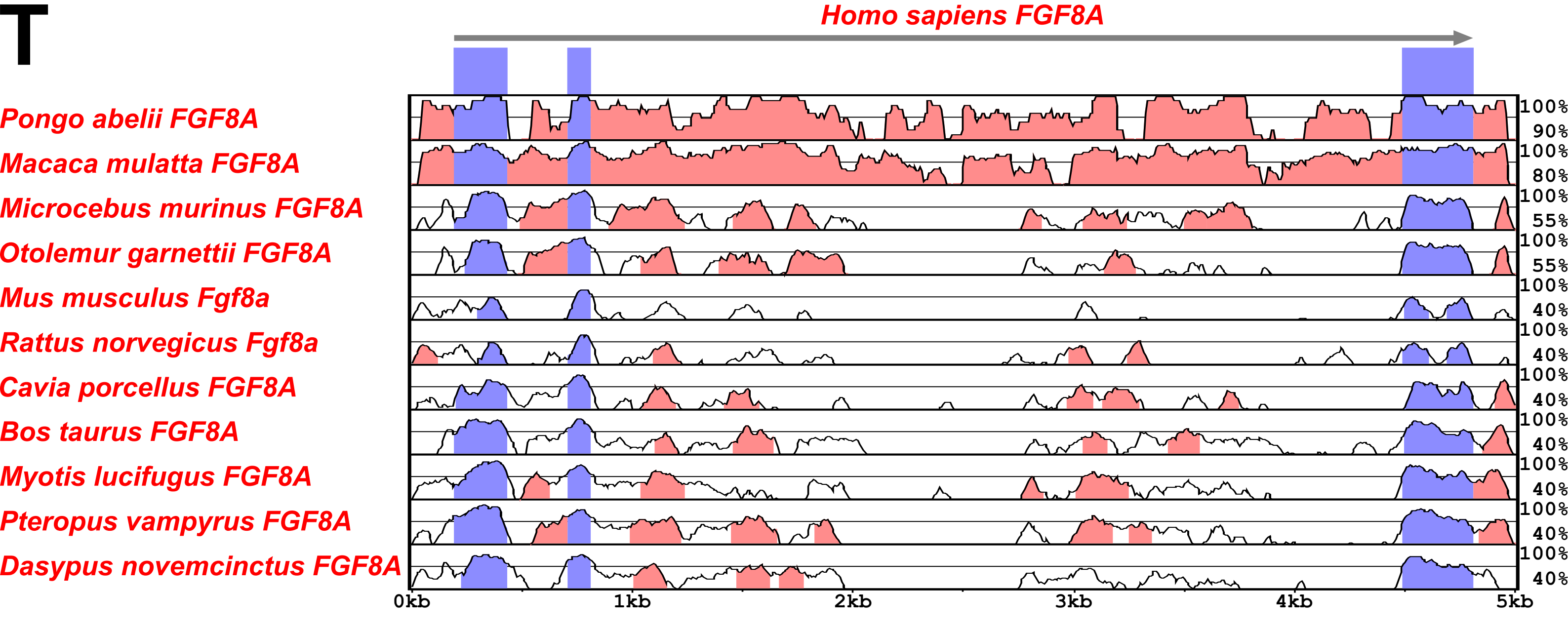
Homo sapiens FGF8A
Pongo abelii FGF8A
Macaca mulatta FGF8A
Microcebus murinus FGF8A
Otolemur garnettii FGF8A
Mus musculus Fgf8a
Rattus norvegicus Fgf8a
Cavia porcellus FGF8A
Bos taurus FGF8A
Myotis lucifugus FGF8A
Pteropus vampyrus FGF8A
Dasypus novemcinctus FGF8A
100%
90%
80%
55%
40%
0kb
1kb
2kb
3kb
4kb
5kb

U

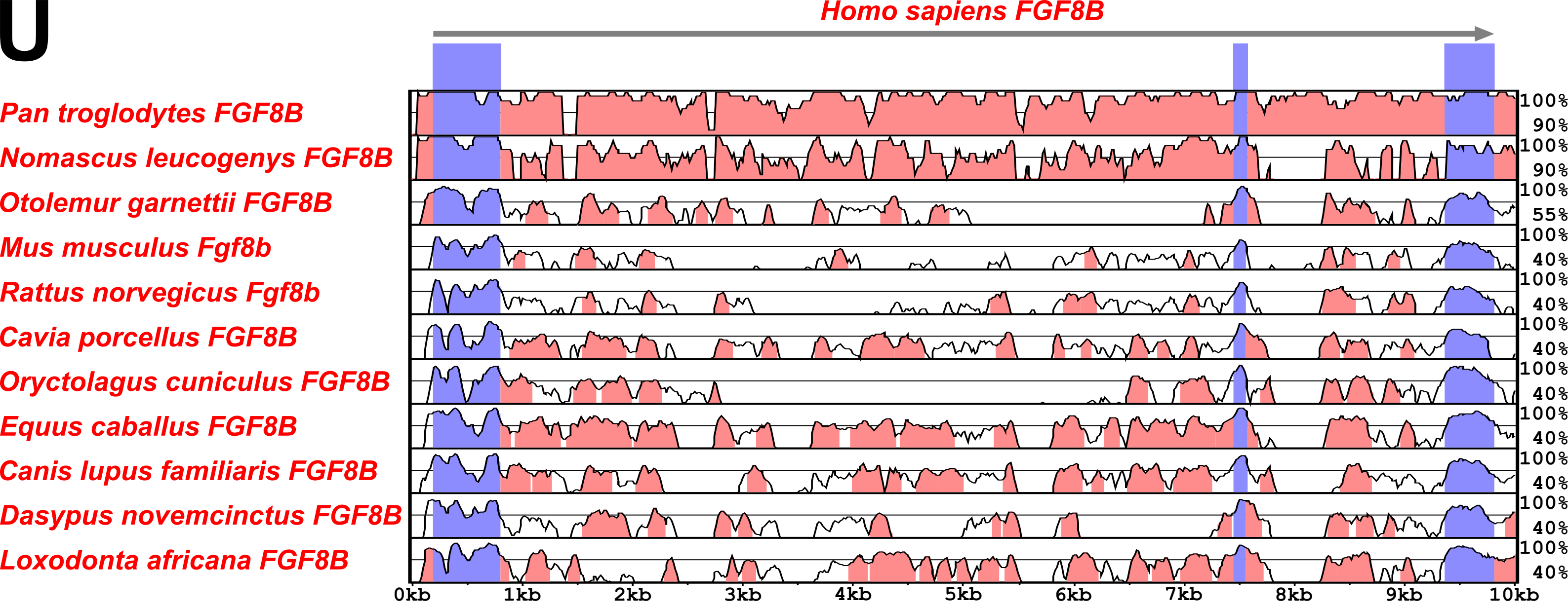
Homo sapiens FGF8B
Pan troglodytes FGF8B
Nomascus leucogenys FGF8B
Otolemur garnettii FGF8B
Mus musculus Fgf8b
Rattus norvegicus Fgf8b
Cavia porcellus FGF8B
Oryctolagus cuniculus FGF8B
Equus caballus FGF8B
Canis lupus familiaris FGF8B
Dasypus novemcinctus FGF8B
Loxodonta africana FGF8B
100%
90%
55%
40%
0kb
1kb
2kb
3kb
4kb
5kb
6kb
7kb
8kb
9kb
10kb

V

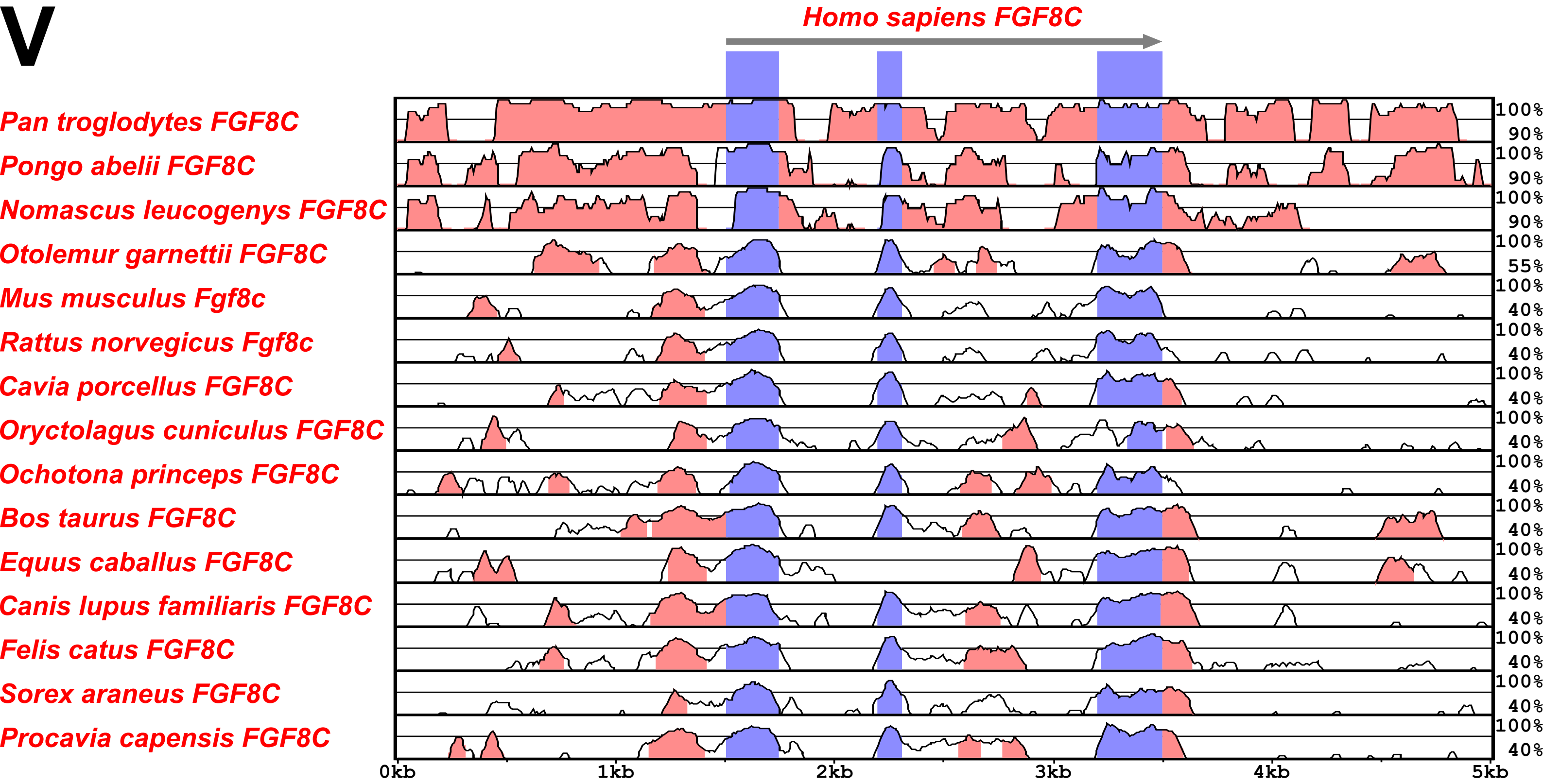
Homo sapiens FGF8C
Pan troglodytes FGF8C
Pongo abelii FGF8C
Nomascus leucogenys FGF8C
Otolemur garnettii FGF8C
Mus musculus Fgf8c
Rattus norvegicus Fgf8c
Cavia porcellus FGF8C
Oryctolagus cuniculus FGF8C
Ochotona princeps FGF8C
Bos taurus FGF8C
Equus caballus FGF8C
Canis lupus familiaris FGF8C
Felis catus FGF8C
Sorex araneus FGF8C
Procavia capensis FGF8C
100%
90%
55%
40%
0kb
1kb
2kb
3kb
4kb
5kb
