# Supplementary material for: Comparative genomic analysis of eutherian fibroblast growth factor genes
Source: BMC Genomics. 2020 Aug 5;21:542. doi: 10.1186/s12864-020-06958-4 (PMC7430813; doi:10.1186/s12864-020-06958-4)
Supplement: Supplementary file 3 — Additional file 3. Pairwise nucleotide sequence identity patterns of eutherian fibroblast growth factor genes. [file 12864_2020_6958_MOESM3_ESM.pdf]

Additional File 3: Pairwise nucleotide sequence identity patterns of eutherian fibroblast growth factor genes.

	$\bar{a}$	a_{max}	a_{min}	$\bar{a}_{ad}$	$\bar{a}$	a_{max}	a_{min}	$\bar{a}_{ad}$	$\bar{a}$	a_{max}	a_{min}	$\bar{a}_{ad}$	$\bar{a}$	a_{max}	a_{min}	$\bar{a}_{ad}$	$\bar{a}$	a_{max}	a_{min}	$\bar{a}_{ad}$
Cluster	***FGF1A***				***FGF1B***				***FGF1C***				***FGF1D***				***FGF2A***			
FGF1A	**0,954**	**1**	**0,931**	**0,011**																
FGF1B	0,678	0,685	0,669	0,003	**0,965**	**0,997**	**0,939**	**0,014**												
FGF1C	0,613	0,626	0,6	0,005	0,635	0,648	0,622	0,005	**0,948**	**1**	**0,921**	**0,013**								
FGF1D	0,537	0,55	0,527	0,004	0,52	0,533	0,508	0,004	0,523	0,538	0,506	0,006	**0,942**	**1**	**0,921**	**0,009**				
FGF2A	0,214	0,22	0,207	0,002	0,217	0,222	0,211	0,002	0,223	0,229	0,215	0,003	0,217	0,227	0,206	0,004	**0,927**	**0,989**	**0,871**	**0,034**
FGF2B	0,248	0,261	0,235	0,004	0,237	0,251	0,227	0,004	0,247	0,263	0,238	0,004	0,24	0,259	0,224	0,005	0,529	0,556	0,504	0,009
FGF3A	0,289	0,304	0,264	0,006	0,302	0,308	0,29	0,003	0,299	0,309	0,284	0,006	0,283	0,297	0,269	0,004	0,236	0,248	0,22	0,005
FGF4A	0,315	0,327	0,303	0,003	0,315	0,327	0,307	0,004	0,3	0,315	0,288	0,004	0,312	0,322	0,299	0,003	0,279	0,292	0,267	0,004
FGF4B	0,307	0,315	0,299	0,002	0,312	0,318	0,307	0,002	0,307	0,32	0,298	0,003	0,305	0,318	0,292	0,004	0,264	0,275	0,252	0,003
FGF4C	0,293	0,301	0,281	0,004	0,307	0,314	0,302	0,002	0,294	0,302	0,284	0,003	0,307	0,316	0,295	0,004	0,274	0,288	0,262	0,004
FGF5A	0,255	0,27	0,214	0,007	0,251	0,26	0,215	0,006	0,26	0,274	0,216	0,007	0,254	0,269	0,214	0,007	0,237	0,251	0,188	0,009
FGF5B	0,269	0,282	0,259	0,004	0,265	0,278	0,258	0,003	0,273	0,284	0,26	0,004	0,243	0,26	0,228	0,004	0,266	0,28	0,252	0,005
FGF5C	0,232	0,242	0,225	0,003	0,24	0,254	0,233	0,003	0,259	0,276	0,246	0,005	0,244	0,261	0,232	0,005	0,246	0,256	0,235	0,004
FGF5D	0,24	0,256	0,191	0,01	0,233	0,25	0,185	0,011	0,235	0,255	0,193	0,01	0,27	0,296	0,212	0,015	0,286	0,321	0,218	0,019
FGF6A	0,227	0,232	0,219	0,003	0,238	0,249	0,23	0,004	0,257	0,277	0,243	0,005	0,233	0,241	0,218	0,005	0,197	0,213	0,186	0,006
FGF6B	0,225	0,269	0,178	0,017	0,228	0,275	0,18	0,018	0,24	0,292	0,189	0,017	0,232	0,272	0,179	0,02	0,165	0,229	0,122	0,017
FGF6C	0,214	0,232	0,153	0,02	0,216	0,239	0,153	0,02	0,251	0,28	0,181	0,023	0,226	0,25	0,16	0,022	0,192	0,223	0,128	0,021
FGF7A	0,272	0,282	0,26	0,004	0,255	0,264	0,238	0,004	0,253	0,265	0,244	0,003	0,29	0,308	0,269	0,008	0,278	0,297	0,269	0,006
FGF7B	0,254	0,268	0,242	0,004	0,244	0,256	0,232	0,004	0,255	0,268	0,243	0,005	0,262	0,28	0,241	0,006	0,285	0,298	0,263	0,005
FGF8A	0,181	0,201	0,145	0,01	0,174	0,192	0,138	0,007	0,187	0,205	0,154	0,01	0,181	0,197	0,151	0,009	0,246	0,272	0,203	0,014
FGF8B	0,162	0,192	0,131	0,02	0,163	0,195	0,134	0,018	0,176	0,218	0,143	0,022	0,146	0,182	0,116	0,019	0,18	0,229	0,139	0,026
FGF8C	0,169	0,18	0,156	0,005	0,172	0,19	0,155	0,006	0,182	0,193	0,169	0,005	0,176	0,19	0,166	0,004	0,225	0,24	0,209	0,007

Cluster	*FGF2B*				*FGF3A*				*FGF4A*				*FGF4B*				*FGF4C*			
FGF2B	**0,913**	**0,995**	**0,852**	**0,026**																
FGF3A	0,233	0,247	0,219	0,004	**0,886**	**0,997**	**0,811**	**0,031**												
FGF4A	0,304	0,324	0,29	0,006	0,306	0,316	0,293	0,005	**0,93**	**1**	**0,87**	**0,029**								
FGF4B	0,297	0,314	0,276	0,005	0,292	0,302	0,279	0,004	0,653	0,672	0,632	0,007	**0,95**	**0,998**	**0,902**	**0,017**				
FGF4C	0,299	0,311	0,286	0,004	0,292	0,303	0,274	0,004	0,629	0,652	0,612	0,006	0,632	0,654	0,616	0,006	**0,94**	**0,998**	**0,891**	**0,015**
FGF5A	0,248	0,268	0,203	0,009	0,272	0,303	0,229	0,007	0,322	0,344	0,271	0,009	0,33	0,353	0,269	0,013	0,331	0,349	0,27	0,01
FGF5B	0,273	0,288	0,257	0,005	0,263	0,272	0,251	0,003	0,36	0,379	0,34	0,006	0,354	0,37	0,337	0,005	0,347	0,362	0,328	0,006
FGF5C	0,238	0,261	0,227	0,005	0,275	0,292	0,257	0,009	0,308	0,327	0,297	0,005	0,299	0,315	0,29	0,004	0,319	0,332	0,304	0,006
FGF5D	0,308	0,347	0,223	0,021	0,249	0,271	0,192	0,011	0,341	0,364	0,269	0,016	0,316	0,34	0,236	0,017	0,349	0,37	0,275	0,015
FGF6A	0,222	0,241	0,206	0,006	0,203	0,215	0,195	0,004	0,302	0,312	0,284	0,005	0,294	0,308	0,284	0,004	0,296	0,307	0,285	0,005
FGF6B	0,193	0,27	0,142	0,021	0,218	0,264	0,173	0,017	0,254	0,293	0,196	0,021	0,259	0,302	0,2	0,022	0,259	0,309	0,201	0,023
FGF6C	0,211	0,24	0,142	0,022	0,22	0,246	0,155	0,018	0,274	0,3	0,193	0,028	0,277	0,313	0,191	0,03	0,285	0,312	0,197	0,028
FGF7A	0,271	0,292	0,259	0,006	0,297	0,315	0,281	0,005	0,321	0,349	0,304	0,008	0,316	0,33	0,299	0,006	0,319	0,337	0,291	0,01
FGF7B	0,272	0,294	0,254	0,006	0,315	0,329	0,299	0,005	0,331	0,342	0,317	0,004	0,316	0,333	0,296	0,006	0,316	0,329	0,301	0,005
FGF8A	0,241	0,27	0,191	0,01	0,199	0,221	0,161	0,011	0,221	0,243	0,175	0,01	0,206	0,224	0,162	0,008	0,219	0,24	0,168	0,012
FGF8B	0,19	0,245	0,142	0,026	0,204	0,247	0,161	0,024	0,182	0,225	0,147	0,021	0,166	0,203	0,133	0,021	0,183	0,221	0,15	0,021
FGF8C	0,241	0,264	0,218	0,008	0,219	0,237	0,196	0,007	0,237	0,253	0,216	0,007	0,215	0,226	0,2	0,004	0,228	0,247	0,208	0,006

Cluster	*FGF5A*				*FGF5B*				*FGF5C*				*FGF5D*				*FGF6A*			
FGF5A	**0,887**	**0,998**	**0,676**	**0,055**																
FGF5B	0,456	0,482	0,38	0,015	**0,93**	**0,994**	**0,864**	**0,028**												
FGF5C	0,288	0,307	0,237	0,009	0,285	0,307	0,271	0,006	**0,818**	**0,975**	**0,745**	**0,054**								
FGF5D	0,375	0,417	0,296	0,024	0,347	0,375	0,271	0,016	0,344	0,393	0,272	0,021	**0,768**	**0,986**	**0,522**	**0,091**				
FGF6A	0,228	0,239	0,193	0,007	0,24	0,252	0,229	0,004	0,244	0,259	0,226	0,007	0,248	0,268	0,185	0,013	**0,934**	**0,964**	**0,902**	**0,017**
FGF6B	0,217	0,259	0,171	0,018	0,214	0,256	0,164	0,019	0,247	0,306	0,188	0,023	0,243	0,311	0,18	0,025	0,433	0,515	0,333	0,042
FGF6C	0,231	0,256	0,162	0,021	0,223	0,251	0,156	0,022	0,274	0,308	0,187	0,026	0,241	0,279	0,162	0,028	0,46	0,507	0,312	0,05
FGF7A	0,291	0,308	0,244	0,008	0,288	0,316	0,266	0,009	0,26	0,284	0,243	0,01	0,319	0,357	0,241	0,021	0,255	0,264	0,241	0,005
FGF7B	0,294	0,309	0,245	0,007	0,323	0,348	0,302	0,007	0,253	0,271	0,241	0,006	0,295	0,321	0,23	0,015	0,238	0,253	0,216	0,007
FGF8A	0,198	0,214	0,154	0,01	0,191	0,211	0,145	0,008	0,217	0,242	0,174	0,015	0,24	0,271	0,18	0,018	0,169	0,189	0,133	0,009
FGF8B	0,16	0,195	0,131	0,017	0,162	0,2	0,134	0,018	0,212	0,27	0,16	0,027	0,197	0,258	0,15	0,028	0,164	0,202	0,129	0,021
FGF8C	0,218	0,239	0,178	0,009	0,206	0,228	0,191	0,005	0,22	0,238	0,193	0,008	0,241	0,275	0,191	0,012	0,173	0,192	0,159	0,005

Cluster	*FGF6B*				*FGF6C*				*FGF7A*				*FGF7B*				*FGF8A*			
FGF6B	**0,748**	**0,993**	**0,485**	**0,077**																
FGF6C	0,518	0,645	0,362	0,058	**0,821**	**0,996**	**0,579**	**0,133**												
FGF7A	0,244	0,298	0,189	0,022	0,249	0,276	0,173	0,026	**0,855**	**0,972**	**0,75**	**0,057**								
FGF7B	0,219	0,265	0,17	0,018	0,225	0,248	0,157	0,021	0,581	0,625	0,529	0,017	**0,916**	**0,993**	**0,824**	**0,028**				
FGF8A	0,166	0,216	0,125	0,015	0,172	0,203	0,115	0,019	0,243	0,266	0,19	0,01	0,216	0,238	0,164	0,011	**0,722**	**0,987**	**0,482**	**0,095**
FGF8B	0,189	0,24	0,148	0,018	0,182	0,228	0,142	0,024	0,182	0,226	0,14	0,022	0,174	0,212	0,139	0,022	0,286	0,357	0,201	0,038
FGF8C	0,165	0,213	0,128	0,015	0,185	0,213	0,124	0,017	0,247	0,261	0,231	0,007	0,239	0,256	0,212	0,007	0,387	0,426	0,299	0,022

Cluster	*FGF8B*				*FGF8C*			
FGF8B	**0,647**	**0,993**	**0,483**	**0,083**				
FGF8C	0,276	0,342	0,211	0,039	**0,788**	**0,99**	**0,675**	**0,049**

$\bar{a}$, average pairwise identity; $\bar{a}_{ad}$, average absolute deviation for $\bar{a}$; a_{max}, largest pairwise identity; a_{min}, smallest pairwise identity; bold, calculation within major cluster.
