# Supplementary material for: Comparative genomic analysis of eutherian fibroblast growth factor genes
Source: BMC Genomics. 2020 Aug 5;21:542. doi: 10.1186/s12864-020-06958-4 (PMC7430813; doi:10.1186/s12864-020-06958-4)
Supplement: Supplementary file 4 — Additional file 4. Protein amino acid sequence alignments of eutherian fibroblast growth factors. The amino acid positions were labelled using white letters on black background (100% sequence identity level), white letters on dark grey background (≥ 75% sequence identity level) or black letters on grey background (≥50% sequence identity level). The 19 invariant amino acid sites were shown using white letters on violet backgrounds and 3 forward amino acid sites were shown using white letters on red backgrounds in reference human FGF1A protein primary structure (top). The stop codons were indicated by &s. [file 12864_2020_6958_MOESM4_ESM.pdf]

```
Homo sapiens FGF1A               - ----------------------------------------------------------------- -
                                            10        20        30        40        50        60
Homo sapiens FGF1A               - ----------------------------------------------------------------- -
Pan troglodytes FGF1A            - ----------------------------------------------------------------- -
Nomascus leucogenys FGF1A        - ----------------------------------------------------------------- -
Callithrix jacchus FGF1A         - ----------------------------------------------------------------- -
Mus musculus Fgf1a               - ----------------------------------------------------------------- -
Rattus norvegicus Fgf1a          - ----------------------------------------------------------------- -
Cavia porcellus FGF1A            - ----------------------------------------------------------------- -
Oryctolagus cuniculus FGF1A      - ----------------------------------------------------------------- -
Equus caballus FGF1A             - ----------------------------------------------------------------- -
Canis lupus familiaris FGF1A     - ----------------------------------------------------------------- -
Dasypus novemcinctus FGF1A       - ----------------------------------------------------------------- -
Homo sapiens FGF1B               - ----------------------------------------------------------------- -
Pan troglodytes FGF1B            - ----------------------------------------------------------------- -
Pongo abelii FGF1B               - ----------------------------------------------------------------- -
Macaca mulatta FGF1B             - ----------------------------------------------------------------- -
Callithrix jacchus FGF1B         - ----------------------------------------------------------------- -
Mus musculus Fgf1b               - ----------------------------------------------------------------- -
Oryctolagus cuniculus FGF1B      - ----------------------------------------------------------------- -
Equus caballus FGF1B             - ----------------------------------------------------------------- -
Dasypus novemcinctus FGF1B       - ----------------------------------------------------------------- -
Homo sapiens FGF1C               - ----------------------------------------------------------------- -
Pan troglodytes FGF1C            - ----------------------------------------------------------------- -
Pongo abelii FGF1C               - ----------------------------------------------------------------- -
Macaca mulatta FGF1C             - ----------------------------------------------------------------- -
Mus musculus Fgf1c               - ----------------------------------------------------------------- -
Rattus norvegicus Fgf1c          - ----------------------------------------------------------------- -
Cavia porcellus FGF1C            - ----------------------------------------------------------------- -
Bos taurus FGF1C                 - ----------------------------------------------------------------- -
Myotis lucifugus FGF1C           - ----------------------------------------------------------------- -
Dasypus novemcinctus FGF1C       - ----------------------------------------------------------------- -
Loxodonta africana FGF1C         - ----------------------------------------------------------------- -
Homo sapiens FGF1D               - ----------------------------------------------------------------- -
Pan troglodytes FGF1D            - ----------------------------------------------------------------- -
Nomascus leucogenys FGF1D        - ----------------------------------------------------------------- -
Macaca mulatta FGF1D             - ----------------------------------------------------------------- -
Otolemur garnettii FGF1D         - ----------------------------------------------------------------- -
Mus musculus Fgf1d               - ----------------------------------------------------------------- -
Rattus norvegicus Fgf1d          - ----------------------------------------------------------------- -
Cavia porcellus FGF1D            - ----------------------------------------------------------------- -
Oryctolagus cuniculus FGF1D      - ----------------------------------------------------------------- -
Bos taurus FGF1D                 - ----------------------------------------------------------------- -
Equus caballus FGF1D             - ----------------------------------------------------------------- -
Canis lupus familiaris FGF1D     - ----------------------------------------------------------------- -
Pteropus vampyrus FGF1D          - ----------------------------------------------------------------- -
Dasypus novemcinctus FGF1D       - ----------------------------------------------------------------- -
Loxodonta africana FGF1D         - ----------------------------------------------------------------- -
Homo sapiens FGF2A               - ----------------------------------------------------------------- -
Pongo abelii FGF2A               - ----------------------------------------------------------------- -
Nomascus leucogenys FGF2A        - ----------------------------------------------------------------- -
Macaca mulatta FGF2A             - ----------------------------------------------------------------- -
Mus musculus Fgf2a               - ----------------------------------------------------------------- -
Rattus norvegicus Fgf2a          - ----------------------------------------------------------------- -
Oryctolagus cuniculus FGF2A      - ----------------------------------------------------------------- -
Dasypus novemcinctus FGF2A       - ----------------------------------------------------------------- -
Homo sapiens FGF2B               - ----------------------------------------------------------------- -
Pan troglodytes FGF2B            - ----------------------------------------------------------------- -
Pongo abelii FGF2B               - ----------------------------------------------------------------- -
Nomascus leucogenys FGF2B        - ----------------------------------------------------------------- -
Callithrix jacchus FGF2B         - ----------------------------------------------------------------- -
Tarsius syrichta FGF2B           - ----------------------------------------------------------------- -
Otolemur garnettii FGF2B         - ----------------------------------------------------------------- -
Mus musculus Fgf2b               - ----------------------------------------------------------------- -
Rattus norvegicus Fgf2b          - ----------------------------------------------------------------- -
Cavia porcellus FGF2B            - ----------------------------------------------------------------- -
Oryctolagus cuniculus FGF2B      - ----------------------------------------------------------------- -
Tursiops truncatus FGF2B         - ----------------------------------------------------------------- -
Bos taurus FGF2B                 - ----------------------------------------------------------------- -
Equus caballus FGF2B             - ----------------------------------------------------------------- -
Canis lupus familiaris FGF2B     - ----------------------------------------------------------------- -
Felis catus FGF2B                - ----------------------------------------------------------------- -
Myotis lucifugus FGF2B           - ----------------------------------------------------------------- -
Pteropus vampyrus FGF2B          - ----------------------------------------------------------------- -
Dasypus novemcinctus FGF2B       - ----------------------------------------------------------------- -
Loxodonta africana FGF2B         - ----------------------------------------------------------------- -
Homo sapiens FGF3A               - ----------------------------------------------------------------- -
Pan troglodytes FGF3A            - ----------------------------------------------------------------- -
Nomascus leucogenys FGF3A        - ----------------------------------------------------------------- -
```

Macaca mulatta FGF3A	-	---	-
Papio hamadryas FGF3A	-	---	-
Callithrix jacchus FGF3A	-	---	-
Otolemur garnettii FGF3A	-	---	-
Mus musculus Fgf3a	-	---	-
Rattus norvegicus Fgf3a	-	---	-
Dipodomys ordii FGF3A	-	---	-
Cavia porcellus FGF3A	-	---	-
Oryctolagus cuniculus FGF3A	-	---	-
Bos taurus FGF3A	-	---	-
Equus caballus FGF3A	-	---	-
Canis lupus familiaris FGF3A	-	---	-
Myotis lucifugus FGF3A	-	---	-
Dasypus novemcinctus FGF3A	-	---	-
Homo sapiens FGF4A	-	---	-
Pan troglodytes FGF4A	-	---	-
Nomascus leucogenys FGF4A	-	---	-
Macaca mulatta FGF4A	-	---	-
Papio hamadryas FGF4A	-	---	-
Callithrix jacchus FGF4A	-	---	-
Mus musculus Fgf4a	-	---	-
Rattus norvegicus Fgf4a	-	---	-
Bos taurus FGF4A	-	---	-
Vicugna pacos FGF4A	-	---	-
Pteropus vampyrus FGF4A	-	---	-
Homo sapiens FGF4B	-	---	-
Pan troglodytes FGF4B	-	---	-
Gorilla gorilla FGF4B	-	---	-
Nomascus leucogenys FGF4B	-	---	-
Callithrix jacchus FGF4B	-	---	-
Otolemur garnettii FGF4B	-	---	-
Mus musculus Fgf4b	-	---	-
Rattus norvegicus Fgf4b	-	---	-
Oryctolagus cuniculus FGF4B	-	---	-
Tursiops truncatus FGF4B	-	---	-
Equus caballus FGF4B	-	---	-
Canis lupus familiaris FGF4B	-	---	-
Myotis lucifugus FGF4B	-	---	-
Pteropus vampyrus FGF4B	-	---	-
Dasypus novemcinctus FGF4B	-	---	-
Loxodonta africana FGF4B	-	---	-
Homo sapiens FGF4C	-	---	-
Pan troglodytes FGF4C	-	---	-
Gorilla gorilla FGF4C	-	---	-
Pongo abelii FGF4C	-	---	-
Nomascus leucogenys FGF4C	-	---	-
Otolemur garnettii FGF4C	-	---	-
Tupaia belangeri FGF4C	-	---	-
Mus musculus Fgf4c	-	---	-
Cavia porcellus FGF4C	-	---	-
Bos taurus FGF4C	-	---	-
Canis lupus familiaris FGF4C	-	---	-
Myotis lucifugus FGF4C	-	---	-
Pteropus vampyrus FGF4C	-	---	-
Dasypus novemcinctus FGF4C	-	---	-
Homo sapiens FGF5A	-	---	-
Pan troglodytes FGF5A	-	---	-
Nomascus leucogenys FGF5A	-	---	-
Macaca mulatta FGF5A	-	---	-
Callithrix jacchus FGF5A	-	---	-
Otolemur garnettii FGF5A	-	---	-
Tupaia belangeri FGF5A	-	---	-
Mus musculus Fgf5a	-	---	-
Rattus norvegicus Fgf5a	-	---	-
Cavia porcellus FGF5A	-	---	-
Oryctolagus cuniculus FGF5A	-	---	-
Myotis lucifugus FGF5A	-	---	-
Dasypus novemcinctus FGF5A	-	---	-
Loxodonta africana FGF5A	-	---	-
Homo sapiens FGF5B	-	---	-
Nomascus leucogenys FGF5B	-	---	-
Macaca mulatta FGF5B	-	---	-
Callithrix jacchus FGF5B	-	---	-
Mus musculus Fgf5b	-	---	-
Rattus norvegicus Fgf5b	-	---	-
Cavia porcellus FGF5B	-	---	-
Oryctolagus cuniculus FGF5B	-	---	-
Bos taurus FGF5B	-	---	-
Vicugna pacos FGF5B	-	---	-

Equus caballus FGF5B	-	---	-
Canis lupus familiaris FGF5B	-	---	-
Myotis lucifugus FGF5B	-	---	-
Sorex araneus FGF5B	-	---	-
Dasypus novemcinctus FGF5B	-	---	-
Loxodonta africana FGF5B	-	---	-
Homo sapiens FGF5C	-	---	-
Pongo abelii FGF5C	-	---	-
Macaca mulatta FGF5C	-	---	-
Mus musculus Fgf5c	-	---	-
Rattus norvegicus Fgf5c	-	---	-
Cavia porcellus FGF5C	-	---	-
Bos taurus FGF5C	-	---	-
Homo sapiens FGF5D	-	---	-
Macaca mulatta FGF5D	-	---	-
Papio hamadryas FGF5D	-	---	-
Otolemur garnettii FGF5D	-	---	-
Mus musculus Fgf5d	-	---	-
Rattus norvegicus Fgf5d	-	---	-
Bos taurus FGF5D	-	---	-
Myotis lucifugus FGF5D	-	---	-
Pteropus vampyrus FGF5D	-	---	-
Homo sapiens FGF6A	-	---	-
Mus musculus Fgf6a	-	---	-
Cavia porcellus FGF6A	-	---	-
Bos taurus FGF6A	-	---	-
Canis lupus familiaris FGF6A	-	---	-
Homo sapiens FGF6B	-	---	-
Pan troglodytes FGF6B	-	---	-
Nomascus leucogenys FGF6B	-	---	-
Macaca mulatta FGF6B	1	--------MEHVPGVLGEMTGSGLRGGDSGSVPNELCAPCTSFIEYEDGIEPRGLFPAPRILIPG	57
Mus musculus Fgf6b	-	---	-
Rattus norvegicus Fgf6b	-	---	-
Cavia porcellus FGF6B	1	--MGH	3
Canis lupus familiaris FGF6B	-	---	-
Felis catus FGF6B	1	--MIPDLHCIT	9
Myotis lucifugus FGF6B	-	---	-
Dasypus novemcinctus FGF6B	-	---	-
Loxodonta africana FGF6B	-	---	-
Homo sapiens FGF6C	1	MIGCPGSLHSQHLLRKFVFINVRGRGGLPPYLKAPCSSRKARAGALRWAGSSLPCSGLAGSPRVS	65
Nomascus leucogenys FGF6C	-	---	-
Macaca mulatta FGF6C	-	---	-
Papio hamadryas FGF6C	-	---	-
Mus musculus Fgf6c	-	---	-
Rattus norvegicus Fgf6c	-	---	-
Bos taurus FGF6C	-	---	-
Homo sapiens FGF7A	-	---	-
Macaca mulatta FGF7A	-	---	-
Callithrix jacchus FGF7A	-	---	-
Mus musculus Fgf7a	-	---	-
Rattus norvegicus Fgf7a	-	---	-
Cavia porcellus FGF7A	-	---	-
Canis lupus familiaris FGF7A	-	---	-
Procavia capensis FGF7A	-	---	-
Homo sapiens FGF7B	-	---	-
Pan troglodytes FGF7B	-	---	-
Pongo abelii FGF7B	-	---	-
Nomascus leucogenys FGF7B	-	---	-
Macaca mulatta FGF7B	-	---	-
Papio hamadryas FGF7B	-	---	-
Callithrix jacchus FGF7B	-	---	-
Mus musculus Fgf7b	-	---	-
Rattus norvegicus Fgf7b	-	---	-
Cavia porcellus FGF7B	-	---	-
Oryctolagus cuniculus FGF7B	-	---	-
Tursiops truncatus FGF7B	-	---	-
Bos taurus FGF7B	-	---	-
Equus caballus FGF7B	-	---	-
Canis lupus familiaris FGF7B	-	---	-
Myotis lucifugus FGF7B	-	---	-
Loxodonta africana FGF7B	-	---	-
Homo sapiens FGF8A	-	---	-
Pongo abelii FGF8A	-	---	-
Macaca mulatta FGF8A	-	---	-
Microcebus murinus FGF8A	1	---MLGCPGHLPAGPVR	14
Otolemur garnettii FGF8A	-	---	-
Mus musculus Fgf8a	-	---	-
Rattus norvegicus Fgf8a	-	---	-
Cavia porcellus FGF8A	-	---	-

Bos taurus FGF8A	-	---	-
Myotis lucifugus FGF8A	-	---	-
Pteropus vampyrus FGF8A	-	---	-
Dasypus novemcinctus FGF8A	-	---	-
Homo sapiens FGF8B	1	-MEVGQVNRQPRVADNFCPHVIHFLERWLEIEGCVCLEPTCLPCASGVFALSFNGLITTQRMWTV	64
Pan troglodytes FGF8B	1	-MEVGQVNRQPRVADNFCPHVIHFLERWLEIEGCVCLEPTCLPCASGVFALSFNGLITTQRMWTV	64
Nomascus leucogenys FGF8B	1	-MEVGQVNRQPRVADNFCPHVIHFLERWLEIEGCVCLEPTCLPCASGVFALSFNGLITTQRMWTV	64
Otolemur garnettii FGF8B	-	---	-
Mus musculus Fgf8b	-	---	-
Rattus norvegicus Fgf8b	1	---MITTQRMWQH	10
Cavia porcellus FGF8B	-	---	-
Oryctolagus cuniculus FGF8B	1	---MWTL	4
Equus caballus FGF8B	-	---	-
Canis lupus familiaris FGF8B	1	---MWTV	4
Dasypus novemcinctus FGF8B	-	---	-
Loxodonta africana FGF8B	-	---	-
Homo sapiens FGF8C	-	---	-
Pan troglodytes FGF8C	-	---	-
Pongo abelii FGF8C	-	---	-
Nomascus leucogenys FGF8C	-	---	-
Otolemur garnettii FGF8C	-	---	-
Mus musculus Fgf8c	-	---	-
Rattus norvegicus Fgf8c	-	---	-
Cavia porcellus FGF8C	-	---	-
Oryctolagus cuniculus FGF8C	-	---	-
Ochotona princeps FGF8C	-	---	-
Bos taurus FGF8C	-	---	-
Equus caballus FGF8C	-	---	-
Canis lupus familiaris FGF8C	-	---	-
Felis catus FGF8C	-	---	-
Sorex araneus FGF8C	-	---	-
Procavia capensis FGF8C	-	---	-

```
Homo sapiens FGF1A               - -----------------------------------------------------------------   -
                                       70        80        90        100       110       120       130
Homo sapiens FGF1A               - -----------------------------------------------------------------   -
Pan troglodytes FGF1A            - -----------------------------------------------------------------   -
Nomascus leucogenys FGF1A        - -----------------------------------------------------------------   -
Callithrix jacchus FGF1A         - -----------------------------------------------------------------   -
Mus musculus Fgf1a               - -----------------------------------------------------------------   -
Rattus norvegicus Fgf1a          - -----------------------------------------------------------------   -
Cavia porcellus FGF1A            - -----------------------------------------------------------------   -
Oryctolagus cuniculus FGF1A      - -----------------------------------------------------------------   -
Equus caballus FGF1A             - -----------------------------------------------------------------   -
Canis lupus familiaris FGF1A     - -----------------------------------------------------------------   -
Dasypus novemcinctus FGF1A       - -----------------------------------------------------------------   -
Homo sapiens FGF1B               - -----------------------------------------------------------------   -
Pan troglodytes FGF1B            - -----------------------------------------------------------------   -
Pongo abelii FGF1B               - -----------------------------------------------------------------   -
Macaca mulatta FGF1B             - -----------------------------------------------------------------   -
Callithrix jacchus FGF1B         - -----------------------------------------------------------------   -
Mus musculus Fgf1b               - -----------------------------------------------------------------   -
Oryctolagus cuniculus FGF1B      - -----------------------------------------------------------------   -
Equus caballus FGF1B             - -----------------------------------------------------------------   -
Dasypus novemcinctus FGF1B       - -----------------------------------------------------------------   -
Homo sapiens FGF1C               - -----------------------------------------------------------------   -
Pan troglodytes FGF1C            - -----------------------------------------------------------------   -
Pongo abelii FGF1C               - -----------------------------------------------------------------   -
Macaca mulatta FGF1C             - -----------------------------------------------------------------   -
Mus musculus Fgf1c               - -----------------------------------------------------------------   -
Rattus norvegicus Fgf1c          - -----------------------------------------------------------------   -
Cavia porcellus FGF1C            - -----------------------------------------------------------------   -
Bos taurus FGF1C                 - -----------------------------------------------------------------   -
Myotis lucifugus FGF1C           - -----------------------------------------------------------------   -
Dasypus novemcinctus FGF1C       - -----------------------------------------------------------------   -
Loxodonta africana FGF1C         - -----------------------------------------------------------------   -
Homo sapiens FGF1D               - -----------------------------------------------------------------   -
Pan troglodytes FGF1D            - -----------------------------------------------------------------   -
Nomascus leucogenys FGF1D        - -----------------------------------------------------------------   -
Macaca mulatta FGF1D             - -----------------------------------------------------------------   -
Otolemur garnettii FGF1D         - -----------------------------------------------------------------   -
Mus musculus Fgf1d               - -----------------------------------------------------------------   -
Rattus norvegicus Fgf1d          - -----------------------------------------------------------------   -
Cavia porcellus FGF1D            - -----------------------------------------------------------------   -
Oryctolagus cuniculus FGF1D      - -----------------------------------------------------------------   -
Bos taurus FGF1D                 - -----------------------------------------------------------------   -
Equus caballus FGF1D             - -----------------------------------------------------------------   -
Canis lupus familiaris FGF1D     - -----------------------------------------------------------------   -
Pteropus vampyrus FGF1D          - -----------------------------------------------------------------   -
Dasypus novemcinctus FGF1D       - -----------------------------------------------------------------   -
Loxodonta africana FGF1D         - -----------------------------------------------------------------   -
Homo sapiens FGF2A               - -----------------------------------------------------------------   -
Pongo abelii FGF2A               - -----------------------------------------------------------------   -
Nomascus leucogenys FGF2A        - -----------------------------------------------------------------   -
Macaca mulatta FGF2A             - -----------------------------------------------------------------   -
Mus musculus Fgf2a               - -----------------------------------------------------------------   -
Rattus norvegicus Fgf2a          - -----------------------------------------------------------------   -
Oryctolagus cuniculus FGF2A      - -----------------------------------------------------------------   -
Dasypus novemcinctus FGF2A       - -----------------------------------------------------------------   -
Homo sapiens FGF2B               - -----------------------------------------------------------------   -
Pan troglodytes FGF2B            - -----------------------------------------------------------------   -
Pongo abelii FGF2B               - -----------------------------------------------------------------   -
Nomascus leucogenys FGF2B        - -----------------------------------------------------------------   -
Callithrix jacchus FGF2B         - -----------------------------------------------------------------   -
Tarsius syrichta FGF2B           - -----------------------------------------------------------------   -
Otolemur garnettii FGF2B         - -----------------------------------------------------------------   -
Mus musculus Fgf2b               - -----------------------------------------------------------------   -
Rattus norvegicus Fgf2b          - -----------------------------------------------------------------   -
Cavia porcellus FGF2B            - -----------------------------------------------------------------   -
Oryctolagus cuniculus FGF2B      - -----------------------------------------------------------------   -
Tursiops truncatus FGF2B         - -----------------------------------------------------------------   -
Bos taurus FGF2B                 - -----------------------------------------------------------------   -
Equus caballus FGF2B             - -----------------------------------------------------------------   -
Canis lupus familiaris FGF2B     - -----------------------------------------------------------------   -
Felis catus FGF2B                - -----------------------------------------------------------------   -
Myotis lucifugus FGF2B           - -----------------------------------------------------------------   -
Pteropus vampyrus FGF2B          - -----------------------------------------------------------------   -
Dasypus novemcinctus FGF2B       - -----------------------------------------------------------------   -
Loxodonta africana FGF2B         - -----------------------------------------------------------------   -
Homo sapiens FGF3A               - -----------------------------------------------------------------   -
Pan troglodytes FGF3A            - -----------------------------------------------------------------   -
Nomascus leucogenys FGF3A        - -----------------------------------------------------------------   -
```

Macaca mulatta FGF3A	-	---	-
Papio hamadryas FGF3A	-	---	-
Callithrix jacchus FGF3A	-	---	-
Otolemur garnettii FGF3A	-	---	-
Mus musculus Fgf3a	-	---	-
Rattus norvegicus Fgf3a	-	---	-
Dipodomys ordii FGF3A	-	---	-
Cavia porcellus FGF3A	-	---	-
Oryctolagus cuniculus FGF3A	-	---	-
Bos taurus FGF3A	-	---	-
Equus caballus FGF3A	-	---	-
Canis lupus familiaris FGF3A	-	---	-
Myotis lucifugus FGF3A	-	---	-
Dasypus novemcinctus FGF3A	-	---	-
Homo sapiens FGF4A	-	---	-
Pan troglodytes FGF4A	-	---	-
Nomascus leucogenys FGF4A	-	---	-
Macaca mulatta FGF4A	-	---	-
Papio hamadryas FGF4A	-	---	-
Callithrix jacchus FGF4A	-	---	-
Mus musculus Fgf4a	-	---	-
Rattus norvegicus Fgf4a	-	---	-
Bos taurus FGF4A	-	---	-
Vicugna pacos FGF4A	-	---	-
Pteropus vampyrus FGF4A	-	---	-
Homo sapiens FGF4B	-	---	-
Pan troglodytes FGF4B	-	---	-
Gorilla gorilla FGF4B	-	---	-
Nomascus leucogenys FGF4B	-	---	-
Callithrix jacchus FGF4B	-	---	-
Otolemur garnettii FGF4B	-	---	-
Mus musculus Fgf4b	-	---	-
Rattus norvegicus Fgf4b	-	---	-
Oryctolagus cuniculus FGF4B	-	---	-
Tursiops truncatus FGF4B	-	---	-
Equus caballus FGF4B	-	---	-
Canis lupus familiaris FGF4B	-	---	-
Myotis lucifugus FGF4B	-	---	-
Pteropus vampyrus FGF4B	-	---	-
Dasypus novemcinctus FGF4B	-	---	-
Loxodonta africana FGF4B	-	---	-
Homo sapiens FGF4C	-	---	-
Pan troglodytes FGF4C	-	---	-
Gorilla gorilla FGF4C	-	---	-
Pongo abelii FGF4C	-	---	-
Nomascus leucogenys FGF4C	-	---	-
Otolemur garnettii FGF4C	-	---	-
Tupaia belangeri FGF4C	-	---	-
Mus musculus Fgf4c	-	---	-
Cavia porcellus FGF4C	-	---	-
Bos taurus FGF4C	-	---	-
Canis lupus familiaris FGF4C	-	---	-
Myotis lucifugus FGF4C	-	---	-
Pteropus vampyrus FGF4C	-	---	-
Dasypus novemcinctus FGF4C	-	---	-
Homo sapiens FGF5A	-	---	-
Pan troglodytes FGF5A	-	---	-
Nomascus leucogenys FGF5A	-	---	-
Macaca mulatta FGF5A	-	---	-
Callithrix jacchus FGF5A	-	---	-
Otolemur garnettii FGF5A	-	---	-
Tupaia belangeri FGF5A	-	---	-
Mus musculus Fgf5a	1	-------------MLVHSLLARSVPRGSYPEATTSSCLSASQQVLPFQYVPSDETISSAESFST-	51
Rattus norvegicus Fgf5a	-	---	-
Cavia porcellus FGF5A	-	---	-
Oryctolagus cuniculus FGF5A	-	---	-
Myotis lucifugus FGF5A	-	---	-
Dasypus novemcinctus FGF5A	-	---	-
Loxodonta africana FGF5A	-	---	-
Homo sapiens FGF5B	-	---	-
Nomascus leucogenys FGF5B	-	---	-
Macaca mulatta FGF5B	-	---	-
Callithrix jacchus FGF5B	-	---	-
Mus musculus Fgf5b	-	---	-
Rattus norvegicus Fgf5b	-	---	-
Cavia porcellus FGF5B	-	---	-
Oryctolagus cuniculus FGF5B	-	---	-
Bos taurus FGF5B	-	---	-
Vicugna pacos FGF5B	-	---	-

Equus caballus FGF5B	-	---	-
Canis lupus familiaris FGF5B	-	---	-
Myotis lucifugus FGF5B	-	---	-
Sorex araneus FGF5B	-	---	-
Dasypus novemcinctus FGF5B	-	---	-
Loxodonta africana FGF5B	-	---	-
Homo sapiens FGF5C	-	---	-
Pongo abelii FGF5C	-	---	-
Macaca mulatta FGF5C	-	---	-
Mus musculus Fgf5c	-	---	-
Rattus norvegicus Fgf5c	-	---	-
Cavia porcellus FGF5C	-	---	-
Bos taurus FGF5C	-	---	-
Homo sapiens FGF5D	-	---	-
Macaca mulatta FGF5D	-	---	-
Papio hamadryas FGF5D	-	---	-
Otolemur garnettii FGF5D	-	---	-
Mus musculus Fgf5d	-	---	-
Rattus norvegicus Fgf5d	-	---	-
Bos taurus FGF5D	1	MVCPRTTAPPRILTRPLWSWPRPRGFQLACVGRPGRLLGSLISQWQSRGCSRGGENGQRVGRVA-	64
Myotis lucifugus FGF5D	-	---	-
Pteropus vampyrus FGF5D	-	---	-
Homo sapiens FGF6A	-	---	-
Mus musculus Fgf6a	-	---	-
Cavia porcellus FGF6A	-	---	-
Bos taurus FGF6A	-	---	-
Canis lupus familiaris FGF6A	-	---	-
Homo sapiens FGF6B	1	-------------------------------------MAICPLHSAGQVACPHYIHLL-TPLPW-	26
Pan troglodytes FGF6B	1	-------------------------------------MAICPLHSAGQVACPHYIHLL-TPLPW-	26
Nomascus leucogenys FGF6B	1	-------------------------------------MAICPLHSAGQVACPHYIHLL-TPLPW-	26
Macaca mulatta FGF6B	58	LCSVNKCPPAWADPSPFCKVDAKPRSYDRLTQEPGKVMATCPLHSAGQLACPQYIHLL-TPLPW-	120
Mus musculus Fgf6b	1	-MIADEYCLSWCFPRLTITSTLKLTTGPCSILGRPWAEAVFSLHRTLQGTQPAPLHLGPHPSPH-	63
Rattus norvegicus Fgf6b	1	-MIADVYCLIV-FPKTDNHKSSKLTTDPCSTLGRSWAEAVFSSHHTLPGTQPAPLHLGPHPSPQ-	62
Cavia porcellus FGF6B	4	YNLEGPFLPPEKLFQVLLCPQSRHRMDTAAWRDLARCHFPLHPLL-CRMSGLCPLHLPPPLSPN-	66
Canis lupus familiaris FGF6B	1	---------------------------------MAIDTLPLHLCFPGQMACLYYTHLH-TPLPW-	30
Felis catus FGF6B	10	KGPPSLCGHQFSAPGQMGSQEVMTGSPAGLERPWPYAPFTLHLCFPGQMAWPPHTHLH-APLPW-	72
Myotis lucifugus FGF6B	-	---	-
Dasypus novemcinctus FGF6B	-	---	-
Loxodonta africana FGF6B	1	--MIGQMACPHHTHLL-TLLPWL	20
Homo sapiens FGF6C	66	PNRWVQLLPGARSSWAGSARLAWVGDGGLGWHVGGTPSTCWRLGAVRGTQGDGATRAPGRSSII-	129
Nomascus leucogenys FGF6C	-	---	-
Macaca mulatta FGF6C	-	---	-
Papio hamadryas FGF6C	-	---	-
Mus musculus Fgf6c	-	---	-
Rattus norvegicus Fgf6c	-	---	-
Bos taurus FGF6C	1	-------------------------------MLGGPQPLPAAWGSGGRCRVMGATRAPGQGSII-	33
Homo sapiens FGF7A	-	---	-
Macaca mulatta FGF7A	-	---	-
Callithrix jacchus FGF7A	-	---	-
Mus musculus Fgf7a	-	---	-
Rattus norvegicus Fgf7a	-	---	-
Cavia porcellus FGF7A	-	---	-
Canis lupus familiaris FGF7A	-	---	-
Procavia capensis FGF7A	-	---	-
Homo sapiens FGF7B	-	---	-
Pan troglodytes FGF7B	-	---	-
Pongo abelii FGF7B	-	---	-
Nomascus leucogenys FGF7B	-	---	-
Macaca mulatta FGF7B	-	---	-
Papio hamadryas FGF7B	-	---	-
Callithrix jacchus FGF7B	-	---	-
Mus musculus Fgf7b	-	---	-
Rattus norvegicus Fgf7b	-	---	-
Cavia porcellus FGF7B	-	---	-
Oryctolagus cuniculus FGF7B	-	---	-
Tursiops truncatus FGF7B	-	---	-
Bos taurus FGF7B	-	---	-
Equus caballus FGF7B	-	---	-
Canis lupus familiaris FGF7B	-	---	-
Myotis lucifugus FGF7B	-	---	-
Loxodonta africana FGF7B	-	---	-
Homo sapiens FGF8A	-	---	-
Pongo abelii FGF8A	-	---	-
Macaca mulatta FGF8A	-	---	-
Microcebus murinus FGF8A	15	RFIKLQARAAALSEHERCAPRTALLARGPLQPQFGAHSPQRIPVAAQPPAPLPPEPRREPHGAA-	78
Otolemur garnettii FGF8A	-	---	-
Mus musculus Fgf8a	-	---	-
Rattus norvegicus Fgf8a	-	---	-
Cavia porcellus FGF8A	-	---	-

```
Bos taurus FGF8A                 - -----------------------------------------------------------------   -
Myotis lucifugus FGF8A           - -----------------------------------------------------------------   -
Pteropus vampyrus FGF8A          - -----------------------------------------------------------------   -
Dasypus novemcinctus FGF8A       - -----------------------------------------------------------------   -
Homo sapiens FGF8B              65 EFFLFDVTLLPFKSLTAKRRESSLGSSHQLLAREKEKASKAWARRVPTGVSGFNLSTSHSEQGT- 128
Pan troglodytes FGF8B           65 EFFLFDVTLLPFKSLTAKRRESSLGSSHQLLAREKEKASKAWARRVPTGVSGFNLSTSHSEQGT- 128
Nomascus leucogenys FGF8B       65 EFFLFDVTLLPFKSLMAKRRESSLGSSRQLLAREKEKASEAWARRVRTGVSGFNLSTSHSEQGT- 128
Otolemur garnettii FGF8B         - -----------------------------------------------------------------   -
Mus musculus Fgf8b               - -----------------------------------------------------------------   -
Rattus norvegicus Fgf8b         11 CFSYLMSYHHPLKVPGKKGGNLAQDPHLSSQLLPR-KKTVRPARSQAVWECQISTSAIATQRCA-  73
Cavia porcellus FGF8B            - -----------------------------------------------------------------   -
Oryctolagus cuniculus FGF8B      5 GFFLFDVTLLPFKSLGGKRRECSRGSSRPSLLLAR-LSPEGSLRFQSQHL-------PFGERGA-  60
Equus caballus FGF8B             - -----------------------------------------------------------------   -
Canis lupus familiaris FGF8B     5 EFFLFDVTGPPFKSLREKRRESSLGLSRKIPTKKRRKRPVRHSRGIKEAVSGFKLQ-PAIQRAV-  67
Dasypus novemcinctus FGF8B       - -----------------------------------------------------------------   -
Loxodonta africana FGF8B         1 -----MSCCYPLKVRGKKGGNPASDPHSRYPQRRRGEKGQEGTGQRSQGECQISNLAPAIQRAA-  59
Homo sapiens FGF8C               - -----------------------------------------------------------------   -
Pan troglodytes FGF8C            - -----------------------------------------------------------------   -
Pongo abelii FGF8C               - -----------------------------------------------------------------   -
Nomascus leucogenys FGF8C        - -----------------------------------------------------------------   -
Otolemur garnettii FGF8C         - -----------------------------------------------------------------   -
Mus musculus Fgf8c               - -----------------------------------------------------------------   -
Rattus norvegicus Fgf8c          - -----------------------------------------------------------------   -
Cavia porcellus FGF8C            1 ----------------------------------------------------------MTRAPL-   6
Oryctolagus cuniculus FGF8C      - -----------------------------------------------------------------   -
Ochotona princeps FGF8C          - -----------------------------------------------------------------   -
Bos taurus FGF8C                 - -----------------------------------------------------------------   -
Equus caballus FGF8C             - -----------------------------------------------------------------   -
Canis lupus familiaris FGF8C     - -----------------------------------------------------------------   -
Felis catus FGF8C                - -----------------------------------------------------------------   -
Sorex araneus FGF8C              - -----------------------------------------------------------------   -
Procavia capensis FGF8C          - -----------------------------------------------------------------   -
```

```
Homo sapiens FGF1A              1 MAAAIAS----------------------------------------------------------   7
                                         140       150       160       170       180       190

Homo sapiens FGF1A              1 MAAAIAS----------------------------------------------------------   7
Pan troglodytes FGF1A           1 MAAAIAS----------------------------------------------------------   7
Nomascus leucogenys FGF1A       1 MAAAIAS----------------------------------------------------------   7
Callithrix jacchus FGF1A        1 MAAAIAS----------------------------------------------------------   7
Mus musculus Fgf1a              1 MAAAIAS----------------------------------------------------------   7
Rattus norvegicus Fgf1a         1 MAAAIAS----------------------------------------------------------   7
Cavia porcellus FGF1A           1 MAAAIAS----------------------------------------------------------   7
Oryctolagus cuniculus FGF1A     1 MAAAIAS----------------------------------------------------------   7
Equus caballus FGF1A            1 MAAAIAS----------------------------------------------------------   7
Canis lupus familiaris FGF1A    1 MAAAIAS----------------------------------------------------------   7
Dasypus novemcinctus FGF1A      1 MAAAIAS----------------------------------------------------------   7
Homo sapiens FGF1B              1 MAAAIAS----------------------------------------------------------   7
Pan troglodytes FGF1B           1 MAAAIAS----------------------------------------------------------   7
Pongo abelii FGF1B              1 MAAAIAS----------------------------------------------------------   7
Macaca mulatta FGF1B            1 MAAAIAS----------------------------------------------------------   7
Callithrix jacchus FGF1B        1 MAAAIAS----------------------------------------------------------   7
Mus musculus Fgf1b              1 MAAAIAS----------------------------------------------------------   7
Oryctolagus cuniculus FGF1B     1 MAAAIAS----------------------------------------------------------   7
Equus caballus FGF1B            1 MAAAIAS----------------------------------------------------------   7
Dasypus novemcinctus FGF1B      1 MAAAIAS----------------------------------------------------------   7
Homo sapiens FGF1C              1 MAAAIAS----------------------------------------------------------   7
Pan troglodytes FGF1C           1 MAAAIAS----------------------------------------------------------   7
Pongo abelii FGF1C              1 MAAAIAS----------------------------------------------------------   7
Macaca mulatta FGF1C            1 MAAAIAS----------------------------------------------------------   7
Mus musculus Fgf1c              1 MAAAIAS----------------------------------------------------------   7
Rattus norvegicus Fgf1c         1 MAAAIAS----------------------------------------------------------   7
Cavia porcellus FGF1C           1 MAAAIAS----------------------------------------------------------   7
Bos taurus FGF1C                1 MAAAIAS----------------------------------------------------------   7
Myotis lucifugus FGF1C          1 MAAAIAS----------------------------------------------------------   7
Dasypus novemcinctus FGF1C      1 MAAAIAS----------------------------------------------------------   7
Loxodonta africana FGF1C        1 MAAAIAS----------------------------------------------------------   7
Homo sapiens FGF1D              1 MAA-LAS----------------------------------------------------------   6
Pan troglodytes FGF1D           1 MAA-LAS----------------------------------------------------------   6
Nomascus leucogenys FGF1D       1 MAA-LAS----------------------------------------------------------   6
Macaca mulatta FGF1D            1 MAA-LAS----------------------------------------------------------   6
Otolemur garnettii FGF1D        1 MAA-LAS----------------------------------------------------------   6
Mus musculus Fgf1d              1 MAA-LAS----------------------------------------------------------   6
Rattus norvegicus Fgf1d         1 MAA-LAS----------------------------------------------------------   6
Cavia porcellus FGF1D           1 MAA-LAS----------------------------------------------------------   6
Oryctolagus cuniculus FGF1D     1 MAA-LAS----------------------------------------------------------   6
Bos taurus FGF1D                1 MAA-LAS----------------------------------------------------------   6
Equus caballus FGF1D            1 MAA-LAS----------------------------------------------------------   6
Canis lupus familiaris FGF1D    1 MAA-LAS----------------------------------------------------------   6
Pteropus vampyrus FGF1D         1 MAA-LAS----------------------------------------------------------   6
Dasypus novemcinctus FGF1D      1 MAA-LAS----------------------------------------------------------   6
Loxodonta africana FGF1D        1 MAA-LAS----------------------------------------------------------   6
Homo sapiens FGF2A              1 MA-AGS------------------ITTLP----------AL--PE--DGG---------------  17
Pongo abelii FGF2A              1 MA-AGS------------------ITTLP----------AL--PE--DGG---------------  17
Nomascus leucogenys FGF2A       1 MA-AGS------------------ITTLP----------AL--PE--DGG---------------  17
Macaca mulatta FGF2A            1 MA-AGS------------------ITTLP----------AL--PE--DGG---------------  17
Mus musculus Fgf2a              1 MA-ASG------------------ITSLP----------AL--PE--DGG---------------  17
Rattus norvegicus Fgf2a         1 MA-AGS------------------ITSLP----------AL--PE--DGG---------------  17
Oryctolagus cuniculus FGF2A     1 MA-AES------------------ITTLP----------AL--PE--DGG---------------  17
Dasypus novemcinctus FGF2A      1 MA-AGS------------------ITTLP----------AL--PE--DGG---------------  17
Homo sapiens FGF2B              1 MA-EGE------------------ITTFT----------AL--TE--K-----------------  15
Pan troglodytes FGF2B           1 MA-EGE------------------ITTFT----------AL--TE--K-----------------  15
Pongo abelii FGF2B              1 MA-EGE------------------ITTFT----------AL--TE--K-----------------  15
Nomascus leucogenys FGF2B       1 MA-EGE------------------ITTFT----------AL--TE--K-----------------  15
Callithrix jacchus FGF2B        1 MA-EGE------------------ITTFT----------AL--TE--K-----------------  15
Tarsius syrichta FGF2B          1 MA-EGE------------------ITTFT----------AL--TE--K-----------------  15
Otolemur garnettii FGF2B        1 MA-EGE------------------ITTFT----------AL--TE--K-----------------  15
Mus musculus Fgf2b              1 MA-EGE------------------ITTFA----------AL--TE--R-----------------  15
Rattus norvegicus Fgf2b         1 MA-EGE------------------ITTFA----------AL--TE--R-----------------  15
Cavia porcellus FGF2B           1 MA-EGE------------------ITTFA----------AL--TE--K-----------------  15
Oryctolagus cuniculus FGF2B     1 MA-EGE------------------VTTFT----------AL--TE--K-----------------  15
Tursiops truncatus FGF2B        1 MA-EGE------------------ITTFT----------AL--TE--K-----------------  15
Bos taurus FGF2B                1 MA-EGE------------------TTTFT----------AL--TE--K-----------------  15
Equus caballus FGF2B            1 MA-EGE------------------ITTFT----------AL--TE--K-----------------  15
Canis lupus familiaris FGF2B    1 MA-EGE------------------ITTFT----------AL--TE--K-----------------  15
Felis catus FGF2B               1 MA-EGE------------------ITTFT----------AL--TE--K-----------------  15
Myotis lucifugus FGF2B          1 MA-EGE------------------VTTFT----------AL--TE--R-----------------  15
Pteropus vampyrus FGF2B         1 MA-EGE------------------VTTFT----------AL--TE--R-----------------  15
Dasypus novemcinctus FGF2B      1 MA-EGE------------------ITTFM----------AL--ME--K-----------------  15
Loxodonta africana FGF2B        1 MA-EGE------------------ITTFT----------AL--TE--K-----------------  15
Homo sapiens FGF3A              1 MSLSFLL-----------------LLFFS-------HL-IL--SA-WA---------------HG  22
Pan troglodytes FGF3A           1 MSLSFLL-----------------LLFFS-------HL-IL--SA-WA---------------HG  22
Nomascus leucogenys FGF3A       1 MSLSFL------------------LLFFS-------HL-IL--SA-WA---------------HG  21
```

Macaca mulatta FGF3A	1	MSLSFLL-----------------LLFFS-------HL-IL--NA-WA---------------HG	22
Papio hamadryas FGF3A	1	MSLSFLL-----------------LLFFS-------HL-IL--NA-WA---------------HG	22
Callithrix jacchus FGF3A	1	MSLSSFL-----------------LLFFS-------HL-IL--SA-WA---------------HG	22
Otolemur garnettii FGF3A	1	MSLSFLL-----------------LLSFS-------HL-IL--SA-WA---------------HG	22
Mus musculus Fgf3a	1	MSLSLLF-----------------LIFCS-------HL-IH--SA-WA---------------HG	22
Rattus norvegicus Fgf3a	1	MSLSLLF-----------------LIFCS-------HL-IL--SA-PA---------------QG	22
Dipodomys ordii FGF3A	1	MSLPFLF-----------------LLFLS-------HL-IV--SA-WA---------------HG	22
Cavia porcellus FGF3A	1	MSLSFFL-----------------LLLLS-------HL-IL--SA-WA---------------HE	22
Oryctolagus cuniculus FGF3A	1	MSLSFLL-----------------LLFFS-------HL-IF--SA-WA---------------HG	22
Bos taurus FGF3A	1	MSLSFLL-----------------LLFLS-------HL-IL--SA-WA---------------QG	22
Equus caballus FGF3A	1	MSLSF-L-----------------LLFLS-------HL-IL--SA-WA---------------HG	21
Canis lupus familiaris FGF3A	1	MSLSLLL-----------------LLFLS-------HL-IL--SA-WA---------------HG	22
Myotis lucifugus FGF3A	1	MSLSF-L-----------------LLFLS-------HL-IL--SA-WA---------------QG	21
Dasypus novemcinctus FGF3A	1	MSLSFLL-----------------LLCLS-------HL-IL--SA-WT---------------HE	22
Homo sapiens FGF4A	1	MAPLAEV-----------------GGFL---GGL--E--GL---G----------------QQVG	22
Pan troglodytes FGF4A	1	MAPLAEV-----------------GGFL---GGL--E--GL---G----------------QQVG	22
Nomascus leucogenys FGF4A	1	MAPLAEV-----------------GGFL---GGL--E--GL---G----------------QQVG	22
Macaca mulatta FGF4A	1	MAPLAEV-----------------GGFL---GGL--E--GL---G----------------QQVG	22
Papio hamadryas FGF4A	1	MAPLAEV-----------------GGFL---GGL--E--GL---G----------------QQVG	22
Callithrix jacchus FGF4A	1	MAPLAEV-----------------GSFL---GGL--E--GL---G----------------QQVG	22
Mus musculus Fgf4a	1	MAPLTEV-----------------GAFL---GGL--E--GL---S----------------QQVG	22
Rattus norvegicus Fgf4a	1	MAPLTEV-----------------GAFL---GGL--E--GL---G----------------QQVG	22
Bos taurus FGF4A	1	MAPLAEV-----------------GGFL---GGL--E--GL---G----------------QQVG	22
Vicugna pacos FGF4A	1	MAPLAEV-----------------GGFL---GGL--E--SL---G----------------QQVG	22
Pteropus vampyrus FGF4A	1	MAPLAEV-----------------GGFL---GGL--E--GL---G----------------QQVG	22
Homo sapiens FGF4B	1	MAPLGEV-----------------GNYF---GVQ--D--AV---P----------------F--G	20
Pan troglodytes FGF4B	1	MAPLGEV-----------------GNYF---GVQ--D--AV---P----------------F--G	20
Gorilla gorilla FGF4B	1	MAPLGEV-----------------GNYF---GVQ--D--AV---P----------------F--G	20
Nomascus leucogenys FGF4B	1	MAPLGEV-----------------GNYF---GVQ--D--AV---P----------------F--G	20
Callithrix jacchus FGF4B	1	MAPLGEV-----------------GNYF---GVQ--D--AV---P----------------F--G	20
Otolemur garnettii FGF4B	1	MAPLGEV-----------------GNYF---GVQ--D--AV---P----------------F--G	20
Mus musculus Fgf4b	1	MAPLGEV-----------------GSYF---GVQ--D--AV---P----------------F--G	20
Rattus norvegicus Fgf4b	1	MAPLGEV-----------------GSYF---GVQ--D--AV---P----------------F--G	20
Oryctolagus cuniculus FGF4B	1	MAPLGEV-----------------GNYF---GVQ--D--AV---P----------------F--G	20
Tursiops truncatus FGF4B	1	MAPLGEV-----------------GNYF---GVQ--D--AA---P----------------F--G	20
Equus caballus FGF4B	1	MAPLGEV-----------------GNYF---GVQ--D--AV---P----------------F--G	20
Canis lupus familiaris FGF4B	1	MAPLGEV-----------------GNYF---GVQ--D--AV---P----------------F--G	20
Myotis lucifugus FGF4B	1	MAPLGEV-----------------GNYF---GVQ--D--AV---P----------------F--G	20
Pteropus vampyrus FGF4B	1	MAPLGEV-----------------GNYF---GVQ--D--AV---P----------------F--G	20
Dasypus novemcinctus FGF4B	1	MAPLGEV-----------------GNYF---GVQ--D--AV---P----------------F--G	20
Loxodonta africana FGF4B	1	MAPLGEV-----------------GNYF---GVQ--D--AV---P----------------F--G	20
Homo sapiens FGF4C	1	MA---EV-----------------GGVF---ASL--DW-DL-H-G----------------FS-S	20
Pan troglodytes FGF4C	1	MA---EV-----------------GGVF---ASL--DW-DL-H-G----------------FS-S	20
Gorilla gorilla FGF4C	1	MA---EV-----------------GGVF---ASL--DW-DL-Q-G----------------FS-S	20
Pongo abelii FGF4C	1	MA---EV-----------------GGVF---ASL--DW-DL-H-G----------------FS-S	20
Nomascus leucogenys FGF4C	1	MA---EV-----------------GGVF---ASL--DW-DL-H-G----------------FS-S	20
Otolemur garnettii FGF4C	1	MA---EV-----------------GGVF---GSL--DW-DL-H-G----------------FS-S	20
Tupaia belangeri FGF4C	1	MS---EV-----------------GGVF---TSL--DW-DL-H-G----------------FS-S	20
Mus musculus Fgf4c	1	MA---EV-----------------GGVF---ASL--DW-DL-H-G----------------FS-S	20
Cavia porcellus FGF4C	1	MA---EV-----------------AGVF---ASL--DW-DL-H-G----------------FS-S	20
Bos taurus FGF4C	1	MA---EV-----------------GGVF---ASL--DW-DL-H-G----------------FS-S	20
Canis lupus familiaris FGF4C	1	MA---EV-----------------GGVF---ASL--DW-DL-H-G----------------FS-S	20
Myotis lucifugus FGF4C	1	MA---EV-----------------GGVF---ATL--DW-DL-H-G----------------FS-S	20
Pteropus vampyrus FGF4C	1	MA---EV-----------------GGVF---ASL--DW-DL-H-G----------------FS-S	20
Dasypus novemcinctus FGF4C	1	MA---EV-----------------GGVF---ASL--DW-DL-H-G----------------FS-A	20
Homo sapiens FGF5A	1	M-WK-WI------------------LT---------H-CAS-AFP--HLPG-CCCCCFLLLFLVS	31
Pan troglodytes FGF5A	1	M-WK-WI------------------LT---------H-CAS-AFP--HLPG-CCCCCFLLLFLVS	31
Nomascus leucogenys FGF5A	1	M-WK-WI------------------LT---------H-CAS-AFP--HLPG-CCCCCFLLLFLVS	31
Macaca mulatta FGF5A	1	M-WK-WI------------------LT---------H-CAS-AFP--HLPG-CCCCCFLLLFLVS	31
Callithrix jacchus FGF5A	1	M-WK-WI------------------LT---------H-CAS-AFP--HLPGGCCCCCFLLLFLVS	32
Otolemur garnettii FGF5A	1	M-WK-WI------------------LT---------H-CAS-AFP--HLPG-CCCCCFLLLFLVS	31
Tupaia belangeri FGF5A	1	M-WK-WI------------------LT---------H-CAS-AFP--HLPG-CCCCCFLLLFLVS	31
Mus musculus Fgf5a	52	M-WK-WI------------------LT---------H-CAS-AFP--HLPG-CC-CCFLLLFLVS	81
Rattus norvegicus Fgf5a	1	M-WK-WI------------------LT---------H-CAS-AFP--HLPG-CC-CCFLLLFLVS	30
Cavia porcellus FGF5A	1	M-WK-WI------------------LT---------H-CAS-AFP--HLPG-CC-CCFLLLFLVS	30
Oryctolagus cuniculus FGF5A	1	M-WK-WI------------------LT---------H-CAS-AFP--HLPGGCC-WCFLLLFLVS	31
Myotis lucifugus FGF5A	1	M-WK-WE------------------LT---------H-GAS-ASP--HLPG-CCCCCFLLLFLVS	31
Dasypus novemcinctus FGF5A	1	M-WK-WI------------------LT---------H-CAS-AFP--HLPG-CC-FCFLLLLLVS	30
Loxodonta africana FGF5A	1	M-WK-WM------------------LT---------H-CAS-AFP--HLPG-CC-CCFLLLLLVS	30
Homo sapiens FGF5B	1	M-HK-WI------------------LTWI-LPTLLYRSC----F---HI-I--C--------LVG	26
Nomascus leucogenys FGF5B	1	M-HK-WI------------------LTWI-LPTLLYRSY----F---HI-I--C--------LVG	26
Macaca mulatta FGF5B	1	M-HK-WI------------------LTWI-LPTLLYRSC----F---HI-I--C--------LVG	26
Callithrix jacchus FGF5B	1	M-HK-WI------------------LTWI-LPTLLYRSC----F---HI-I--C--------LVG	26
Mus musculus Fgf5b	1	M-RK-WI------------------LTRI-LPTLLYRSC----F---HL-V--C--------LVG	26
Rattus norvegicus Fgf5b	1	M-RK-WI------------------LTRI-LPTPLYRSC----F---HL-V--C--------LVG	26
Cavia porcellus FGF5B	1	M-HK-WI------------------LSWI-LPTLLYRSC----F---LG-V--C--------LVG	26
Oryctolagus cuniculus FGF5B	1	M-HK-WI------------------LTWI-LPTLLYRSC----F---HI-I--C--------LVA	26
Bos taurus FGF5B	1	M-RK-WI------------------LTWI-LPSLLYRSC----F---HI-I--C--------LVG	26
Vicugna pacos FGF5B	1	M-RK-WI------------------LTWI-LPSLLYRSC----F---HI-I--C--------LVG	26

```
Equus caballus FGF5B               1 M-RK-WI------------------LTWI-LPTLLYRSC----F---HI-I--C--------LVG  26
Canis lupus familiaris FGF5B       1 M-RK-WI------------------LTWI-LPTLLYRSC----F---HI-I--C--------LVG  26
Myotis lucifugus FGF5B             1 M-RK-WI------------------LTWI-LPTLLYSSC----F---HI-I--C--------LVG  26
Sorex araneus FGF5B                1 M-RK-WI------------------LTWI-LPTLLYRSF----F---HI-I--C--------LVG  26
Dasypus novemcinctus FGF5B         1 M-HK-WI------------------LTWI-LPTLLYRSF----F---HV-I--C--------LVG  26
Loxodonta africana FGF5B           1 M-HR-WV------------------LTWV-LPTLLYRSC----L---HL-I--C--------LVG  26
Homo sapiens FGF5C                 1 M-G---L------------------I-WLLLLSLL-E--P--GWP-AAGPG--------------  22
Pongo abelii FGF5C                 1 M-G---L------------------I-WLLLLSLL-E--P--GWP-AAGPG--------------  22
Macaca mulatta FGF5C               1 M-G---L------------------I-WLLLLSLL-E--P--GWP-AAGPG--------------  22
Mus musculus Fgf5c                 1 M-G---L------------------I-WLLLLSLL-E--P--SWP-TTGPG--------------  22
Rattus norvegicus Fgf5c            1 M-G---L------------------I-WLLLLSLL-E--P--GWP-ATGPG--------------  22
Cavia porcellus FGF5C              1 M-G---L------------------I-WLLLLSLL-E--P--GWP-AADPG--------------  22
Bos taurus FGF5C                   1 M-D---L------------------I-WLLLLSLL-E--P--GWP-AAGPV--------------  22
Homo sapiens FGF5D                 1 MRRRLWL-----------------GLAWLLL--------AR-A-P-DAA-G--------------  22
Macaca mulatta FGF5D               1 MRRRLWL-----------------GLAWLLL--------AR-A-P-DAG-G--------------  22
Papio hamadryas FGF5D              1 MRRRLWL-----------------GLAWLLL--------AR-A-P-DAA-G--------------  22
Otolemur garnettii FGF5D           1 MRRRLWL-----------------GLVWLLL--------AQ-E-P-DA-----------------  20
Mus musculus Fgf5d                 1 MRSRLWL-----------------GLAWLLL--------AR-A-P-GA-----------------  20
Rattus norvegicus Fgf5d            1 MRRRLWL-----------------GLAWLLL--------AR-A-P-GA-----------------  20
Bos taurus FGF5D                  65 MRGRLWL-----------------GLVWLLL--------AR-A-P-GTA-G--------------  86
Myotis lucifugus FGF5D             1 MRCRLWL-----------------GLAWLLL--------AR-A-P-GAA-R--------------  22
Pteropus vampyrus FGF5D            1 MCGRLWL-----------------GLAWLLL--------AR-A-P-GAA-G--------------  22
Homo sapiens FGF6A                 1 MYSAPSACTCLCLHFLLLCFQVQV-----------------------------------------  24
Mus musculus Fgf6a                 1 MYSAPSACTCLCLHFLLLCFQVQV-----------------------------------------  24
Cavia porcellus FGF6A              1 MYSAPSACSCLCLHFLLLCFQVQV-----------------------------------------  24
Bos taurus FGF6A                   1 MYSAPSACTCLCLHFLLLCFQVQV-----------------------------------------  24
Canis lupus familiaris FGF6A       1 MYSAPSACTCLCLHFLLLCFQVQV-----------------------------------------  24
Homo sapiens FGF6B                27 MDQW-W-CH--------------------------------------------------------  33
Pan troglodytes FGF6B             27 MDQW-W-SH--------------------------------------------------------  33
Nomascus leucogenys FGF6B         27 MDQW-W-CH--------------------------------------------------------  33
Macaca mulatta FGF6B             121 MDQW-W-CH-------------------------------------------------------- 127
Mus musculus Fgf6b                64 MGQW-W-CH--------------------------------------------------------  70
Rattus norvegicus Fgf6b           63 MGQW-W-CH--------------------------------------------------------  69
Cavia porcellus FGF6B             67 -GPA-M-VS--------------------------------------------------------  72
Canis lupus familiaris FGF6B      31 MDPW-W-CH--------------------------------------------------------  37
Felis catus FGF6B                 73 MDQW-W-CH--------------------------------------------------------  79
Myotis lucifugus FGF6B             1 MDQR-W-CH--------------------------------------------------------   7
Dasypus novemcinctus FGF6B         - -----------------------------------------------------------------   -
Loxodonta africana FGF6B          21 -DWW-W-CY--------------------------------------------------------  26
Homo sapiens FGF6C               130 M--V-Y-C--------------------------------------------------------- 133
Nomascus leucogenys FGF6C          1 M--V-Y-C---------------------------------------------------------   4
Macaca mulatta FGF6C               1 M--A-Y-C---------------------------------------------------------   4
Papio hamadryas FGF6C              1 M--A-Y-C---------------------------------------------------------   4
Mus musculus Fgf6c                 1 M--A-C-C---------------------------------------------------------   4
Rattus norvegicus Fgf6c            1 M--A-C-C---------------------------------------------------------   4
Bos taurus FGF6C                  34 M--A-C-C---------------------------------------------------------  37
Homo sapiens FGF7A                 1 MS-GPG----------------------T-AAVA--LL-PA--VL-LALL--------APWAGRG  27
Macaca mulatta FGF7A               1 MS-GPG----------------------T-AAVA--LL-PA--VL-LALL--------APWAGRG  27
Callithrix jacchus FGF7A           1 MS-GPG----------------------T-AAAA--LL-PA--VL-LALL--------APWAGRG  27
Mus musculus Fgf7a                 1 MA-KRG----------------------P-TTGT--LL-PR--VL-LALV--------VALADRG  27
Rattus norvegicus Fgf7a            1 MA-KRG----------------------P-TTGT--LL-PG--VL-LALV--------VALADRG  27
Cavia porcellus FGF7A              1 MV-GPG----------------------T-VAAA--LL-PG--LL-LALL--------GPWAGQG  27
Canis lupus familiaris FGF7A       1 MA-GPG----------------------A-AAAA--LL-PA--VL-LAVL--------APWAGRG  27
Procavia capensis FGF7A            1 MA----------------------------------LL-PA--LL-LALL--------TPWAGRG  19
Homo sapiens FGF7B                 1 MALGQKL-----------------FITMS-RGAG--RL-QG--TL-WALV--------FLGILVG  33
Pan troglodytes FGF7B              1 MALGQKL-----------------FITMS-RGAG--HL-QG--TL-WALV--------FLGILVG  33
Pongo abelii FGF7B                 1 MALGQRL-----------------FITMS-RGAG--RL-QG--TL-WALV--------FLGILVG  33
Nomascus leucogenys FGF7B          1 MALGQRL-----------------FITMS-RGAG--RL-QG--TL-WALV--------FLGILAG  33
Macaca mulatta FGF7B               1 MALGQRL-----------------FITMS-RGAG--RL-RG--TL-WALV--------FLGILVG  33
Papio hamadryas FGF7B              1 MALGQRL-----------------FITMS-RGAG--RL-RG--TL-WALV--------FLGILVG  33
Callithrix jacchus FGF7B           1 MALGQRL-----------------FITMS-QGAG--RL-QG--TL-WALI--------FLGILVG  33
Mus musculus Fgf7b                 1 MALGQRL-----------------FITMS-RGAG--RV-QG--TL-QALV--------FLGVLVG  33
Rattus norvegicus Fgf7b            1 MALGQRL-----------------FITMS-RGAG--RV-QG--TL-QALV--------FLGVLVG  33
Cavia porcellus FGF7B              1 MALEQRL-----------------FITMS-RTAG--HL-QR--TL-QALV--------FLGILVG  33
Oryctolagus cuniculus FGF7B        1 MARGQRL-----------------FITMS-RGAG--RP-QG--TL-RALV--------FLGVLVG  33
Tursiops truncatus FGF7B           1 MALGQRQ-----------------LITMS-QGAG--RL-QG--TL-RALV--------FLGVLVG  33
Bos taurus FGF7B                   1 MARGQTP-----------------LITMS-RGAG--RP-QG--TL-RALV--------FLGVLVG  33
Equus caballus FGF7B               1 MALGQRL-----------------FITMS-RGAG--RL-QG--TL-RALV--------FLGVLVG  33
Canis lupus familiaris FGF7B       1 MALGQRL-----------------FITMS-RGAG--RL-QG--TL-WALV--------FLGVLVG  33
Myotis lucifugus FGF7B             1 MALGQRL-----------------FIAMS-RGAG--RL-QG--TL-QALL--------FLGVLVG  33
Loxodonta africana FGF7B           1 MALGQRL-----------------FITMS-RGAG--RL-QG--TL-QALV--------FLGVLVG  33
Homo sapiens FGF8A                 1 MR----S-----------------GCVVV-------H--VW--IL--AGL-------WL--AVAG  22
Pongo abelii FGF8A                 1 MR----S-----------------GCVVV-------H--AW--IL--AGL-------WL--AVAG  22
Macaca mulatta FGF8A               1 MR----S-----------------GCVVV-------H--AW--IL--ASL-------WL--AVAG  22
Microcebus murinus FGF8A          79 MP-SGQS-----------------GCVAA-------R--AL--IL--AGL-------WL--TAAG 103
Otolemur garnettii FGF8A           1 MP-SGLR-----------------GRVVA-------G--AL--AL--ASF-------WL--AVAG  25
Mus musculus Fgf8a                 1 MA-RKWN-----------------GRAVA-------R--AL--VL--ATL-------WL--AVSG  25
Rattus norvegicus Fgf8a            1 MA-RKWS-----------------GRIVA-------R--AL--VL--ATL-------WL--AVSG  25
Cavia porcellus FGF8A              1 MW-SAPS-----------------GCVVI-------R--AL--VL--AGL-------WL--AVAG  25
```

```
Bos taurus FGF8A                 1 MR-SAPS-----------------RCAVA-------R--AL--VL--AGL-------WL--AAAG  25
Myotis lucifugus FGF8A           1 MQ-SAWS-----------------RRVVA-------R--AL--VL--ASL-------GL--ASAG  25
Pteropus vampyrus FGF8A          1 MR----S-----------------PCAVA-------R--AL--VL--AGL-------WL--ASAA  22
Dasypus novemcinctus FGF8A       1 MR-GAPS-----------------GRTVA-------R--VL--AL--AGL-------WL--AAAG  25
Homo sapiens FGF8B             129 ML-GAR------------------LRLWV---------CALC-SVC-S--------------MSV 149
Pan troglodytes FGF8B          129 ML-GAR------------------LRLWV---------CALC-SVC-S--------------VSV 149
Nomascus leucogenys FGF8B      129 ML-GAR------------------LRLWV---------CALC-SVC-S--------------MSV 149
Otolemur garnettii FGF8B         1 ML-GTC------------------LRLWV---------CALC-SVC-S--------------VSI  21
Mus musculus Fgf8b               1 ML-GTC------------------LRLLV---------GVLC-TVC-S--------------LGT  21
Rattus norvegicus Fgf8b         74 ML-GAC------------------LRLLV---------GALC-TVC-S--------------LGT  94
Cavia porcellus FGF8B            1 ML-GTC------------------LGLLA------------C-TV--S--------------L-V  16
Oryctolagus cuniculus FGF8B     61 ML-GARL-----------------LRLLV---------CALG-SVC-S--------------WCV  82
Equus caballus FGF8B             1 MS-GPC------------------LGLLV---------YVLC-SA--------------------  16
Canis lupus familiaris FGF8B    68 MS-GTR------------------LGFLV---------SVLC-WV--------------------  83
Dasypus novemcinctus FGF8B       1 MS-GAR------------------LRLLV---------CALG-SVC------------------L  18
Loxodonta africana FGF8B        60 ML-GAR------------------LRLWV---------CTLC-SAC-S--------------MCS  80
Homo sapiens FGF8C               1 MD-SDE-------------------TGFE-------H----------SGL-------WVS-VLAG  20
Pan troglodytes FGF8C            1 MD-SDE-------------------TGFE-------H----------SGL-------WVS-VLAG  20
Pongo abelii FGF8C               1 MD-SDE-------------------TGFE-------H----------SGL-------WVP-VLAG  20
Nomascus leucogenys FGF8C        1 MD-SDE-------------------TGFE-------H----------SGL-------WVP-VLAG  20
Otolemur garnettii FGF8C         1 MG-WDKA-----------------RTGFK-------H----------PGP-------WFP-LLAV  22
Mus musculus Fgf8c               1 ME-WMRS-----------------RVG---------T----------LGL-------WVRLLLAV  21
Rattus norvegicus Fgf8c          1 MD-WMKS-----------------RVG---------A----------PGL-------WVCLLLPV  21
Cavia porcellus FGF8C            7 MD-WAR-------------------TECE-------R----------PRL-------WVS-MLAI  26
Oryctolagus cuniculus FGF8C      1 MD-WGK-------------------AKCR-------P----------PGL-------WVP-ALAA  20
Ochotona princeps FGF8C          1 MD------------------------GLQ-------P----------PGL-------RVP-VLAA  16
Bos taurus FGF8C                 1 MG-WDE-------------------AKFK-------H----------LGL-------WVP-VLAV  20
Equus caballus FGF8C             1 MD-WDK-------------------TGFK-------Y----------QGL-------WVP-VLAV  20
Canis lupus familiaris FGF8C     1 MG-WAE-------------------AGFE-------H----------LGL-------WVP-VLAV  20
Felis catus FGF8C                1 MG-WDE-------------------AGSQ-------R----------LGL-------WVLMMLGV  21
Sorex araneus FGF8C              1 MV-WDK-------------------ARGQ-------Q----------LGL-------WAP-MLLG  20
Procavia capensis FGF8C          1 MD-WAK-------------------FGIE-------H----------PGL-------WVP-VMAV  20
```

```
Homo sapiens FGF1A              8 --SL-I--RQKRQARE-SNSDR-VSAS-KRR---SSPSKDGRSLCERHVL---------------  46
                                     200       210       220       230       240       250       260
Homo sapiens FGF1A              8 --SL-I--RQKRQARE-SNSDR-VSAS-KRR---SSPSKDGRSLCERHVL---------------  46
Pan troglodytes FGF1A           8 --SL-I--RQKRQARE-SNSDR-VSAS-KRR---SSPSKDGRSLCERHVL---------------  46
Nomascus leucogenys FGF1A       8 --SL-I--RQKRQARE-SNSDR-VSAS-KRR---SSPSKDGRSLCERHVL---------------  46
Callithrix jacchus FGF1A        8 --SL-I--RQKRQARE-SNSDR-VSAS-KRR---SSPSKDGRSLCERHVL---------------  46
Mus musculus Fgf1a              8 --SL-I--RQKRQARE-SNSDR-VSAS-KRR---SSPSKDGRSLCERHVL---------------  46
Rattus norvegicus Fgf1a         8 --SL-I--RQKRQARE-SNSDR-VSAS-KRR---SSPSKDGRSLCERHVL---------------  46
Cavia porcellus FGF1A           8 --SL-I--RQKRQARE-SNSDR-VSAS-KRR---SSPSKDGRSLCERHVL---------------  46
Oryctolagus cuniculus FGF1A     8 --SL-I--RQKRQARE-SNSDR-VSAS-KRR---SSPSKDGRSLCERHVL---------------  46
Equus caballus FGF1A            8 --SL-I--RQKRQARE-SNSDR-VSAS-KRR---SSPSKDGRSLCERHVL---------------  46
Canis lupus familiaris FGF1A    8 --SL-I--RQKRQARE-SNSDR-VSAS-KRR---SSPSKDGRSLCERHVL---------------  46
Dasypus novemcinctus FGF1A      8 --SL-I--RQKRQARE-SNSDR-VSAS-KRR---SSPSKDGRSLCERHVL---------------  46
Homo sapiens FGF1B              8 --GL-I--RQKRQARE-QHWDRP-SAS-RRR---SSPSKN-RGLCNGNLV---------------  45
Pan troglodytes FGF1B           8 --GL-I--RQKRQARE-QHWDRP-SAS-RRR---SSPSKN-RGLCNGNLV---------------  45
Pongo abelii FGF1B              8 --GL-I--RQKRQARE-QHWDRP-SAS-RRR---SSPSKN-RGLCNGNLV---------------  45
Macaca mulatta FGF1B            8 --GL-I--RQKRQARE-QHWDRP-SAS-RRR---SSPSKN-RGLCNGNLV---------------  45
Callithrix jacchus FGF1B        8 --GL-I--RQKRQARE-QHWDRP-SAS-RRR---SSPSKN-RGLCNGNLV---------------  45
Mus musculus Fgf1b              8 --GL-I--RQKRQARE-QHWDRP-SAS-RRR---SSPSKN-RGLCNGNLV---------------  45
Oryctolagus cuniculus FGF1B     8 --GL-I--RQKRQARE-QHWDRP-SAS-RRR---SSPSKN-RGLCNGNLV---------------  45
Equus caballus FGF1B            8 --GL-I--RQKRQARE-QHWDRP-SAS-RRR---SSPSKN-RGLCNGNLV---------------  45
Dasypus novemcinctus FGF1B      8 --GL-I--RQKRQARE-QHWDRP-SAS-RRR---SSPSKN-RGLCNGNLV---------------  45
Homo sapiens FGF1C              8 --SL-I--RQKRQARE--R-EK--SNACK---CVSSPSKG-KTSCDKNKL---------------  43
Pan troglodytes FGF1C           8 --SL-I--RQKRQARE--R-EK--SNACK---CVSSPSKG-KTSCDKNKL---------------  43
Pongo abelii FGF1C              8 --SL-I--RQKRQARE--R-EK--SNACK---CVSSPSKG-KTSCDKNKL---------------  43
Macaca mulatta FGF1C            8 --SL-I--RQKRQARE--R-EK--SNACK---CVSSPSKG-KTSCDKNKL---------------  43
Mus musculus Fgf1c              8 --SL-I--RQKRQARE--R-EK--SNACK---CVSSPSKG-KTSCDKNKL---------------  43
Rattus norvegicus Fgf1c         8 --SL-I--RQKRQARE--R-EK--SNACK---CVSSPSKG-KTSCDKNKL---------------  43
Cavia porcellus FGF1C           8 --SL-I--RQKRQARE--R-EK--SNACK---CVSSPSKG-KTSCDKNKL---------------  43
Bos taurus FGF1C                8 --SL-I--RQKRQARE--R-EK--SNACK---CVSSPSKG-KTSCDKNKL---------------  43
Myotis lucifugus FGF1C          8 --SL-I--RQKRQARE--R-EK--SNACK---CVSSPSKG-KTSCDKNKL---------------  43
Dasypus novemcinctus FGF1C      8 --SL-I--RQKRQARE--R-EK--SNACK---CVSSPSKG-KTSCDKNKL---------------  43
Loxodonta africana FGF1C        8 --SL-I--RQKRQARE--R-EK--SNACK---CVSSPSKG-KTSCDKNKL---------------  43
Homo sapiens FGF1D              7 --SL-I--RQKREVRE-PGGSRPVSAQ--RRVC---P-RGTKSLCQKQLL---------------  44
Pan troglodytes FGF1D           7 --SL-I--RQKREVRE-PGGSRPVSAQ--RRVC---P-RGTKSLCQKQLL---------------  44
Nomascus leucogenys FGF1D       7 --SL-I--RQKREVRE-PGGSRPVSSQ--RRVC---P-RGTKSLCQKQLL---------------  44
Macaca mulatta FGF1D            7 --SL-I--RQKREVRE-PGGSRPVSAQ--RRVC---P-RGTKSLCQKQLL---------------  44
Otolemur garnettii FGF1D        7 --SL-I--RQKREVRE-PGGSRPVSAQ--RRVC---P-RGTKSLCQKQLL---------------  44
Mus musculus Fgf1d              7 --SL-I--RQKREVRE-PGGSRPVSAQ--RRVC---P-RGTKSLCQKQLL---------------  44
Rattus norvegicus Fgf1d         7 --SL-I--RQKREVRE-PGGSRPVSAQ--RRVC---P-RGTKSLCQKQLL---------------  44
Cavia porcellus FGF1D           7 --SL-I--RQKREVRE-PGGSRPVSAQ--RRVC---P-RGTKSLCQKQLL---------------  44
Oryctolagus cuniculus FGF1D     7 --SL-I--RQKREVRE-PGGSRPVSAQ--RRVC---P-RGTKSLCQKQLL---------------  44
Bos taurus FGF1D                7 --SL-I--RQKREVRE-PGGSRPVSAQ--RRVC---P-RGTKSLCQKQLL---------------  44
Equus caballus FGF1D            7 --SL-I--RQKREVRE-PGGSRPVSAQ--RRVC---P-RGTKSLCQKQLL---------------  44
Canis lupus familiaris FGF1D    7 --SL-I--RQKREVRE-PGGSRPVSAQ--RRVC---P-RGTKSLCQKQLL---------------  44
Pteropus vampyrus FGF1D         7 --SL-I--RQKREVRE-PGGSRPVSAQ--RRVC---P-RGTKSLCQKQLL---------------  44
Dasypus novemcinctus FGF1D      7 --SL-I--RQKREVRE-PGGSRPVTAQ--RRVC---P-RGTKSLCQKQLL---------------  44
Loxodonta africana FGF1D        7 --SL-I--RQKREVRE-PGSSRPVSAQ--RRVC---P-RGTKSLCQKQLL---------------  44
Homo sapiens FGF2A              - -----------------------------------------------------------------   -
Pongo abelii FGF2A              - -----------------------------------------------------------------   -
Nomascus leucogenys FGF2A       - -----------------------------------------------------------------   -
Macaca mulatta FGF2A            - -----------------------------------------------------------------   -
Mus musculus Fgf2a              - -----------------------------------------------------------------   -
Rattus norvegicus Fgf2a         - -----------------------------------------------------------------   -
Oryctolagus cuniculus FGF2A     - -----------------------------------------------------------------   -
Dasypus novemcinctus FGF2A      - -----------------------------------------------------------------   -
Homo sapiens FGF2B              - -----------------------------------------------------------------   -
Pan troglodytes FGF2B           - -----------------------------------------------------------------   -
Pongo abelii FGF2B              - -----------------------------------------------------------------   -
Nomascus leucogenys FGF2B       - -----------------------------------------------------------------   -
Callithrix jacchus FGF2B        - -----------------------------------------------------------------   -
Tarsius syrichta FGF2B          - -----------------------------------------------------------------   -
Otolemur garnettii FGF2B        - -----------------------------------------------------------------   -
Mus musculus Fgf2b              - -----------------------------------------------------------------   -
Rattus norvegicus Fgf2b         - -----------------------------------------------------------------   -
Cavia porcellus FGF2B           - -----------------------------------------------------------------   -
Oryctolagus cuniculus FGF2B     - -----------------------------------------------------------------   -
Tursiops truncatus FGF2B        - -----------------------------------------------------------------   -
Bos taurus FGF2B                - -----------------------------------------------------------------   -
Equus caballus FGF2B            - -----------------------------------------------------------------   -
Canis lupus familiaris FGF2B    - -----------------------------------------------------------------   -
Felis catus FGF2B               - -----------------------------------------------------------------   -
Myotis lucifugus FGF2B          - -----------------------------------------------------------------   -
Pteropus vampyrus FGF2B         - -----------------------------------------------------------------   -
Dasypus novemcinctus FGF2B      - -----------------------------------------------------------------   -
Loxodonta africana FGF2B        - -----------------------------------------------------------------   -
Homo sapiens FGF3A             23 EKRL-A-P--KGQ-P-GPAA-T-D-----RN-----PR-GSSSR-QSSSSA--M-SSSSASSS--  62
Pan troglodytes FGF3A          23 EKRL-A-P--KGQ-P-GPAA-T-D-----RN-----PR-GSSSR-QSSSSA--M-SSSSASSS--  62
Nomascus leucogenys FGF3A      22 EKRL-A-P--KGQ-P-GPAA-T-D-----RN-----PR-GSSSR-QSSSSA--M-SSSSASSS--  61
```

Macaca mulatta FGF3A	23	EKRL-V-P--KGQ-L-GPAA-T-D-----RN-----PR-GSSSR-QSSSSA--M-SSSSASSS--	62
Papio hamadryas FGF3A	23	EKRL-V-P--KGQ-L-GPAA-T-D-----RN-----PR-GSSSR-QSSSSA--M-SSSSASSS--	62
Callithrix jacchus FGF3A	23	EKRL-A-P--KGQ-S-GPAA-S-D-----RN-----PR-GSSSR-QSGSSA--M-SSSSASSS--	62
Otolemur garnettii FGF3A	23	EKRL-A-P--RGQ-P-GPAA-P-D-----TN-----PG-GSSSR-RSSGGT-TS-SSSSASSS--	63
Mus musculus Fgf3a	23	EKRL-T-P--EGQ-P----A-P-P-----RN-----PG-DSSGS-RGRSSA-TF-SSSSASSP--	60
Rattus norvegicus Fgf3a	23	EKRL-T-P--EGQ-P----A-P-P-----RN-----PG-DSSGS-RGRSSA-TF-ASSSASSP--	60
Dipodomys ordii FGF3A	23	EKRL-A-P--KGQ-L-GSAV-T-D-----KN-----PG-DSGNR-GNSSSTRWS-SPSSASSS-S	65
Cavia porcellus FGF3A	23	EKLV-A-L--KGQ-P-GSAA-A-A-----RN-----PG-DSSSS-RSGSSA-TS-SASSAPSS--	63
Oryctolagus cuniculus FGF3A	23	EKRL-A-P--KGQ-P-GPAA-A-D-----RN-----LG-GSSSS-RSAGST----SSSSASSA--	61
Bos taurus FGF3A	23	EKRL-A-P--KGQ-P-GPAA-T-E-----RN-----PG-GASSR-RSSSSTATS-SSSPASSS--	64
Equus caballus FGF3A	22	EKRL-A-P--KGQ-P-GPVA-T-D-----RN-----PR-GASSS-RSGSSTTSS-SSSSASSS--	63
Canis lupus familiaris FGF3A	23	EKHL-A-P--KGQ-P-GPAA-T-G-----RN-----PG-GAGGS-STSTSGGTT-SSSSSSVS-S	65
Myotis lucifugus FGF3A	22	EKRL-A-P--KGQ-L-GPAA-T-E-----KI-----PG-GSRSR-RNSSST----TSSSASS---	59
Dasypus novemcinctus FGF3A	23	EKRL-A-P--KGQ-P-GPAV-T-G-----RN----------SSR-SSSRST----TSSTAFSS--	57
Homo sapiens FGF4A	23	SHFL-L-PPAG-ERPPLL-GER------R-----SAAE---------------------------	45
Pan troglodytes FGF4A	23	SHFL-L-PPAG-ERPPLL-GER------R-----SAAE---------------------------	45
Nomascus leucogenys FGF4A	23	SHFL-L-PPAR-ERPPLL-GER------R-----GAAE---------------------------	45
Macaca mulatta FGF4A	23	SHFL-L-PPAG-ERPPLL-GER------R-----GAAE---------------------------	45
Papio hamadryas FGF4A	23	SHFL-L-PPAG-ERPPLL-GER------R-----GAAE---------------------------	45
Callithrix jacchus FGF4A	23	SHFL-L-PLAG-EQPPLL-SER------K-----SAAE---------------------------	45
Mus musculus Fgf4a	23	SHFL-L-PPAG-ERPPLL-GER------R-----GALE---------------------------	45
Rattus norvegicus Fgf4a	23	SHFL-L-PPAG-ERPPLL-GER------R-----GALE---------------------------	45
Bos taurus FGF4A	23	SHFL-L-PPAG-ERPPLL-GER------R-----SAAE---------------------------	45
Vicugna pacos FGF4A	23	SHFL-L-PPAG-ERPPLL-GER------R-----GAAE---------------------------	45
Pteropus vampyrus FGF4A	23	SHFL-L-PPAG-ERPPLL-GER------L-----GAAE---------------------------	45
Homo sapiens FGF4B	21	NVPV-L-P-V--DSPVLL-SDH------L-----GQSE---------------------------	41
Pan troglodytes FGF4B	21	NVPV-L-P-V--DSPVLL-SDH------L-----GQSE---------------------------	41
Gorilla gorilla FGF4B	21	NVPV-L-P-V--DSPVLL-SDH------L-----GQSE---------------------------	41
Nomascus leucogenys FGF4B	21	NVPV-L-P-V--DSPVLL-SDH------L-----GQSE---------------------------	41
Callithrix jacchus FGF4B	21	NVPV-L-P-V--ESPVLL-SDH------L-----SQSD---------------------------	41
Otolemur garnettii FGF4B	21	NVPV-L-P-V--DSPVLL-SDH------L-----GQSE---------------------------	41
Mus musculus Fgf4b	21	NVPV-L-P-V--DSPVLL-SDH------L-----GQSE---------------------------	41
Rattus norvegicus Fgf4b	21	NVPV-L-P-V--DSPVLL-SDH------L-----GQSE---------------------------	41
Oryctolagus cuniculus FGF4B	21	NVPV-L-P-V--DSPVLL-SDH------L-----GQSE---------------------------	41
Tursiops truncatus FGF4B	21	NVPV-L-P-A--DSPVLL-SDH------L-----GQSE---------------------------	41
Equus caballus FGF4B	21	NVPV-L-P-V--DSPVLL-SDH------L-----GQSE---------------------------	41
Canis lupus familiaris FGF4B	21	NVPV-L-P-V--DSPVLL-SDH------L-----GQSE---------------------------	41
Myotis lucifugus FGF4B	21	NVPV-L-P-V--DSPVLL-SDH------L-----GQSE---------------------------	41
Pteropus vampyrus FGF4B	21	NVPV-L-P-V--DSPVLL-SDH------L-----GQSE---------------------------	41
Dasypus novemcinctus FGF4B	21	NVPV-L-P-V--DSPVLL-SDH------L-----GQSE---------------------------	41
Loxodonta africana FGF4B	21	NVPV-L-P-A--DSPVLL-SDH------L-----GQSE---------------------------	41
Homo sapiens FGF4C	21	SLGN-V-PLA--DSPGFL-NER------L-----GQIE---------------------------	42
Pan troglodytes FGF4C	21	SLGN-V-PLA--DSPGFL-NER------L-----GQIE---------------------------	42
Gorilla gorilla FGF4C	21	SLGN-V-PLA--DSPGFL-NER------L-----GQIE---------------------------	42
Pongo abelii FGF4C	21	SLGN-V-PLA--DSPGFL-NER------L-----GQIE---------------------------	42
Nomascus leucogenys FGF4C	21	SLGN-V-PLA--DSPGFL-NER------L-----GQIE---------------------------	42
Otolemur garnettii FGF4C	21	SLGN-V-PLA--DSPGFL-NER------P-----DQIE---------------------------	42
Tupaia belangeri FGF4C	21	SLGN-V-PLA--DSPGFL-NER------L-----GQIE---------------------------	42
Mus musculus Fgf4c	21	SLGN-V-PLA--DSPGFL-NER------L-----GQIE---------------------------	42
Cavia porcellus FGF4C	21	SLGN-V-PLA--DSPGFL-NER------L-----GQIE---------------------------	42
Bos taurus FGF4C	21	SLGN-V-PLA--DSPGFL-NER------L-----GQIE---------------------------	42
Canis lupus familiaris FGF4C	21	SVGN-V-PIA--DSSGFL-NER------L-----GQIE---------------------------	42
Myotis lucifugus FGF4C	21	SLGN-M-PLA--DSPGFL-NER------L-----GQIE---------------------------	42
Pteropus vampyrus FGF4C	21	SLGS-M-PLA--DSPGFL-NER------L-----GQIE---------------------------	42
Dasypus novemcinctus FGF4C	21	SLGN-V-PLA--DSPGFL-NER------L-----GQIE---------------------------	42
Homo sapiens FGF5A	32	SVPV-TCQALGQD--M----V--------------S-PEATNSS-----------------SSSFS	58
Pan troglodytes FGF5A	32	SVPV-TCQALGQD--M----V--------------S-PEATNSS-----------------SSSFS	58
Nomascus leucogenys FGF5A	32	SVPV-TCQALGQD--T----V--------------S-PEATNSS-----------------SSSFS	58
Macaca mulatta FGF5A	32	CVPV-TCQALGQD--M----V--------------S-PETTNSS-----------------SSSFS	58
Callithrix jacchus FGF5A	33	SIPV-TCQALGQD--M----V--------------S-PEATNSS-----------------SSSSS	59
Otolemur garnettii FGF5A	32	SVPA-TCQALGQD--M----V--------------S-PEATNSS-----------------SSSFS	58
Tupaia belangeri FGF5A	32	SVPV-TCQALGQD--M----V--------------S-PEATNS------------------SSSFS	57
Mus musculus Fgf5a	82	SFPV-TCQALGQD--M----V--------------S-QEATNCSS-S--------------SSSFS	110
Rattus norvegicus Fgf5a	31	SVPV-TCQALGQD--M----V--------------S-PEATNSSS-SSSSSSS--------SSSFS	65
Cavia porcellus FGF5A	31	SVPV-TCQALGQD--M----V--------------S-PEATNSSS-SSSSSSSS-------SSSFS	66
Oryctolagus cuniculus FGF5A	32	SVPV-TCQALGQD--M----V--------------S-PEATNSSS-SSSSSSSSSSS----SSSFS	70
Myotis lucifugus FGF5A	32	SVPA-TCQALGQD--M----V--------------P-PEATNSSS-SSSSFSSSSSSSSSSSSSFS	74
Dasypus novemcinctus FGF5A	31	SVPV-TCQALGQD--M----V--------------S-PEATNSSS-SSSSSS---------SSSFS	64
Loxodonta africana FGF5A	31	SVPV-TCQALGQD--M----V--------------S-PEATNSSS-SSSSS----------SSSFS	63
Homo sapiens FGF5B	27	TISL-ACNDMTPEQ-MAT-NV---------N-C-SSPE---------------------------	50
Nomascus leucogenys FGF5B	27	TISL-ACNDMTPEQ-MAT-NV---------N-C-SSPE---------------------------	50
Macaca mulatta FGF5B	27	TISL-ACNDMTPEQ-MAT-NV---------N-C-SSPE---------------------------	50
Callithrix jacchus FGF5B	27	TISL-ACNDMTPEQ-MAT-NV---------N-C-SSPE---------------------------	50
Mus musculus Fgf5b	27	TISL-ACNDMSPEQ-TAT-SV---------N-C-SSPE---------------------------	50
Rattus norvegicus Fgf5b	27	TISL-ACNDMSPEQ-TAT-SV---------N-C-SSPE---------------------------	50
Cavia porcellus FGF5B	27	TLAL-ACNDMAPEQ-MAT-NA---------N-C-SSPE---------------------------	50
Oryctolagus cuniculus FGF5B	27	TISL-ACNDMTPEQ-MAA-NV---------N-C-SSPE---------------------------	50
Bos taurus FGF5B	27	TISL-ACNDMTPEQ-MAT-NV---------N-C-SSPE---------------------------	50
Vicugna pacos FGF5B	27	TISL-ACNDMTPEQ-MAT-NV---------N-C-SSPE---------------------------	50

Equus caballus FGF5B	27	TISL-ACNDMTPEQ-MAT-NV---------N-C-SSPE---------------------------	50
Canis lupus familiaris FGF5B	27	TISL-ACNDMTPEQ-MAT-NV---------N-C-SSPE---------------------------	50
Myotis lucifugus FGF5B	27	TISL-ACNDMTPEQ-MAA-NV---------N-C-SSPE---------------------------	50
Sorex araneus FGF5B	27	TISL-ACNDMTPEQ-MAT-NV---------N-C-SSPE---------------------------	50
Dasypus novemcinctus FGF5B	27	TISL-ACNDMTPEQ-MAT-NV---------N-C-SSPE---------------------------	50
Loxodonta africana FGF5B	27	TASL-ACNDMTPEQ-MAA-NA---------N-C-SSPE---------------------------	50
Homo sapiens FGF5C	-	---	-
Pongo abelii FGF5C	-	---	-
Macaca mulatta FGF5C	-	---	-
Mus musculus Fgf5c	-	---	-
Rattus norvegicus Fgf5c	-	---	-
Cavia porcellus FGF5C	-	---	-
Bos taurus FGF5C	-	---	-
Homo sapiens FGF5D	-	---	-
Macaca mulatta FGF5D	-	---	-
Papio hamadryas FGF5D	-	---	-
Otolemur garnettii FGF5D	-	---	-
Mus musculus Fgf5d	-	---	-
Rattus norvegicus Fgf5d	-	---	-
Bos taurus FGF5D	-	---	-
Myotis lucifugus FGF5D	-	---	-
Pteropus vampyrus FGF5D	-	---	-
Homo sapiens FGF6A	25	-------LVA-EENVDF-----------R-----IHVE---------------------------	38
Mus musculus Fgf6a	25	-------LAA-EENVDF-----------R-----IHVE---------------------------	38
Cavia porcellus FGF6A	25	-------LAA-EENVDF-----------R-----IHVE---------------------------	38
Bos taurus FGF6A	25	-------LAA-EENVDF-----------R-----IHVE---------------------------	38
Canis lupus familiaris FGF6A	25	-------LAA-EENVDF-----------R-----IHVE---------------------------	38
Homo sapiens FGF6B	34	-------PKQ-IDTI-FP-LVT-A----K-----GENH--------------------------P	52
Pan troglodytes FGF6B	34	-------PKQ-IDTI-FP-LVT-A----K-----GENH--------------------------P	52
Nomascus leucogenys FGF6B	34	-------PKQ-IDTI-FP-LVT-A----K-----GENH--------------------------P	52
Macaca mulatta FGF6B	128	-------PKQ-IDTI-FP-LVT-A----K-----GENH--------------------------P	146
Mus musculus Fgf6b	71	-------PKQ-IDTI-FP-LVT-A----K-----GENH--------------------------P	89
Rattus norvegicus Fgf6b	70	-------PKQ-IDTI-FP-LVT-A----K-----GENH--------------------------P	88
Cavia porcellus FGF6B	73	-------PKQ-IDTI-FP-LVT-A----K-----GENH--------------------------P	91
Canis lupus familiaris FGF6B	38	-------PKQ-IDTI-FP-LVT-A----K-----GENH--------------------------P	56
Felis catus FGF6B	80	-------PKQ-IDTI-FP-LVT-A----K-----GENH--------------------------P	98
Myotis lucifugus FGF6B	8	-------PKQ-IDTI-FP-LVT-A----K-----GENH--------------------------P	26
Dasypus novemcinctus FGF6B	-	---	-
Loxodonta africana FGF6B	27	-------PKQ-IDTI-FP-LVT-A----K-----GENH--------------------------P	45
Homo sapiens FGF6C	134	--------------V-F--FNF-L----K-----VTVQ--------------------------S	145
Nomascus leucogenys FGF6C	5	--------------V-F--FNF-L----K-----VTVQ--------------------------S	16
Macaca mulatta FGF6C	5	--------------V-F--FNF-L----K-----VTVQ--------------------------S	16
Papio hamadryas FGF6C	5	--------------V-F--FNF-L----K-----VTVQ--------------------------S	16
Mus musculus Fgf6c	5	--------------V-F--FNF-L----K-----VTVQ--------------------------S	16
Rattus norvegicus Fgf6c	5	--------------V-F--FNF-L----K-----VTVQ--------------------------S	16
Bos taurus FGF6C	38	--------------V-F--FNF-L----K-----VTVQ--------------------------S	49
Homo sapiens FGF7A	28	-GAA-A-P----TAPNGT-LEA-EL-E--RR---WESLV---AL-SLARL---------------	58
Macaca mulatta FGF7A	28	-GAA-A-P----TAPNGT-LEA-EL-E--RR---WESLV---AR-SLARL---------------	58
Callithrix jacchus FGF7A	28	-GAA-A-P----TAPNGT-LEA-EL-E--RS---WENLL---AR-SLKRL---------------	58
Mus musculus Fgf7a	28	--TA-A-P-------NGT-RHA-EL-G--HG---WDGLV---AR-SLARL---------------	54
Rattus norvegicus Fgf7a	28	--TA-A-P-------NGT-RHA-EL-G--HG---WDGLV---AR-SLARL---------------	54
Cavia porcellus FGF7A	28	-GAA-A-P----TAPNDT-LEA-EL-E--RR---WESLV---AR-SLARL---------------	58
Canis lupus familiaris FGF7A	28	-GAA-A-P----TAPNGT-LGA-EL-E--RR---WESLV---VR-SLARL---------------	58
Procavia capensis FGF7A	20	-G-A-A-P----SAPNGT-LEA-EL-E--LR---WDSLV---AR-SLARL---------------	49
Homo sapiens FGF7B	34	MVVP-S-P--AGTRANNT-L-L-DS----RG---WGTLL---SR-SRAGL---------------	65
Pan troglodytes FGF7B	34	MVVP-S-P--AGTRANNT-L-L-DS----RG---WGTLL---SR-SRAGL---------------	65
Pongo abelii FGF7B	34	MVVP-S-P--AGTRANNT-L-L-DS----RG---WGTLL---SR-SRAGL---------------	65
Nomascus leucogenys FGF7B	34	MVVP-L-P--AGTRANNT-L-L-DS----RG---WGTLL---SR-SRAGL---------------	65
Macaca mulatta FGF7B	34	MVVP-S-P--AGTRANNT-L-L-DS----RG---WGTLL---SR-SRAGL---------------	65
Papio hamadryas FGF7B	34	MVVP-S-P--AGTRANNT-L-L-DS----RG---WGTLL---SR-SRAGL---------------	65
Callithrix jacchus FGF7B	34	MVVS-L-P--AGSRANNT-Q-L-DS----RG---WGTLL---SR-SHTGL---------------	65
Mus musculus Fgf7b	34	MVVP-S-P--AGARANGT-L-L-DS----RG---WGTLL---SR-SRAGL---------------	65
Rattus norvegicus Fgf7b	34	MVVP-S-P--AGARANGT-L-L-DS----RG---WGTLL---SR-SRAGL---------------	65
Cavia porcellus FGF7B	34	MVVP-L-P--AGTRANST-L-L-DS----RD---WSKLL---SR-SRARL---------------	65
Oryctolagus cuniculus FGF7B	34	MVVP-S-P--AGTRANST-L-L-AS----RG---WGALL---SR-SRAGL---------------	65
Tursiops truncatus FGF7B	34	MVVP-S-P--AGSRANST-L-L-AS----RG---WGTLL---SR-SRAGL---------------	65
Bos taurus FGF7B	34	MVVP-S-P--AGTRANGT-L-L-AS----RG---WGTLL---SR-SRAGL---------------	65
Equus caballus FGF7B	34	MVVP-S-P--AGTRANST-L-L-DS----RG---WGTLL---SR-SRAGL---------------	65
Canis lupus familiaris FGF7B	34	MVVP-L-P--AGTHANGT-L-L-DS----RG---WGTLL---SR-SRAGL---------------	65
Myotis lucifugus FGF7B	34	MVVP-S-P--AGTRANST-L-L-DS----RG---WGTLL---SR-SRAGL---------------	65
Loxodonta africana FGF7B	34	MVVP-S-P--AGTRANST-L-L-DS----RG---WGTLL---SR-SRAGL---------------	65
Homo sapiens FGF8A	23	-RPL-A---	26
Pongo abelii FGF8A	23	-RPL-A---	26
Macaca mulatta FGF8A	23	-RPL-A---	26
Microcebus murinus FGF8A	104	-RPL-A---	107
Otolemur garnettii FGF8A	26	-RPL-A---	29
Mus musculus Fgf8a	26	-RPL-A---	29
Rattus norvegicus Fgf8a	26	-RPL-V---	29
Cavia porcellus FGF8A	26	-RPL-A---	29

```
Bos taurus FGF8A                 26 -RPL-A------------------------------------------------------------  29
Myotis lucifugus FGF8A           26 -GPL-G------------------------------------------------------------  29
Pteropus vampyrus FGF8A          23 -GPL-A------------------------------------------------------------  26
Dasypus novemcinctus FGF8A       26 -RPL-A------------------------------------------------------------  29
Homo sapiens FGF8B              150 -L---------------------------------------------------------------- 150
Pan troglodytes FGF8B           150 -L---------------------------------------------------------------- 150
Nomascus leucogenys FGF8B       150 -L---------------------------------------------------------------- 150
Otolemur garnettii FGF8B         22 -V----------------------------------------------------------------  22
Mus musculus Fgf8b               22 -A----------------------------------------------------------------  22
Rattus norvegicus Fgf8b          95 -A----------------------------------------------------------------  95
Cavia porcellus FGF8B            17 -G----------------------------------------------------------------  17
Oryctolagus cuniculus FGF8B      83 -V----------------------------------------------------------------  83
Equus caballus FGF8B             17 -V----------------------------------------------------------------  17
Canis lupus familiaris FGF8B     84 -V----------------------------------------------------------------  84
Dasypus novemcinctus FGF8B       19 -P----------------------------------------------------------------  19
Loxodonta africana FGF8B         81 -V----------------------------------------------------------------  81
Homo sapiens FGF8C               21 -LLLGAC-----------------------------------------------------------  26
Pan troglodytes FGF8C            21 -LLLGAC-----------------------------------------------------------  26
Pongo abelii FGF8C               21 -LLLGAC-----------------------------------------------------------  26
Nomascus leucogenys FGF8C        21 -LLLGAC-----------------------------------------------------------  26
Otolemur garnettii FGF8C         23 -LLLGAC-----------------------------------------------------------  28
Mus musculus Fgf8c               22 -FLLGVY-----------------------------------------------------------  27
Rattus norvegicus Fgf8c          22 -FLLGVC-----------------------------------------------------------  27
Cavia porcellus FGF8C            27 -LLVGAC-----------------------------------------------------------  32
Oryctolagus cuniculus FGF8C      21 -LLLGAC-----------------------------------------------------------  26
Ochotona princeps FGF8C          17 -LLLGVG-----------------------------------------------------------  22
Bos taurus FGF8C                 21 -LLLGTC-----------------------------------------------------------  26
Equus caballus FGF8C             21 -LLLGAC-----------------------------------------------------------  26
Canis lupus familiaris FGF8C     21 -LLLEAC-----------------------------------------------------------  26
Felis catus FGF8C                22 -LLPEAC-----------------------------------------------------------  27
Sorex araneus FGF8C              21 -LLLGAC-----------------------------------------------------------  26
Procavia capensis FGF8C          21 -LLLGAC-----------------------------------------------------------  26
```

```
Homo sapiens FGF1A              47 --GVFSK-VRFC--------SGRKRPV--RRRPEPQLKGIVTR-LF-SQQ-GY-----FLQMHPD  90
                                            270       280       290       300       310       320
Homo sapiens FGF1A              47 --GVFSK-VRFC--------SGRKRPV--RRRPEPQLKGIVTR-LF-SQQ-GY-----FLQMHPD  90
Pan troglodytes FGF1A           47 --GVFSK-VRFC--------SGRKRPV--RRRPEPQLKGIVTR-LF-SQQ-GY-----FLQMHPD  90
Nomascus leucogenys FGF1A       47 --GVFSK-VRFC--------SGRKRPV--RRRPEPQLKGIVTR-LF-SQQ-GY-----FLQMHPD  90
Callithrix jacchus FGF1A        47 --GVFSK-VRFC--------SGRKRPV--RRRPEPQLKGIVTR-LF-SQQ-GY-----FLQMHPD  90
Mus musculus Fgf1a              47 --GVFSK-VRFC--------SGRKRPV--RRRPEPQLKGIVTR-LF-SQQ-GY-----FLQMHPD  90
Rattus norvegicus Fgf1a         47 --GVFSK-VRFC--------SGRKRPV--RRRPEPQLKGIVTR-LF-SQQ-GY-----FLQMHPD  90
Cavia porcellus FGF1A           47 --GVFSK-VRFC--------SGRKRPV--RRRPEPQLKGIVTR-LF-SQQ-GY-----FLQMHPD  90
Oryctolagus cuniculus FGF1A     47 --GVFSK-VRFC--------SGRKRPV--RRRPEPQLKGIVTR-LF-SQQ-GY-----FLQMHPD  90
Equus caballus FGF1A            47 --GVFSK-VRFC--------SGRKRPV--RRRPEPQLKGIVTR-LF-SQQ-GY-----FLQMHPD  90
Canis lupus familiaris FGF1A    47 --GVFSK-VRFC--------SGRKRPV--RRRPEPQLKGIVTR-LF-SQQ-GY-----FLQMHPD  90
Dasypus novemcinctus FGF1A      47 --GVFSK-VRFC--------SGRKRPV--RRRPEPQLKGIVTR-LF-SQQ-GY-----FLQMHPD  90
Homo sapiens FGF1B              46 --DIFSK-VRI---------FGLKKRR--LRRQDPQLKGIVTR-LYC-RQ-GY-----YLQMHPD  88
Pan troglodytes FGF1B           46 --DIFSK-VRI---------FGLKKRR--LRRQDPQLKGIVTR-LYC-RQ-GY-----YLQMHPD  88
Pongo abelii FGF1B              46 --DIFSK-VRI---------FGLKKRR--LRRQDPQLKGIVTR-LYC-RQ-GY-----YLQMHPD  88
Macaca mulatta FGF1B            46 --DIFSK-VRI---------FGLKKRR--LRRQDPQLKGIVTR-LYC-RQ-GY-----YLQMHPD  88
Callithrix jacchus FGF1B        46 --DIFSK-VRI---------FGLKKRR--LRRQDPQLKGIVTR-LYC-RQ-GY-----YLQMHPD  88
Mus musculus Fgf1b              46 --DIFSK-VRI---------FGLKKRR--LRRQDPQLKGIVTR-LYC-RQ-GY-----YLQMHPD  88
Oryctolagus cuniculus FGF1B     46 --DIFSK-VRI---------FGLKKRR--LRRQDPQLKGIVTR-LYC-RQ-GY-----YLQMHPD  88
Equus caballus FGF1B            46 --DIFSK-VRI---------FGLKKRR--LRRQDPQLKGIVTR-LYC-RQ-GY-----YLQMHPD  88
Dasypus novemcinctus FGF1B      46 --DIFSK-VRI---------FGLKKRR--LRRQDPQLKGIVTR-LYC-RQ-GY-----YLQMHPD  88
Homo sapiens FGF1C              44 --NVFSR-VKL---------FGSKKRR--RRRPEPQLKGIVTK-LY-SRQ-GY-----HLQLQAD  86
Pan troglodytes FGF1C           44 --NVFSR-VKL---------FGSKKRR--RRRPEPQLKGIVTK-LY-SRQ-GY-----HLQLQAD  86
Pongo abelii FGF1C              44 --NVFSR-VKL---------FGSKKRR--RRRPEPQLKGIVTK-LY-SRQ-GY-----HLQLQAD  86
Macaca mulatta FGF1C            44 --NVFSR-VKL---------FGSKKRR--RRRPEPQLKGIVTK-LY-SRQ-GY-----HLQLQAD  86
Mus musculus Fgf1c              44 --NVFSR-VKL---------FGSKKRR--RRRPEPQLKGIVTK-LY-SRQ-GY-----HLQLQAD  86
Rattus norvegicus Fgf1c         44 --NVFSR-VKL---------FGSKKRR--RRRPEPQLKGIVTK-LY-SRQ-GY-----HLQLQAD  86
Cavia porcellus FGF1C           44 --NVFSR-VKL---------FGSKKRR--RRRPEPQLKGIVTK-LY-SRQ-GF-----HLQLQAD  86
Bos taurus FGF1C                44 --NVFSR-VKL---------FGSKKRR--RRRPEPQLKGIVTK-LY-SRQ-GY-----HLQLQAD  86
Myotis lucifugus FGF1C          44 --NVFSR-VKL---------FGSKKRR--RRRPEPQLKGIVTK-LY-SRQ-GF-----HLQLQAD  86
Dasypus novemcinctus FGF1C      44 --NVFSR-VKL---------FGSKKRR--RRRPEPQLKGIVTK-LY-SRQ-GF-----HLQLQAD  86
Loxodonta africana FGF1C        44 --NVFSR-VKL---------FGSKKRR--RRRPEPQLKGIVTK-LY-SRQ-GY-----HLQLQAD  86
Homo sapiens FGF1D              45 --ILLSK-VRLC--------GGRPARP--DRGPEPQLKGIVTK-LFC-RQ-GF-----YLQANPD  88
Pan troglodytes FGF1D           45 --ILLSK-VRLC--------GGRPARP--DRGPEPQLKGIVTK-LFC-RQ-GF-----YLQANPD  88
Nomascus leucogenys FGF1D       45 --ILLSK-VRLC--------GGRPARP--DRGPEPQLKGIVTK-LFC-RQ-GF-----YLQANPD  88
Macaca mulatta FGF1D            45 --ILLSK-VRLC--------GGRPARP--DRGPEPQLKGIVTK-LFC-RQ-GF-----YLQANPD  88
Otolemur garnettii FGF1D        45 --ILLSK-VRLC--------GGRPTRP--DRGPEPQLKGIVTK-LFC-RQ-GF-----YLQANPD  88
Mus musculus Fgf1d              45 --ILLSK-VRLC--------GGRPTRQ--DRGPEPQLKGIVTK-LFC-RQ-GF-----YLQANPD  88
Rattus norvegicus Fgf1d         45 --ILLSK-VRLC--------GGRPTRQ--DRGPEPQLKGIVTK-LFC-RQ-GF-----YLQANPD  88
Cavia porcellus FGF1D           45 --ILLSK-VRLC--------GGRPARP--DRGLEPQLKGIVTK-LFC-RQ-GF-----YLQANPD  88
Oryctolagus cuniculus FGF1D     45 --ILLSK-VRLC--------GGRPTRP--DRGPEPQLKGIVTK-LFC-RQ-GF-----YLQANPD  88
Bos taurus FGF1D                45 --ILLSK-VRLC--------GGRPART--DRGPEPQLKGIVTK-LFC-RQ-GF-----YLQANPD  88
Equus caballus FGF1D            45 --ILLSK-VRLC--------GGRPARP--DRGLEPQLKGIVTK-LFC-RQ-GF-----YLQANPD  88
Canis lupus familiaris FGF1D    45 --ILLSK-VRLC--------GGRPARP--DRGPEPQLKGIVTK-LFC-RQ-GF-----YLQANPD  88
Pteropus vampyrus FGF1D         45 --ILLSK-VRLC--------GGRPARP--NRGPEPQLKGIVTK-LFC-RQ-GF-----YLQANPD  88
Dasypus novemcinctus FGF1D      45 --VLLSK-VRLC--------GGRPSRP--DRGPEPQLKGIVTK-LFC-RQ-GF-----YLQANPD  88
Loxodonta africana FGF1D        45 --ILLSK-VRLC--------GGRSVRP--DRGLEPQLKGIVTK-LFC-RQ-GF-----YLQANPD  88
Homo sapiens FGF2A              18 --------SGA-FPP-----GHFKD-P---------------KRLYCKNG-GF-----FLRIHPD  46
Pongo abelii FGF2A              18 --------SGA-FPP-----GHFKD-P---------------KRLYCKNG-GF-----FLRIHPD  46
Nomascus leucogenys FGF2A       18 --------SGA-FPP-----GHFKD-P---------------KRLYCKNG-GF-----FLRIHPD  46
Macaca mulatta FGF2A            18 --------SGA-FPP-----GHFKD-P---------------KRLYCKNG-GF-----FLRIHPD  46
Mus musculus Fgf2a              18 ---------AA-FPP-----GHFKD-P---------------KRLYCKNG-GF-----FLRIHPD  45
Rattus norvegicus Fgf2a         18 ---------GA-FPP-----GHFKD-P---------------KRLYCKNG-GF-----FLRIHPD  45
Oryctolagus cuniculus FGF2A     18 --------SGA-FPP-----GHFKD-P---------------KRLYCKNG-GF-----FLRIHPD  46
Dasypus novemcinctus FGF2A      18 --------SGA-FPP-----GHFKD-P---------------KRLYCKNG-GF-----FLRIHPD  46
Homo sapiens FGF2B              16 ---------FN-LPP-----GNYKK-P---------------KLLYCSNG-GH-----FLRILPD  43
Pan troglodytes FGF2B           16 ---------FN-LPP-----GNYKK-P---------------KLLYCSNG-GH-----FLRILPD  43
Pongo abelii FGF2B              16 ---------FN-LPP-----GNYKK-P---------------KLLYCSNG-GH-----FLRILPD  43
Nomascus leucogenys FGF2B       16 ---------FN-LPP-----GNYKK-P---------------KLLYCSNG-GH-----FLRILPD  43
Callithrix jacchus FGF2B        16 ---------FD-LPP-----GNYKK-P---------------KLLYCSNG-GH-----FLRILPD  43
Tarsius syrichta FGF2B          16 ---------FN-LPP-----GNYKK-P---------------KLLYCSNG-GH-----FLRILPD  43
Otolemur garnettii FGF2B        16 ---------FN-LPL-----GNYKK-P---------------KLLYCSNG-GH-----FLRILPD  43
Mus musculus Fgf2b              16 ---------FN-LPL-----GNYKK-P---------------KLLYCSNG-GH-----FLRILPD  43
Rattus norvegicus Fgf2b         16 ---------FN-LPL-----GNYKK-P---------------KLLYCSNG-GH-----FLRILPD  43
Cavia porcellus FGF2B           16 ---------FN-LPP-----GNYKK-P---------------KLLYCSNG-GH-----FLRILPD  43
Oryctolagus cuniculus FGF2B     16 ---------FN-LPA-----GNYKL-P---------------KLLYCSNG-GH-----FLRILPD  43
Tursiops truncatus FGF2B        16 ---------FN-LPP-----GNYKK-P---------------KLLYCSNG-GH-----FLRILPD  43
Bos taurus FGF2B                16 ---------FN-LPL-----GNYKK-P---------------KLLYCSNG-GY-----FLRILPD  43
Equus caballus FGF2B            16 ---------FN-LPP-----GNYKK-P---------------KLLYCSNG-GH-----FLRILPD  43
Canis lupus familiaris FGF2B    16 ---------FN-LPP-----GNYMK-P---------------KLLYCSNG-GH-----FLRILPD  43
Felis catus FGF2B               16 ---------FN-LPP-----GNYKK-P---------------KLLYCSNG-GH-----FLRILPD  43
Myotis lucifugus FGF2B          16 ---------FN-LPL-----ENYKK-P---------------KLLYCSNG-GH-----FLRILPD  43
Pteropus vampyrus FGF2B         16 ---------FN-LPP-----GNYKK-P---------------KLLYCSNG-GH-----FLRILPD  43
Dasypus novemcinctus FGF2B      16 ---------FN-LPL-----ENYKH-P---------------RLLYCRNG-GH-----FLRILPD  43
Loxodonta africana FGF2B        16 ---------FN-LPP-----GNYKK-P---------------KLLYCSNG-GH-----FLRILPD  43
Homo sapiens FGF3A              63 -P-AASLGSQG-SGLEQS-SFQW-S-PS-GRRTG--------S-LYCRVGIGF-----HLQIYPD 106
Pan troglodytes FGF3A           63 -A-AASLGSQG-SGLEQS-SFQW-S-PS-GRRTG--------S-LYCRVGIGF-----HLQIYPD 106
Nomascus leucogenys FGF3A       62 -P-AASLGSQG-SGLEQS-SFQW-S-PS-GRRTG--------S-LYCRVGIGF-----HLQIYPD 105
```

Macaca mulatta FGF3A	63	-P-AASLGSQG-SGLEQS-SFQW-S-PS-GRRTG--------S-LYCRVGIGF-----HLQIYPD	106
Papio hamadryas FGF3A	63	-P-AASLGSQG-SGLEQS-SFQW-S-PS-GRRTG--------S-LYCRVGIGF-----HLQIYPD	106
Callithrix jacchus FGF3A	63	-P-AASLGSQG-SGLEQS-SFQW-S-PS-GRRTG--------S-LYCRVGIGF-----HLQIYPD	106
Otolemur garnettii FGF3A	64	-P-AASLGSPG-SGLEQS-SFQW-S-PS-GRRTG--------S-LYCRVGIGF-----HLQIYPD	107
Mus musculus Fgf3a	61	-V-AASPGSQG-SGSEHS-SFQW-S-PS-GRRTG--------S-LYCRVGIGF-----HLQIYPD	104
Rattus norvegicus Fgf3a	61	-V-AASPGSQG-SGSEHS-SFQW-S-PS-GRRTG--------S-LYCRVGIGF-----HLQIYPD	104
Dipodomys ordii FGF3A	66	AS-LASSGSPG-SGAEQG-SFQW-S-PS-GRRTG--------S-LYCRVGIGF-----HLQIYPD	110
Cavia porcellus FGF3A	64	-P-AASRGSQG-SGSEQG-SFQW-S-PS-GRRTG--------S-LYCRVGIGF-----HLQIYPD	107
Oryctolagus cuniculus FGF3A	62	-P-AASLGSQG-SGLEQS-SFQW-S-PS-GRRTG--------S-LYCRVGIGF-----HLQIYPD	105
Bos taurus FGF3A	65	-S-AASRGGPG-SSLEQS-SFQW-S-PS-GRRTG--------S-LYCRVGIGF-----HLQIYPD	108
Equus caballus FGF3A	64	-P-AASLGSQA-SGLEQG-SFQW-S-PS-GRRTG--------S-LYCRVGIGF-----HLQIYPD	107
Canis lupus familiaris FGF3A	66	AP-GASPGIRG-SGSEQG-SFQW-S-PS-GRRTG--------S-LYCRVGIGF-----HLQIYPD	110
Myotis lucifugus FGF3A	60	-P-ATPPGSPG-SGLEQS-SFQW-T-PS-GRRTG--------S-LYCRVGIGF-----HLQIYPD	103
Dasypus novemcinctus FGF3A	58	-P-AASLGSQG-SGLEQS-SFQW-S-PS-GRRTG--------S-LYCRVGIGF-----HLQIYPD	101
Homo sapiens FGF4A	46	----RSA--RG-GPG--AAQLAHLHG-ILRRR----------Q-LYCR-T-GF-----HLQILPD	82
Pan troglodytes FGF4A	46	----RSA--RG-GPG--AAQLAHLHG-ILRRR----------Q-LYCR-T-GF-----HLQILPD	82
Nomascus leucogenys FGF4A	46	----RSA--RG-GPG--AAELAHLHG-ILRRR----------Q-LYCR-T-GF-----HLQILPD	82
Macaca mulatta FGF4A	46	----RSA--RG-GPG--AAELAHLHG-ILRRR----------Q-LYCR-T-GF-----HLQILPD	82
Papio hamadryas FGF4A	46	----RSA--RG-GPG--AAELAHLHG-ILRRR----------Q-LYCR-T-GF-----HLQILPD	82
Callithrix jacchus FGF4A	46	----RST--RG-WQG--AAELAHLHG-ILRRR----------Q-LYCR-T-GF-----HLQILPD	82
Mus musculus Fgf4a	46	----RGA--RG-GPG--SVELAHLHG-ILRRR----------Q-LYCR-T-GF-----HLQILPD	82
Rattus norvegicus Fgf4a	46	----RGA--RG-GPG--SVELAHLHG-ILRRR----------Q-LYCR-T-GF-----HLQILPD	82
Bos taurus FGF4A	46	----RGA--RG-GPG--AAELAHLHG-FLRRR----------Q-LYCR-T-GF-----HLQILPD	82
Vicugna pacos FGF4A	46	----RGA--RG-GPG--AAELAHLHG-ILRRR----------Q-LYCR-T-GF-----HLQILPD	82
Pteropus vampyrus FGF4A	46	----RGT--RG-GPG--AAELAHLHG-ILRRR----------Q-LYCR-T-GF-----HLQILPD	82
Homo sapiens FGF4B	42	----AGGLPRG--PA--VTDLDHLKG-ILRRR----------Q-LYCR-T-GF-----HLEIFPN	79
Pan troglodytes FGF4B	42	----AGGLPRG--PA--VTDLDHLKG-ILRRR----------Q-LYCR-T-GF-----HLEIFPN	79
Gorilla gorilla FGF4B	42	----AGGLPRG--PA--VTDLDHLKG-ILRRR----------Q-LYCR-T-GF-----HLEIFPN	79
Nomascus leucogenys FGF4B	42	----AGGLPRG--PA--VTDLDHLKG-ILRRR----------Q-LYCR-T-GF-----HLEIFPN	79
Callithrix jacchus FGF4B	42	----AGGLPRG--PA--VTDLDHLKG-ILRRR----------Q-LYCR-T-GF-----HLEIFPN	79
Otolemur garnettii FGF4B	42	----AGGLPRG--PA--VTDLDHLKG-ILRRR----------Q-LYCR-T-GF-----HLEIFPN	79
Mus musculus Fgf4b	42	----AGGLPRG--PA--VTDLDHLKG-ILRRR----------Q-LYCR-T-GF-----HLEIFPN	79
Rattus norvegicus Fgf4b	42	----AGGLPRG--PA--VTDLDHLKG-ILRRR----------Q-LYCR-T-GF-----HLEIFPN	79
Oryctolagus cuniculus FGF4B	42	----AGGLPRG--PA--VTDLDHLKG-ILRRR----------Q-LYCR-T-GF-----HLEIFPN	79
Tursiops truncatus FGF4B	42	----AGGLPRG--PA--VTDLDHLKG-ILRRR----------Q-LYCR-T-GF-----HLEIFPN	79
Equus caballus FGF4B	42	----AGGLPRG--PA--VTDLDHLKG-ILRRR----------Q-LYCR-T-GF-----HLEIFPN	79
Canis lupus familiaris FGF4B	42	----AGGLPRG--PA--VTDLDHLKG-ILRRR----------Q-LYCR-T-GF-----HLEIFPN	79
Myotis lucifugus FGF4B	42	----AGGLPRG--PA--VTDLDHLKG-ILRRR----------Q-LYCR-T-GF-----HLEIFPN	79
Pteropus vampyrus FGF4B	42	----AGGLPRG--PA--VTDLDHLKG-ILRRR----------Q-LYCR-T-GF-----HLEIFPN	79
Dasypus novemcinctus FGF4B	42	----AGGLPRG--PA--VTDLDHLKG-ILRRR----------Q-LYCR-T-GF-----HLEIFPN	79
Loxodonta africana FGF4B	42	----AGGLPRG--PA--VTDLDHLKG-ILRRR----------Q-LYCR-T-GF-----HLEIFPN	79
Homo sapiens FGF4C	43	-----GKLQRG-SP----TDFAHLKG-ILRRR----------Q-LYCR-T-GF-----HLEIFPN	78
Pan troglodytes FGF4C	43	-----GKLQRG-SP----TDFAHLKG-ILRRR----------Q-LYCR-T-GF-----HLEIFPN	78
Gorilla gorilla FGF4C	43	-----GKLQRG-SP----TDFAHLKG-ILRRR----------Q-LYCR-T-GF-----HLEIFPN	78
Pongo abelii FGF4C	43	-----GKLQRG-SP----TDFAHLKG-ILRRR----------Q-LYCR-T-GF-----HLEIFPN	78
Nomascus leucogenys FGF4C	43	-----GKLQRG-SP----TDFAHLKG-ILRRR----------Q-LYCR-T-GF-----HLEIFPN	78
Otolemur garnettii FGF4C	43	-----GKLQRG-TP----IDFAHLKG-ILRRR----------Q-LYCR-T-GF-----HLEIFPN	78
Tupaia belangeri FGF4C	43	-----GKLQRG-SP----TDFAHLKG-ILRRR----------Q-LYCR-T-GF-----HLEIFPN	78
Mus musculus Fgf4c	43	-----GKLQRG-SP----TDFAHLKG-ILRRR----------Q-LYCR-T-GF-----HLEIFPN	78
Cavia porcellus FGF4C	43	-----GKLQRG-SP----TDFAHLKG-ILRRR----------Q-LYCR-T-GF-----HLEIFPN	78
Bos taurus FGF4C	43	-----GKLQRG-SP----TDFAHLKG-ILRRR----------Q-LYCR-T-GF-----HLEIFPN	78
Canis lupus familiaris FGF4C	43	-----GKLQRG-SP----TDFAHLKG-ILRRR----------Q-LYCR-T-GF-----HLEIFPN	78
Myotis lucifugus FGF4C	43	-----GKLQRG-SP----TDFAHLKG-ILRRR----------Q-LYCR-T-GF-----HLEIFPN	78
Pteropus vampyrus FGF4C	43	-----GKLQRG-SP----TDFAHLKG-ILRRR----------Q-LYCR-T-GF-----HLEIFPN	78
Dasypus novemcinctus FGF4C	43	-----GKLQRG-SP----TDFAHLKG-ILRRR----------Q-LYCR-T-GF-----HLEIFPN	78
Homo sapiens FGF5A	59	SPSSAGRHVR--S-------YNHLQG-DVRWR----------K-LF-SFT-KY-----FLKIEKN	95
Pan troglodytes FGF5A	59	SPSSAGRHVR--S-------YNHLQG-DVRWR----------K-LF-SFT-KY-----FLKIEKN	95
Nomascus leucogenys FGF5A	59	SPSSAGRHVR--S-------YNHLQG-DVRWR----------K-LF-SFT-KY-----FLKIEKN	95
Macaca mulatta FGF5A	59	SPSSAGRHVR--S-------YNHLQG-DVRWR----------K-LF-SFT-KY-----FLKIEKN	95
Callithrix jacchus FGF5A	60	SPSSAGRHVR--S-------YNHLQG-DVRWR----------K-LF-SFT-KY-----FLKIEKN	96
Otolemur garnettii FGF5A	59	SPSSAGRHVR--S-------YNHLQG-DVRWR----------K-LF-SFT-KY-----FLKIEKN	95
Tupaia belangeri FGF5A	58	PPSSAGRHVR--S-------YNHLQG-DVRWR----------K-LF-SFT-KY-----FLKIEKN	94
Mus musculus Fgf5a	111	SPSSAGRHVR--S-------YNHLQG-DVRWR----------R-LF-SFT-KY-----FLTIEKN	147
Rattus norvegicus Fgf5a	66	SPSSAGRHVR--S-------YNHLQG-DVRWR----------K-LF-SFT-KY-----FLKIEKN	102
Cavia porcellus FGF5A	67	SPSSAGRHVR--S-------YNHLQG-DVRWR----------K-LF-SFT-KY-----FLKIEKN	103
Oryctolagus cuniculus FGF5A	71	SPSSAGRHVR--S-------YNHLQG-DVRWR----------K-LF-SFT-KY-----FLKIEKN	107
Myotis lucifugus FGF5A	75	SPPSAGRHVR--S-------YNHLQG-DVRWR----------K-LF-SFT-KY-----FLKIEKN	111
Dasypus novemcinctus FGF5A	65	PPSSAGRHVR--S-------YNHLQG-DVRWR----------K-LF-SFT-KY-----FLKIEKN	101
Loxodonta africana FGF5A	64	SPSSAGRHVR--S-------YNHLQG-DVRWR----------K-LF-SFT-KY-----FLKIEKN	100
Homo sapiens FGF5B	51	------RHTR--S-------YDYMEGGDIRVR----------R-LFCR-T-QW-----YLRIDKR	82
Nomascus leucogenys FGF5B	51	------RHTR--S-------YDYMEGGDIRVR----------R-LFCR-T-QW-----YLRIDKR	82
Macaca mulatta FGF5B	51	------RHTR--S-------YDYMEGGDIRVR----------R-LFCR-T-QW-----YLRIDKR	82
Callithrix jacchus FGF5B	51	------RHTR--S-------YDYMEGGDIRVR----------R-LFCR-T-QW-----YLRIDKR	82
Mus musculus Fgf5b	51	------RHTR--S-------YDYMEGGDIRVR----------R-LFCR-T-QW-----YLRIDKR	82
Rattus norvegicus Fgf5b	51	------RHTR--S-------YDYMEGGDIRVR----------R-LFCR-T-QW-----YLRIDKR	82
Cavia porcellus FGF5B	51	------RHTR--S-------YDYMEGGDIRVR----------R-LFCR-T-QW-----YLRIDKR	82
Oryctolagus cuniculus FGF5B	51	------RHTR--S-------YDYMEGGDIRVR----------R-LFCR-T-QW-----YLRIDKR	82
Bos taurus FGF5B	51	------RHTR--S-------YDYMEGGDIRVR----------R-LFCR-T-QW-----YLRIDKR	82
Vicugna pacos FGF5B	51	------RHTR--S-------YDYMEGGDIRVR----------R-LFCR-T-QW-----YLRIDKR	82

```
Equus caballus FGF5B              51 ------RHTR--S-------YDYMEGGDIRVR----------R-LFCR-T-QW-----YLRIDKR  82
Canis lupus familiaris FGF5B      51 ------RHTR--S-------YDYMEGGDIRVR----------R-LFCR-T-QW-----YLRIDKR  82
Myotis lucifugus FGF5B            51 ------RHTR--S-------YDYMEGGDIRVR----------R-LFCR-T-QW-----YLRIDKR  82
Sorex araneus FGF5B               51 ------RHTR--S-------YDYMEGGDIRVR----------R-LFCR-T-QW-----YLRIDKR  82
Dasypus novemcinctus FGF5B        51 ------RHTR--S-------YDYMEGGDIRVR----------R-LFCR-T-QW-----YLRIDKR  82
Loxodonta africana FGF5B          51 ------RHTR--S-------YDYMEGGDIRVR----------R-LFCR-T-QW-----YLRIDKR  82
Homo sapiens FGF5C                23 ----A-RLRR--DAGGRGGVYEHL-GGAPRRR----------K-LYCA-T-KY-----HLQLHPS  61
Pongo abelii FGF5C                23 ----A-RLRR--DAGGRGGVYEHL-GGAPRRR----------K-LYCA-T-KY-----HLQLHPS  61
Macaca mulatta FGF5C              23 ----A-RLRR--DAGGRGGVYEHL-GGAPRRR----------K-LYCA-T-KY-----HLQLHPS  61
Mus musculus Fgf5c                23 ----T-RLRR--DAGGRGGVYEHL-GGAPRRR----------K-LYCA-T-KY-----HLQLHPS  61
Rattus norvegicus Fgf5c           23 ----T-RLRR--DAGGRGGVYEHL-GGAPRRR----------K-LYCA-T-KY-----HLQLHPS  61
Cavia porcellus FGF5C             23 ----A-RLRR--DAGGRGGVYEHL-GGAPRRR----------K-LYCA-T-KY-----HLQLHPS  61
Bos taurus FGF5C                  23 ----A-RPRR--DAGGRGGVYEHL-GGAPRRR----------K-LYCA-T-KY-----HLQLHPS  61
Homo sapiens FGF5D                23 TP-SASRGPR--S-------YPHLEG-DVRWR----------R-LF-SST-HF-----FLRVDPG  58
Macaca mulatta FGF5D              23 TP-SAPQRTR--S-------YPHLEG-DVRWR----------R-LF-SST-HF-----FLRVDPG  58
Papio hamadryas FGF5D             23 TP-SAPQRTR--S-------YPHLEG-DVRWR----------R-LF-SST-HF-----FLRVDPG  58
Otolemur garnettii FGF5D          21 -P-TALRRVR--N-------YPHLEG-DVRWR----------R-LF-SST-HF-----FLRVDPT  55
Mus musculus Fgf5d                21 -P-GG---------------YPHLEG-DVRWR----------R-LF-SST-HF-----FLRVDLG  49
Rattus norvegicus Fgf5d           21 -P-GG---------------YPHLEG-DVRWR----------R-LF-SST-HF-----FLRVDPG  49
Bos taurus FGF5D                  87 TL-NTPRRPR--S-------YPHLEG-DVRWR----------R-LF-SST-HF-----FLLVDPS 122
Myotis lucifugus FGF5D            23 TP-GGPRRPR--S-------YPHLEG-DVRWR----------R-LF-SST-HF-----FLRVDPS  58
Pteropus vampyrus FGF5D           23 SP-SSPRRPR--S-------YPHLEG-DVRWR----------R-LF-SST-HF-----FLRVDPS  58
Homo sapiens FGF6A                39 ---NQT---R--------A-RDDVSRKQLRLY----------Q-LY-SRTSGK-----HIQVL-G  70
Mus musculus Fgf6a                39 ---NQT---R--------A-RDDVSRKQLRLY----------Q-LY-SRTSGK-----HIQVL-G  70
Cavia porcellus FGF6A             39 ---NQT---R--------A-RDDVSRKQLRLY----------Q-LY-SRTSGK-----HIQVL-G  70
Bos taurus FGF6A                  39 ---NQT---R--------A-RDDVSRKQLRLY----------Q-LY-SRTSGK-----HIQVL-G  70
Canis lupus familiaris FGF6A      39 ---NQT---R--------A-RDDVSRKQLRLY----------Q-LY-SRTSGK-----HIQVL-G  70
Homo sapiens FGF6B                53 SP-NFNQYVR--D---QGAMTDQLSRRQIREY----------Q-LY-SRTSGK-----HVQV-TG  93
Pan troglodytes FGF6B             53 SP-NFNQYVR--D---QGAMTDQLSRRQIREY----------Q-LY-SRTSGK-----HVQV-TG  93
Nomascus leucogenys FGF6B         53 SP-NFNQYVR--D---QGAMTDQLSRRQIREY----------Q-LY-SRTSGK-----HVQV-TG  93
Macaca mulatta FGF6B             147 SP-NFNQYVR--D---QGAMTDQLSRRQIREY----------Q-LY-SRTSGK-----HVQV-TG 187
Mus musculus Fgf6b                90 SP-NFNQYVR--D---QGAMTDQLSRRQIREY----------Q-LY-SRTSGK-----HVQV-TG 130
Rattus norvegicus Fgf6b           89 SP-NFNQYVR--D---QGAMTDQLSRRQIREY----------Q-LY-SRTSGK-----HVQV-TG 129
Cavia porcellus FGF6B             92 SP-NFNQYVR--D---QGAMTDQLSRRQIREY----------Q-LY-SRTSGK-----HVQV-TG 132
Canis lupus familiaris FGF6B      57 SP-NFNQYVR--D---QGAMTDQLSRRQIREY----------Q-LY-SRTSGK-----HVQV-TG  97
Felis catus FGF6B                 99 SP-NFNQYVR--D---QGAMTDQLSRRQIREY----------Q-LY-SRTSGK-----HVQV-TG 139
Myotis lucifugus FGF6B            27 SP-NFNQYVR--D---QGAMTDQLSRRQIREY----------Q-LY-SRTSGK-----HVQV-TG  67
Dasypus novemcinctus FGF6B         1 -------------------MTDQLSRRQVREY----------Q-LY-SRTSGR-----HVQV-AG  28
Loxodonta africana FGF6B          46 SP-NFNQYVR--D---QGAMTDQLSRRQIREY----------Q-LY-SRTSGK-----HVQV-TG  86
Homo sapiens FGF6C               146 SP-NFTQHVR--E---QSLVTDQLSRRLIRTY----------Q-LY-SRTSGK-----HVQVLAN 187
Nomascus leucogenys FGF6C         17 SP-NFTQHVR--E---QSLVTDQLSRRLIRTY----------Q-LY-SRTSGK-----HVQVLAN  58
Macaca mulatta FGF6C              17 SP-NFTQHVR--E---QSLVTDQLSRRLIRTY----------Q-LY-SRTSGK-----HVQVLAN  58
Papio hamadryas FGF6C             17 SP-NFTQHVR--E---QSLVTDQLSRRLIRTY----------Q-LY-SRTSGK-----HVQVLAN  58
Mus musculus Fgf6c                17 SP-NFTQHVR--E---QSLVTDQLSRRLIRTY----------Q-LY-SRTSGK-----HVQVLAN  58
Rattus norvegicus Fgf6c           17 SP-NFTQHVR--E---QSLVTDQLSRRLIRTY----------Q-LY-SRTSGK-----HVQVLAN  58
Bos taurus FGF6C                  50 SP-NFTQHVR--E---QSLVTDQLSRRLIRTY----------Q-LY-SRTSGK-----HVQVLAN  91
Homo sapiens FGF7A                59 -PVAAQP-KEA-A-V-QSGAGDYL-LGIKRLR----------R-LYCNVGIGF-----HLQALPD 101
Macaca mulatta FGF7A              59 -PVAAQP-KEA-A-V-QSGAGDYL-LGIKRLR----------R-LYCNVGIGF-----HLQALPD 101
Callithrix jacchus FGF7A          59 -PVAAQP-KEA-A-V-QSGAGDYL-LGIKRLR----------R-LYCNVGIGF-----HLQVLPD 101
Mus musculus Fgf7a                55 -PVAAQP-PQA-A-V-RSGAGDYL-LGLKRLR----------R-LYCNVGIGF-----HLQVLPD  97
Rattus norvegicus Fgf7a           55 -PVAAQP-PQA-A-V-RSGAGDYL-LGLKRLR----------R-LYCNVGIGF-----HLQVLPD  97
Cavia porcellus FGF7A             59 -PVAAQP-QEA-V-V-QSGAGDYL-LGIKRLR----------R-LYCNVGIGF-----HLQVLPD 101
Canis lupus familiaris FGF7A      59 -PVAAQP-KEA-A-V-QSGAGDYL-LGIKRLR----------R-LYCNVGIGF-----HLQVLPD 101
Procavia capensis FGF7A           50 -PAAARP-KEA-A-V-QSGAGDYL-LGIKRLR----------R-LYCNVGIGF-----HLQVLPD  92
Homo sapiens FGF7B                66 ---AG----EI-AGV-NWESG-YL-VGIKRQR----------R-LYCNVGIGF-----HLQVLPD 103
Pan troglodytes FGF7B             66 ---AG----EI-AGV-NWESG-YL-VGIKRQR----------R-LYCNVGIGF-----HLQVLPD 103
Pongo abelii FGF7B                66 ---AG----EI-AGV-NWESG-YL-VGIKRQR----------R-LYCNVGIGF-----HLQVLPD 103
Nomascus leucogenys FGF7B         66 ---AG----EI-AGV-NWESG-YL-VGIKRQR----------R-LYCNVGIGF-----HLQVLPD 103
Macaca mulatta FGF7B              66 ---AG----EI-AGV-NWESG-YL-VGIKRQR----------R-LYCNVGIGF-----HLQVPPD 103
Papio hamadryas FGF7B             66 ---AG----EI-AGV-NWESG-YL-VGIKRQR----------R-LYCNVGIGF-----HLQVPPD 103
Callithrix jacchus FGF7B          66 ---AG----EI-TRV-NWESG-YL-VGIKRQR----------R-LYCNVGIGF-----HLQVPPD 103
Mus musculus Fgf7b                66 ---AG----EI-SGV-NWESG-YL-VGIKRQR----------R-LYCNVGIGF-----HLQVPPD 103
Rattus norvegicus Fgf7b           66 ---AG----EI-SGV-NWESG-YL-VGIKRQR----------R-LYCNVGIGF-----HLQVPPD 103
Cavia porcellus FGF7B             66 ---AG----EI-AGV-NWENG-YL-VGIKRQR----------R-LYCNVGIGF-----HLQVPLN 103
Oryctolagus cuniculus FGF7B       66 ---AG----EI-AGV-NWESG-YL-VGIKRQR----------R-LYCNVGIGF-----HLQVPPD 103
Tursiops truncatus FGF7B          66 ---AG----EV-AGV-NWESG-YL-VGIKRQR----------R-LYCNVGIGF-----HLQVPPD 103
Bos taurus FGF7B                  66 ---AG----EI-AGV-NWESG-YL-VGIKRQR----------R-LYCNVGIGF-----HLQVPPD 103
Equus caballus FGF7B              66 ---AG----EI-AGV-NWESG-YL-VGIKRQR----------R-LYCNVGIGF-----HLQVPPD 103
Canis lupus familiaris FGF7B      66 ---AG----EI-AGV-NWESG-YL-VGIKRQR----------R-LYCNVGIGF-----HLQVPPD 103
Myotis lucifugus FGF7B            66 ---AG----EI-AGV-NWESG-YL-VGIKRQR----------R-LYCNVGIGF-----HLQVPPD 103
Loxodonta africana FGF7B          66 ---AG----DI-AGV-NWESG-YL-VGIKRQR----------R-LYCNVGIGF-----HLQVPPD 103
Homo sapiens FGF8A                27 -------FSDA-GPH---VHYGWGD-PI-RLR----------H-LY-TSG-PHGLSSCFLRIRAD  65
Pongo abelii FGF8A                27 -------FSDS-GPH---VHYGWGD-PI-RLR----------H-LY-TSG-PHGLSSCFLRIRAD  65
Macaca mulatta FGF8A              27 -------FSDA-GPH---VHYGWGD-PI-RLR----------H-LY-TSG-PHGLSSCFLRIRTD  65
Microcebus murinus FGF8A         108 -------FSDA-GPH---VHYGWGE-PI-RLR----------H-LY-TAG-PHGLSSCFLRIRAD 146
Otolemur garnettii FGF8A          30 -------FSDA-GPH---VHYGWGE-PI-RLR----------H-LY-TAG-PHGLSSCFLRVRTD  68
Mus musculus Fgf8a                30 -QQSQ-SVSDE-DPL---FLYGWGK-IT-RLQ----------Y-LY-SAG-PY-VSNCFLRIRSD  72
Rattus norvegicus Fgf8a           30 -QQSQ-SVSDE-GPL---FLYGWGK-IT-RLQ----------Y-LY-SAG-PY-VSNCFLRIRSD  72
Cavia porcellus FGF8A             30 -RRSL-ALSDQ-GPH---LYYGWDQ-PI-RLR----------H-LY-AAG-PYGRSRCFLRIHTD  73
```

```
Bos taurus FGF8A                 30 -------FSDA-GPH---VHYGWGE-SV-RLR----------H-LY-TAG-PQGLYSCFLRIHSD  68
Myotis lucifugus FGF8A           30 -------LSDA-GPH---VHYGWGE-SI-RLR----------H-LY-TSG-PHGPSSCFLRIRAD  68
Pteropus vampyrus FGF8A          27 -------LSDA-GPH---VHYGWGE-AI-RLR----------H-LY-TAG-PHGPSSCFLRIRAD  65
Dasypus novemcinctus FGF8A       30 --------SDA-GPH---MHYGWGE-PV-RLR----------H-LY-TAG-PYGLFSCFLHIRAD  67
Homo sapiens FGF8B              151 -R-AY---PNA-SPL---LGSSWGGL-I--------------H-LY-TAT-ARN-SY-HLQIHKN 186
Pan troglodytes FGF8B           151 -R-AY---PNA-SPL---LGSSWGGL-I--------------H-LY-TAT-ARN-SY-HLQIHKN 186
Nomascus leucogenys FGF8B       151 -R-AY---PNA-SPL---LGSSWGGL-I--------------H-LY-TAT-ARN-SY-HLQIHKN 186
Otolemur garnettii FGF8B         23 -R-AY---PNA-SPL---LSSSWGGL-T--------------H-LY-TAS-ARN-SY-HLQIHKD  58
Mus musculus Fgf8b               23 -R-AY---PDT-SPL---LGSNWGSL-T--------------H-LY-TAT-ART-SY-HLQIHRD  58
Rattus norvegicus Fgf8b          96 -R-AY---SDT-SPL---LGSNWGSL-T--------------H-LY-TAT-ARN-SY-HLQIHRD 131
Cavia porcellus FGF8B            18 ---AY---PDA-SPL---LTSSWGGL-I--------------H-LY-TAT-ARN-SY-HLQIHKD  52
Oryctolagus cuniculus FGF8B      84 -R-AY---PDT-SPL---LSSSWAGL-T--------------H-LY-TAT-ARN-SY-HLQIHKD 119
Equus caballus FGF8B             18 -K-AY---PNA-SPL---LDSSWGSL-T--------------H-LY-TAT-ARN-SY-HLQIHKD  53
Canis lupus familiaris FGF8B     85 -R-AY---SNT-SPL---LGSSWGSL-T--------------H-LY-TAT-ARN-SY-HLQIHKD 120
Dasypus novemcinctus FGF8B       20 -R-AH---PDA-SPL---LGSGWGGL-T--------------H-LY-TAT-ARN-SY-HLQIHKD  55
Loxodonta africana FGF8B         82 -R-AY---PNA-SPL---LHSSWGGL-T--------------H-LY-TAT-ARN-SY-HLQIHKD 117
Homo sapiens FGF8C               27 -Q-AH-PIPDS-SPL---LQFG-GQ--V-RQR----------Y-LY-TDD-AQQTEA-HLEIRED  66
Pan troglodytes FGF8C            27 -Q-AH-PIPDS-SPL---LQFG-GQ--V-RQR----------Y-LY-TDD-AQQTEA-HLEIRED  66
Pongo abelii FGF8C               27 -Q-AH-PIPDS-SPL---LQFG-GQ--V-RQR----------Y-LY-TDD-AQQTEA-HLEIRED  66
Nomascus leucogenys FGF8C        27 -Q-AH-PIPDS-SPL---LQFG-GQ--V-RQR----------Y-LY-TDD-AQQTEA-HLEIRED  66
Otolemur garnettii FGF8C         29 -Q-AH-PIPDS-SPL---LQFG-GQ--V-RQR----------Y-LY-TDD-AQETEA-HLEIRED  68
Mus musculus Fgf8c               28 -Q-AY-PIPDS-SPL---LQFG-GQ--V-RQR----------Y-LY-TDD-DQDTEA-HLEIRED  67
Rattus norvegicus Fgf8c          28 -E-AY-PISDS-SPL---LQFG-GQ--V-RQR----------Y-LY-TDD-DQDTEA-HLEIRED  67
Cavia porcellus FGF8C            33 -Q-AH-PIPDS-SPL---LQFG-GQ--V-RQR----------Y-LY-TDD-AQDTEV-HLEIRAD  72
Oryctolagus cuniculus FGF8C      27 -Q-AH-PIPDS-SPL---LQFG-DQ--V-RQQ----------H-LY-TDD-AQETEA-HLEIRAD  66
Ochotona princeps FGF8C          23 -Q-AR-PIPDS-SPL---LQFG-GQ--V-RQR----------H-LY-TDD-AQESEV-HLEIRAD  62
Bos taurus FGF8C                27 -R-AH-PIPDS-SPL---LQFG-GQ--V-RQR----------Y-LY-TDD-AQETEA-HLEIRAD  66
Equus caballus FGF8C             27 -Q-SH-PIPDS-SPL---LQFG-GQ--V-RQR----------H-LY-TDD-AQETEA-HLEIRAD  66
Canis lupus familiaris FGF8C     27 -R-AH-PIPDS-SPL---LQFG-GQ--V-RQR----------Y-LY-TDD-AQETEA-HLEIRAD  66
Felis catus FGF8C                28 -Q-AH-PIPDS-SPL---LQFG-GQ--V-RQR----------F-LY-TDD-AQETEV-HLEIKAD  67
Sorex araneus FGF8C              27 -Q-AH-PLPDS-SPL---LQFG-GQ--V-RLR----------F-LY-TDD-AQRTGA-HLEIRAD  66
Procavia capensis FGF8C          27 -Q-GY-PIPDS-SPL---LQFG-GQ--V-RQR----------Y-LY-TDD-AQETEA-HLEIRAD  66
```

Homo sapiens FGF1A	91	**GTID-GTKDE-NS-DYT---L-FNLIPVGLRVVAIQGV-KASLYV-AMNGEGYLYSSDV--FTPE**	144
		330 340 350 360 370 380 390	
Homo sapiens FGF1A	91	GTID-GTKDE-NS-DYT---L-FNLIPVGLRVVAIQGV-KASLYV-AMNGEGYLYSSDV--FTPE	144
Pan troglodytes FGF1A	91	GTID-GTKDE-NS-DYT---L-FNLIPVGLRVVAIQGV-KASLYV-AMNGEGYLYSSDV--FTPE	144
Nomascus leucogenys FGF1A	91	GTID-GTKDE-NS-DYT---L-FNLIPVGLRVVAIQGV-KASLYV-AMNGEGYLYSSDV--FTPE	144
Callithrix jacchus FGF1A	91	GTID-GTKDE-NS-DYT---L-FNLIPVGLRVVAIQGV-KASLYV-AMNGEGYLYSSDV--FTPE	144
Mus musculus Fgf1a	91	GTID-GTKDE-NS-DYT---L-FNLIPVGLRVVAIQGV-KASLYV-AMNGEGYLYSSDV--FTPE	144
Rattus norvegicus Fgf1a	91	GTID-GTKDE-NS-DYT---L-FNLIPVGLRVVAIQGV-KASLYV-AMNGEGYLYSSDV--FTPE	144
Cavia porcellus FGF1A	91	GTID-GTKDE-NS-DYT---L-FNLIPVGLRVVAIQGV-KASLYV-AMNGEGYLYSSDV--FTPE	144
Oryctolagus cuniculus FGF1A	91	GTID-GTKDE-NS-DYT---L-FNLIPVGLRVVAIQGV-KASLYV-AMNGEGYLYSSDV--FTPE	144
Equus caballus FGF1A	91	GTID-GTKDE-NS-DYT---L-FNLIPVGLRVVAIQGV-KASLYV-AMNGEGYLYSSDV--FTPE	144
Canis lupus familiaris FGF1A	91	GTID-GTKDE-NS-DYT---L-FNLIPVGLRVVAIQGV-KASLYV-AMNGEGYLYSSDV--FTPE	144
Dasypus novemcinctus FGF1A	91	GTID-GTKDE-NS-DYT---L-FNLIPVGLRVVAIQGV-KAGLYV-AMNGEGYLYSSDV--FTPE	144
Homo sapiens FGF1B	89	GALD-GTKDD-ST-NST---L-FNLIPVGLRVVAIQGV-KTGLYI-AMNGEGYLYPSEL--FTPE	142
Pan troglodytes FGF1B	89	GALD-GTKDD-ST-NST---L-FNLIPVGLRVVAIQGV-KTGLYI-AMNGEGYLYPSEL--FTPE	142
Pongo abelii FGF1B	89	GALD-GTKDD-ST-NST---L-FNLIPVGLRVVAIQGV-KTGLYI-AMNGEGYLYPSEL--FTPE	142
Macaca mulatta FGF1B	89	GALD-GTKDD-ST-NST---L-FNLIPVGLRVVAIQGV-KTGLYI-AMNGEGYLYPSEL--FTPE	142
Callithrix jacchus FGF1B	89	GALD-GTKDD-ST-NST---L-FNLIPVGLRVVAIQGV-KTGLYI-AMNGEGYLYPSEL--FTPE	142
Mus musculus Fgf1b	89	GALD-GTKDD-ST-NST---L-FNLIPVGLRVVAIQGV-KTGLYI-AMNGEGYLYPSEL--FTPE	142
Oryctolagus cuniculus FGF1B	89	GALD-GTKDD-ST-NST---L-FNLIPVGLRVVAIQGV-KTGLYI-AMNGEGYLYPSEL--FTPE	142
Equus caballus FGF1B	89	GALD-GTKDD-ST-NST---L-FNLIPVGLRVVAIQGV-KTGLYI-AMNGEGYLYPSEL--FTPE	142
Dasypus novemcinctus FGF1B	89	GALD-GTKDD-ST-NST---L-FNLIPVGLRVVAIQGV-KTGLYL-AMNGEGYLYPSEL--FTPE	142
Homo sapiens FGF1C	87	GTID-GTKDE-DS-TYT---L-FNLIPVGLRVVAIQGV-QTKLYL-AMNSEGYLYTSEL--FTPE	140
Pan troglodytes FGF1C	87	GTID-GTKDE-DS-TYT---L-FNLIPVGLRVVAIQGV-QTKLYL-AMNSEGYLYTSEL--FTPE	140
Pongo abelii FGF1C	87	GTID-GTKDE-DS-TYT---L-FNLIPVGLRVVAIQGV-QTKLYL-AMNSEGYLYTSEL--FTPE	140
Macaca mulatta FGF1C	87	GTID-GTKDE-DS-TYT---L-FNLIPVGLRVVAIQGV-QTKLYL-AMNSEGYLYTSEL--FTPE	140
Mus musculus Fgf1c	87	GTID-GTKDE-DS-TYT---L-FNLIPVGLRVVAIQGV-QTKLYL-AMNSEGYLYTSEH--FTPE	140
Rattus norvegicus Fgf1c	87	GTID-GTKDE-DS-TYT---L-FNLIPVGLRVVAIQGV-QTKLYL-AMNSEGYLYTSEH--FTPE	140
Cavia porcellus FGF1C	87	GTID-GTKDE-DS-TYT---L-FNLIPVGLRVVAIQGV-QTKLYL-AMNSEGYLYTSEH--FTPE	140
Bos taurus FGF1C	87	GTID-GTKDE-DS-TYT---L-FNLIPVGLRVVAIQGV-QTKLYL-AMNSEGYLYTSEH--FTPE	140
Myotis lucifugus FGF1C	87	GTID-GTKDE-DS-TYT---L-FNLIPVGLRVVAIQGV-QTKLYL-AMNSEGYLYTSEH--FTPE	140
Dasypus novemcinctus FGF1C	87	GTID-GTKDE-DS-TYT---L-FNLIPVGLRVVAIQGV-QTKLYL-AMNSEGYLYTSEH--FTPE	140
Loxodonta africana FGF1C	87	GTID-GTKDE-DS-TFT---L-FNLIPVGLRVVAIQGV-QTKLYL-AMNSEGYLYTSEH--FTPE	140
Homo sapiens FGF1D	89	GSIQ-GTPED-TS-SFT---H-FNLIPVGLRVVTIQSA-KLGHYM-AMNAEGLLYSSPH--FTAE	142
Pan troglodytes FGF1D	89	GSIQ-GTPED-TS-SFT---H-FNLIPVGLRVVTIQSA-KLGHYM-AMNAEGLLYSSPH--FTAE	142
Nomascus leucogenys FGF1D	89	GSIQ-GTPED-TS-SFT---H-FNLIPVGLRVVTIQSA-KLGHYM-AMNAEGLLYSSPH--FTAE	142
Macaca mulatta FGF1D	89	GSIQ-GTPED-TS-SFT---H-FNLIPVGLRVVTIQSA-KLGHYM-AMNAEGLLYSSPH--FTAE	142
Otolemur garnettii FGF1D	89	GSIQ-GTPED-TS-SFT---H-FNLIPVGLRVVTIQSA-KLGHYM-AMNAEGLLYSSPH--FTAE	142
Mus musculus Fgf1d	89	GSIQ-GTPED-TS-SFT---H-FNLIPVGLRVVTIQSA-KLGHYM-AMNAEGLLYSSPH--FTAE	142
Rattus norvegicus Fgf1d	89	GSIQ-GTPED-TS-SFT---H-FNLIPVGLRVVTIQSA-KLGHYM-AMNAEGLLYSSPH--FTAE	142
Cavia porcellus FGF1D	89	GSIQ-GTPED-TS-SFT---H-FNLIPVGLRVVTIQSA-KLGHYM-AMNAEGLLYCSPH--FTAE	142
Oryctolagus cuniculus FGF1D	89	GSIQ-GAPED-TS-SFT---H-FNLIPVGLRVVTIQSA-KLGHYM-AMNAEGLLYSSPH--FTAE	142
Bos taurus FGF1D	89	GSIQ-GTPED-TS-SFT---H-FNLIPVGLRVVTIQSA-KLGHYM-AMNAEGLLYSSPH--FTAE	142
Equus caballus FGF1D	89	GSIQ-GTPED-TS-SFT---H-FNLIPVGLRVVTIQSA-KLGHYM-AMNAEGLLYSSPH--FTAE	142
Canis lupus familiaris FGF1D	89	GSIQ-GTPED-TS-SFT---H-FNLIPVGLRVVTIQSA-KLGHYM-AMNAEGLLYSSPH--FTAE	142
Pteropus vampyrus FGF1D	89	GSIQ-GTPED-TS-SFT---H-FNLIPVGLRVVTIQSA-KLGHYM-AMNADGLLYSSPH--FTAE	142
Dasypus novemcinctus FGF1D	89	GSIQ-GTPED-TS-AFT---H-FNLIPVGLRVVTIQSA-KLGHYM-AMNAEGLLYSSAH--FTAE	142
Loxodonta africana FGF1D	89	GSIQ-GTPED-TS-SFT---H-FNLIPVGLRVVTIQST-KLGHYM-AMNAEGLLYSSPH--FTAE	142
Homo sapiens FGF2A	47	GRVD-GVREK-SD-PHI--KLQLQAEERG--VVSIKGVC-ANRYL-AMKEDGRLLASK-CV-TDE	100
Pongo abelii FGF2A	47	GRVD-GVREK-SD-PHI--KLQLQAEERG--VVSIKGVC-ANRYL-AMKEDGRLLASK-CV-TDE	100
Nomascus leucogenys FGF2A	47	GRVD-GVREK-SD-PHI--KLQLQAEERG--VVSIKGVC-ANRYL-AMKEDGRLLASK-CV-TDE	100
Macaca mulatta FGF2A	47	GRVD-GVREK-SD-PHI--KLQLQAEERG--VVSIKGVC-ANRYL-AMKEDGRLLASK-CV-TDE	100
Mus musculus Fgf2a	46	GRVD-GVREK-SD-PHV--KLQLQAEERG--VVSIKGVC-ANRYL-AMKEDGRLLASK-CV-TEE	99
Rattus norvegicus Fgf2a	46	GRVD-GVREK-SD-PHV--KLQLQAEERG--VVSIKGVC-ANRYL-AMKEDGRLLASK-CV-TEE	99
Oryctolagus cuniculus FGF2A	47	GRVD-GVREK-SD-PHI--KLQLQAEERG--VVSIKGVC-ANRYL-AMKEDGRLLASK-CV-TDE	100
Dasypus novemcinctus FGF2A	47	GRVD-GVREK-SD-PNI--KLQLQAEERG--VVSIKGVC-ANRYL-AMREDGRLQASK-CV-TDE	100
Homo sapiens FGF2B	44	GTVD-GTRDR-SD-QHI--QLQLSAESVG--EVYIKST-ETGQYL-AMDTDGLLYGSQ--TPNEE	97
Pan troglodytes FGF2B	44	GTVD-GTRDR-SD-QHI--QLQLSAESVG--EVYIKST-ETGQYL-AMDTDGLLYGSQ--TPNEE	97
Pongo abelii FGF2B	44	GTVD-GTRDR-SD-QHI--QLQLSAESVG--EVYIKST-ETGQYL-AMDTDGLLYGSQ--TPNEE	97
Nomascus leucogenys FGF2B	44	GTVD-GTRDR-SD-QHI--QLQLSAESVG--EVYIKST-ETGQYL-AMDTDGLLYGSQ--TPNEE	97
Callithrix jacchus FGF2B	44	GTVD-GTRDR-SD-QHI--QLQLSAESVG--EVYIKST-ETGQYL-AMDTDGLLYGSQ--TPNEE	97
Tarsius syrichta FGF2B	44	GTVD-GTRDR-SD-QHI--QLQLSAESVG--EVYIKST-ETGQYL-AMDTDGLLYGSQ--TPNEE	97
Otolemur garnettii FGF2B	44	GTVD-GTQDR-SD-QHI--QLQLSAESVG--EVYIKST-QTGQYL-AMDSDGLLYGSQ--TPNEE	97
Mus musculus Fgf2b	44	GTVD-GTRDR-SD-QHI--QLQLSAESAG--EVYIKGT-ETGQYL-AMDTEGLLYGSQ--TPNEE	97
Rattus norvegicus Fgf2b	44	GTVD-GTRDR-SD-QHI--QLQLSAESAG--EVYIKGT-ETGQYL-AMDTEGLLYGSQ--TPNEE	97
Cavia porcellus FGF2B	44	GTVD-GTRDR-SD-QHI--QLQLSAEGVG--EVYIQST-ETGQYL-AMDTDGLLYGSQ--TPSEE	97
Oryctolagus cuniculus FGF2B	44	GTVD-GTRDR-SD-QHI--QLQLSAESVG--EVYIKST-ETGQYL-AMDTDGLLYGSQ--TPSEE	97
Tursiops truncatus FGF2B	44	GTVD-GTRDR-SD-QHI--QLQLSAESVG--EVYIKST-ETGQYL-AMDTDGLLYGSQ--TPNEE	97
Bos taurus FGF2B	44	GTVD-GTKDR-SD-QHI--QLQLCAESIG--EVYIKST-ETGQFL-AMDTDGLLYGSQ--TPNEE	97
Equus caballus FGF2B	44	GTVD-GTRDR-SD-QHI--QLQLSAESVG--EVYIKST-ETGQYL-AMDTDGLLYGSQ--TPNEE	97
Canis lupus familiaris FGF2B	44	GTVD-GTRDR-SD-QHI--QLQLSAESVG--EVYIKST-ETGQYL-AMDTDGLLYGSQ--TPNEE	97
Felis catus FGF2B	44	GTVD-GTRDR-SD-QHI--QLQLSAESVG--EVYIKST-ETGQYL-AMDTDGLLYGSQ--TPNEE	97
Myotis lucifugus FGF2B	44	GTVD-GTRDR-SD-QHI--QLQLSAESVG--EVYIKST-ESGQYL-AMDSDGLLYGSQ--TPNEE	97
Pteropus vampyrus FGF2B	44	GTVD-GTRDK-SD-QHI--QLQLSAESVG--EVYIKST-ESGQYL-AMDSDGLLYGSQ--TPDED	97
Dasypus novemcinctus FGF2B	44	GTVD-GTRDR-SD-QHI--QLQLSAESVG--EVYIKSA-ETGQYL-AMDTDGLLYGSE--TPSEE	97
Loxodonta africana FGF2B	44	GTVD-GTRDR-SD-QHI--QLQLSAESVG--EVYIKGT-ETGQYL-AMDTDGLLYGSQ--TPNEE	97
Homo sapiens FGF3A	107	GKVN-GSHEA-NM--LS--VLEIFAVSQG--IVGIRGV-FSNKFL-AMSKKGKLHASA--KFTDD	159
Pan troglodytes FGF3A	107	GKVN-GSHEA-NM--LS--VLEIFAVSQG--IVGIRGV-FSNKFL-AMSKKGKLHASA--KFTDD	159
Nomascus leucogenys FGF3A	106	GKVN-GSHEA-NM--LS--ILEIFAVSQG--IVGIRGV-FSNKFL-AMSKKGKLHASA--KFTDD	158

```
Macaca mulatta FGF3A            107 GKVN-GSHEA-NM--LS--ILEIFAVSQG--IVGIRGV-FSNKFL-AMSKKGKLHASA--KFTDD 159
Papio hamadryas FGF3A           107 GKVN-GSHEA-NM--LS--ILEIFAVSQG--IVGIRGV-FSNKFL-AMSKKGKLHASA--KFTDD 159
Callithrix jacchus FGF3A        107 GKVN-GSHEA-NM--LS--ILEIFAVSQG--IVGIRGV-FSNKFL-AMSKKGKLHASA--KFTDD 159
Otolemur garnettii FGF3A        108 GKVN-GSHEA-NK--LS--ILEIFAVSQG--IVGIRGV-FSNKFL-AMSKKGKLHASA--KFTDD 160
Mus musculus Fgf3a             105 GKVN-GSHEA-SV--LS--ILEIFAVSQG--IVGIRGV-FSNKFL-AMSKKGKLHASA--KFTDD 157
Rattus norvegicus Fgf3a         105 GKVN-GSHEA-SV--LS--ILEIFAVSQG--IVGIRGV-FSNKFL-AMSKKGKLHASA--KFTDD 157
Dipodomys ordii FGF3A          111 GKVN-GSHEA-NM--LS--ILEIFAVSQG--IVGIRGV-FSNKFL-AMSKKGKLHASA--KFTDD 163
Cavia porcellus FGF3A          108 GKVN-GSHEA-NM--LS--ILEIFAVSQG--IVGIRGV-FSNKFL-AMSKKGKLHASA--KFTDD 160
Oryctolagus cuniculus FGF3A    106 GKVN-GSHEA-NM--LS--ILEIFAVSQG--IVGIRGV-FSNKFL-AMSKKGKLHASA--KFRDD 158
Bos taurus FGF3A               109 GKVN-GSHEA-NM--LS--ILEIFAVSQG--IVGIRGV-FSNKFL-AMSKKGKLHASA--KFTDD 161
Equus caballus FGF3A           108 GKVN-GSHEA-NM--LS--ILEIFAVSQG--IVGIRGV-FSNKFL-AMSKKGKLHASA--KFTDD 160
Canis lupus familiaris FGF3A   111 GKVN-GSHEA-NM--LS--ILEIFAVSQG--IVGIRGV-FSNKFL-AMSKKGKLHASA--KFTDD 163
Myotis lucifugus FGF3A         104 GKVN-GSHEA-NM--LS--ILEIFAVSQG--IVGIRGV-FSNKFL-AMSKKGKLHASA--KFTDD 156
Dasypus novemcinctus FGF3A     102 GKVN-GSHEA-NM--LS--ILEIFAVSQG--IVGIRGV-FSNKFL-AMSKKGKLHASA--KFTDD 154
Homo sapiens FGF4A              83 GSVQ-GTRQD-HS-LFG--ILEFISVAVGL--VSIRGV-DSGLYL-GMNDKGELYGSEK--LTSE 136
Pan troglodytes FGF4A           83 GSVQ-GTRQD-HS-LFG--ILEFISVAVGL--VSIRGV-DSGLYL-GMNDKGELYGSEK--LTSE 136
Nomascus leucogenys FGF4A       83 GSVQ-GTRQD-HS-LFG--ILEFISVAVGL--VSIRGV-DSGLYL-GMNDKGELYGSEK--LTSE 136
Macaca mulatta FGF4A            83 GSVQ-GTRQD-HS-LFG--ILEFISVAVGL--VSIRGV-DSGLYL-GMNDKGELYGSEK--LTSE 136
Papio hamadryas FGF4A           83 GSVQ-GTRQD-HS-LFG--ILEFISVAVGL--VSIRGV-DSGLYL-GMNDKGELYGSEK--LTSE 136
Callithrix jacchus FGF4A        83 GSVQ-GTRRD-HS-LFG--ILEFISVAVGL--VSIRGV-DSGLYL-GMNDKGELYGSEK--LTSE 136
Mus musculus Fgf4a              83 GTVQ-GTRQD-HS-LFG--ILEFISVAVGL--VSIRGV-DSGLYL-GMNDKGELYGSEK--LTSE 136
Rattus norvegicus Fgf4a         83 GSVQ-GTRQD-HS-LFG--ILEFISVAVGL--VSIRGV-DSGLYL-GMNGKGELYGSEK--LTSE 136
Bos taurus FGF4A                83 GSVQ-GTRQD-HS-LFG--ILEFISVAVGL--VSIRGV-DSGLYL-GMNDKGELYGSEK--LTSE 136
Vicugna pacos FGF4A             83 GSVQ-GTRQD-HS-LFG--ILEFISVAVGL--VSIRGV-DSGLYL-GMNDKGELYGSEK--LTSE 136
Pteropus vampyrus FGF4A         83 GSVQ-GTRQD-HS-LFG--ILEFISVAVGL--VSIRGV-DSGLYL-GMNDKGELYGSEK--LTSE 136
Homo sapiens FGF4B              80 GTIQ-GTRKD-HS-RFG--ILEFISIAVGL--VSIRGV-DSGLYL-GMNEKGELYGSEK--LTQE 133
Pan troglodytes FGF4B           80 GTIQ-GTRKD-HS-RFG--ILEFISIAVGL--VSIRGV-DSGLYL-GMNEKGELYGSEK--LTQE 133
Gorilla gorilla FGF4B           80 GTIQ-GTRKD-HS-RFG--ILEFISIAVGL--VSIRGV-DSGLYL-GMNEKGELYGSEK--LTQE 133
Nomascus leucogenys FGF4B       80 GTIQ-GTRKD-HS-RFG--ILEFISIAVGL--VSIRGV-DSGLYL-GMNEKGELYGSEK--LTQE 133
Callithrix jacchus FGF4B        80 GTIQ-GTRKD-HS-RFG--ILEFISIAVGL--VSIRGV-DSGLYL-GMNDKGELYGSEK--LTQE 133
Otolemur garnettii FGF4B        80 GTIQ-GTRKD-HS-RFG--ILEFISIAVGL--VSIRGV-DSGLYL-GMNEKGELYGSDK--LTQE 133
Mus musculus Fgf4b              80 GTIQ-GTRKD-HS-RFG--ILEFISIAVGL--VSIRGV-DSGLYL-GMNEKGELYGSEK--LTQE 133
Rattus norvegicus Fgf4b         80 GTIQ-GTRKD-HS-RFG--ILEFISIAVGL--VSIRGV-DSGLYL-GMNEKGELYGSEK--LTQE 133
Oryctolagus cuniculus FGF4B     80 GTIQ-GTRKD-HS-RFG--ILEFISIAVGL--VSIRGV-DSGLYL-GMNEKGELYGSEK--LTQE 133
Tursiops truncatus FGF4B        80 GTIQ-GTRKD-HS-RFG--ILEFISIAVGL--VSIRGV-DSGLYL-GMNEKGELYGSEK--LTQE 133
Equus caballus FGF4B            80 GTIQ-GTRKD-HS-RFG--ILEFISIAVGL--VSIRGV-DSGLYL-GMNEKGELYGSEK--LTQE 133
Canis lupus familiaris FGF4B    80 GTIQ-GTRKD-HS-RFG--ILEFISIAVGL--VSIRGV-DSGLYL-GMNEKGELYGSEK--LTQE 133
Myotis lucifugus FGF4B          80 GTIQ-GTRKD-HS-RFG--ILEFISIAVGL--VSIRGV-DSGLYL-GMNEKGELYGSEK--LTQE 133
Pteropus vampyrus FGF4B         80 GTIQ-GTRKD-HS-RFG--ILEFISIAVGL--VSIRGV-DSGLYL-GMNEKGELFGSEK--LTQE 133
Dasypus novemcinctus FGF4B      80 GTIQ-GTRKD-HS-RFG--ILEFISIAVGL--VSIRGV-DSGLYL-GMNEKGELYGSEK--LTQE 133
Loxodonta africana FGF4B        80 GTIQ-GTRKD-HS-RFG--ILEFISIAVGL--VSIRGV-DSGLYL-GMNEKGELYGSEK--LTQE 133
Homo sapiens FGF4C              79 GTVH-GTRHD-HS-RFG--ILEFISLAVGL--ISIRGV-DSGLYL-GMNERGELYGSKK--LTRE 132
Pan troglodytes FGF4C           79 GTVH-GTRHD-HS-RFG--ILEFISLAVGL--ISIRGV-DSGLYL-GMNERGELYGSKK--LTRE 132
Gorilla gorilla FGF4C           79 GTVH-GTRHD-HS-RFG--ILEFISLAVGL--ISIRGV-DSGLYL-GMNERGELYGSKK--LTRE 132
Pongo abelii FGF4C              79 GTVH-GTRHD-HS-RFG--ILEFISLAVGL--ISIRGV-DSGLYL-GMNERGELYGSKK--LTRE 132
Nomascus leucogenys FGF4C       79 GTVH-GTRHD-HS-RFG--ILEFISLAVGL--ISIRGV-DSGLYL-GMNERGELYGSKK--LTRE 132
Otolemur garnettii FGF4C        79 GTVH-GTRQD-HS-RFG--ILEFISLAVGL--ISIQGV-DSGLYL-GMNERGELYGSKK--LTRE 132
Tupaia belangeri FGF4C          79 GTVH-GTRHD-HS-RFG--ILEFISLAVGL--ISIRGV-DSGLYL-GMNERGELYGSKK--LTHE 132
Mus musculus Fgf4c              79 GTVH-GTRHD-HS-RFG--ILEFISLAVGL--ISIRGV-DSGLYL-GMNERGELYGSKK--LTRE 132
Cavia porcellus FGF4C           79 GTVH-GTRHD-HS-RFG--ILEFISLAVGL--ISIRGV-DSGLYL-GMNERGELYGSKK--LTRE 132
Bos taurus FGF4C                79 GTVH-GTRHD-HS-RFG--ILEFISLAVGL--ISIRGV-DSGLYL-GMNERGELYGSKK--LTRE 132
Canis lupus familiaris FGF4C    79 GTVH-GTRHD-HS-RFG--ILEFISLAVGL--ISIRGV-DSGLYL-GMNERGELYGSKK--LTRE 132
Myotis lucifugus FGF4C          79 GTVH-GTRHD-HS-RFG--ILEFISLAVGL--ISIRGV-DSGLYL-GMNERGELYGSKK--LTHE 132
Pteropus vampyrus FGF4C         79 GTVH-GTRHD-HS-RFG--ILEFISLAVGL--ISIRGV-DSGLYL-GMNERGELYGSKK--LTRE 132
Dasypus novemcinctus FGF4C      79 GTVH-GTRHD-HS-RFG--ILEFISLAVGL--ISIRGV-DSGLYL-GMNERGELYGSKK--LTRE 132
Homo sapiens FGF5A              96 GKVS-GTKKE--NCPYS--ILEITSVEIG--VVAVKAI-NSNYYL-AMNKKGKLYGSKE--FNND 149
Pan troglodytes FGF5A           96 GKVS-GTKKE--NCPYS--ILEITSVEIG--VVAVKAI-NSNYYL-AMNKKGKLYGSKE--FNND 149
Nomascus leucogenys FGF5A       96 GKVS-GTKKE--NCPYS--ILEITSVEIG--VVAVKAI-NSNYYL-AMNKKGKLYGSKE--FNND 149
Macaca mulatta FGF5A            96 GKVS-GTKKE--NCPYS--ILEITSVEIG--VVAVKAI-NSNYYL-AMNKKGKLYGSKE--FNND 149
Callithrix jacchus FGF5A        97 GKVS-GTKKE--NCPYS--ILEITSVEIG--VVAVKAI-NSNYYL-AMNKKGKLYGSKE--FNND 150
Otolemur garnettii FGF5A        96 GKVG-GTKKE--NCPYS--ILEITSVEIG--VVAVKAI-NSNYYL-AMNKKGKLYGSKE--FNND 149
Tupaia belangeri FGF5A          95 GKVS-GTKKE--NCPYS--ILEITSVEIG--VVAVKAI-NSNYYL-AMNKKGKLYGSKE--FNTD 148
Mus musculus Fgf5a             148 GKVS-GTKNE--DCPYS--VLEITSVEIG--VVAVKAI-NSNYYL-AMNKKGKLYGSKE--FNND 201
Rattus norvegicus Fgf5a         103 GKVS-GTKKE--NCPYS--ILEITSVEIG--VVAVKAI-NSNYYL-AMNKKGKLYGSKE--FNND 156
Cavia porcellus FGF5A          104 GKVS-GTKKE--NCPYS--ILEITSVEIG--VVAVKAI-NSNYYL-AMNKKGKLYGSKE--FNND 157
Oryctolagus cuniculus FGF5A    108 GKVS-GTKKE--NCPYS--ILEITSVEIG--VVAVKAI-NSNYYL-AMNKKGKLYGSKE--FNND 161
Myotis lucifugus FGF5A         112 GKVS-GTKKE--NCPYS--IKITSVEIG--VVAVKAI-NSNYYL-AMNKRGKLYGSKE--FNND 165
Dasypus novemcinctus FGF5A     102 GKVS-GTKKE--NCPYS--ILEITSVEIG--VVAVKAI-NSNYYL-AMNKKGKLYGSKE--FNND 155
Loxodonta africana FGF5A       101 GKVS-GTKKE--NCPYS--ILEITSVEIG--VVAVKAI-NSNYYL-AMNKKGKLYGSKE--FNTD 154
Homo sapiens FGF5B              83 GKVK-GT-QEMKN-NYN--IMEIRTVAVG--IVAIKGV-ESEFYL-AMNKEGKLYAKKEC--NED 136
Nomascus leucogenys FGF5B       83 GKVK-GT-QEMKN-NYN--IMEIRTVAVG--IVAIKGV-ESEFYL-AMNKEGKLYAKKEC--NED 136
Macaca mulatta FGF5B            83 GKVK-GT-QEMKN-NYN--IMEIRTVAVG--IVAIKGV-ESEFYL-AMNKEGKLYAKKEC--NED 136
Callithrix jacchus FGF5B        83 GKVK-GT-QEMKN-NYN--IMEIRTVAVG--IVAIKGV-ESEFYL-AMNKEGKLYAKKDC--NED 136
Mus musculus Fgf5b              83 GKVK-GT-QEMKN-SYN--IMEIRTVAVG--IVAIKGV-ESEYYL-AMNKEGKLYAKKEC--NED 136
Rattus norvegicus Fgf5b         83 GKVK-GT-QEMRN-SYN--IMEIRTVAVG--IVAIKGV-ESEYYL-AMNKEGKLYAKKEC--NED 136
Cavia porcellus FGF5B           83 GKVK-GT-QEMKN-SYN--IMEIRTVAVG--IVAIKGV-ESEYYL-AMNKEGKLYAKKEC--NED 136
Oryctolagus cuniculus FGF5B     83 GKVK-GT-QEMKN-NYN--IMEIRTVAVG--IVAIKGV-ESEYYL-AMNKEGKLYAKKEC--NED 136
Bos taurus FGF5B                83 GKVK-GT-QEMKN-NYN--IMEIRTVAVG--IVAIKGV-ESEYYL-AMNKEGKLYAKKEC--NED 136
Vicugna pacos FGF5B             83 GKVK-GT-QEMKN-NYN--IMEIRTVAVG--IVAIKGV-ESEYYL-AMNKEGKLYAKKEC--NED 136
```

```
Equus caballus FGF5B                 83 GKVK-GT-QEMKN-NFN--IMEIRTVAVG--IVAIKGV-ESEYYL-AMNKEGKLYAKKEC--NED 136
Canis lupus familiaris FGF5B         83 GKVK-GT-QEMKN-SYN--IMEIRTVAVG--IVAIKGV-ESEYYL-AMNKEGKLYAKKEC--NED 136
Myotis lucifugus FGF5B               83 GKVK-GT-QEMKN-NYN--IMEIRTVEVG--IVAIKGV-ESEYFL-AMNKEGKLYAKKEC--NED 136
Sorex araneus FGF5B                  83 GKVK-GT-QEMKN-NYN--IMEIRTVAVG--IVAIKGV-ESEYYL-AMNKDGKLYAKKEC--NED 136
Dasypus novemcinctus FGF5B           83 GKVK-GT-QEMKN-NYN--IMEIKTVAVG--IVAIKGV-ESEYYL-AMNKEGKLYAKKEC--NDD 136
Loxodonta africana FGF5B             83 GKVK-GT-QEMRN-NYN--IMEIRTVAVG--IVAIKGV-ESEYYL-AMNKEGKLYAKKEC--NED 136
Homo sapiens FGF5C                   62 GRVN-GS-LE-NS-AYS--ILEITAVEVG--IVAIRGL-FSGRYL-AMNKRGRLYASEH--YSAE 114
Pongo abelii FGF5C                   62 GRVN-GS-LE-NS-AYS--ILEITAVEVG--IVAIRGL-FSGRYL-AMNKRGRLYASEH--YSAE 114
Macaca mulatta FGF5C                 62 GRVN-GS-LE-NS-AYS--ILEITAVEVG--IVAIRGL-FSGRYL-AMNKRGRLYASEH--YSAE 114
Mus musculus Fgf5c                   62 GRVN-GS-LE-NS-AYS--ILEITAVEVG--VVAIKGL-FSGRYL-AMNKRGRLYASDH--YNAE 114
Rattus norvegicus Fgf5c              62 GRVN-GS-LE-NS-AYS--ILEITAVEVG--VVAIKGL-FSGRYL-AMNKRGRLYASEH--YNAE 114
Cavia porcellus FGF5C                62 GRVN-GS-LE-NS-AYS--ILEITAVEVG--VVAIKGL-FSGRYL-AMNRRGRLYASEH--YSAE 114
Bos taurus FGF5C                     62 GRVN-GS-LE-NS-AYS--ILEITAVEVG--VVAIKGL-FSGRYL-AMNKRGRLYASES--YNAE 114
Homo sapiens FGF5D                   59 GRVQ-GTRWR-HG-QDS--ILEIRSVHVG--VVVIKAV-SSGFYV-AMNRRGRLYGSRL--YTVD 112
Macaca mulatta FGF5D                 59 GRVQ-GTRWR-HG-QDS--ILEIRSVHVG--VVVIKAV-SSGFYV-AMDRRGRLYGSRL--YTVD 112
Papio hamadryas FGF5D                59 GRVQ-GTRWR-HG-KDS--ILEIRSVHVG--VVVIKAV-SSGFYV-AMDRRGCLYGSRL--YTVD 112
Otolemur garnettii FGF5D             56 GRVQ-GTRWR-HS-LEI-GVVEIRSVRVG--VVVIKAA-YSGFYV-AMNRRGRLYGSRV--YTVD 110
Mus musculus Fgf5d                   50 GRVQ-GTRWR-HG-QDS--IVEIRSVRVG--TVVIKAV-YSGFYV-AMNRRGRLYGSRV--YSVD 103
Rattus norvegicus Fgf5d              50 GRVQ-GTRWR-HG-QDS--IVEIRSVRVG--TVVIKAV-YSGFYV-AMNRRGRLYGSRV--YSVD 103
Bos taurus FGF5D                    123 GRVQ-GTRWR-DN-PDS--VLEIRSIRVG--VVVLKAV-HSGFYV-AMNRLGRLYGSRF--CAAH 176
Myotis lucifugus FGF5D               59 GLVQ-GTRWC-HS-PDT-GIIEIRSIRVG--VVALKAV-HTGFFV-AMSRRGHLYGSRI--YTAH 113
Pteropus vampyrus FGF5D              59 GRVQ-GTRWR-HT-PDS--VIEIRSIRVG--VVALRAV-HTGFFV-AMSRRGHLYGSRI--YSAH 112
Homo sapiens FGF6A                   71 RRIS-ARGED-GD-KYAQLLVETDTF--GSQ-VRIKGK-ETEFYLC-MNRKGKLVGKPD-GTSKE 126
Mus musculus Fgf6a                   71 RRIS-ARGED-GD-KYAQLLVETDTF--GSQ-VRIKGK-ETEFYLC-MNRKGKLVGKPD-GTSKE 126
Cavia porcellus FGF6A                71 RRIS-ARGED-GD-KYAQLLVETDTF--GSQ-VRIKGK-ETEFYLC-MNRKGKLVGKPD-GTSKE 126
Bos taurus FGF6A                     71 RRIS-ARGED-GD-KYAQLLVETDTF--GSQ-VRIKGK-ETEFYLC-MNRKGKLVGKPD-GTSKE 126
Canis lupus familiaris FGF6A         71 RRIS-ARGED-GD-KYAQLLVETDTF--GSQ-VRIKGK-ETEFYLC-MNRKGKLVGKPD-GTSKE 126
Homo sapiens FGF6B                   94 RRIS-ATAED-GN-KFAKLIVETDTF--GSR-VRIKGA-ESEKYIC-MNKRGKLIGKPS-GKSKD 149
Pan troglodytes FGF6B                94 RRIS-ATAED-GN-KFAKLIVETDTF--GSR-VRIKGA-ESEKYIC-MNKRGKLIGKPS-GKSKD 149
Nomascus leucogenys FGF6B            94 RRIS-ATAED-GN-KFAKLIVETDTF--GSR-VRIKGA-ESEKYIC-MNKRGKLIGKPS-GKSKD 149
Macaca mulatta FGF6B                188 RRIS-ATAED-GN-KFAKLIVETDTF--GSR-VRIKGA-ESEKYIC-MNKRGKLIGKPS-GKSKD 243
Mus musculus Fgf6b                  131 RRIS-ATAED-GN-KFAKLIVETDTF--GSR-VRIKGA-ESEKYIC-MNKRGKLIGKPS-GKSKD 186
Rattus norvegicus Fgf6b              130 RRIS-ATAED-GN-KFAKLIVETDTF--GSR-VRIKGA-ESEKYIC-MNKRGKLIGKPS-GKSKD 185
Cavia porcellus FGF6B                133 RRIS-ATAED-GN-KFAKLIVETDTF--GSR-VRIKGA-ESEKYIC-MNKKGKLIGKPS-GKSKD 188
Canis lupus familiaris FGF6B         98 RRIS-ATAED-GN-KFAKLIVETDTF--GSR-VRIKGA-ESEKYIC-MNKRGKLIGKPS-GKSKD 153
Felis catus FGF6B                    140 RRIS-ATAED-GN-KFAKLIVETDTF--GSR-VRIKGA-ESEKYIC-MNKRGKLIGKPS-GKSKD 195
Myotis lucifugus FGF6B               68 RRIS-ATAED-GN-KFAKLIVETDTF--GSR-VRIKGA-ESEKYIC-MNKRGKLIGKPS-GKSKD 123
Dasypus novemcinctus FGF6B           29 RRVS-ATAED-GN-KFAKLIVETDTF--GSR-VRIKGA-ESKKYIC-MNKRGKLIGKPS-GKSKD 84
Loxodonta africana FGF6B             87 RRIS-ATAED-GN-KFAKLIVETDTF--GSR-VRIKGA-ESEKYIC-MNKRGKLIGKPS-GKSKD 142
Homo sapiens FGF6C                   188 KRIN-AMAED-GD-PFAKLIVETDTF--GSR-VRVRGA-ETGLYIC-MNKKGKLIAKSN-GKGKD 243
Nomascus leucogenys FGF6C            59 KRIN-AMAED-GD-PFAKLIVETDTF--GSR-VRVRGA-ETGLYIC-MNKKGKLIAKSN-GKGKD 114
Macaca mulatta FGF6C                 59 KRIN-AMAED-GD-PFAKLIVETDTF--GSR-VRVRGA-ETGLYIC-MNKKGKLIAKSN-GKGKD 114
Papio hamadryas FGF6C                59 KRIN-AMAED-GD-PFAKLIVETDTF--GSR-VRVRGA-ETGLYIC-MNKKGKLIAKSN-GKGKD 114
Mus musculus Fgf6c                   59 KRIN-AMAED-GD-PFAKLIVETDTF--GSR-VRVRGA-ETGLYIC-MNKKGKLIAKSN-GKGKD 114
Rattus norvegicus Fgf6c              59 KRIN-AMAED-GD-PFAKLIVETDTF--GSR-VRVRGA-ETGLYIC-MNKKGKLIAKSN-GKGKD 114
Bos taurus FGF6C                     92 KRIN-AMAED-GD-PFAKLIVETDTF--GSR-VRVRGA-ETGLYIC-MNKKGKLIAKSN-GKGKD 147
Homo sapiens FGF7A                   102 GRIG-GAHAD-TR--DS--LLELSPVERG--VVSIFGV-ASRFFV-AMSSKGKLYGSP--FFTDE 154
Macaca mulatta FGF7A                 102 GRIG-GAHAD-TR--DS--LLELSPVERG--VVSIFGV-ASRFFV-AMSSKGKLYGSP--FFTDE 154
Callithrix jacchus FGF7A             102 GRIG-GAHAD-TR--DS--LLELSPVERG--VVSIFGV-ASRFFV-AMSSKGKLYGSP--FFTSE 154
Mus musculus Fgf7a                   98 GRIG-GVHAD-TR--DS--LLELSPVQRG--VVSIFGV-ASRFFV-AMSSRGKLFGVP--FFTDE 150
Rattus norvegicus Fgf7a              98 GRIG-GVHAD-TR--DS--LLELSPVQRG--VVSIFGV-ASRFFV-AMSSRGKLFGVP--FFTDE 150
Cavia porcellus FGF7A                102 GRIG-GVHAD-TA--DS--LLELSPVERG--VVSIFGV-ASRFFV-AMSSKGKLYGSP--FFTDE 154
Canis lupus familiaris FGF7A         102 GRIG-GVHAD-TS--DS--LLELSPVERG--VVSIFGV-ASRFFV-AMSSKGKLYGSP--FFTEE 154
Procavia capensis FGF7A              93 GRIG-GVHAD-TG--DS--LLELSPVERG--VVSIFGV-SSRFFV-AMNSKGKLYGSP--FFTEE 145
Homo sapiens FGF7B                   104 GRIS-GTHEE-NP--YS--LLEISTVERG--VVSLFGV-RSALFV-AMNSKGRLYATP--SFQEE 156
Pan troglodytes FGF7B                104 GRIS-GTHEE-NP--YS--LLEISTVERG--VVSLFGV-RSALFV-AMNSKGRLYATP--SFQEE 156
Pongo abelii FGF7B                   104 GRIS-GTHEE-NP--YS--LLEISTVERG--VVSLFGV-RSALFV-AMNSKGRLYATP--SFQEE 156
Nomascus leucogenys FGF7B            104 GRIS-GTHEE-NP--YS--LLEISTVERG--VVSLFGV-RSALFV-AMNSKGRLYATP--SFQEE 156
Macaca mulatta FGF7B                 104 GRIS-GTHEE-NP--YS--LLEISTVERG--VVSLFGV-RSALFV-AMNSKGRLYATP--SFQEE 156
Papio hamadryas FGF7B                104 GRIS-GTHEE-NP--YS--LLEISTVERG--VVSLFGV-RSALFV-AMNSKGRLYATP--SFREE 156
Callithrix jacchus FGF7B             104 GRIS-GTHKE-TP--YS--LLEISTVERG--VVSLFGV-RSALFI-AMNSEGRLYATP--SFQEE 156
Mus musculus Fgf7b                   104 GRIS-GTHEE-NP--YS--LLEISTVERG--VVSLFGV-KSALFI-AMNSKGRLYTTP--SFHDE 156
Rattus norvegicus Fgf7b              104 GRIS-GTHEE-NP--YS--LLEISTVERG--VVSLFGV-KSALFI-AMNSKGRLYTTP--SFQDE 156
Cavia porcellus FGF7B                104 GHIS-GTHEE-NP--YS--LLEISTVEQG--VVSLFGV-RSALFV-AMNRRGRLYTTP--SFQNE 156
Oryctolagus cuniculus FGF7B          104 GRVS-GVHEE-SP--YS--LLEISTVERG--VVSLFGV-RSALFV-AMNSKGRLYTTP--SFQEE 156
Tursiops truncatus FGF7B             104 GRIS-GTHEE-NP--YS--LLEISTVERG--VVSLFGV-KSALFV-AMNSKGRLYTTP--SFQEE 156
Bos taurus FGF7B                     104 GRIS-GTHEE-NP--YS--LLEISTVERG--VVSLFGV-KSALFV-AMNSKGKLYATP--SFQEE 156
Equus caballus FGF7B                 104 GRIS-GTHEE-NP--YS--LLEISTVERG--VVSLFGV-KSALFI-AMNSKGRLYTTP--SFQEE 156
Canis lupus familiaris FGF7B         104 GRIS-GTHEE-NP--YS--LLEISTVERG--VVSLFGV-KSALFV-AMNSKGRLYTTP--SFQEE 156
Myotis lucifugus FGF7B               104 GRIS-GTHEE-NP--YS--LLEISTVERG--VVSLFGV-KSALFV-AMNSKGRLYTTT--SFQEE 156
Loxodonta africana FGF7B             104 GRVR-GTHEE-NP--YS--LLEISTVERG--VVSLFGV-KSALFI-AMNSKGRLYTTP--SFQEE 156
Homo sapiens FGF8A                   66 GVVDC-ARGQ--S-AHS--LLEIKAVA--LRTVAIKGV-HSVRYLC-MGADGKMQGLL--QYSEE 118
Pongo abelii FGF8A                   66 GVVDC-ARGQ--S-AHS--LLEIKAVA--LRTVAIKGV-HSVRYLC-MGADGKMQGLL--QYSEE 118
Macaca mulatta FGF8A                 66 GVVDC-ARGQ--S-AHS--LLEIKAVA--LRTVAIKGV-HSVRYLC-MGADGKMQGLL--QYSEE 118
Microcebus murinus FGF8A             147 GSVDC-ARGQ--S-AHS--LLEIRAVA--LRTVAIKGV-HSVRYLC-MGADGRMQGLL--RYSEE 199
Otolemur garnettii FGF8A             69 GAVDC-ARGQ--S-AHS--LLEIRAVA--LRTVAIKGV-HSARYLC-MGADGRMQGLP--QYSEE 121
Mus musculus Fgf8a                   73 GSVDC-EEDQ--N-ERN--LLEFRAVA--LKTIAIKDV-SSVRYLC-MSADGKIYGLI--RYSEE 125
Rattus norvegicus Fgf8a              73 GSVDC-EEDQ--N-ERN--LLEFRAVA--LKTIAIKDV-SSVRYLC-MSADGKIYGLI--RYSEE 125
Cavia porcellus FGF8A                74 GAVDC-VEEQ--S-EHC--LLEIRAVA--LETVAIKDI-NSVRYLC-MGPDGRMRGLP--WYSEE 126
```

Bos taurus FGF8A	69	GAVDC-AQVQ--S-AHS--LMEIRAVA--LSTVAIKGE-RSVLYLC-MDADGKMQGLT--QYSAE	121
Myotis lucifugus FGF8A	69	GAVDC-ARGQ--S-AHS--LVEIRAVA--LRKVAIKGV-HSALYLC-MGGDGRMLGLP--QFSPE	121
Pteropus vampyrus FGF8A	66	GAVDC-ARGQ--S-AHS--LVEIRAVA--LRNVAIKGV-HSVRYLC-MGADGRMLGLL--QYSAD	118
Dasypus novemcinctus FGF8A	68	GTVDG-KRSQ--S-AHS--LLEIKAVA--LRTVAIKGV-SSALYLC-MSADGSLQGLP--QYSAE	120
Homo sapiens FGF8B	187	GHVD-GAPHQ--T-IYS--ALMIRSEDAG--FVVITGV-MSRRYLC-MDFRGNIFGSH--YFDPE	239
Pan troglodytes FGF8B	187	GHVD-GAPHQ--T-IYS--ALMIRSEDAG--FVVITGV-MSRRYLC-MDFRGNIFGSH--YFNPE	239
Nomascus leucogenys FGF8B	187	GHVD-GAPHQ--T-IYS--ALMIRSEDAG--FVVITGV-MSRRYLC-MDFRGNIFGSH--YFNPE	239
Otolemur garnettii FGF8B	59	GHVD-GTPHQ--T-IYS--ALMIRSEDAG--FVVITGV-MSRRYLC-MDFKGNIFGSH--SFHPE	111
Mus musculus Fgf8b	59	GHVD-GTPHQ--T-IYS--ALMITSEDAG--SVVITGA-MTRRFLC-MDLHGNIFGSL--HFSPE	111
Rattus norvegicus Fgf8b	132	GHVD-GTPHQ--T-IYS--ALMITSEDAG--SVVIIGA-MTRRFLC-MDLRGNIFGSY--HFSPE	184
Cavia porcellus FGF8B	53	GHID-GAPYP--T-IYS--ALMIRSEDAG--FVVITGV-TSRRFLC-MDFRGNIFGSH--HFNPQ	105
Oryctolagus cuniculus FGF8B	120	GQVD-GTPHQ--T-IYS--ALMIRSEDAG--FVVITGV-MSRRYLC-MDFRGNIFGSH--YFDPQ	172
Equus caballus FGF8B	54	GHVD-GTPHQ--T-IYS--ALMIRSEDAG--FVVITGV-MSRRYLC-MDFRGNIFGSH--HFSPE	106
Canis lupus familiaris FGF8B	121	GHVD-GTPHQ--T-IYS--ALMIRSEDAG--FVVITGV-MSRRYLC-MDFRGNIFGSH--LFSPE	173
Dasypus novemcinctus FGF8B	56	GHVD-GTPQQ--T-IYS--ALMISSEDAG--FVVITGV-MSRRYLC-MDFRGNIFGSH--DFTPD	108
Loxodonta africana FGF8B	118	GHVD-GTPDQ--T-IYS--ALIIRSEEAG--FVVITGV-MSRRYLC-MDFRGNIFGSH--YFNPE	170
Homo sapiens FGF8C	67	GTVG-GAADQ--S-PES--LLQLKALKPG--VIQILGV-KTSRFLCQ-RPDGALYGSL--HFDPE	119
Pan troglodytes FGF8C	67	GTVG-GAADQ--S-PES--LLQLKALKPG--VIQILGV-KTSRFLCQ-RPDGALYGSL--HFDPE	119
Pongo abelii FGF8C	67	GTVG-GAADQ--S-PES--LLQLKALKPG--VIQILGV-KTSRFLCQ-RPDGALYGSL--HFDPE	119
Nomascus leucogenys FGF8C	67	GTVG-GAADQ--S-PES--LLQLKALKPG--VIQILGV-KTSRFLCQ-RPDGALYGSL--HFDPE	119
Otolemur garnettii FGF8C	69	GTVV-GAAQQ--S-PES--LLELKALKPG--VIQILGV-KTSRFLCQ-RPDGGLYGSL--YFDPK	121
Mus musculus Fgf8c	68	GTVV-GAAHR--S-PES--LLELKALKPG--VIQILGV-KASRFLCQ-QPDGALYGSP--HFDPE	120
Rattus norvegicus Fgf8c	68	GTVV-GTAHR--S-PES--LLELKALKPG--VIQILGV-KASRFLCQ-QPDGTLYGSP--HFDPE	120
Cavia porcellus FGF8C	73	GSVR-GIAHR--S-PES--LLELKALKPG--VIQILGI-RTSRFLCQ-RPDGSLYGSL--HFDPE	125
Oryctolagus cuniculus FGF8C	67	GTVV-GAARR--S-PES--LLQMKALQPG--IIQILGV-QTSRFLCQ-RPDGTLYGSL--HFDRE	119
Ochotona princeps FGF8C	63	GTVA-GTARR--S-PES--LLEMKALKPG--VIQILGV-HTSRFLCQ-RPDGTLYGSL--HFDHK	115
Bos taurus FGF8C	67	GTVV-GAARQ--S-PES--LLELKALKPG--VIQILGV-KTSRFLCQ-GPDGKLYGSL--HFDPK	119
Equus caballus FGF8C	67	GTVA-GAVHR--S-PES--LLELKALKPG--VIQILGV-KTSRFLCQ-GPDGTLYGSL--HFDPV	119
Canis lupus familiaris FGF8C	67	GTVV-GAARQ--S-PES--LLELKALKPG--VIQILGV-KTSRFLCQ-GPDGTLYGSL--HFDPV	119
Felis catus FGF8C	68	GTVV-GTARR--S-PES--LLELKALKPG--VIQILGV-KTSRFLCQ-GPDGTLYGSL--RFDPA	120
Sorex araneus FGF8C	67	GTVQ-GAAHR--T-PEC--LLELKALKPG--VIQILGV-STSRFLCQ-RPDGVLYGSL--RFDPE	119
Procavia capensis FGF8C	67	GTVV-GAAHR--S-PES--LLELKALKPG--IIQILGV-KTSRFLCQ-GPDGVLYGSL--RFDPV	119

***Homo sapiens* FGF1A**	145	**-CKFKE-SVFENYYVIY-SSTLYRQQESG----------RAWFLGLNKEGQIMKG--NRVKKTKP**	194
		400 410 420 430 440 450	
***Homo sapiens* FGF1A**	145	-CKFKE-SVFENYYVIY-SSTLYRQQESG----------RAWFLGLNKEGQIMKG--NRVKKTKP	194
***Pan troglodytes* FGF1A**	145	-CKFKE-SVFENYYVIY-SSTLYRQQESG----------RAWFLGLNKEGQIMKG--NRVKKTKP	194
***Nomascus leucogenys* FGF1A**	145	-CKFKE-SVFENYYVIY-SSTLYRQQESG----------RAWFLGLNKEGQIMKG--NRVKKTKP	194
***Callithrix jacchus* FGF1A**	145	-CKFKE-SVFENYYVIY-SSTLYRQQESG----------RAWFLGLNKEGQIMKG--NRVKKTKP	194
***Mus musculus* Fgf1a**	145	-CKFKE-SVFENYYVIY-SSTLYRQQESG----------RAWFLGLNKEGQIMKG--NRVKKTKP	194
***Rattus norvegicus* Fgf1a**	145	-CKFKE-SVFENYYVIY-SSTLYRQQESG----------RAWFLGLNKEGQIMKG--NRVKKTKP	194
***Cavia porcellus* FGF1A**	145	-CKFKE-SVFENYYVIY-SSTLYRQQESG----------RAWFLGLNKEGQIMKG--NRVKKTKP	194
***Oryctolagus cuniculus* FGF1A**	145	-CKFKE-SVFENYYVIY-SSTLYRQQESG----------RAWFLGLNKEGQIMKG--NRVKKTKP	194
***Equus caballus* FGF1A**	145	-CKFKE-SVFENYYVIY-SSTLYRQQESG----------RAWFLGLNKEGQIMKG--NRVKKTKP	194
***Canis lupus familiaris* FGF1A**	145	-CKFKE-SVFENYYVIY-SSTLYRQQESG----------RAWFLGLNKEGQIMKG--NRVKKTKP	194
***Dasypus novemcinctus* FGF1A**	145	-CKFKE-SVFENYYVIY-SSTLYRQQESG----------RAWFLGLNKEGQIMKG--NRVKKTKP	194
***Homo sapiens* FGF1B**	143	-CKFKE-SVFENYYVIY-SSMLYRQQESG----------RAWFLGLNKEGQAMKG--NRVKKTKP	192
***Pan troglodytes* FGF1B**	143	-CKFKE-SVFENYYVIY-SSMLYRQQESG----------RAWFLGLNKEGQAMKG--NRVKKTKP	192
***Pongo abelii* FGF1B**	143	-CKFKE-SVFENYYVIY-SSMLYRQQESG----------RAWFLGLNKEGQAMKG--NRVKKTKP	192
***Macaca mulatta* FGF1B**	143	-CKFKE-SVFENYYVIY-SSMLYRQQESG----------RAWFLGLNKEGQAMKG--NRVKKTKP	192
***Callithrix jacchus* FGF1B**	143	-CKFKE-SVFENYYVIY-SSMLYRQQESG----------RAWFLGLNKEGQAMKG--NRVKKTKP	192
***Mus musculus* Fgf1b**	143	-CKFKE-SVFENYYVIY-SSMLYRQQESG----------RAWFLGLNKEGQVMKG--NRVKKTKP	192
***Oryctolagus cuniculus* FGF1B**	143	-CKFKE-SVFENYYVIY-SSMLYRQQESG----------RAWFLGLNKEGQVMKG--NRVKKTKP	192
***Equus caballus* FGF1B**	143	-CKFKE-SVFENYYVIY-SSMLYRQQESG----------RAWFLGLNKEGQVMKG--NRVKKTKP	192
***Dasypus novemcinctus* FGF1B**	143	-CKFKE-SVFENYYVIY-SSMLYRQQESG----------RAWFLGLNKEGQVMKG--NRVKKTKP	192
***Homo sapiens* FGF1C**	141	-CKFKE-SVFENYYVTY-SSMIYRQQQSG----------RGWYLGLNKEGEIMKG--NHVKKNKP	190
***Pan troglodytes* FGF1C**	141	-CKFKE-SVFENYYVTY-SSMIYRQQQSG----------RGWYLGLNKEGEIMKG--NHVKKNKP	190
***Pongo abelii* FGF1C**	141	-CKFKE-SVFENYYVTY-SSMIYRQQQSG----------RGWYLGLNKEGEIMKG--NHVKKNKP	190
***Macaca mulatta* FGF1C**	141	-CKFKE-SVFENYYVTY-SSMIYRQQQSG----------RGWYLGLNKEGEIMKG--NHVKKNKP	190
***Mus musculus* Fgf1c**	141	-CKFKE-SVFENYYVTY-SSMIYRQQQSG----------RGWYLGLNKEGEIMKG--NHVKKNKP	190
***Rattus norvegicus* Fgf1c**	141	-CKFKE-SVFENYYVTY-SSMIYRQQQSG----------RGWYLGLNKEGEIMKG--NHVKKNKP	190
***Cavia porcellus* FGF1C**	141	-CKFKE-SVFENYYVTY-SSMIYRQQQSG----------RGWYLGLNKEGEIMKG--NHVKKNKP	190
***Bos taurus* FGF1C**	141	-CKFKE-SVFENYYVTY-SSMIYRQQQSG----------RGWYLGLNKEGEIMKG--NHVKKNKP	190
***Myotis lucifugus* FGF1C**	141	-CKFKE-SVFENYYVTY-SSMIYRQQQSG----------RGWYLGLNKEGEIMKG--NHVKKNKP	190
***Dasypus novemcinctus* FGF1C**	141	-CKFKE-SVFENYYVTY-SSMIYRQQQSG----------RGWYLGLNKEGEIMKG--NHVKKNKP	190
***Loxodonta africana* FGF1C**	141	-CKFKE-SVFENYYVTY-SSMIYRQQQSG----------RGWYLGLNKEGEIMKG--NHVKKNKP	190
***Homo sapiens* FGF1D**	143	-CRFKEC-VFENYYVLY-ASALYRQRRSG----------RAWYLGLDKEGQVMKG--NRVKKTKA	192
***Pan troglodytes* FGF1D**	143	-CRFKEC-VFENYYVLY-ASALYRQRRSG----------RAWYLGLDKEGQVMKG--NRVKKTKA	192
***Nomascus leucogenys* FGF1D**	143	-CRFKEC-VFENYYVLY-ASALYRQRRSG----------RAWYLGLDKEGQVMKG--NRVKKTKA	192
***Macaca mulatta* FGF1D**	143	-CRFKEC-VFENYYVLY-ASALYRQRRSG----------RAWYLGLDKEGQVMKG--NRVKKTKA	192
***Otolemur garnettii* FGF1D**	143	-CRFKEC-VFENYYVLY-ASALYRQCRSG----------RAWYLGLDKEGRVMKG--NRVKKTKA	192
***Mus musculus* Fgf1d**	143	-CRFKEC-VFENYYVLY-ASALYRQRRSG----------RAWYLGLDKEGRVMKG--NRVKKTKA	192
***Rattus norvegicus* Fgf1d**	143	-CRFKEC-VFENYYVLY-ASALYRQRRSG----------RAWYLGLDKEGRVMKG--NRVKKTKA	192
***Cavia porcellus* FGF1D**	143	-CRFKEC-VFENYYVLY-ASALYRQRRSG----------RAWYLGLDKEGQVMKG--NRVKKTKA	192
***Oryctolagus cuniculus* FGF1D**	143	-CRFKEC-VFENYYVLY-ASALYRQRRSG----------RAWYLGLDKEGRVMKG--NRVKKTKA	192
***Bos taurus* FGF1D**	143	-CRFKEC-VFENYYVLY-ASALYRQRRSG----------RAWYLGLDKEGRVMKG--NRVKKTKA	192
***Equus caballus* FGF1D**	143	-CRFKEC-VFENYYVLY-ASALYRQ-RSG----------RAWYLGLDKEGRVMKG--NRVKKTKA	191
***Canis lupus familiaris* FGF1D**	143	-CRFKEC-VFENYYVLY-ASALYRQRRSG----------RAWYLGLDKEGRVMKG--NRVKKTKA	192
***Pteropus vampyrus* FGF1D**	143	-CRFKEC-VFENYYVLY-ASALYRQCRSG----------RAWYLGLDKEGRVMKG--NRVKKTKA	192
***Dasypus novemcinctus* FGF1D**	143	-CRFKEC-VFENYYVLY-ASALYRQRSSG----------RAWYLGLDKEGRVMKG--NRVKKTKA	192
***Loxodonta africana* FGF1D**	143	-CRFKEC-VFENYYVLY-ASALYRQCHSG----------RAWYLGLDKEGRVMKG--NRVKKTKA	192
***Homo sapiens* FGF2A**	101	-CFFFE-RLESNNYNTY-RSR--KYT--------------SWYVALKRTGQ------Y--KLGSK	138
***Pongo abelii* FGF2A**	101	-CFFFE-RLESNNYNTY-RSR--KYT--------------SWYVALKRTGQ------Y--KLGSK	138
***Nomascus leucogenys* FGF2A**	101	-CFFFE-RLESNNYNTY-RSR--KYT--------------SWYVALKRTGQ------Y--KLGSK	138
***Macaca mulatta* FGF2A**	101	-CFFFE-RLESNNYNTY-RSR--KYT--------------SWYVALKRTGQ------Y--KLGSK	138
***Mus musculus* Fgf2a**	100	-CFFFE-RLESNNYNTY-RSR--KYS--------------SWYVALKRTGQ------Y--KLGSK	137
***Rattus norvegicus* Fgf2a**	100	-CFFFE-RLESNNYNTY-RSR--KYS--------------SWYVALKRTGQ------Y--KLGSK	137
***Oryctolagus cuniculus* FGF2A**	101	-CFFFE-RLESNNYNTY-RSR--KYS--------------SWYVALKRTGQ------Y--KLGSK	138
***Dasypus novemcinctus* FGF2A**	101	-CFFFE-RLESNNYNTY-RSR--KYS--------------SWYVALKRTGQ------Y--KLGPK	138
***Homo sapiens* FGF2B**	98	-CLFLE-RLEENHYNTY-ISK--KHAEK------------NWFVGLKKNGS----C----KRGPR	137
***Pan troglodytes* FGF2B**	98	-CLFLE-RLEENHYNTY-ISK--KHAEK------------NWFVGLKKNGS----C----KRGPR	137
***Pongo abelii* FGF2B**	98	-CLFLE-RLEENHYNTY-ISK--KHAEK------------NWFVGLKKNGS----C----KRGPR	137
***Nomascus leucogenys* FGF2B**	98	-CLFLE-RLEENHYNTY-ISK--KHAEK------------NWFVGLKKNGS----C----KRGPR	137
***Callithrix jacchus* FGF2B**	98	-CLFLE-RLEENHYNTY-ISK--KHAEK------------NWFVGLKKNGS----C----KRGPR	137
***Tarsius syrichta* FGF2B**	98	-CLFLE-RLEENHYNTY-VSK--KHAEK------------NWFVGLKKNGS----C----KRGPR	137
***Otolemur garnettii* FGF2B**	98	-CLFLE-RLEENHYNTY-VSK--KHAEK------------NWFVGLKKNGS----C----KRGPR	137
***Mus musculus* Fgf2b**	98	-CLFLE-RLEENHYNTY-TSK--KHAEK------------NWFVGLKKNGS----C----KRGPR	137
***Rattus norvegicus* Fgf2b**	98	-CLFLE-RLEENHYNTY-TSK--KHAEK------------NWFVGLKKNGS----C----KRGPR	137
***Cavia porcellus* FGF2B**	98	-CLFLE-RLEENHYNTY-TSK--KHVEK------------NWFVGLKKNGS----C----KRGPR	137
***Oryctolagus cuniculus* FGF2B**	98	-CLFLE-RLEENHYNTY-TSK--KHAEK------------NWFVGLKKNGS----C----KRGPR	137
***Tursiops truncatus* FGF2B**	98	-CLFLE-RLEENHYNTY-ASK--KHAEK------------NWFVGLKKNGS----C----KRGPR	137
***Bos taurus* FGF2B**	98	-CLFLE-RLEENHYNTY-ISK--KHAEK------------HWFVGLKKNGR----S----KLGPR	137
***Equus caballus* FGF2B**	98	-CLFLE-RLEENHYNTY-TSK--KHAEK------------NWFVGLKKNGS----C----KRGPR	137
***Canis lupus familiaris* FGF2B**	98	-CLFLE-RLEENHYNTY-TSK--KHAEK------------NWFVGLKKNGS----C----KRGPR	137
***Felis catus* FGF2B**	98	-CLFLE-RLEENHYNTY-TSK--KHAEK------------NWFVGLKKNGS----C----KRGPR	137
***Myotis lucifugus* FGF2B**	98	-CLFLE-RLEENHYNTY-TSK--KHAEK------------NWFVGLKKNGS----C----KRGPR	137
***Pteropus vampyrus* FGF2B**	98	-CLFLE-RLEENHYNTY-TSK--KHAEK------------NWFVGLKKNGS----C----KRGPR	137
***Dasypus novemcinctus* FGF2B**	98	-CLFME-KLEENNYNTY-ISK--KHAEK------------KWFVGLKKDGS----S----KRGPQ	137
***Loxodonta africana* FGF2B**	98	-CLFLE-RLEENHYNTY-TSK--KHAEK------------NWFVGLKKNGS----C----KRGPR	137
***Homo sapiens* FGF3A**	160	-CKFRE-RFQENSYNTY-ASAI--H-RTE-------KTGREWYVALNKRGKAKRGCSPRVKPQHI	211
***Pan troglodytes* FGF3A**	160	-CKFRE-RFQENSYNTY-ASAI--H-RTE-------KTGREWYVALNKRGKAKRGCSPRVKPQHI	211
***Nomascus leucogenys* FGF3A**	159	-CKFRE-RFQENSYNTY-ASAI--H-RTE-------KTGREWYVALNKRGKAKRGCSPRVKPQHI	210

```
Macaca mulatta FGF3A              160 -CKFRE-RFQENSYNTY-ASAI--H-RTE-------KTGREWYVALNKRGKAKRGCSPRVKPQHI 211
Papio hamadryas FGF3A             160 -CKFRE-RFQENSYNTY-ASAI--H-RTE-------KTGREWYVALNKRGKAKRGCSPRVKPQHI 211
Callithrix jacchus FGF3A          160 -CKFRE-RFQENSYNTY-ASAI--H-RTE-------KTGREWYVALNKRGKAKRGCSPRVKPQHI 211
Otolemur garnettii FGF3A          161 -CKFRE-RFQENSYNTY-ASVI--H-RTE-------TTGREWYVALNKRGKAKRGCSPRVKPQHV 212
Mus musculus Fgf3a                158 -CKFRE-RFQENSYNTY-ASAI--H-RTE-------KTGREWYVALNKRGKAKRGCSPRVKPQHV 209
Rattus norvegicus Fgf3a           158 -CKFRE-RFQENSYNTY-ASAI--H-RTE-------KTGREWYVALNKRGKAKRGCSPRVKPQHV 209
Dipodomys ordii FGF3A             164 -CKFRE-RFQENSYNTY-ASAI--H-RTE-------KTGREWYVALNKRGKAKRGCSPRVKPQHI 215
Cavia porcellus FGF3A             161 -CKFRE-RFQENSYNTY-ASAI--H-RTE-------KTGREWYVALNKRGKAKRGCSPRVKPQHV 212
Oryctolagus cuniculus FGF3A       159 -CKFRE-RFQENSYNTY-ASAI--H-RTE-------RTGREWYVALNKRGKAKRGCSPRVKPQHV 210
Bos taurus FGF3A                  162 -CKFRE-RFQENSYNTY-ASAI--H-RTE-------KTGREWYVALNKRGKAKRGCSPRVKPQHV 213
Equus caballus FGF3A              161 -CKFRE-RFQENSYNTY-ASAI--H-RTE-------KTGREWYVALNKRGKAKRGCSPRVKPQHI 212
Canis lupus familiaris FGF3A      164 -CKFRE-RFQENSYNTY-ASAI--H-RSE-------PAGREWYVALNKRGKAKRGCSPRVKPQHV 215
Myotis lucifugus FGF3A            157 -CKFRE-RFQENSYNTY-ASAI--H-RTE-------NTGREWYVALNKRGKAKRGCSPRVKPQHI 208
Dasypus novemcinctus FGF3A        155 -CKFRE-RFQENCYNTY-ASAI--H-RTE-------KTGREWYVALNKRGKAKRGCSPRVKPQHV 206
Homo sapiens FGF4A                137 -CIFRE-QFEENWYNTY-SSNIYKHGDTG----------RRYFVALNKDGTPRDG--ARSKRHQK 186
Pan troglodytes FGF4A             137 -CIFRE-QFEENWYNTY-SSNIYKHGDTG----------RRYFVALNKDGTPRDG--ARSKRHQK 186
Nomascus leucogenys FGF4A         137 -CIFRE-QFEENWYNTY-SSNIYKHGDTG----------RRYFVALNKDGTPRDG--ARSKRHQK 186
Macaca mulatta FGF4A              137 -CIFRE-QFEENWYNTY-SSNTYKHGDTG----------RRYFVALNKDGTPRDG--ARSKRHQK 186
Papio hamadryas FGF4A             137 -CIFRE-QFEENWYNTY-SSNIYKHGDTG----------RRYFVALNKDGTPRDG--ARSKRHQK 186
Callithrix jacchus FGF4A          137 -CIFRE-QFEENWYNTY-SSNIYKHGDTG----------RRYFVALNKDGTPRDG--ARSKRHQK 186
Mus musculus Fgf4a                137 -CIFRE-QFEENWYNTY-SSNIYKHGDTG----------RRYFVALNKDGTPRDG--ARSKRHQK 186
Rattus norvegicus Fgf4a           137 -CIFRE-QFEENWYNTY-SSNIYKHGDTG----------RRYFVALNKDGTPRDG--ARSKRHQK 186
Bos taurus FGF4A                  137 -CIFRE-QFEENWYNTY-SSNIYKHGDTG----------RRYFVALNKDGTPRDG--ARSKRHQK 186
Vicugna pacos FGF4A               137 -CIFRE-QFEENWYNTY-SSNIYKHGDTG----------RRYFVALNKDGTPRDG--ARSKRHQK 186
Pteropus vampyrus FGF4A           137 -CIFRE-QFEENWYNTY-SSNIYKHGDTG----------RRYFVALNKDGTPRDG--AKSKRHQK 186
Homo sapiens FGF4B                134 -CVFRE-QFEENWYNTY-SSNLYKHVDTG----------RRYYVALNKDGTPREG--TRTKRHQK 183
Pan troglodytes FGF4B             134 -CVFRE-QFEENWYNTY-SSNLYKHVDTG----------RRYYVALNKDGTPREG--TRTKRHQK 183
Gorilla gorilla FGF4B             134 -CVFRE-QFEENWYNTY-SSNLYKHVDTG----------RRYYVALNKDGTPREG--TRTKRHQK 183
Nomascus leucogenys FGF4B         134 -CVFRE-QFEENWYNTY-SSNLYKHVDTG----------RRYYVALNKDGTPREG--TRTKRHQK 183
Callithrix jacchus FGF4B          134 -CVFRE-QFEENWYNTY-SSNIYKHVDTG----------RRYYVALNKDGTPREG--TRTKRHQK 183
Otolemur garnettii FGF4B          134 -CVFRE-QFEENWYNTY-SSNLYKHVDTG----------RRYYVALNKDGTPREG--TRTKRHQK 183
Mus musculus Fgf4b                134 -CVFRE-QFEENWYNTY-SSNLYKHVDTG----------RRYYVALNKDGTPREG--TRTKRHQK 183
Rattus norvegicus Fgf4b           134 -CVFRE-QFEENWYNTY-SSNLYKHVDTG----------RRYYVALNKDGTPREG--TRTKRHQK 183
Oryctolagus cuniculus FGF4B       134 -CVFRE-QFEENWYNTY-SSNLYKHVDTG----------RRYYVALNKDGTPREG--TRTKRHQK 183
Tursiops truncatus FGF4B          134 -CVFRE-QFEENWYNTY-SSNLYKHVDTG----------RRFYVALNKDGTPREG--TRTKRHQK 183
Equus caballus FGF4B              134 -CVFRE-QFEENWYNTY-SSNLYKHVDTG----------RRYYVALNKDGTPREG--TRTKRHQK 183
Canis lupus familiaris FGF4B      134 -CVFRE-QFEENWYNTY-SSNLYKHVDTG----------RRYYVALNKDGTPREG--TRTKRHQK 183
Myotis lucifugus FGF4B            134 -CVFRE-QFEENWYNTY-SSNLYKHVDTG----------RRYYVALNKDGTPREG--TRTKRHQK 183
Pteropus vampyrus FGF4B           134 -CVFRE-QFEENWYNTY-SSNLYKHVDTG----------RRYYVALNKDGTPREG--TRTKRHQK 183
Dasypus novemcinctus FGF4B        134 -CVFRE-QFEENWYNTY-SSNLYKHVDTG----------RRYYVALNKDGTPREG--TRTKRHQK 183
Loxodonta africana FGF4B          134 -CVFRE-QFEENWYNTY-SSNLYKHVDTG----------RRYYVALNKDGTPREG--TRTKRHQK 183
Homo sapiens FGF4C                133 -CVFRE-QFEENWYNTY-ASTLYKHSDSE----------RQYYVALNKDGSPREG--YRTKRHQK 182
Pan troglodytes FGF4C             133 -CVFRE-QFEENWYNTY-ASTLYKHSDSE----------RQYYVALNKDGSPREG--YRTKRHQK 182
Gorilla gorilla FGF4C             133 -CVFRE-QFEENWYNTY-ASTLYKHSDSE----------RQYYVALNKDGSPREG--YRTKRHQK 182
Pongo abelii FGF4C                133 -CVFRE-QFEENWYNTY-ASTLYKHSDSE----------RQYYVALNKDGSPREG--YRTKRHQK 182
Nomascus leucogenys FGF4C         133 -CVFRE-QFEENWYNTY-ASTLYKHSDSE----------RQYYVALNKDGSPREG--YRTKRHQK 182
Otolemur garnettii FGF4C          133 -CVFRE-QFEENWYNTY-ASTLYKHSDSE----------RQYYVALNKDGSPREG--YRTKRHQK 182
Tupaia belangeri FGF4C            133 -CVFRE-QFEENWYNTY-ASTLYKHSDSE----------RQYYVALNKDGSPREG--CRTKRHQK 182
Mus musculus Fgf4c                133 -CVFRE-QFEENWYNTY-ASTLYKHSDSE----------RQYYVALNKDGSPREG--YRTKRHQK 182
Cavia porcellus FGF4C             133 -CVFRE-QFEENWYNTY-ASTLYKHMDSE----------RHYYVALNKDGSPREG--YRTKRHQK 182
Bos taurus FGF4C                  133 -CVFRE-QFEENWYNTY-ASTLYKHSDSE----------RQYYVALNKDGSPREG--YRTKRHQK 182
Canis lupus familiaris FGF4C      133 -CVFRE-QFEENWYNTY-ASTLYKHLDSE----------RQYYVALNKDGSPREG--YRTKRHQK 182
Myotis lucifugus FGF4C            133 -CVFRE-QFEENWYNTY-ASTLYKHLDSE----------RQYYVALNKDGSPREG--YRTKRHQK 182
Pteropus vampyrus FGF4C           133 -CVFRE-QFEENWYNTY-ASTLYKHSDSE----------RQYYVALNKDGSPREG--YRTKRHQK 182
Dasypus novemcinctus FGF4C        133 -CVFRE-QFEENWYNTY-ASTLYKHLDSE----------RQYYVALNKDGSPREG--YRTKRHQK 182
Homo sapiens FGF5A                150 -CKLKE-RIEENGYNTY-ASFNW-Q-HNG----------RQMYVALNGKGAPRRG--QKTRRKNT 197
Pan troglodytes FGF5A             150 -CKLKE-RIEENGYNTY-ASFNW-Q-HNG----------RQMYVALNGKGAPRRG--QKTRRKNT 197
Nomascus leucogenys FGF5A         150 -CKLKE-RIEENGYNTY-ASFNW-Q-HNG----------RQMYVALNGKGAPRRG--QKTRRKNT 197
Macaca mulatta FGF5A              150 -CKLKE-RIEENGYNTY-ASFNW-Q-HNG----------RQMYVALNGKGAPRRG--QKTRRKNT 197
Callithrix jacchus FGF5A          151 -CKLKE-RIEENGYNTY-ASFNW-Q-HNG----------RQMYVALNGKGAPRRG--QKTRRKNT 198
Otolemur garnettii FGF5A          150 -CKLME-RIEENGYNTY-ASFNW-Q-HKG----------RQMYVALNGKGAPRRG--QKTRRKNT 197
Tupaia belangeri FGF5A            149 -CKLKE-RIEENGYNTY-ASFNW-Q-HNG----------RQMYVALNGKGAPRRG--QKTRRKNT 196
Mus musculus Fgf5a                202 -CKLKE-RIEENGYNTY-ASFNW-Q-HNG----------RQMYVALNGKGAPRRG--QKTRRKNT 249
Rattus norvegicus Fgf5a           157 -CKLKE-RIEENGYNTY-ASFNW-Q-HNG----------RQMYVALNGKGAPRRG--QKTRRKNT 204
Cavia porcellus FGF5A             158 -CKLKE-RIEENGYNTY-ASFNW-Q-HNG----------RQMYVALNGKGAPRRG--QKTRRKNT 205
Oryctolagus cuniculus FGF5A       162 -CKLKE-RIEENGYNTY-ASFNW-Q-HNG----------RQMYVALNGKGAPRRG--QKTRRKNT 209
Myotis lucifugus FGF5A            166 -CKLKE-RIEENGYNTY-ASFNW-Q-HNG----------RQMYVALNGKGAPRRG--QKTRRKNT 213
Dasypus novemcinctus FGF5A        156 -CKLKE-RIEENGYNTY-ASFNW-Q-HNG----------RQMYVALNGKGAPRRG--QKTRRKNT 203
Loxodonta africana FGF5A          155 -CKLKE-RIEENGYNTY-ASFNW-Q-HNG----------RQMYVALNGKGAPRRG--QRTRRKNT 202
Homo sapiens FGF5B                137 -CNFKE-LILENHYNTY-ASAKW-T-HNG----------GEMFVALNQKGIPVRG--KKTKKEQK 184
Nomascus leucogenys FGF5B         137 -CNFKE-LILENHYNTY-ASAKW-T-HNG----------GEMFVALNQKGIPVRG--KKTKKEQK 184
Macaca mulatta FGF5B              137 -CNFKE-LILENHYNTY-ASAKW-T-HNG----------GEMFVALNQKGIPVRG--KKTKKEQK 184
Callithrix jacchus FGF5B          137 -CNFKE-LILENHYNTY-ASAKW-T-HNG----------GEMFVALNQKGIPVRG--KKTKKEQK 184
Mus musculus Fgf5b                137 -CNFKE-LILENHYNTY-ASAKW-T-HSG----------GEMFVALNQKGIPVKG--KKTKKEQK 184
Rattus norvegicus Fgf5b           137 -CNFKE-LILENHYNTY-ASAKW-T-HSG----------GEMFVALNQKGLPVKG--KKTKKEQK 184
Cavia porcellus FGF5B             137 -CNFKE-LILENHYNTY-ASAKW-T-HSG----------GEMFVALSHRGVPVKG--KKTKKEQK 184
Oryctolagus cuniculus FGF5B       137 -CNFKE-LILENHYNTY-ASAKW-T-HSG----------GEMFVALNQKGVPVRG--KKTKKEQK 184
Bos taurus FGF5B                  137 -CNFKE-LILENHYNTY-ASAKW-T-HSG----------GEMFVALNQKGVPVRG--KKTKKEQK 184
Vicugna pacos FGF5B               137 -CNFKE-LILENHYNTY-ASAKW-T-HSG----------GEMFVALNQKGVPVRG--KKTKKEQK 184
```

```
Equus caballus FGF5B            137 -CNFKE-LILENHYNTY-ASAKW-T-HSG----------GEMFVALNQKGVPVRG--KKTKKEQK 184
Canis lupus familiaris FGF5B    137 -CNFKE-LILENHYNTY-ASAKW-T-HSG----------GEMFVALNQKGVPVRG--KKTKKEQK 184
Myotis lucifugus FGF5B          137 -CNFKE-LILENHYNTY-ASAKW-T-HGG----------GEMFVALSQKGLPVRG--KKTKKEQK 184
Sorex araneus FGF5B             137 -CNFKE-LILENHYNTY-ASAKW-T-HGG----------GEMFVALNQKGFPVKG--KKTKKEQK 184
Dasypus novemcinctus FGF5B      137 -CNFKE-LILENHYNTY-ASAKW-T-HNG----------GEMFVALNQKGFPVRG--KKTKKEQK 184
Loxodonta africana FGF5B        137 -CNFKE-LILENHYNTY-ASAKW-T-HSG----------GEMFVALNQKGIPVRG--KKTKKEQR 184
Homo sapiens FGF5C              115 -CEFVE-RIHELGYNTY-ASRLYRTVSSTPGARRQPSAERLWYVSVNGKGRPRRG--FKTRRTQK 174
Pongo abelii FGF5C              115 -CEFVE-RIHELGYNTY-ASRLYRTVSSTPGARRQPSAERLWYVSVNGKGRPRRG--FKTRRTQK 174
Macaca mulatta FGF5C            115 -CEFVE-RIHELGYNTY-ASRLYRTVSSTPGARRQPSAERLWYVSVNGKGRPRRG--FKTRRTQK 174
Mus musculus Fgf5c              115 -CEFVE-RIHELGYNTY-ASRLYRTGSSGPGAQRQPGAQRPWYVSVNGKGRPRRG--FKTRRTQK 174
Rattus norvegicus Fgf5c         115 -CEFVE-RIHELGYNTY-ASRLYRTGPSGPGARRQPGAQRPWYVSVNGKGRPRRG--FKTRRTQK 174
Cavia porcellus FGF5C           115 -CEFVE-RIHELGYNTY-ASRLYRTAPSGPGARRQPGTERLWYVSVNGKGRPRRG--FKTRRTQK 174
Bos taurus FGF5C                115 -CEFVE-RIHELGYNTY-ASRLYRTAPSGRGARRQPSAERLWYVSVNGKGRPRRG--FKTRRTQK 174
Homo sapiens FGF5D              113 -CRFRE-RIEENGHNTY-ASQRWR--RRG----------QPMFLALDRRGGPRPG--GRTRRYHL 160
Macaca mulatta FGF5D            113 -CRFRE-RIEENGHNTY-ASQRWH--RHG----------QPMFLALDRRGGPRPG--SRTRRYHL 160
Papio hamadryas FGF5D           113 -CRFRE-RIEENGHNTY-ASQRWH--RHG----------QPMFLALDRRGGPRPG--GRTRRYHL 160
Otolemur garnettii FGF5D        111 -CRFQE-RIEENGYNTY-AALRWR--HHG----------QPMFLALDRRGAPRPG--GRTQRHHL 158
Mus musculus Fgf5d              104 -CRFRE-RIEENGYNTY-ASRRWR--HRG----------RPMFLALDSQGIPRQG--RRTRRHQL 151
Rattus norvegicus Fgf5d         104 -CRFRE-RIEENGYNTY-ASRRWR--HHG----------RPMFLALDSQGIPRQG--RRTRRHQL 151
Bos taurus FGF5D                177 -CRFRE-RIEENGYNTY-ASVRWR--HQG----------RPMFLALDGRGAPRLG--GRTQRHHP 224
Myotis lucifugus FGF5D          114 -CRFQE-RIEENGYNTY-ASLRWR--HRG----------RPMFLALDRRGVPRRG--GRTRRHHL 161
Pteropus vampyrus FGF5D         113 -CRFRE-RIEENGYNTY-ASLRWR--QRG----------RPMFLALDGRGAPRRG--DRTRRHHL 160
Homo sapiens FGF6A              127 -CVFIE-KVLEN--N-YTALMSAKY-S-G------------WYVGFTKKGRPRKG--PKTRENQQ 170
Mus musculus Fgf6a              127 -CVFIE-KVLEN--N-YTALMSAKY-S-G------------WYVGFTKKGRPRKG--PKTRENQQ 170
Cavia porcellus FGF6A           127 -CVFIE-KVLEN--N-YTALMSAKY-S-G------------WYVGFTKKGRPRKG--PKTRENQQ 170
Bos taurus FGF6A                127 -CVFIE-KVLEN--N-YTALMSAKY-S-G------------WYVGFTKKGRPRKG--PKTRENQQ 170
Canis lupus familiaris FGF6A    127 -CVFIE-KVLEN--N-YTALMSAKY-S-G------------WYVGFTKKGRPRKG--PKTRENQQ 170
Homo sapiens FGF6B              150 -CVFTE-IVLEN--N-YTAFQNARH-E-G------------WFMAFTRQGRPRQA--SRSRQNQR 193
Pan troglodytes FGF6B           150 -CVFTE-IVLEN--N-YTAFQNARH-E-G------------WFMAFTRQGRPRQA--SRSRQNQR 193
Nomascus leucogenys FGF6B       150 -CVFTE-IVLEN--N-YTAFQNARH-E-G------------WFMAFTRQGRPRQA--SRSRQNQR 193
Macaca mulatta FGF6B            244 -CVFTE-IVLEN--N-YTAFQNARH-E-G------------WFMAFTRQGRPRQA--SRSRQNQR 287
Mus musculus Fgf6b              187 -CVFTE-IVLEN--N-YTAFQNARH-E-G------------WFMAFTRQGRPRQA--SRSRQNQR 230
Rattus norvegicus Fgf6b         186 -CVFTE-IVLEN--N-YTAFQNARH-E-G------------WFMAFTRQGRPRQA--SRSRQNQR 229
Cavia porcellus FGF6B           189 -CVFTE-IVLEN--N-YTAFQNARH-E-G------------WFMAFTRQGRPRQA--SRSRQNQR 232
Canis lupus familiaris FGF6B    154 -CVFTE-IVLEN--N-YTAFQNARH-E-G------------WFMAFTRQGRPRQA--SRSRQNQR 197
Felis catus FGF6B               196 -CVFTE-IVLEN--N-YTAFQNARH-E-G------------WFMAFTRQGRPRQA--SRSRQNQR 239
Myotis lucifugus FGF6B          124 -CVFTE-IVLEN--N-YTAFQNARH-E-G------------WFMAFTRQGRPRQA--SRSRQNQR 167
Dasypus novemcinctus FGF6B       85 -CVFTE-IVLEN--N-YTAFQNARH-E-G------------WFMAFTRQGRPRQA--SRSRQNQR 128
Loxodonta africana FGF6B        143 -CVFTE-IVLEN--N-YTAFQNARH-E-G------------WFMAFTRQGRPRQA--SRSRQNQR 186
Homo sapiens FGF6C              244 -CVFTE-IVLEN--N-YTALQNAKY-E-G------------WYMAFTRKGRPRKG--SKTRQHQR 287
Nomascus leucogenys FGF6C       115 -CVFTE-IVLEN--N-YTALQNAKY-E-G------------WYMAFTRKGRPRKG--SKTRQHQR 158
Macaca mulatta FGF6C            115 -CVFTE-IVLEN--N-YTALQNAKY-E-G------------WYMAFTRKGRPRKG--SKTRQHQR 158
Papio hamadryas FGF6C           115 -CVFTE-IVLEN--N-YTALQNAKY-E-G------------WYMAFTRKGRPRKG--SKTRQHQR 158
Mus musculus Fgf6c              115 -CVFTE-IVLEN--N-YTALQNAKY-E-G------------WYMAFTRKGRPRKG--SKTRQHQR 158
Rattus norvegicus Fgf6c         115 -CVFTE-IVLEN--N-YTALQNAKY-E-G------------WYMAFTRKGRPRKG--SKTRQHQR 158
Bos taurus FGF6C                148 -CVFTE-IVLEN--N-YTALQNAKY-E-G------------WYMAFTRKGRPRKG--SKTRQHQR 191
Homo sapiens FGF7A              155 -CTFKE-ILLPNNYNAY-ESY--KYP--G------------MFIALSKNGKTKKG-N-RVSPTMK 198
Macaca mulatta FGF7A            155 -CKFKE-ILLPNNYNAY-ESY--KYP--G------------MFIALSKNGKTKKG-N-RVSPTMK 198
Callithrix jacchus FGF7A        155 -CRFKE-ILLPNNYNAY-ESY--KYP--S------------MFIALRKNGKTKNG-N-RVSPTNK 198
Mus musculus Fgf7a              151 -CKFKE-ILLPNNYNAY-ESY--AYP--G------------MFMALSKNGRTKKG-N-RVSPTMK 194
Rattus norvegicus Fgf7a         151 -CKFKE-ILLPNNYNAY-ESY--AYP--G------------MFMALSKNGRTKKG-N-RVSPTMK 194
Cavia porcellus FGF7A           155 -CKFKE-TLLPNNYNAY-ESH--SYA--G------------MFIALSKNGRTKKG-N-RVSPTMK 198
Canis lupus familiaris FGF7A    155 -CKFKE-ILLPNNYNAY-ECY--RYP--G------------MFIALSKNGKTKKG-S-RVSPTMK 198
Procavia capensis FGF7A         146 -CMFRE-TLLPNNYNAY-ESY--KFP--G------------AFIALSKNGKTKKG-N-RVSPTMK 189
Homo sapiens FGF7B              157 -CKFRE-TLLPNNYNAY-ESD--LYQ--G------------TYIALSKYGRVKRG-S-KVSPIMT 200
Pan troglodytes FGF7B           157 -CKFRE-TLLPNNYNAY-ESD--LYR--G------------TYIALSKYGRVKRG-S-KVSPIMT 200
Pongo abelii FGF7B              157 -CKFRE-TLLPNNYNAY-ESD--LYR--G------------TYIALSKYGRVKRG-S-KVSPIMT 200
Nomascus leucogenys FGF7B       157 -CKFRE-TLLPNNYNAY-ESD--LYR--G------------TYIALSKYGRVKRG-S-KVSPIMT 200
Macaca mulatta FGF7B            157 -CKFRE-TLLPNNYNAY-ESD--LYR--G------------TYIALSKYGRVKRG-S-KVSPTMT 200
Papio hamadryas FGF7B           157 -CKFRE-TLLPNNYNAY-ESD--LYR--G------------TYIALSKHGRVKRG-S-KVSPTMT 200
Callithrix jacchus FGF7B        157 -CKFRE-TLLPNNYNAY-ESD--LYR--G------------TYIALSKYGRAKRG-S-KVSLIMT 200
Mus musculus Fgf7b              157 -CKFRE-TLLPNNYNAY-ESD--LYR--G------------TYIALSKYGRVKRG-S-KVSPIMT 200
Rattus norvegicus Fgf7b         157 -CKFRE-TLLPNNYNAY-ESD--LYR--G------------TYIALSKYGRVKRG-S-KVSPIMT 200
Cavia porcellus FGF7B           157 -CKFRE-IILPNNYNAY-ESD--LFR--G------------TYIALSKYGRVKRG-N-KVSPMMT 200
Oryctolagus cuniculus FGF7B     157 -CKFRE-TLLPNNYNAY-ESD--LYR--G------------TYIALSKYGRVKRG-S-KVSPIMT 200
Tursiops truncatus FGF7B        157 -CKFRE-TLLPNNYNAY-ESD--LYR--G------------TYIALSKYGRVKRG-S-KVSPIMT 200
Bos taurus FGF7B                157 -CKFRE-TLLPNNYNAY-ESD--LYR--G------------AYIALSKYGRVKRG-S-KVSPTMT 200
Equus caballus FGF7B            157 -CKFRE-TLLPNNYNAY-ESD--LYR--G------------TYIALSKYGRVKRG-S-KVSPIMT 200
Canis lupus familiaris FGF7B    157 -CKFRE-TLLPNNYNAY-ESD--LYR--G------------TYIALSKYGRVKRG-S-KVSPIMT 200
Myotis lucifugus FGF7B          157 -CKFRE-TLLPNNYNAY-ESD--LYR--G------------TYIALSKYGRVKRG-S-KVSPIMT 200
Loxodonta africana FGF7B        157 -CRFRE-TLLPNNYNAY-ESD--LYR--G------------TYIALSKYGRVKRG-N-KVSPIMT 200
Homo sapiens FGF8A              119 DCAFEE-EIRPDGYNVY-RSE--KHR-------------LPVSLSSAKQ---RQL--Y-KNRG-- 158
Pongo abelii FGF8A              119 DCAFEE-EIRPDGYNVY-RSE--KHR-------------LPVSLSSAKQ---RQL--Y-KNRG-- 158
Macaca mulatta FGF8A            119 DCAFEE-EIRPDGYNVY-RSE--KHR-------------LPVSLSSAKQ---RQL--Y-KNRG-- 158
Microcebus murinus FGF8A        200 DCAFEE-EIRPDGYNVY-RSE--KHR-------------LPVSLSSARQ---RQL--Y-KGRG-- 239
Otolemur garnettii FGF8A        122 DCAFEE-EIRPDGYNVY-WSE--KHR-------------LPVSLSSARQ---RQL--Y-KGRG-- 161
Mus musculus Fgf8a              126 DCTFRE-EMDCLGYNQY-RSM--KHH-------------LHIIFIQAKP---REQ--L-QDQK-- 165
Rattus norvegicus Fgf8a         126 DCTFRE-EMDCLGYNQY-RSM--KHH-------------LHIIFIKAKP---REQ--L-QGQK-- 165
Cavia porcellus FGF8A           127 DCAFKE-EISYPGYSVY-RSQ--KHH-------------LPIVLSSVKQ---RQQ--Y-QSKG-- 166
```

```
Bos taurus FGF8A                122 DCAFEE-EIRPDGYNVY-WSR--KHH-------------LPVSLSSSRQ---RQL--F-KSRG--  161
Myotis lucifugus FGF8A          122 DCAFEE-EIRPDGYNVY-RSQ--KHQ-------------LPVSLSSARQ---RQL--F-KARG--  161
Pteropus vampyrus FGF8A         119 DCAFEE-EIRPDGYNVY-HSK--KHH-------------LPVSLSSAKQ---RQL--Y-KDRG--  158
Dasypus novemcinctus FGF8A      121 DCAFEE-EIRPDGYNVY-WSR--KHG-------------LPVSLSSAKQ---RQL--Y-RGRG--  160
Homo sapiens FGF8B              240 NCRFQH-QTLENGYDVY-HSP--QYH-------------FLVSLGRAKR--AF-L--PGMNPPP-  281
Pan troglodytes FGF8B           240 NCRFQH-QTLENGYDVY-YSP--QYH-------------FLVSLGRAKR--AF-L--PSMNPPP-  281
Nomascus leucogenys FGF8B       240 NCRFQH-QTLENGYDVY-HSP--QHH-------------FLVSLGRAKR--AF-L--PGMNPPP-  281
Otolemur garnettii FGF8B        112 SCRFRH-RTLENGYDVY-LSP--QHH-------------FLVSLGRSKR--PF-L--PGMNPPP-  153
Mus musculus Fgf8b              112 NCKFRQ-WTLENGYDVY-LSQ--KHH-------------YLVSLGRAKR--IF-Q--PGTNPPP-  153
Rattus norvegicus Fgf8b         185 NCRFRQ-WTLENGYDVY-LSP--KHH-------------YLVSLGRSKR--IF-Q--PGTNPPP-  226
Cavia porcellus FGF8B           106 DCRFQH-RTLENGYDVY-LSP--EHH-------------FLISLGRTKK--FF-L--PGTNPPP-  147
Oryctolagus cuniculus FGF8B     173 NCRFRH-RTLENGYDVY-HSP--EHH-------------FLVSLGRAKR--PF-L--PGMNPPP-  214
Equus caballus FGF8B            107 SCSFRQ-RTLENGYDVY-HSP--QHR-------------FLVSLGRAKR--AF-L--PGTNPPP-  148
Canis lupus familiaris FGF8B    174 SCRFRQ-RTLENGYDVY-HSP--QHR-------------FLVSLGQAKR--AF-L--PGTNPPP-  215
Dasypus novemcinctus FGF8B      109 SCRFRQ-RTLENGYDVY-HSP--QHH-------------FLVSLGRAKR--AF-Q--PGSNPPP-  150
Loxodonta africana FGF8B        171 NCRFKH-WTLENGYDVY-HSP--QHH-------------FLVSLGRVKK--AF-L--PGMNPPP-  212
Homo sapiens FGF8C              120 ACSFRE-LLLEDGYNVY-QSE--AHG-------------LPLHL-PGNK-SPHRD--P-APRGP-  161
Pan troglodytes FGF8C           120 ACSFRE-LLLEDGYNVY-QSE--AHG-------------LPLHL-PGNK-SPHRD--P-APRGP-  161
Pongo abelii FGF8C              120 ACSFRE-LLLEDGYNVY-QSE--AHG-------------LPLHL-PGNK-SPHRD--P-APRGP-  161
Nomascus leucogenys FGF8C       120 ACSFRE-LLLEDGYNVY-QSE--AHG-------------LPLHL-PGNK-SPHRD--P-APRGP-  161
Otolemur garnettii FGF8C        122 ACSFRE-LLLEDGYNVY-WSE--TYG-------------LPLHL-PPAN-SPYWG--P-SLRSP-  163
Mus musculus Fgf8c              121 ACSFRE-LLLEDGYNVY-QSE--AHG-------------LPLRL-PQKD-SPNQD--A-TSWGP-  162
Rattus norvegicus Fgf8c         121 ACSFRE-LLLKDGYNVY-QSE--AHG-------------LPLRL-PQKD-S--QD--P-ATRGP-  160
Cavia porcellus FGF8C           126 ACSFRE-LLLADGYNVY-KSE--AHG-------------LPLHL-LRGD-SLSQE--P-APPGP-  167
Oryctolagus cuniculus FGF8C     120 ACSFRE-LLREDGYNVY-LSE--ALG-------------LPLRL-SPGS-SPRRA--P-APRGP-  161
Ochotona princeps FGF8C         116 ACSFRE-QLLEDGYNVY-HSE--THG-------------LPLRL-------SARD--R-APRGP-  152
Bos taurus FGF8C                120 ACSFRE-LLLEDGYNVY-QSE--TLG-------------LPLRL-PPQR-SSNRD--P-APRGP-  161
Equus caballus FGF8C            120 ACSFRE-LLLEDGYNVY-QSE--TLG-------------LPLRL-PHHS-SPYQD--P-APRAP-  161
Canis lupus familiaris FGF8C    120 ACSFRE-LLLEDGYNIY-HSE--TLG-------------LPLRL-RPHN-SAYRD--L-APRGP-  161
Felis catus FGF8C               121 ACSFRE-LLLEDGYNIY-HSE--TLG-------------LPLRL-PPHN-SPYRD--L-APRAP-  162
Sorex araneus FGF8C             120 ACSFRE-LLLQDGYNVY-QSE--ALG-------------LPLYLHPPSA-PVSQE--P-ASRGA-  162
Procavia capensis FGF8C         120 ACSFRE-LLLEDGYNVY-QSE--AHG-------------LPLRL-PSHN-SPQRD--L-ASRVP-  161
```

```
Homo sapiens FGF1A          195 SSHFVPKPIEVC-MYREPSLH--EIG-EKQGRS------RK-S-SGT----PTMNGGKVV----- 238
                                  460       470       480       490       500       510       520
Homo sapiens FGF1A          195 SSHFVPKPIEVC-MYREPSLH--EIG-EKQGRS------RK-S-SGT----PTMNGGKVV----- 238
Pan troglodytes FGF1A       195 SSHFVPKPIEVC-MYREPSLH--EIG-EKQGRS------RK-S-SGT----PTMNGGKVV----- 238
Nomascus leucogenys FGF1A   195 SSHFVPKPIEVC-MYREPSLH--EIG-EKQGRS------RK-S-SGT----PTMNGGKVV----- 238
Callithrix jacchus FGF1A    195 SSHFVPKPIEVC-MYREPSLH--EIG-EKQGRS------RK-S-SGT----PTMNGGKVV----- 238
Mus musculus Fgf1a          195 SSHFVPKPIEVC-MYREPSLH--EIG-EKQGRS------RK-S-SGT----PTMNGGKVV----- 238
Rattus norvegicus Fgf1a     195 SSHFVPKPIEVC-MYREPSLH--EIG-EKQGRS------RK-S-SGT----PTMNGGKVV----- 238
Cavia porcellus FGF1A       195 SSHFVPKPIEVC-MYREPSLH--EIG-EKQGRS------RK-S-SGT----PTMNGGKVV----- 238
Oryctolagus cuniculus FGF1A 195 SSHFVPKPIEVC-MYREPSLH--EIG-EKQGRS------RK-S-SGT----PTMNGGKVV----- 238
Equus caballus FGF1A        195 SSHFVPKPIEVC-MYREPSLH--EIG-EKQGRS------RK-S-SGT----PTMNGGKVV----- 238
Canis lupus familiaris FGF1A 195 SSHFVPKPIEVC-MYREPSLH--EIG-EKQGRS------RK-S-SGT----PTMNGGKVV----- 238
Dasypus novemcinctus FGF1A  195 SSHFVPKPIEVC-MYREPSLH--EIG-EKQGRS------RK-S-SGT----PTMNGGKVV----- 238
Homo sapiens FGF1B          193 AAHFLPKPLEV-AMYREPSLH--DVG-ETVPKPG-V-TPSK-STSAS----AIMNGGKPV----- 241
Pan troglodytes FGF1B       193 AAHFLPKPLEV-AMYREPSLH--DVG-ETVPKPG-V-TPSK-STSAS----AIMNGGKPV----- 241
Pongo abelii FGF1B          193 AAHFLPKPLEV-AMYREPSLH--DVG-ETVPKPG-V-TPSK-STSAS----AIMNGGKPV----- 241
Macaca mulatta FGF1B        193 AAHFLPKPLEV-AMYREPSLH--DVG-ETVPKPG-V-TPSK-STSAS----AIMNGGKPV----- 241
Callithrix jacchus FGF1B    193 AAHFLPKPLEV-AMYREPSLH--DVG-ETVPKPG-V-TPSK-STSAS----AIMNGGKPV----- 241
Mus musculus Fgf1b          193 AAHFLPKPLEV-AMYREPSLH--DVG-ETVPKAG-V-TPSK-STSAS----AIMNGGKPV----- 241
Oryctolagus cuniculus FGF1B 193 AAHFLPKPLEV-AMYREPSLH--DVG-ETVPKAG-V-TPSK-STSAS----AIMNGGKPV----- 241
Equus caballus FGF1B        193 AAHFLPKPLEV-AMYREPSLH--DVG-ETVPKAG-V-TPSK-STSAS----AIMNGGKPV----- 241
Dasypus novemcinctus FGF1B  193 AAHFLPKPLEV-AMYREPSLH--DVG-ETVPKPG-V-TPSK-STSAS----AIMNGGKPV----- 241
Homo sapiens FGF1C          191 AAHFLPKPLKV-AMYKEPSLH--DLT-EFS-RSGSG-TPTK-SRSVS----GVLNGGKSMS---H 241
Pan troglodytes FGF1C       191 AAHFLPKPLKV-AMYKEPSLH--DLT-EFS-RSGSG-TPTK-SRSVS----GVLNGGKSMS---H 241
Pongo abelii FGF1C          191 AAHFLPKPLKV-AMYKEPSLH--DLT-EFS-RSGSG-TPTK-SRSVS----GVLNGGKSMS---H 241
Macaca mulatta FGF1C        191 AAHFLPKPLKV-AMYKEPSLH--DLT-EFS-RSGSG-TPTK-SRSVS----GVLNGGKSMS---H 241
Mus musculus Fgf1c          191 AAHFLPKPLKV-AMYKEPSLH--DLT-EFS-RSGSG-TPTK-SRSVS----GVLNGGKSMS---H 241
Rattus norvegicus Fgf1c     191 AAHFLPKPLKV-AMYKEPSLH--DLT-EFS-RSGSG-TPTK-SRSVS----GVLNGGKSMS---H 241
Cavia porcellus FGF1C       191 AAHFLPKPLKV-AMYKEPSLH--DLT-EFS-RSGSG-TPTK-SRSVS----GVLNGGKSMS---H 241
Bos taurus FGF1C            191 AAHFLPKPLKV-AMYKEPSLH--DLT-EFS-RSGSG-TPTK-SRSVS----GVLNGGKSMS---H 241
Myotis lucifugus FGF1C      191 AAHFLPKPLKV-AMYKEPSLH--DLT-EFS-RSGSG-TPTK-SRSVS----GVLNGGKSMS---H 241
Dasypus novemcinctus FGF1C  191 AAHFLPKPLKV-AMYKEPSLH--DLT-EFS-RSGSG-TPTK-SRSVS----GVLNGGKSMS---H 241
Loxodonta africana FGF1C    191 AAHFLPKPLKV-AMYKEPSLH--DLT-EFS-RSGSG-TPTK-SRSVS----GVLNGGKSMS---H 241
Homo sapiens FGF1D          193 AAHFLPKLLEV-AMYQEPSLH--SVP-EASP-S-S--PPAP------------------------ 225
Pan troglodytes FGF1D       193 AAHFLPKLLEV-AMYQEPSLH--SVP-EASP-S-S--PPAP------------------------ 225
Nomascus leucogenys FGF1D   193 AAHFLPKLLEV-AMYREPSLH--SVP-EASP-S-S--PPAP------------------------ 225
Macaca mulatta FGF1D        193 AAHFLPKLMEV-AMYREPSLH--SVP-EASP-S-S--PPAP------------------------ 225
Otolemur garnettii FGF1D    193 AAHFVPKLLEV-AMYREPSLH--SVP-ETSP-S-S--LPAP------------------------ 225
Mus musculus Fgf1d          193 AAHFVPKLLEV-AMYREPSLH--SVP-ETSP-S-S--PPAH------------------------ 225
Rattus norvegicus Fgf1d     193 AAHFVPKLLEV-AMYREPSLH--SVP-ETSP-S-S--PPAH------------------------ 225
Cavia porcellus FGF1D       193 AAHFVPKLLEV-AMYREPSLH--SVP-ETSP-S-S--PPAP------------------------ 225
Oryctolagus cuniculus FGF1D 193 AAHFVPKLLEV-AMYREPSLH--SVP-ETSP-S-S--PPAP------------------------ 225
Bos taurus FGF1D            193 AAHFVPKLLEV-AMYREPSLH--SVP-ETSP-S-S--PPAP------------------------ 225
Equus caballus FGF1D        192 AAHFVPKLLEV-AMYREPSLH--SVP-ETSP-S-S--PPAL------------------------ 224
Canis lupus familiaris FGF1D 193 AAHFVPKLLEV-AMYREPSLH--SVP-ETSP-P-S--PPAP------------------------ 225
Pteropus vampyrus FGF1D     193 AAHFVPKLLEV-AMYREPSLH--SVP-ETSP-S-S--HPAP------------------------ 225
Dasypus novemcinctus FGF1D  193 AAHFVPKLLEV-AMYREPSLH--SVP-ETSP-S-S--PPAP------------------------ 225
Loxodonta africana FGF1D    193 AAHFVPKLLEV-AMYREPSLH--SVP-ETSP-S-S--PPAP------------------------ 225
Homo sapiens FGF2A          139 -TGPGQK-A---ILF---------------------LPM-S-AKS-------------------- 155
Pongo abelii FGF2A          139 -TGPGQK-A---ILF---------------------LPM-S-AKS-------------------- 155
Nomascus leucogenys FGF2A   139 -TGPGQK-A---ILF---------------------LPM-S-AKS-------------------- 155
Macaca mulatta FGF2A        139 -TGPGQK-A---ILF---------------------LPM-S-AKS-------------------- 155
Mus musculus Fgf2a          138 -TGPGQK-A---ILF---------------------LPM-S-AKS-------------------- 154
Rattus norvegicus Fgf2a     138 -TGPGQK-A---ILF---------------------LPM-S-AKS-------------------- 154
Oryctolagus cuniculus FGF2A 139 -TGPGQK-A---ILF---------------------LPM-S-AKS-------------------- 155
Dasypus novemcinctus FGF2A  139 -TGPGQK-A---ILF---------------------LPM-S-AKS-------------------- 155
Homo sapiens FGF2B          138 -THYGQK-A---ILF---------------------LPL-P-VSSD------------------- 155
Pan troglodytes FGF2B       138 -THYGQK-A---ILF---------------------LPL-P-VSSD------------------- 155
Pongo abelii FGF2B          138 -THYGQK-A---ILF---------------------LPL-P-VSSD------------------- 155
Nomascus leucogenys FGF2B   138 -THYGQK-A---ILF---------------------LPL-P-VSSD------------------- 155
Callithrix jacchus FGF2B    138 -THYGQK-A---ILF---------------------LPL-P-VSSD------------------- 155
Tarsius syrichta FGF2B      138 -THYGQK-A---ILF---------------------LPL-P-VSSD------------------- 155
Otolemur garnettii FGF2B    138 -THYGQK-A---ILF---------------------LPL-P-VSSD------------------- 155
Mus musculus Fgf2b          138 -THYGQK-A---ILF---------------------LPL-P-VSSD------------------- 155
Rattus norvegicus Fgf2b     138 -THYGQK-A---ILF---------------------LPL-P-VSSD------------------- 155
Cavia porcellus FGF2B       138 -THYGQK-A---ILF---------------------LPL-P-V-SD------------------- 154
Oryctolagus cuniculus FGF2B 138 -THYGQK-A---ILF---------------------LPL-P-VSSD------------------- 155
Tursiops truncatus FGF2B    138 -THYGQK-A---ILF---------------------LPL-P-VSSD------------------- 155
Bos taurus FGF2B            138 -THFGQK-A---ILF---------------------LPL-P-VSSD------------------- 155
Equus caballus FGF2B        138 -THYGQK-A---ILF---------------------LPL-P-VSSD------------------- 155
Canis lupus familiaris FGF2B 138 -THYGQK-A---ILF---------------------LPL-P-VSSD------------------- 155
Felis catus FGF2B           138 -THYGQK-A---ILF---------------------LPL-P-VSSD------------------- 155
Myotis lucifugus FGF2B      138 -THYGQK-A---ILF---------------------LPL-P-VSSD------------------- 155
Pteropus vampyrus FGF2B     138 -THYGQK-A---ILF---------------------LPL-P-VSSD------------------- 155
Dasypus novemcinctus FGF2B  138 -THYGQK-A---ILF---------------------LPL-P-VSSD------------------- 155
Loxodonta africana FGF2B    138 -THYGQK-A---ILF---------------------LPL-P-VSSD------------------- 155
Homo sapiens FGF3A          212 STHFLPRFKQS-EQ-PELS-FTVTVP-EKK-K-----PPSPIKPKIP-LSAPRKNTNSV-K---- 260
Pan troglodytes FGF3A       212 STHFLPRFKQS-EQ-PELS-FTVTVP-EKK-K-----PPSPIKPKIP-LSAPRKNTNSV-K---- 260
Nomascus leucogenys FGF3A   211 STHFLPRFKQS-EQ-PELS-FTVTVP-EKK-K-----PPSPIKPKVP-LSTPRKNTNSV-K---- 259
```

```
Macaca mulatta FGF3A          212 STHFLPRFKQS-EQ-PELS-FTVTVP-EKK-K-----PPSPIKPKVP-LSAPRKNTNTV-K---- 260
Papio hamadryas FGF3A         212 STHFLPRFKQS-EQ-PELS-FTVTVP-EKK-K-----PPSPIKPKVP-LSAPRKNTNTV-K---- 260
Callithrix jacchus FGF3A      212 STHFLPRFKHS-EQ-PELS-FTVTVP-EKK-K-----PPSPIKPKVP-LSAPRKTPNTV-K---- 260
Otolemur garnettii FGF3A      213 STHFLPRFKQS-EQ-PELS-FTVTVP-EKK-K-----PPTPTKPKVP-LSVPRKTPKTV-K---- 261
Mus musculus Fgf3a            210 STHFLPRFKQS-EQ-PELS-FTVTVP-EKK-K-----PP--VKPKVP-LSQPRRSPSPV-K---- 256
Rattus norvegicus Fgf3a       210 STHFLPRFKQS-EQ-PELS-FTVTVP-EKK-K-----PPSPVKPKVP-LSPPRRSPSPV-K---- 258
Dipodomys ordii FGF3A         216 STHFLPRFKQS-EQ-PELS-FTVTVP-EKK-K-----PPNPVKPKVP-LSTPRKNPNTV-K---- 264
Cavia porcellus FGF3A         213 STHFLPRFKQS-EQ-PELS-FTVTVP-EKK-K-----PPNPVKPKVP-LSTSRRSPNTG-K---- 261
Oryctolagus cuniculus FGF3A   211 STHFLPRFKQS-EQ-PELS-FTVTVP-EKK-K-----PPNPIKPKVP-LSAPRRSPNTV-K---- 259
Bos taurus FGF3A              214 STHFLPRFKQL-EQ-PELS-FTVTVP-EKK-K-----PPNPVKPKVP-LSAPRRSPNTV-K---- 262
Equus caballus FGF3A          213 STHFLPRFKQL-EQ-PELS-FTVTVP-EKK-K-----APNPAKPKVP-LSAPRRSPNTV-K---- 261
Canis lupus familiaris FGF3A  216 STHFLPRFKQL-EH-PELS-FTVTVP-EKK-K-----PPSHVKPKVP-LSAPRKSPNTV-K---- 264
Myotis lucifugus FGF3A        209 STHFLPRFKQS-EQ-PELS-FTVTVP-EKK-K-----PPSPAKPKVPTLSAPRKSPNTV-K---- 258
Dasypus novemcinctus FGF3A    207 STHFLPRFKQL-EQ-PELS-FTVTVP-EKK-K-----PPNPVKPKVS-LSAPRKSPNTV-K---- 255
Homo sapiens FGF4A            187 FTHFLPR-P-V-D--PE-RV--PELYKDL-L-MYT------------------------------ 211
Pan troglodytes FGF4A         187 FTHFLPR-P-V-D--PE-RV--PELYKDL-L-MYT------------------------------ 211
Nomascus leucogenys FGF4A     187 FTHFLPR-P-V-D--PE-RV--PELYKDL-L-MYT------------------------------ 211
Macaca mulatta FGF4A          187 FTHFLPR-P-V-D--PE-RV--PELYKDL-L-MYT------------------------------ 211
Papio hamadryas FGF4A         187 FTHFLPR-P-V-D--PE-RV--PELYKDL-L-MYT------------------------------ 211
Callithrix jacchus FGF4A      187 FTHFLPR-P-V-D--PE-RV--PELYKDL-L-MYT------------------------------ 211
Mus musculus Fgf4a            187 FTHFLPR-P-V-D--PE-RV--PELYKDL-L-MYT------------------------------ 211
Rattus norvegicus Fgf4a       187 FTHFLPR-P-V-D--PE-RV--PELYKDL-L-VYTG----------------------------- 212
Bos taurus FGF4A              187 FTHFLPR-P-V-D--PE-RV--PELYKDL-L-MYS------------------------------ 211
Vicugna pacos FGF4A           187 FTHFLPR-P-V-D--PE-RV--PELYKDL-L-MYS------------------------------ 211
Pteropus vampyrus FGF4A       187 FTHFLPR-P-V-D--PE-RV--PELYKDI-L-IYS------------------------------ 211
Homo sapiens FGF4B            184 FTHFLPR-P-V-D--PD-KV--PELYKDI-L-SQS------------------------------ 208
Pan troglodytes FGF4B         184 FTHFLPR-P-V-D--PD-KV--PELYKDI-L-SQS------------------------------ 208
Gorilla gorilla FGF4B         184 FTHFLPR-P-V-D--PD-KV--PELYKDI-L-SQS------------------------------ 208
Nomascus leucogenys FGF4B     184 FTHFLPR-P-V-D--PD-KV--PELYKDI-L-SQS------------------------------ 208
Callithrix jacchus FGF4B      184 FTHFLPR-P-V-D--PD-KV--PELYKDI-L-SQS------------------------------ 208
Otolemur garnettii FGF4B      184 FTHFLPR-P-V-D--PD-KV--PELYKDI-L-SQS------------------------------ 208
Mus musculus Fgf4b            184 FTHFLPR-P-V-D--PD-KV--PELYKDI-L-SQS------------------------------ 208
Rattus norvegicus Fgf4b       184 FTHFLPR-P-V-D--PD-KV--PELYKDI-L-SQS------------------------------ 208
Oryctolagus cuniculus FGF4B   184 FTHFLPR-P-V-D--PD-KV--PELYKDI-L-SQS------------------------------ 208
Tursiops truncatus FGF4B      184 FTHFLPR-P-V-D--PD-KV--PELYKGI-L-SQS------------------------------ 208
Equus caballus FGF4B          184 FTHFLPR-P-V-D--PD-KV--PELYKDI-L-SQS------------------------------ 208
Canis lupus familiaris FGF4B  184 FTHFLPR-P-V-D--PD-KV--PELYKDI-L-SQS------------------------------ 208
Myotis lucifugus FGF4B        184 FTHFLPR-P-V-D--PD-KV--PELYKDI-L-SQS------------------------------ 208
Pteropus vampyrus FGF4B       184 FTHFLPR-P-V-D--PD-KV--PELYKDI-L-SQS------------------------------ 208
Dasypus novemcinctus FGF4B    184 FTHFLPR-P-V-D--PD-KV--PELYKDI-L-SQS------------------------------ 208
Loxodonta africana FGF4B      184 FTHFLPR-P-V-D--PD-KV--PELYKDI-L-SQS------------------------------ 208
Homo sapiens FGF4C            183 FTHFLPR-P-V-D--PS-KL--PSMSRDL-F-HYR------------------------------ 207
Pan troglodytes FGF4C         183 FTHFLPR-P-V-D--PS-KL--PSMSRDL-F-HYR------------------------------ 207
Gorilla gorilla FGF4C         183 FTHFLPR-P-V-D--PS-KL--PSMSRDL-F-HYR------------------------------ 207
Pongo abelii FGF4C            183 FTHFLPR-P-V-D--PS-KL--PSMSRDL-F-HYR------------------------------ 207
Nomascus leucogenys FGF4C     183 FTHFLPR-P-V-D--PS-KL--PSMSRDL-F-HYR------------------------------ 207
Otolemur garnettii FGF4C      183 FTHFLPR-P-V-E--PS-KL--PFLSRDL-F-HYR------------------------------ 207
Tupaia belangeri FGF4C        183 FTHFLPR-P-V-D--PS-KL--PSLSRDL-F-RYR------------------------------ 207
Mus musculus Fgf4c            183 FTHFLPR-P-V-D--PS-KL--PSMSRDL-F-RYR------------------------------ 207
Cavia porcellus FGF4C         183 FTHFLPR-P-V-D--PS-KL--PSISRDL-F-HYR------------------------------ 207
Bos taurus FGF4C              183 FTHFLPR-P-V-D--PS-KL--PTMSRDL-F-RYR------------------------------ 207
Canis lupus familiaris FGF4C  183 FTHFLPR-P-V-D--PS-KL--PSMSRDL-F-RYR------------------------------ 207
Myotis lucifugus FGF4C        183 FTHFLPR-P-V-D--PS-KL--PSMSRDL-F-HYR------------------------------ 207
Pteropus vampyrus FGF4C       183 FTHFLPR-P-V-D--PS-KL--PSMSRDL-F-RYR------------------------------ 207
Dasypus novemcinctus FGF4C    183 FTHFLPR-P-V-D--PS-KL--PSMSRDL-F-RYR------------------------------ 207
Homo sapiens FGF5A            198 SAHFLPM-V-V----HS------------------------------------------------ 208
Pan troglodytes FGF5A         198 SAHFLPM-V-V----HS------------------------------------------------ 208
Nomascus leucogenys FGF5A     198 SAHFLPM-V-V----HS------------------------------------------------ 208
Macaca mulatta FGF5A          198 SAHFLPM-V-V----HS------------------------------------------------ 208
Callithrix jacchus FGF5A      199 SAHFLPM-V-V----HS------------------------------------------------ 209
Otolemur garnettii FGF5A      198 SAHFLPM-V-V----QS------------------------------------------------ 208
Tupaia belangeri FGF5A        197 SAHFLPM-V-V----HS------------------------------------------------ 207
Mus musculus Fgf5a            250 SAHFLPM-T-I----QT------------------------------------------------ 260
Rattus norvegicus Fgf5a       205 SAHFLPM-V-V----HS------------------------------------------------ 215
Cavia porcellus FGF5A         206 SAHFLPM-V-V----HS------------------------------------------------ 216
Oryctolagus cuniculus FGF5A   210 SAHFLPM-V-V----HS------------------------------------------------ 220
Myotis lucifugus FGF5A        214 SAHFLPM-V-V----HS------------------------------------------------ 224
Dasypus novemcinctus FGF5A    204 SAHFLPM-V-V----HS------------------------------------------------ 214
Loxodonta africana FGF5A      203 SAHFLPM-V-V----HS------------------------------------------------ 213
Homo sapiens FGF5B            185 TAHFLPM-A-I-----T------------------------------------------------ 194
Nomascus leucogenys FGF5B     185 TAHFLPM-A-I-----T------------------------------------------------ 194
Macaca mulatta FGF5B          185 TAHFLPM-A-I-----T------------------------------------------------ 194
Callithrix jacchus FGF5B      185 TAHFLPM-A-I-----T------------------------------------------------ 194
Mus musculus Fgf5b            185 TAHFLPM-A-I-----T------------------------------------------------ 194
Rattus norvegicus Fgf5b       185 TAHFLPM-A-I-----T------------------------------------------------ 194
Cavia porcellus FGF5B         185 TAHFLPM-A-V-----T------------------------------------------------ 194
Oryctolagus cuniculus FGF5B   185 TAHFLPM-A-I-----T------------------------------------------------ 194
Bos taurus FGF5B              185 TAHFLPM-A-I-----T------------------------------------------------ 194
Vicugna pacos FGF5B           185 TAHFLPM-A-I-----T------------------------------------------------ 194
```

Equus caballus FGF5B	185	TAHFLPM-A-I-----T--	194
Canis lupus familiaris FGF5B	185	TAHFLPM-A-I-----T--	194
Myotis lucifugus FGF5B	185	TAHFLPM-A-I-----T--	194
Sorex araneus FGF5B	185	TAHFLPM-A-I-----T--	194
Dasypus novemcinctus FGF5B	185	TVHFLPL-P-I-----S--	194
Loxodonta africana FGF5B	185	IAHFLPM-A-V-----T--	194
Homo sapiens FGF5C	175	SSLFLPR-V-L-D--HR-D-H--EMVRQL----QSGLPRPPGKGVQP----RRRRQ-KQ-SP-D-	218
Pongo abelii FGF5C	175	SSLFLPR-V-L-D--HR-D-H--EMVRQL----QGGLPRPPGKGVQP----RRRRQ-KQ-SP-D-	218
Macaca mulatta FGF5C	175	SSLFLPR-V-L-D--HR-D-H--EMVRQL----QGGLPRPPGKGVQP----RRRRQ-KQ-SLG--	218
Mus musculus Fgf5c	175	SSLFLPR-V-L-G--HK-D-H--EMVRLL----QSSQPRAPGEGSQP----RQRRQKKQ-SPSDH	221
Rattus norvegicus Fgf5c	175	SSLFLPR-V-L-G--HK-D-H--EMVRLL----QSGQPQAPGEGSQP----RQRRQKKQ-SPGDH	221
Cavia porcellus FGF5C	175	SSLFLPR-V-L-D--HK-D-H--ELVRLL----QSSPVQAPGKGVQP----RRRHRKKQ-NPGAR	221
Bos taurus FGF5C	175	SSLFLPR-V-L-D--RK-D-H--EMVRLL----LGT---AGLRGGQA----RPPPPGR----AAS	215
Homo sapiens FGF5D	161	SAHFLPV-L-V-----S--	170
Macaca mulatta FGF5D	161	SAHFLPV-L-V-----S--	170
Papio hamadryas FGF5D	161	SAHFLPV-L-V-----S--	170
Otolemur garnettii FGF5D	159	STHFLPV-L-V-----S--	168
Mus musculus Fgf5d	152	STHFLPV-L-V----SS--	162
Rattus norvegicus Fgf5d	152	STHFLPV-L-V----SS--	162
Bos taurus FGF5D	225	STLFLPV-L-V-----S--	234
Myotis lucifugus FGF5D	162	STHFLPV-M-V-----S--	171
Pteropus vampyrus FGF5D	161	STHFLPV-L-V-----S--	170
Homo sapiens FGF6A	171	DVHFMKRYPKG-Q---------PELQKPFKY--TT----V--TKRSR-----RI-R-----P-TH	205
Mus musculus Fgf6a	171	DVHFMKRYPKG-Q---------AELQKPFKY--TT----V--TKRSR-----RI-R-----P-TH	205
Cavia porcellus FGF6A	171	DVHFMKRYPKG-Q---------AELQKPFKY--TT----V--TKRSR-----RI-R-----P-TH	205
Bos taurus FGF6A	171	DVHFMKRYPKG-Q---------AELQKPFKY--TT----V--TKRSR-----RI-R-----P-TH	205
Canis lupus familiaris FGF6A	171	DVHFMKRYPKG-Q---------AELQKPFKY--TT----V--TKRSR-----RI-R-----P-TH	205
Homo sapiens FGF6B	194	EAHFIKRLYQG-QL-PFPN-H-AEKQKQFEF-VGS----AP-TRRTK-----RTRR-----P-Q-	236
Pan troglodytes FGF6B	194	EAHFIKRLYQG-QL-PFPN-H-AEKQKQFEF-VGS----AP-TRRTK-----RTRR-----P-Q-	236
Nomascus leucogenys FGF6B	194	EAHFIKRLYQG-QL-PFPN-H-AEKQKQFEF-VGS----AP-TRRTK-----RTRR-----P-Q-	236
Macaca mulatta FGF6B	288	EAHFIKRLYQG-QL-PFPN-H-AEKQKQFEF-VGS----AP-TRRTK-----RTRR-----P-Q-	330
Mus musculus Fgf6b	231	EAHFIKRLYQG-QL-PFPN-H-AERQKQFEF-VGS----AP-TRRTK-----RTRR-----P-Q-	273
Rattus norvegicus Fgf6b	230	EAHFIKRLYQG-QL-PFPN-H-AERQKQFEF-VGS----AP-TRRTK-----RTRR-----P-Q-	272
Cavia porcellus FGF6B	233	EAHFIKRLYQG-QL-PFPN-H-AERQKQFEF-VGS----AP-TRRTK-----RTRG-----P-Q-	275
Canis lupus familiaris FGF6B	198	EAHFIKRLYQG-QL-PFPN-H-AERQKQFEF-VGS----AP-TRRTK-----RTRR-----P-Q-	240
Felis catus FGF6B	240	EAHFIKRLYQG-QL-PFPN-H-AERQKQFEF-VGS----AP-TRRTK-----RTRR-----P-Q-	282
Myotis lucifugus FGF6B	168	EAHFIKRLYEG-QL-PFPN-Q-VERQKQFEF-VGS----AP-TRRTK-----RTRR-----P-Q-	210
Dasypus novemcinctus FGF6B	129	EAHFIKRLHQG-RL-PFPN-H-ADRQKQFEF-VGS----AP-TRRTK-----RTRR-----P-Q-	171
Loxodonta africana FGF6B	187	EAHFIKRLYQG-QL-PFPN-H-AERQKQFEF-VGS----AP-TRRTK-----RTRR-----P-Q-	229
Homo sapiens FGF6C	288	EVHFMKRLPRG-H-------HTTEQSLRFEF-LNY----PPFTRSLR-----GSQRTWA--P-E-	330
Nomascus leucogenys FGF6C	159	EVHFMKRLPRG-H-------HTTEQSLRFEF-LNY----PPFTRSLR-----GSQRTWA--P-E-	201
Macaca mulatta FGF6C	159	EVHFMKRLPRG-H-------HTTEQSLRFEF-LNY----PPFTRSLR-----GSQRTWA--P-E-	201
Papio hamadryas FGF6C	159	EVHFMKRLPRG-H-------HTTEQSLRFEF-LNY----PPFTRSLR-----GSQRTWA--P-E-	201
Mus musculus Fgf6c	159	EVHFMKRLPRG-H-------HTTEQSLRFEF-LNY----PPFTRSLR-----GSQRTWA--P-E-	201
Rattus norvegicus Fgf6c	159	EVHFMKRLPRG-H-------HTTEQSLRFEF-LNY----PPFTRSLR-----GSQRTWA--P-E-	201
Bos taurus FGF6C	192	EVHFMKRLPRG-H-------HTTEQSLRFEF-LNY----PPFTRSLR-----GSQRTWA--P-E-	234
Homo sapiens FGF7A	199	VTHFLPRL---	206
Macaca mulatta FGF7A	199	VTHFLPRL---	206
Callithrix jacchus FGF7A	199	VTHFLPRL---	206
Mus musculus Fgf7a	195	VTHFLPRL---	202
Rattus norvegicus Fgf7a	195	VTHFLPRL---	202
Cavia porcellus FGF7A	199	VTHFLPRL---	206
Canis lupus familiaris FGF7A	199	VTHFLPRL---	206
Procavia capensis FGF7A	190	VTHFLPRL---	197
Homo sapiens FGF7B	201	VTHFLPRI---	208
Pan troglodytes FGF7B	201	VTHFLPRI---	208
Pongo abelii FGF7B	201	VTHFLPRI---	208
Nomascus leucogenys FGF7B	201	VTHFLPRI---	208
Macaca mulatta FGF7B	201	VTHFLPRI---	208
Papio hamadryas FGF7B	201	VTHFLPRI---	208
Callithrix jacchus FGF7B	201	VTHFLPRM---	208
Mus musculus Fgf7b	201	VTHFLPRI---	208
Rattus norvegicus Fgf7b	201	VTHFLPRI---	208
Cavia porcellus FGF7B	201	ATHFLPRI---	208
Oryctolagus cuniculus FGF7B	201	VTHFLPRI---	208
Tursiops truncatus FGF7B	201	VTHFLPRI---	208
Bos taurus FGF7B	201	VTHFLPRI---	208
Equus caballus FGF7B	201	VTHFLPRI---	208
Canis lupus familiaris FGF7B	201	VTHFLPRI---	208
Myotis lucifugus FGF7B	201	VTHFLPRI---	208
Loxodonta africana FGF7B	201	VTHFLPRI---	208
Homo sapiens FGF8A	159	---FL----------P-LS-HFL--------------PMLP-MV-----PEEPEDLRGHLE----	184
Pongo abelii FGF8A	159	---FL----------P-LS-HFL--------------PMLP-MV-----PEEPEDLRRHLE----	184
Macaca mulatta FGF8A	159	---FL----------P-LS-HFL--------------PMLP-MA-----PEEPEDLRGHLE----	184
Microcebus murinus FGF8A	240	---FL----------P-LS-HFL--------------PMLP-VT-----PAETGDLRDHLE----	265
Otolemur garnettii FGF8A	162	---FL----------P-LS-HFL--------------PMLP-VT-----PAEPGDLRDHLE----	187
Mus musculus Fgf8a	166	---------------P--S-NFI--------------PVFH-RS-----FFETGD---QLR----	185
Rattus norvegicus Fgf8a	166	---------------P--S-NFI--------------PIFH-RS-----FFESTD---QLR----	185
Cavia porcellus FGF8A	167	---VV----------P-LS-YFL--------------PMLP-KA-----SVEPSD---EEE----	189

```
Bos taurus FGF8A               162 ---FL----------P-LS-HFL--------------PMLS-TI-----PAEPEDLQEPLK---- 187
Myotis lucifugus FGF8A         162 ---FL----------P-LS-HFL--------------PMLP-SS-----PAGPVPRERPSE---- 187
Pteropus vampyrus FGF8A        159 ---FL----------P-LS-HFL--------------PMLP-RS-----PTEPENFEDHLE---- 184
Dasypus novemcinctus FGF8A     161 ----L----------P-LS-HFL--------------PMLS-VT-----PAEPRERGGDAE---- 185
Homo sapiens FGF8B             282 YSQFLSR--RN-EI-P-LI-HFNT-------------P-IP-RRHTR-SAED-DSERDPLNVLK- 322
Pan troglodytes FGF8B          282 YSQFLSR--RN-EI-P-LI-HFNT-------------P-IP-RRHTR-SAED-DSERDPLNVLK- 322
Nomascus leucogenys FGF8B      282 YSQFLSR--RN-EI-P-LL-HFNT-------------P-TP-RRHTR-SAED-DSERDPLNVLK- 322
Otolemur garnettii FGF8B       154 FSQFLSR--RN-DI-P-LI-HFNT-------------P-RP-RRHTR-SAEDDDSELDPLNVLK- 195
Mus musculus Fgf8b             154 FSQFLAR--RN-EV-P-LL-HFYT-------------V-RP-RRHTR-SAED-PPERDPLNVLK- 194
Rattus norvegicus Fgf8b        227 FSQFLAR--RN-EV-P-LL-HFYT-------------A-RP-RRHTR-SAED-PPERDPLNVLK- 267
Cavia porcellus FGF8B          148 YSQFLSR--RN-EL-P-LA-RFVT-------------P-GP-RRHTR-SAEE-DQGRDPLSVLK- 188
Oryctolagus cuniculus FGF8B    215 YSQFLSR--RN-EI-P-LI-HFNT-------------P-RP-RRHTR-SAED-AWEQDPLNVLK- 255
Equus caballus FGF8B           149 YSQFLSR--RN-EI-P-LV-HFNT-------------P-RP-RRHTR-SAED-NSERDPLNVLK- 189
Canis lupus familiaris FGF8B   216 YSQFLSR--RN-EI-P-LV-HFHT-------------P-RP-RRHTR-SAE--APERDPLNVLK- 255
Dasypus novemcinctus FGF8B     151 YSQFLSR--RN-EI-P-LM-RFST-------------P-RP-RRHTR-SAQD-HADPDPLRVLK- 191
Loxodonta africana FGF8B       213 YSQFLSR--RN-EI-P-LI-YFNT-------------P-KP-RRHTR-SAED-DSERDPLNVLK- 253
Homo sapiens FGF8C             162 -ARFL----------P-L-------------------PGLP-PA----LPEPP-GILAP------ 183
Pan troglodytes FGF8C          162 -ARFL----------P-L-------------------PGLP-PA----PPEPP-GILAP------ 183
Pongo abelii FGF8C             162 -ARFL----------P-L-------------------PGLP-PA----PPEPP-GILAP------ 183
Nomascus leucogenys FGF8C      162 -ARFL----------P-L-------------------PGLP-PA----PPEPP-GILAP------ 183
Otolemur garnettii FGF8C       164 -ARFL----------P-L-------------------PGPP-AA----SPELP-GILAL------ 185
Mus musculus Fgf8c             163 -VRFL----------P-M-------------------PGLL-HE----PQDQA-GFLPP------ 184
Rattus norvegicus Fgf8c        161 -VRFL----------P-M-------------------PGLP-HE----PQEQP-GVLPP------ 182
Cavia porcellus FGF8C          168 -ARFL----------P-L-------------------PGLP-AT----PPEPP-RMLPP------ 189
Oryctolagus cuniculus FGF8C    162 -ARFL----------P-L-------------------PGLP-PD----LPEPP-GLLAA------ 183
Ochotona princeps FGF8C        153 -ARFL----------P-L-------------------PGPP-PDLLV-PPLPP-DVLAP------ 177
Bos taurus FGF8C               162 -ARFL----------P-L-------------------PGLP-AA----PPDPP-GILAP------ 183
Equus caballus FGF8C           162 -ARFL----------P-L-------------------PGFP-PA----PPEPP-GIPAP------ 183
Canis lupus familiaris FGF8C   162 -ARFL----------P-L-------------------PGLL-PA----PPEPP-GILAP------ 183
Felis catus FGF8C              163 -ARFL----------P-L-------------------PGLL-PA----PPEPP-GILAP------ 184
Sorex araneus FGF8C            163 -VRFL----------P-L-------------------PGLP-PA----SLEPP-RPPAP------ 184
Procavia capensis FGF8C        162 -ARFL----------P-L-------------------PGRL-TV----LPEPS-GVLGP------ 183
```

```
Homo sapiens FGF1A            239 -NQD-ST------------------------------------------------------& 243
                                         530       540       550       560       570       580
Homo sapiens FGF1A            239 -NQD-ST------------------------------------------------------& 243
Pan troglodytes FGF1A         239 -NQD-ST------------------------------------------------------& 243
Nomascus leucogenys FGF1A     239 -NQD-ST------------------------------------------------------& 243
Callithrix jacchus FGF1A      239 -NQD-ST------------------------------------------------------& 243
Mus musculus Fgf1a            239 -NQD-ST------------------------------------------------------& 243
Rattus norvegicus Fgf1a       239 -NQD-ST------------------------------------------------------& 243
Cavia porcellus FGF1A         239 -NQD-ST------------------------------------------------------& 243
Oryctolagus cuniculus FGF1A   239 -NQD-ST------------------------------------------------------& 243
Equus caballus FGF1A          239 -NQD-ST------------------------------------------------------& 243
Canis lupus familiaris FGF1A  239 -NQD-ST------------------------------------------------------& 243
Dasypus novemcinctus FGF1A    239 -NQD-ST------------------------------------------------------& 243
Homo sapiens FGF1B            242 -NKSKTT------------------------------------------------------& 247
Pan troglodytes FGF1B         242 -NKSKTT------------------------------------------------------& 247
Pongo abelii FGF1B            242 -NKSKTT------------------------------------------------------& 247
Macaca mulatta FGF1B          242 -NKSKTT------------------------------------------------------& 247
Callithrix jacchus FGF1B      242 -NKSKTT------------------------------------------------------& 247
Mus musculus Fgf1b            242 -NKCKTT------------------------------------------------------& 247
Oryctolagus cuniculus FGF1B   242 -NKSKTT------------------------------------------------------& 247
Equus caballus FGF1B          242 -NKSKTT------------------------------------------------------& 247
Dasypus novemcinctus FGF1B    242 -SKSKTT------------------------------------------------------& 247
Homo sapiens FGF1C            242 -NES--T------------------------------------------------------& 245
Pan troglodytes FGF1C         242 -NES--T------------------------------------------------------& 245
Pongo abelii FGF1C            242 -NES--T------------------------------------------------------& 245
Macaca mulatta FGF1C          242 -NES--T------------------------------------------------------& 245
Mus musculus Fgf1c            242 -NES--T------------------------------------------------------& 245
Rattus norvegicus Fgf1c       242 -NES--T------------------------------------------------------& 245
Cavia porcellus FGF1C         242 -NES--T------------------------------------------------------& 245
Bos taurus FGF1C              242 -NES--T------------------------------------------------------& 245
Myotis lucifugus FGF1C        242 -NES--T------------------------------------------------------& 245
Dasypus novemcinctus FGF1C    242 -NES--T------------------------------------------------------& 245
Loxodonta africana FGF1C      242 -NES--T------------------------------------------------------& 245
Homo sapiens FGF1D              - -------------------------------------------------------------&   -
Pan troglodytes FGF1D           - -------------------------------------------------------------&   -
Nomascus leucogenys FGF1D       - -------------------------------------------------------------&   -
Macaca mulatta FGF1D            - -------------------------------------------------------------&   -
Otolemur garnettii FGF1D        - -------------------------------------------------------------&   -
Mus musculus Fgf1d              - -------------------------------------------------------------&   -
Rattus norvegicus Fgf1d         - -------------------------------------------------------------&   -
Cavia porcellus FGF1D           - -------------------------------------------------------------&   -
Oryctolagus cuniculus FGF1D     - -------------------------------------------------------------&   -
Bos taurus FGF1D                - -------------------------------------------------------------&   -
Equus caballus FGF1D            - -------------------------------------------------------------&   -
Canis lupus familiaris FGF1D    - -------------------------------------------------------------&   -
Pteropus vampyrus FGF1D         - -------------------------------------------------------------&   -
Dasypus novemcinctus FGF1D      - -------------------------------------------------------------&   -
Loxodonta africana FGF1D        - -------------------------------------------------------------&   -
Homo sapiens FGF2A              - -------------------------------------------------------------&   -
Pongo abelii FGF2A              - -------------------------------------------------------------&   -
Nomascus leucogenys FGF2A       - -------------------------------------------------------------&   -
Macaca mulatta FGF2A            - -------------------------------------------------------------&   -
Mus musculus Fgf2a              - -------------------------------------------------------------&   -
Rattus norvegicus Fgf2a         - -------------------------------------------------------------&   -
Oryctolagus cuniculus FGF2A     - -------------------------------------------------------------&   -
Dasypus novemcinctus FGF2A      - -------------------------------------------------------------&   -
Homo sapiens FGF2B              - -------------------------------------------------------------&   -
Pan troglodytes FGF2B           - -------------------------------------------------------------&   -
Pongo abelii FGF2B              - -------------------------------------------------------------&   -
Nomascus leucogenys FGF2B       - -------------------------------------------------------------&   -
Callithrix jacchus FGF2B        - -------------------------------------------------------------&   -
Tarsius syrichta FGF2B          - -------------------------------------------------------------&   -
Otolemur garnettii FGF2B        - -------------------------------------------------------------&   -
Mus musculus Fgf2b              - -------------------------------------------------------------&   -
Rattus norvegicus Fgf2b         - -------------------------------------------------------------&   -
Cavia porcellus FGF2B           - -------------------------------------------------------------&   -
Oryctolagus cuniculus FGF2B     - -------------------------------------------------------------&   -
Tursiops truncatus FGF2B        - -------------------------------------------------------------&   -
Bos taurus FGF2B                - -------------------------------------------------------------&   -
Equus caballus FGF2B            - -------------------------------------------------------------&   -
Canis lupus familiaris FGF2B    - -------------------------------------------------------------&   -
Felis catus FGF2B               - -------------------------------------------------------------&   -
Myotis lucifugus FGF2B          - -------------------------------------------------------------&   -
Pteropus vampyrus FGF2B         - -------------------------------------------------------------&   -
Dasypus novemcinctus FGF2B      - -------------------------------------------------------------&   -
Loxodonta africana FGF2B        - -------------------------------------------------------------&   -
Homo sapiens FGF3A            261 -YRLKFR-FG---------------------------------------------------& 268
Pan troglodytes FGF3A         261 -YRLKFR-FG---------------------------------------------------& 268
Nomascus leucogenys FGF3A     260 -YRLKFR-FG---------------------------------------------------& 267
```

Macaca mulatta FGF3A	261	-YRLKFR-FG--&	268
Papio hamadryas FGF3A	261	-YRLKFR-FG--&	268
Callithrix jacchus FGF3A	261	-YRLKFR-FG--&	268
Otolemur garnettii FGF3A	262	-YRLKFR-FG--&	269
Mus musculus Fgf3a	257	-YRLKFR-FG--&	264
Rattus norvegicus Fgf3a	259	-YRLKFR-FG--&	266
Dipodomys ordii FGF3A	265	-YRLKFR-FG--&	272
Cavia porcellus FGF3A	262	-YRLKFR-FG--&	269
Oryctolagus cuniculus FGF3A	260	-YRLKFR-FG--&	267
Bos taurus FGF3A	263	-YRLKFR-FG--&	270
Equus caballus FGF3A	262	-YRLKFR-FG--&	269
Canis lupus familiaris FGF3A	265	-YRLKFR-FG--&	272
Myotis lucifugus FGF3A	259	-YRLKFR-FG--&	266
Dasypus novemcinctus FGF3A	256	-YRLKFR-FG--&	263
Homo sapiens FGF4A	-	--&	-
Pan troglodytes FGF4A	-	--&	-
Nomascus leucogenys FGF4A	-	--&	-
Macaca mulatta FGF4A	-	--&	-
Papio hamadryas FGF4A	-	--&	-
Callithrix jacchus FGF4A	-	--&	-
Mus musculus Fgf4a	-	--&	-
Rattus norvegicus Fgf4a	-	--&	-
Bos taurus FGF4A	-	--&	-
Vicugna pacos FGF4A	-	--&	-
Pteropus vampyrus FGF4A	-	--&	-
Homo sapiens FGF4B	-	--&	-
Pan troglodytes FGF4B	-	--&	-
Gorilla gorilla FGF4B	-	--&	-
Nomascus leucogenys FGF4B	-	--&	-
Callithrix jacchus FGF4B	-	--&	-
Otolemur garnettii FGF4B	-	--&	-
Mus musculus Fgf4b	-	--&	-
Rattus norvegicus Fgf4b	-	--&	-
Oryctolagus cuniculus FGF4B	-	--&	-
Tursiops truncatus FGF4B	-	--&	-
Equus caballus FGF4B	-	--&	-
Canis lupus familiaris FGF4B	-	--&	-
Myotis lucifugus FGF4B	-	--&	-
Pteropus vampyrus FGF4B	-	--&	-
Dasypus novemcinctus FGF4B	-	--&	-
Loxodonta africana FGF4B	-	--&	-
Homo sapiens FGF4C	-	--&	-
Pan troglodytes FGF4C	-	--&	-
Gorilla gorilla FGF4C	-	--&	-
Pongo abelii FGF4C	-	--&	-
Nomascus leucogenys FGF4C	-	--&	-
Otolemur garnettii FGF4C	-	--&	-
Tupaia belangeri FGF4C	-	--&	-
Mus musculus Fgf4c	-	--&	-
Cavia porcellus FGF4C	-	--&	-
Bos taurus FGF4C	-	--&	-
Canis lupus familiaris FGF4C	-	--&	-
Myotis lucifugus FGF4C	-	--&	-
Pteropus vampyrus FGF4C	-	--&	-
Dasypus novemcinctus FGF4C	-	--&	-
Homo sapiens FGF5A	-	--&	-
Pan troglodytes FGF5A	-	--&	-
Nomascus leucogenys FGF5A	-	--&	-
Macaca mulatta FGF5A	-	--&	-
Callithrix jacchus FGF5A	-	--&	-
Otolemur garnettii FGF5A	-	--&	-
Tupaia belangeri FGF5A	-	--&	-
Mus musculus Fgf5a	-	--&	-
Rattus norvegicus Fgf5a	-	--&	-
Cavia porcellus FGF5A	-	--&	-
Oryctolagus cuniculus FGF5A	-	--&	-
Myotis lucifugus FGF5A	-	--&	-
Dasypus novemcinctus FGF5A	-	--&	-
Loxodonta africana FGF5A	-	--&	-
Homo sapiens FGF5B	-	--&	-
Nomascus leucogenys FGF5B	-	--&	-
Macaca mulatta FGF5B	-	--&	-
Callithrix jacchus FGF5B	-	--&	-
Mus musculus Fgf5b	-	--&	-
Rattus norvegicus Fgf5b	-	--&	-
Cavia porcellus FGF5B	-	--&	-
Oryctolagus cuniculus FGF5B	-	--&	-
Bos taurus FGF5B	-	--&	-
Vicugna pacos FGF5B	-	--&	-

```
Equus caballus FGF5B            -  -------------------------------------------------------------&   -
Canis lupus familiaris FGF5B    -  -------------------------------------------------------------&   -
Myotis lucifugus FGF5B          -  -------------------------------------------------------------&   -
Sorex araneus FGF5B             -  -------------------------------------------------------------&   -
Dasypus novemcinctus FGF5B      -  -------------------------------------------------------------&   -
Loxodonta africana FGF5B        -  -------------------------------------------------------------&   -
Homo sapiens FGF5C            219  -NLEPSHVQA--S---RL--GSQLEASAH--------------------------------& 239
Pongo abelii FGF5C            219  -SLEPSYVQA-P----RL--GSQLEASAH--------------------------------& 239
Macaca mulatta FGF5C          219  G-LEPSHVQA-P----RL--GSQLEASAH--------------------------------& 239
Mus musculus Fgf5c            222  GKMETLSTRATPS--TQL--HTGGLAVA---------------------------------& 245
Rattus norvegicus Fgf5c       222  GKMEHLPTKATTS--AQL--DTGGLAMA---------------------------------& 245
Cavia porcellus FGF5C         222  RGQEPLHVQAT-----------MGL------------------------------------& 235
Bos taurus FGF5C              216  MRQRRRRQQRRPR--DR---DRGGRA-----------------------------------& 236
Homo sapiens FGF5D              -  -------------------------------------------------------------&   -
Macaca mulatta FGF5D            -  -------------------------------------------------------------&   -
Papio hamadryas FGF5D           -  -------------------------------------------------------------&   -
Otolemur garnettii FGF5D        -  -------------------------------------------------------------&   -
Mus musculus Fgf5d              -  -------------------------------------------------------------&   -
Rattus norvegicus Fgf5d         -  -------------------------------------------------------------&   -
Bos taurus FGF5D                -  -------------------------------------------------------------&   -
Myotis lucifugus FGF5D          -  -------------------------------------------------------------&   -
Pteropus vampyrus FGF5D         -  -------------------------------------------------------------&   -
Homo sapiens FGF6A            206  -PA----------------------------------------------------------& 207
Mus musculus Fgf6a            206  -PG----------------------------------------------------------& 207
Cavia porcellus FGF6A         206  -PG----------------------------------------------------------& 207
Bos taurus FGF6A              206  -PG----------------------------------------------------------& 207
Canis lupus familiaris FGF6A  206  -PG----------------------------------------------------------& 207
Homo sapiens FGF6B            237  -PLT---------------------------------------------------------& 239
Pan troglodytes FGF6B         237  -PLT---------------------------------------------------------& 239
Nomascus leucogenys FGF6B     237  -PLT---------------------------------------------------------& 239
Macaca mulatta FGF6B          331  -PLT---------------------------------------------------------& 333
Mus musculus Fgf6b            274  -SQT---------------------------------------------------------& 276
Rattus norvegicus Fgf6b       273  -SQT---------------------------------------------------------& 275
Cavia porcellus FGF6B         276  -PLT---------------------------------------------------------& 278
Canis lupus familiaris FGF6B  241  -PLT---------------------------------------------------------& 243
Felis catus FGF6B             283  -PLT---------------------------------------------------------& 285
Myotis lucifugus FGF6B        211  -PRT---------------------------------------------------------& 213
Dasypus novemcinctus FGF6B    172  -PLT---------------------------------------------------------& 174
Loxodonta africana FGF6B      230  -PIT---------------------------------------------------------& 232
Homo sapiens FGF6C            331  -PR----------------------------------------------------------& 332
Nomascus leucogenys FGF6C     202  -PR----------------------------------------------------------& 203
Macaca mulatta FGF6C          202  -PR----------------------------------------------------------& 203
Papio hamadryas FGF6C         202  -PR----------------------------------------------------------& 203
Mus musculus Fgf6c            202  -PR----------------------------------------------------------& 203
Rattus norvegicus Fgf6c       202  -PR----------------------------------------------------------& 203
Bos taurus FGF6C              235  -PR----------------------------------------------------------& 236
Homo sapiens FGF7A              -  -------------------------------------------------------------&   -
Macaca mulatta FGF7A            -  -------------------------------------------------------------&   -
Callithrix jacchus FGF7A        -  -------------------------------------------------------------&   -
Mus musculus Fgf7a              -  -------------------------------------------------------------&   -
Rattus norvegicus Fgf7a         -  -------------------------------------------------------------&   -
Cavia porcellus FGF7A           -  -------------------------------------------------------------&   -
Canis lupus familiaris FGF7A    -  -------------------------------------------------------------&   -
Procavia capensis FGF7A         -  -------------------------------------------------------------&   -
Homo sapiens FGF7B              -  -------------------------------------------------------------&   -
Pan troglodytes FGF7B           -  -------------------------------------------------------------&   -
Pongo abelii FGF7B              -  -------------------------------------------------------------&   -
Nomascus leucogenys FGF7B       -  -------------------------------------------------------------&   -
Macaca mulatta FGF7B            -  -------------------------------------------------------------&   -
Papio hamadryas FGF7B           -  -------------------------------------------------------------&   -
Callithrix jacchus FGF7B        -  -------------------------------------------------------------&   -
Mus musculus Fgf7b              -  -------------------------------------------------------------&   -
Rattus norvegicus Fgf7b         -  -------------------------------------------------------------&   -
Cavia porcellus FGF7B           -  -------------------------------------------------------------&   -
Oryctolagus cuniculus FGF7B     -  -------------------------------------------------------------&   -
Tursiops truncatus FGF7B        -  -------------------------------------------------------------&   -
Bos taurus FGF7B                -  -------------------------------------------------------------&   -
Equus caballus FGF7B            -  -------------------------------------------------------------&   -
Canis lupus familiaris FGF7B    -  -------------------------------------------------------------&   -
Myotis lucifugus FGF7B          -  -------------------------------------------------------------&   -
Loxodonta africana FGF7B        -  -------------------------------------------------------------&   -
Homo sapiens FGF8A            185  --------------S-DMFS-SPLETDSMDPFGLVTGLE-AV-----------RS-PSFEK& 216
Pongo abelii FGF8A            185  --------------S-DMFS-SPLETDSMDPFGLVTGLE-AV-----------RS-PSFEK& 216
Macaca mulatta FGF8A          185  --------------S-DMFS-SPLETDSMDPFGLVTGLE-AV-----------RS-PSFEK& 216
Microcebus murinus FGF8A      266  --------------S-DMFA-SPLETDSMDPFGIATRLG-VV-----------KS-PSFQK& 297
Otolemur garnettii FGF8A      188  --------------S-DMFS-LPLETDSMDPFGIATRLG-VV-----------KS-PSFQK& 219
Mus musculus Fgf8a            186  --------------S-KMFS-LPLESDSMDPFRMVEDVDHLV-----------KS-PSFQK& 218
Rattus norvegicus Fgf8a       186  --------------S-KMFS-LPLESDSMDPFRMVEDVDHLV-----------KS-PSFQK& 218
Cavia porcellus FGF8A         190  --------------S-SVFS-LPLKTDSMDPFGMASEIG-LV-----------KS-PSFQK& 221
```

```
Bos taurus FGF8A              188 --------------P-D-FF-LPLKTDSMDPFGLATKLG-SV-----------KS-PSFYN& 218
Myotis lucifugus FGF8A        188 --------------P-DEFS-SPLETDSMDPFGIANNLR-LV-----------RS-PSFQE& 219
Pteropus vampyrus FGF8A       185 --------------A-DTFS-SPLETDDMDPFGIASKLG-LE-----------ES-PSFQK& 216
Dasypus novemcinctus FGF8A    186 --------------P-GVFS-QPLEIESMDPFGIASGLG-LV-----------KS-PSFQK& 217
Homo sapiens FGF8B            323 -PRARMT-PAPASCSQELPSAEDNSPMASDPLGVVRGGR-VNTHAGGTGPEGCRPFAKFI-& 379
Pan troglodytes FGF8B         323 -PRARMT-PAPASCSQELPSAEDNSPMASDPLGVVRGGR-VNTHAGGTGPEGCRPFPKFI-& 379
Nomascus leucogenys FGF8B     323 -PRARMT-PAPASCSQELLSSEDNSPMASDPLGVVRGGR-VNTHAGGTGPEGCRPFPKFI-& 379
Otolemur garnettii FGF8B      196 -PRPRAT-PGPASCSQELPSAEDNSLVASDPLGVVRGNR-VNAHAGRAGLDRCRPFPRYF-& 252
Mus musculus Fgf8b            195 -PRPRAT-PVPVSCSRELPSAEEGGPAASDPLGVLRRGR-GDARGGAGGADRCRPFPRFV-& 251
Rattus norvegicus Fgf8b       268 -PRPRAT-PIPVSCSRELPSAEEGGPAASDPLGVLRRGR-GDARRGAGGTDRCRPFPRFV-& 324
Cavia porcellus FGF8B         189 -LRPRAT-PAPASCSQELPSAED-AAQASDPLGVLRGAR-VHAH-GGPRPARCRPGPGAK-& 243
Oryctolagus cuniculus FGF8B   256 -PRFRLT-PAPASCSQEAPSAEDNGLVASDPFGVLRGNR-VNMHGDRMGPERCHHFPKFI-& 312
Equus caballus FGF8B          190 -PRPRMT-PAPASCSQELPSAEDNSVLASDPLGVVRGNR-VNTHAGGAGVERCRPFPKFF-& 246
Canis lupus familiaris FGF8B  256 -PRPRLA-PAPASCSQELPSAEDPGAPASDPLGVLRGHR-ANARAGGVGVDRCRAFPTPI-& 312
Dasypus novemcinctus FGF8B    192 -PRLRLT-PAPASCSQELPSDEDDGAVASDPLRVVLGRR-PHARAAGAGGERCRPGPQLS-& 248
Loxodonta africana FGF8B      254 -PRPRMT-PAPASCSQELLSAEDNSVVANDPLGVVRSNR-VNTHAGGIGVERCRPFPKFI-& 310
Homo sapiens FGF8C            184 ---------------------QPPDVGSSDPLSMV-GPS-QG-----------RS-PSYAS& 209
Pan troglodytes FGF8C         184 ---------------------QPPDVGSSDPLSMV-GPS-QG-----------RS-PSYTS& 209
Pongo abelii FGF8C            184 ---------------------QPPDVGSSDPLSMV-GPS-QG-----------RS-PSYAS& 209
Nomascus leucogenys FGF8C     184 ---------------------QPPDVGSSDPLSMV-GPS-QG-----------RS-PSYAS& 209
Otolemur garnettii FGF8C      186 ---------------------EPPDVGSSDPLSMV-GPS-QG-----------RS-PSYAS& 211
Mus musculus Fgf8c            185 ---------------------EPPDVGSSDPLSMV-EPL-QG-----------RS-PSYAS& 210
Rattus norvegicus Fgf8c       183 ---------------------EPPDVGSSDPLSMV-EPL-QG-----------RS-PSYAS& 208
Cavia porcellus FGF8C         190 ---------------------GPPDVGSSDPLSMV-GPL-WD-----------RS-PSYTS& 215
Oryctolagus cuniculus FGF8C   184 ---------------------APPDVDSPDPLSMV-QPA-LD-----------QS-PSYTS& 209
Ochotona princeps FGF8C       178 ---------------------EPPDVDSPDPLSMV-GPL-QG-----------QS-PSYTS& 203
Bos taurus FGF8C              184 ---------------------EPPDVGSSDPLSMV-GPS-YG-----------RS-PSYTS& 209
Equus caballus FGF8C          184 ---------------------EPPDVGSSDPLSMV-GPS-RS-----------RS-PSYTS& 209
Canis lupus familiaris FGF8C  184 ---------------------EPPDVGSSDPLSMV-GPS-QG-----------RS-PSYAS& 209
Felis catus FGF8C             185 ---------------------EPPDVGSSDPLSMV-GAS-QG-----------RS-PSYAS& 210
Sorex araneus FGF8C           185 ---------------------VPPDVGSSDPLSMV-GPP-ER-----------HS-PSYTS& 210
Procavia capensis FGF8C       184 ---------------------EPPDVDSSDPLSMV-GPS-QG-----------RS-PSYAS& 209
```
